# Supplementary material for: Xanthomonas immunity proteins protect against the cis-toxic effects of their cognate T4SS effectors
Source: EMBO Rep. 2024 Feb 8;25(3):27. doi: 10.1038/s44319-024-00060-6 (PMC10933484; doi:10.1038/s44319-024-00060-6)
Supplement: Supplementary file 1 — Appendix [file 44319_2024_60_MOESM1_ESM.pdf]

APPENDIX

for

The protective function of an immunity protein against the *cis*-toxic effects of a *Xanthomonas* Type IV Secretion System Effector

Gabriel U. Oka[1,2], Diorge P. Souza[1,3], Germán G. Sgro[1,4], Cristiane R. Guzzo[5], German Dunger[1,6] and Chuck S. Farah[1]*

Table of Contents

Appendix Table S1…. page 2

Appendix Table S2…. page 2

Appendix Table S3…. pages 3-4

Appendix Table S4…. page 4

Appendix Table S5…. page 5

Appendix Figure S1…. page 6

Appendix Figure S2…. pages 7-8

Appendix Figure S3…. page 9

Appendix Figure S4…. page 10

Appendix Figure S5…. page 11

Appendix Figure S6…. page 12

Appendix Figure S7…. page 13

Appendix Figure S8…. pages 14-15

APPENDIX TABLES

Appendix Table S1. Analysis of cellular propidium iodide permeability (PIP) in Movies EV1-5*

	Total cells counted at beginning of movie A/ movie B	PIP events counted during movies movie A/ movie B	% of cells undergoing PIP events movie A/ movie B	Final % of cells undergoing PIP events
**WT**	**991 / 1024**	**11 / 6**	**1.1% / 0.6%**	**0.8%**
**ΔX-Tfi^{XAC2610}**	**549 / 595**	**72 / 54**	**13.1% / 9.0%**	**11.0%**
**ΔX-Tfe^{XAC2609}ΔX-Tfi^{XAC2610}**	**446 / 730**	**2 / 1**	**0.5% / 0.13 %**	**0.3%**
**ΔX-Tfe^{XAC2609}ΔX-Tfi^{XAC2610} + X-Tfe^{XAC2609}NT**	**440 / 583**	**85 / 165**	**19.3 % / 28. 3%**	**23.8%**
**ΔX-Tfi^{XAC2610}Δ*virD4***	**530 / 582**	**41 / 68**	**7.7 % / 11.7 %**	**9.7%**

*Each movie presents two consecutive experimental sessions, here indicated as movies A and B. Counted values read for each session are separated by a backslash (A/B).

Appendix Table S2. Analysis TEM micrographs of *X. citri* strains.

	WT	ΔvirB7	ΔX-Tfi^{XAC2610}	ΔX-Tfi^{XAC2610}C	ΔX-Tfi^{XAC2610} ΔVirB7
**total number of cells counted**	**2640**	**675**	**2152**	**1212**	**1337**
**micrographs analyzed**	**15**	**5**	**10**	**8**	**10**
***mean of cells per micrograph**	**176 +/- 11.7**	**135 +/- 20.9**	**215 +/- 17.9**	**151 +/- 9.8**	**166 +/- 7.8**
**total of damaged cells identified**	**3**	**1**	**39**	**4**	**15**
**mean of damaged cells per micrograph***	**0.2 +/- 0.1**	**0.2 +/- 0.2**	**3.9 +/- 0.76**	**0.5 +/- 0.5**	**1.5 +/- 0.45**
**percentage of damaged cells per micrograph***	**0.11% +/- 0.06%**	**0.15% +/- 0.15%**	**1.95% +/- 0.39%**	**0.4% +/- 0.4%**	**0.88% +/- 0.25%**

*all values are mean (+/- SE)

Appendix Table S3. Oligonucleotides used in this study.

Oligonucleotides	Description (Reference)	Sequence 5' - 3'
F_UP2610	Forward primer to amplify the upstream region of *xac2610* for construction of the pNPTS138-xac2610 (This study).	TTCTGGATCCGGCCTCTGTTCATCCTGTGT
R_UP_2610	Reverse primer to amplified the upstream region of *xac2610* for construction of the pNPTS138-xac2610 (This study)	TACATCCATGGACTGCAAGCTGTGATCGAGA
F_DOWN_2610	Forward primer to amplified the downstream region of *xac2610* or construction of the pNPTS138-xac2610 (This study)	TACATCCATGGACCGAAGACACGAATGATCC
R_DOWN_2610	Reverse primer to amplified the downstream region of *xac2610* for construction of the pNPTS138-xac2610 (This study)	TTCTGGATCCGCCTCGAGCTTGCTCACT
F_UP_2609-2610	Forward primer to amplified the upstream region of *xac2610* for construction of the pNPTS138-*xac2610-xac2609* (This study)	TTCTGGATCCGGCCTCTGTTCATCCTGTGT
R_UP_2609-2610	Reverse primer to amplified the upstream region of *xac2610* for construction of the pNPTS138-*xac2610-xac2609* (This study)	TACATGAATTCACTGCAAGCTGTGATCGAGA
F_DOWN_2609_2610	Forward primer to amplified the downstream region of *xac2609* for construction of the pNPTS138-*xac2610-xac2609* (This study)	TACATGAATTCCAGACCCAGGAAAGTGCTTC
R_DOWN_2609_2610	Reverse primer to amplified the downstream region of *xac2609* for construction of the pNPTS138-*xac2610-xac2609* (This study)	TTCTGGATCCCCTAGATCCCTGTCCAGACG
F_pBRAXAC2610_*Nco*I	Forward primer for cloning of *xac2610* in pBRa vector (This study).	AAGGATCCAACCATGGTGACGCGCGAATTAGCAAGG
R_pBRAXAC2610_*Sal*I	Reverse primer for cloning of *xac2610* in pBRa vector (This study).	CCCCAAGAATTCGTCGACTTAGTTGGACACCTCCTTCATTTC
F_XAC2609-E48A	Forward primer for mutagenesis of pBRA-XAC2609Nt plasmid at codon 48	GCGACGGCTCAACATGCGACGCGTAATTTTCAG
R_XAC2609-E48A	Forward primer for mutagenesis of pBRA-XAC2609Nt plasmid at codon 48	CTGAAAATTACGCGTCGCATGTTGAGCCGTCGC
F_UP_VirB4	Forward primer to amplified the upstream region of *virB4* for construction of the pNPTS138-VirB4 (This study)	ccgaagctagcgaattcgtggatccCAGGGGCGTGGGGTGGCG
R_UP_VirB4	Reverse primer to amplified the upstream region of *virB4* for construction of the pNPTS138-VirB4 (This study)	ttgcgcttacCCAGATGCCAGATCAACAGGTAGTCGC
F_DOWN_VirB4	Forward primer to amplified the downstream region of *virB4* for construction of the pNPTS138-VirB4 (This study)	tggcatctggGTAAGCGCAAGTCGGCCAAG
R_DOWN_VirB4	Reverse primer to amplified the downstream region of *virB4* for construction of the pNPTS138-VirB4 (This study)	gcttctctgcaggatatctggatccAACTCCCACTGGAAGATCGC
F_UP_B5	Forward primer to amplified the upstream region of *virB5* for construction of the pNPTS138-VirB5 (This study)	ATAGGATCCTTCGTGCGCGCGTGC
R_UP_B5	Reverse primer to amplified the upstream region of *virB5* for construction of the pNPTS138-VirB5 This study)	TTGTCGATCTCGTGCCTGGACTGACTATGC
F_DOWN_B5	Forward primer to amplified the downstream region of *virB5* for construction of the pNPTS138-VirB5 (This study)	TCCAGGCAC*GAGATCGACAAGGACTGATG*
R_DOWN_B5	Reverse primer to amplified the downstream region of *virB5* for construction of the pNPTS138-VirB5 (This study)	GTGGGATCCGCAGAATACGCCGTGAACT
F_UP_VirB6	Forward primer to amplified the upstream region of *virB6* (This study)	ccgaagctagcgaattcgtggatccAATGGTTGGCCGATTTCTATG
R_UP_VirB6	Reverse primer to amplified the upstream region of *virB6* (This study)	tacctgaactGTAGTTGTTGATCAGACGAAAAAATTC
F_DOWN_VirB6	Forward primer to amplified the downstream region of *virB6* (This study)	caacaactacAGTTCAGGTAATCGTGGG
R_DOWN_VirB6	Reverse primer to amplified the downstream region of *virB6* (This study)	gcttctctgcaggatatctggatccATCGGTCAAAATGATTGAG
F_UP_VirB8	Forward primer to amplify the upstream region of *virB8* (This study)	AGTCACTAGTCACCCATAGCGTGTCAGAAG
R_UP_VirB8	Reverse primer to amplify the upstream region of *virB8* (This study)	AGTCGATATCCAACATGTCTAGATCCGTCACC
F_DOWN_VirB8	Forward primer to amplify the downstream region of *virB8* (This study)	AGTCGATATCATGCCCAAGAATGTCAATGG
R_DOWN_VirB8	Reverse primer to amplify the downstream region of *virB8* (This study)	AGTCGGATCCATGGCTGCAAGAAACACCAG
F_UP_VirB9	Forward primer to amplify the upstream region of *virB9* (Sgro *et al.*, 2018)	AGTCACTAGTAGCTCTAAGGGCTGCCGTC
R_UP_VirB9	Reverse primer to amplify the upstream region of *virB9* (Sgro *et al.*, 2018)	AGTCGATATCGTTAAAAAGTTTCATCGACCACTTG
F_DOWN_VirB9	Forward primer to amplify the downstream region of *virB9* (Sgro *et al.*, 2018)	AGTCGATATCAACGTCGTCGGTCTGCG
R_DOWN_VirB9	Reverse primer to amplify the downstream region of *virB9* (Sgro *et al.*, 2018)	AGTCGGATCCGACGTTCAACACCGTTCCTTG

F_UP_VirB10	Forward primer to amplify the upstream region of *virB10* (Sgro *et al.*, 2018)	AGTCACTAGTACGATGGGGTTCGTCTACC
R_UP_VirB10	Reverse primer to amplify the upstream region of *virB10* (Sgro *et al.*, 2018)	AGTCGATATCAATATTCGAATTCACTTTTGCTTG
F_DOWN_VirB10	Forward primer to amplify the downstream region of *virB10* (Sgro *et al.*, 2018)	AGTCGATATCGTCCTGCCGAAGTGAATG
R_DOWN_VirB10	Reverse primer to amplify the downstream region of *virB10* (Sgro *et al.*, 2018)	AGTCGGATCCCCTTGATGTGAACCACGATG
F_2610-Y170A	Forward primer for mutagenesis of pET28a-HisXAC260 Y170A	agagggaccaccagcattaccctcttttccagaccacacc
R_2610-Y170A	Reverse primer for mutagenesis of pET28a-HisXAC260 Y170A	ggtgtggtctggaaaagagggtaatgctggtggtccctct

Appendix Table S4. Plasmids used in this study

Plasmids	Properties	References
pBRA	pBAD24 derivative vector which has an arabinose-inducible promoter that is constitutively active plasmid in Xac; Sp^r	M.Marroquin,unpublished
pBRA-XAC2609Nt	pBRA encoding X-Tfe^{XAC2609} N-terminus (residues 1-306); Sp^r	(Souza *et al*, 2015)
pBRA-XAC2609NtE48A	pBRA-XAC2609Nt with a point mutation at codon 48; Sp^r	This study
pBRA-2609FL	pBRA encoding X-Tfe^{XAC2609} full length (residues 1-431); Sp^r	(Souza *et al*, 2015)
pBRA-XAC2610	pBRA encoding X-Tfi^{XAC2610} ; Sp^r	This study
pBRA-VirB7	pBRA encoding VirB7	(Souza *et al*, 2015)
pNPTS138	Suicide vector for generation of gene knockouts (*sacB*), Km^r	M.R.Alley unpublished
pNPTS138-*xac2610*	Suicide vector for generation of the *X. citri* ΔX-Tfi^{XAC2610}, and doubles delection of ΔX-Tfi^{XAC2610} and ΔVir(B4-B10,D4) ΔX-Tfi^{XAC2610} strains.	This study
pNPTS138-*xac2610-xac2609*	Suicide vector for generation of the *X. citri*ΔX-Tfe^{XAC2609} ΔX-Tfi^{XAC2610} strain; Km^r	This study
pBBR-5GFP	Broad host range plasmid cloning vector pBBR1MCS-5 expressing GFP; Gm^r	(Dunger *et al*, 2014)
pBBR-2RFP	Broad host range plasmid cloning vector pBBR1MCS-5 expressing RFP; Gm^r	(Oka *et al*, 2022)
pET28a-XAC2610His-22-267	Production of the His-tagged HIS-X-Tfi^{XAC2610}(22-267)	(Alegria *et al*, 2005)
pET28a-XAC2610His-22-267Y170A	Production of the His-tagged HIS-X-Tfi^{XAC2610}(22-267) Y170A	This study
pET11a-XAC2609(1-308)	Eptopic Production of the X-Tfe^{XAC2609}(1-308)	(Souza *et al*, 2015)
pET11a-XAC2610(55-267)	Production of the XTfi^{XAC2610} folded domain (residues 55-267)	(Souza *et al*, 2015)
pNPTS138-VirB4	Suicide vector for delection of virB4 in the *X. citri* ΔVirB4 , Km^r	This study
pNPTS138-VirB5	Suicide vector for delection of virB5 in the *X. citri* ΔVirB5 , Km^r	This study
pNPTS138-VirB6	Suicide vector for delection of virB6 in the *X. citri* ΔVirB6, Km^r	This study
pNPTS138-VirB7	Suicide vector for delection of virB7 in the *X. citri* in ΔVirB7 Δ/ Km^r	(Souza *et al*, 2011)
pNPTS138-VirB8	Suicide vector for delection of *virB8* in *X. citri* ΔVirB8, Km^r	This study
pNPTS138-VirB9	Suicide vector for delection of *virB9* in *X. citri* ΔVirB9, Km^r	(Sgro *et al*, 2018)
pNPTS138-VirB10	Suicide vector for delection of *virB10* in *X. citri* ΔVirB10 Km^r	(Sgro *et al*, 2018)
pNPTS138-VirD4	Suicide vector for delection of *virD4* in *X. citri* ΔVirD4 , Km^r	(Souza et al, 2015)

Appendix Table S5. Bacterial strains used in this study

Bacterial strains	Genotype	**reference**
X. citri wild type	*Xanthomonas citri* subsp. *citri* strain 306, wild type strain; Ap^r	(da Silva *et al*, 2002)
X. citri Δ*virB7*	*X. citri* Δ*virB7 XAC2622*; Ap^r	(Souza *et al*, 2015)
X. citri ΔX-Tfi^{XAC2610}	*X. citri* Δ*xac2610*, Ap^r	This study
X. citri wild type pBRA GFP	*X. citri* wild type pBRA pBBR-5GFP; Ap^r Gm^r	(Souza *et al*, 2015)
X. citri Δ*virD4* GFP	*X. citri* Δ*virD4XAC2623* pBBR-5GFP; Ap^r	(Souza *et al*, 2015)
X. citri ΔX-Tfi^{XAC2610} pBRA	*X. citri* Δ*xac2610* pBRA; Ap^r Sp^r	This study
X. citri ΔX-Tfi^{XAC2610} pBRA GFP	*X. citri* Δ*xac2610* pBRA pBBR-5GFP; Ap^r Sp^r Gm^r	This study
X. citri ΔX-Tfi^{XAC2610}+ X-Tfi^{XAC2610}	*X. citri* Δ*xac2610* pBRA-XAC2610; Ap^r Sp^r	This study
X. citri ΔX-Tfi^{XAC2610} + X-Tfi^{XAC2610} GFP	*X. citri* Δ*xac2610* pBRA-XAC2610 pBBR-5GFP; Ap^r Sp^r Gm^r	This study
X. citri ΔX-Tfi^{XAC2610}ΔVirB7	*X. citri* Δ*virB7XAC2622* Δ*xac2610;* Ap^r	This study
X. citri ΔX-Tfi^{XAC2610}ΔVirB7 pBRA	*X. citri* Δ*virB7XAC2622* Δ*xac2610* pBRA*;* Ap^r Sp^r	This study
X. citri ΔX-Tfi^{XAC2610}ΔVirB7 pBRA GFP	*X. citri* Δ*virB7XAC2622* Δ*xac2610* pBRA pBBR-5GFP*;* Ap^r Sp^r Gm^r	This study
X. citri ΔX-Tfi^{XAC2610}ΔVirB7 + X-Tfi^{XAC2610}	*X. citri* Δ*virB7XAC2622* Δ*xac2610* pBRA-XAC2610*;* Ap^r Sp^r	This study
X. citri ΔX-Tfi^{XAC2610}ΔVirB7 + X-Tfi^{XAC2610} GFP	*X. citri* Δ*virB7XAC2622* Δ*xac2610* pBRA-XAC2610 pBBR-5GFP*;* Ap^r Sp^r Gm^r	This study
X. citri ΔX-Tfi^{XAC2610}Δ*virD4* GFP	*X. citri* Δ*virD4XAC2623* Δ*xac2610* pBBR-5GFP*;* Ap^r Gm^r	This study
X. citri ΔX-Tfi^{XAC2610}ΔTfe^{XAC2609}	*X. citri* Δ*xac2610*Δ*xac2609;* Ap^r	This study
X. citri ^{0}ΔX-Tfe^{XAC2609}ΔX-Tfi^{XAC261} pBRA	*X. citri* Δ*xac2610*Δ*xac2609* pBRA*;* Ap^r Sp^r	This study
X. citri ΔX-Tfi^{XAC2610}ΔX-Tfe^{XAC2609} pBRA GFP	*X. citri* Δ*xac2610*Δ*xac2609* pBRA pBBR-5GFP; Ap^r Sp^r Gm^r	This study
X. citri ΔX-Tfe^{XAC2609}ΔX-Tfi^{XAC2610} + X-Tfe^{XAC2609}FL GFP	*X. citri* Δ*xac2610*Δ*xac2609* pBRA-2609FL pBBR-5GFP; Ap^r Sp^r Gm^r	This study
X. citri ΔX-Tfe^{XAC2609}ΔX-Tfi^{XAC2610} + X-Tfe^{XAC2609}NT GFP	*X. citri* Δ*xac2610*Δ*xac2609* pBRA-2609Nt pBBR-5GFP; Ap^r Sp^r Gm^r	This study
X. citri ΔX-Tfe^{XAC2609}ΔX-Tfi^{XAC2610} + X-Tfe^{XAC2609NT} E48A GFP	*X. citri* Δ*xac2610*Δ*xac2609* pBRA-XAC2609NtE48A pBBR-5GFP; Ap^r Sp^r Gm^r	This study
X. citri Δ*virB4*	*X. citri* Δ*virB4 XAC2614*; Ap^r	This study
X. citri Δ*virB5*	*X. citri* Δ*virB5 XAC2613*; Ap^r	This study
X. citri Δ*virB6*	*X. citri* Δ*virB6 XAC2612*; Ap^r	This study
X. citri Δ*virB8*	*X. citri* Δ*virB8 XAC2621*; Ap^r	(Sgro *et al*, 2018)
X. citri Δ*virB9*	*X. citri* Δ*virB9 XAC2620*; Ap^r	(Sgro *et al*, 2018)
X. citri Δ*virB10*	*X. citri* Δ*virB10 XAC2619*; Ap^r	(Sgro *et al*, 2018)
X. citri ΔX-Tfi^{XAC2610} Δ*virB4*	*X. citri* Δ*virB4 XAC2614*; Δ*xac2610*; Ap^r	This study
X. citri ΔX-Tfi^{XAC2610}Δ*virB5*	*X. citri* Δ*virB5 XAC2613*; Δ*xac2610*; Ap^r	This study
X. citri ΔX-Tfi^{XAC2610}Δ*virB6*	*X. citri* Δ*virB6 XAC2612*; Δ*xac2610* Ap^r	This study
X. citri ΔX-Tfi^{XAC2610}Δ*virB8*	*X. citri* Δ*virB8 XAC2621*; Δ*xac2610*; Ap^r	This study
X. citri ΔX-Tfi^{XAC2610}Δ*virB9*	*X. citri* Δ*virB9 XAC2620*; Δ*xac2610*; Ap^r	This study
X. citri ΔX-Tfi^{XAC2610}Δ*virB10*	*X. citri* Δ*virB10 XAC2619*; Δ*xac2610*; Ap^r	This study
E. coli MG1655		
X. citri Δ8Δ2609-GFP	*X. citri strain strain deleted of the X-Tfe/i*$^{XAC2885/XAC2884,}$ *X-Tfe/i*$^{XAC0574/XAC0573,}$ *X-Tfe/i*$^{XAC0096/XAC0097,}$ *X-Tfe/i*$^{XAC3634/XAC3633}$, *X-Tfe/i*$^{XAC0466/XAC047}$,*X-Tfe/i*$^{XAC3266/XAC3267}$,*X-Tfe/i*$^{XAC4264//xac42633/XAC4262}$ *with X-Tfe*^{XAC2609} *substituted by msGFP*; Ap^r	*(Oka et al, 2022)*
X. citri Δ8Δ2609-GFP kan^R	*X. citri strain Δ8Δ2609-GFP*+ *pBBR-2RFP*; *Ap*^{R}, *Km*^{R}	This study
X. citri ΔX-Tfe^{XAC2609}ΔX-Tfi^{XAC2610} kan^R	*X. citri* ΔX-Tfe^{XAC2609}ΔX-Tfi^{XAC2610} + pBBR-2RFP; *Ap*^{R}, *kan*^{R}	This study
X. citri Δ*virB7 kan*^{R}	*X. citri* Δ*virB7* + pBBR-2RFP; *Ap*^{R}, *kan*^{R}	This study
X. citri wild-type *kan*^{R}	*X. citri* wild-type + pBBR-2RFP; *Ap*^{R}, *kan*^{R}	This study
X. citri wild-type *gent*^{R}	*X. citri* Wild-type + pBBR-5GFP; *Ap*^{R}, *gent*^{R}	This study

APPENDIX FIGURES

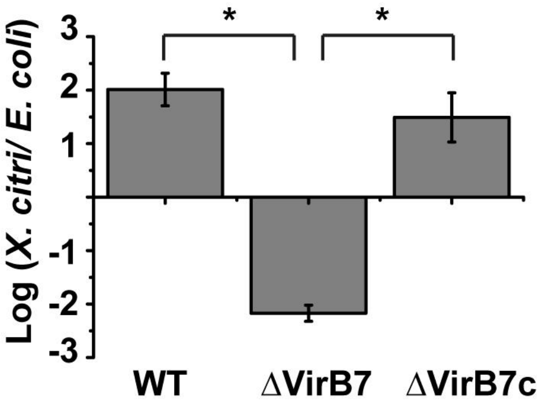

Appendix Figure S1. Inter-species bacterial competition assay.

Ratio of the number of viable *X. citri* and *E. coli* Bl21(DE3) cells after 48 hours of co-culture on Luria-Bertani (LB) agar at 28 ºC. *X. citri* strains: WT (wild type carrying the empty pBRA vector), ΔVirB7 (*virB7* knockout strain carrying the empty pBRA vector), ΔVirB7c (*virB7* knockout carrying the plasmid pBRA-VirB7).

Data information: Asterisks (*) indicate significant differences at the *p-value* <0.001 (ANOVA). Error bars ± s.d. n=4.

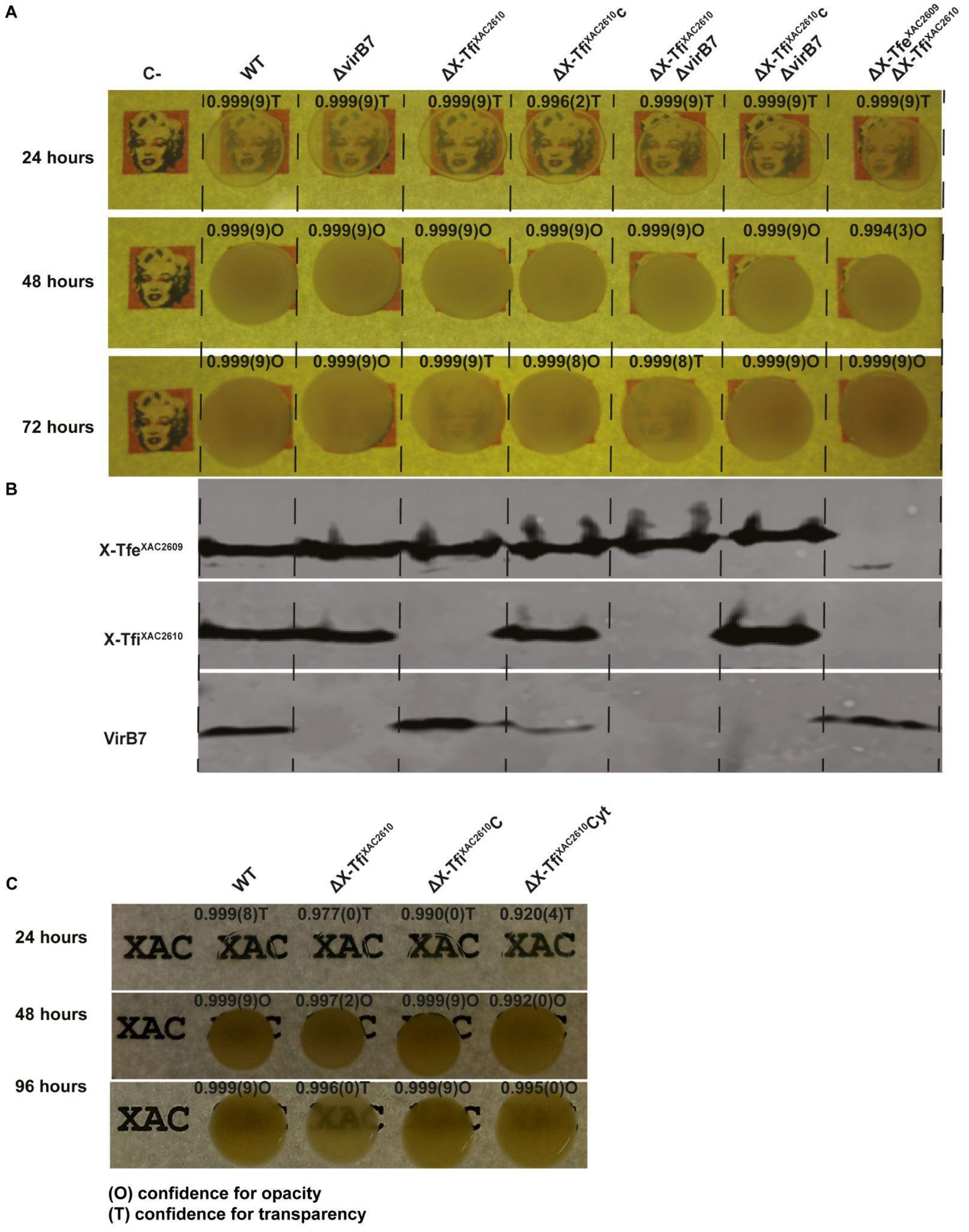

Appendix Figure S2. Colony opacity assay.

(A) *X. citri* wild-type mutant strains were grown on an LB agar plate placed above a paper sheet printed with a picture. Plate images were acquired after 24, 48 and 72 hours of growth at 28°C. *X. citri* wild-type (WT) strain, ΔVirB7 strain, ΔX-Tfi^{XAC2610} strain,

ΔX-Tfi^{XAC2610}ΔVirB7 strain, ΔX-Tfe^{XAC2609}ΔX-Tfi^{XAC2610} strain, and complemented strains (ΔX-Tfi^{XAC2610} + X-Tfi^{XAC2610} (ΔX-Tfi^{XAC2610}c), ΔX-Tfi^{XAC2610}ΔVirB7 + X-Tfi^{XAC2610} (ΔX-Tfi^{XAC2610}cΔVirB7) used are indicated above each lane. Above each colony are the convolutional neural network confidence tendency index for opacity (O) and transparency (T) as described in Materials and Methods.

(B) Immunodetection by Western Blot of X-Tfe^{XAC2609}, X-Tfi^{XAC2610}, and VirB7 of the cell extract of each *X. citri* strain used in (A).

(C) *X. citri* wild-type and derived mutants were grown on an LB agar plate placed above a paper sheet printed with the letters "XAC". Plate images were acquired after 24, 48, and 96 hours of growth. *X. citri* wild-type (WT), ΔX-Tfi^{XAC2610}, ΔX-Tfi^{XAC2610} transformed with pBRA-X-Tfi^{XAC2610} (ΔX-Tfi^{XAC2610}c), ΔX-Tfi^{XAC2610} transformed with pBRA-X-Tfi^{XAC2610}His-22-267 (ΔX-Tfi^{XAC2610}cyt).

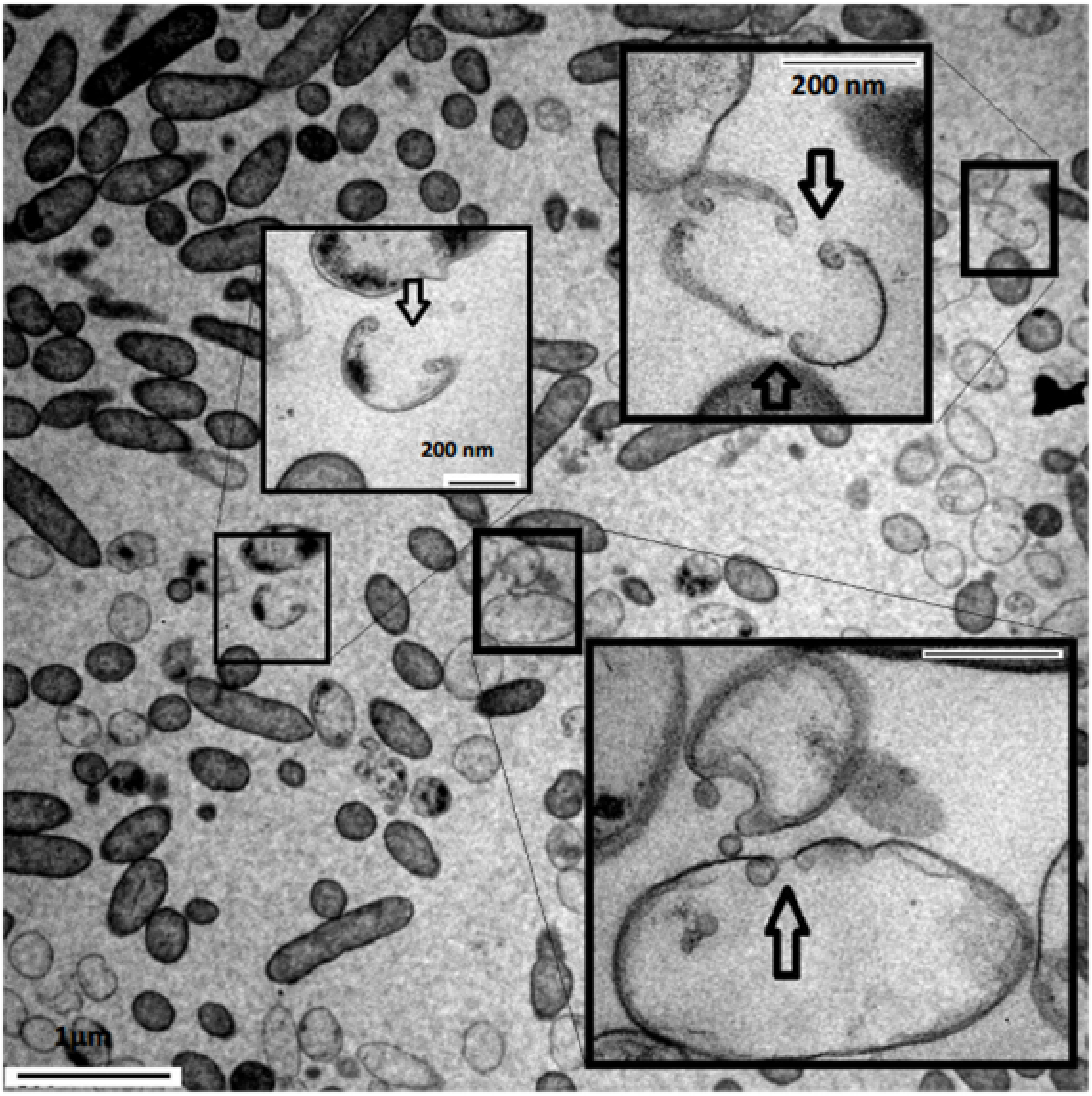

Appendix Figure S3. Transmission electron microscopy (TEM) of *X. citri* ΔX-Tfi^{XAC2610} cells.

Insets highlight cells having an impaired cellular envelope (arrows). Sample preparation is described in the Materials and Methods section.

Data information: Scale bar = 1 µm. See Appendix Table S2 for detailed information regarding total number of micrographs and cells analyzed and the total number of damaged cells identified.

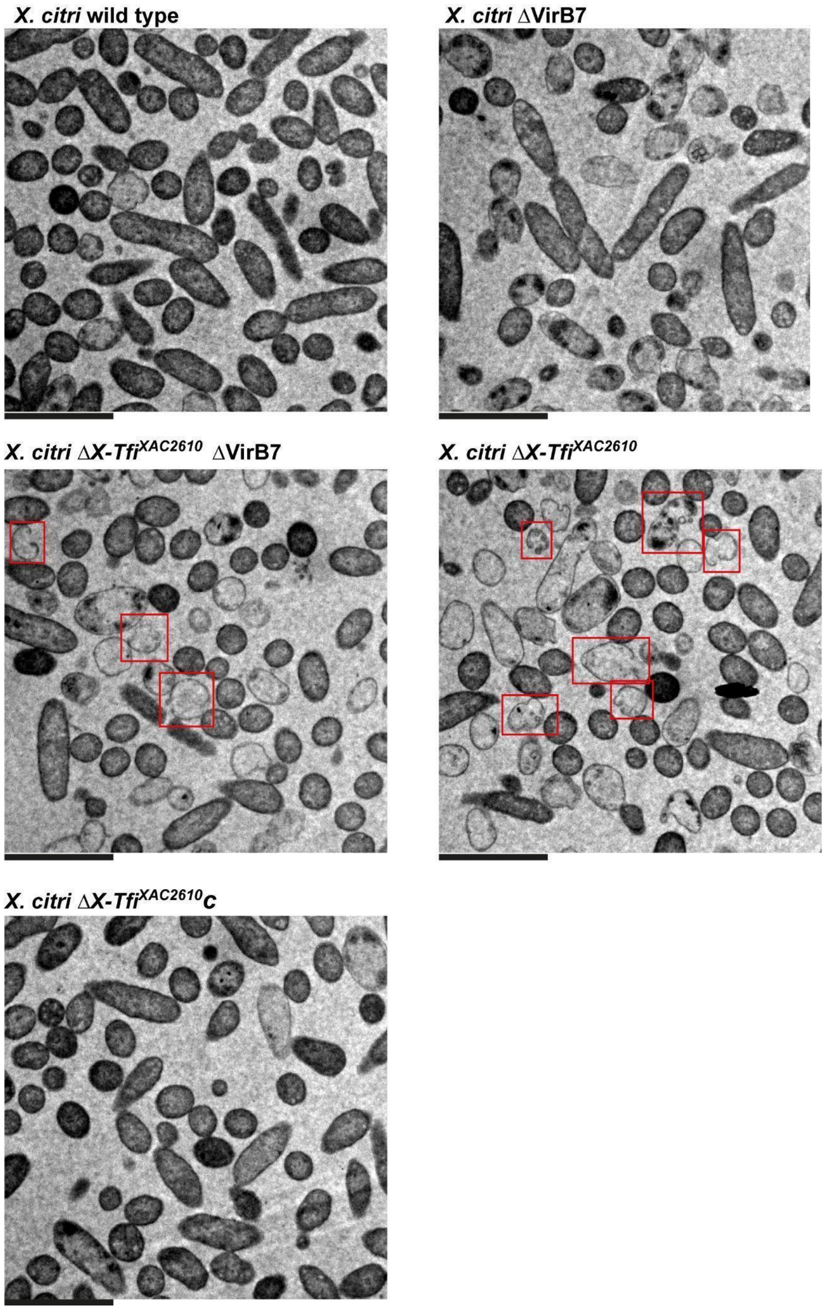

Appendix Figure S4. Transmission electron microscopy (TEM) of different X. citri strains.

Micrographs of *X. citri* wild-type, ΔX-Tfi^{XAC2610}, ΔVirB7, ΔX-Tfi^{XAC2610}-ΔVirB7 and ΔX-Tfi^{XAC2610} complemented with pBRA-XAC2610 vector (ΔX-Tfi^{XAC2610}c). Examples of damaged cells are highlighted in red rectangles. Sample preparation is described in the Materials and Methods section.

Data information: Scale bar = 1 µm. See Appendix Table S2 for detailed information regarding total number of micrographs and cells analyzed and the total number of damaged cells identified.

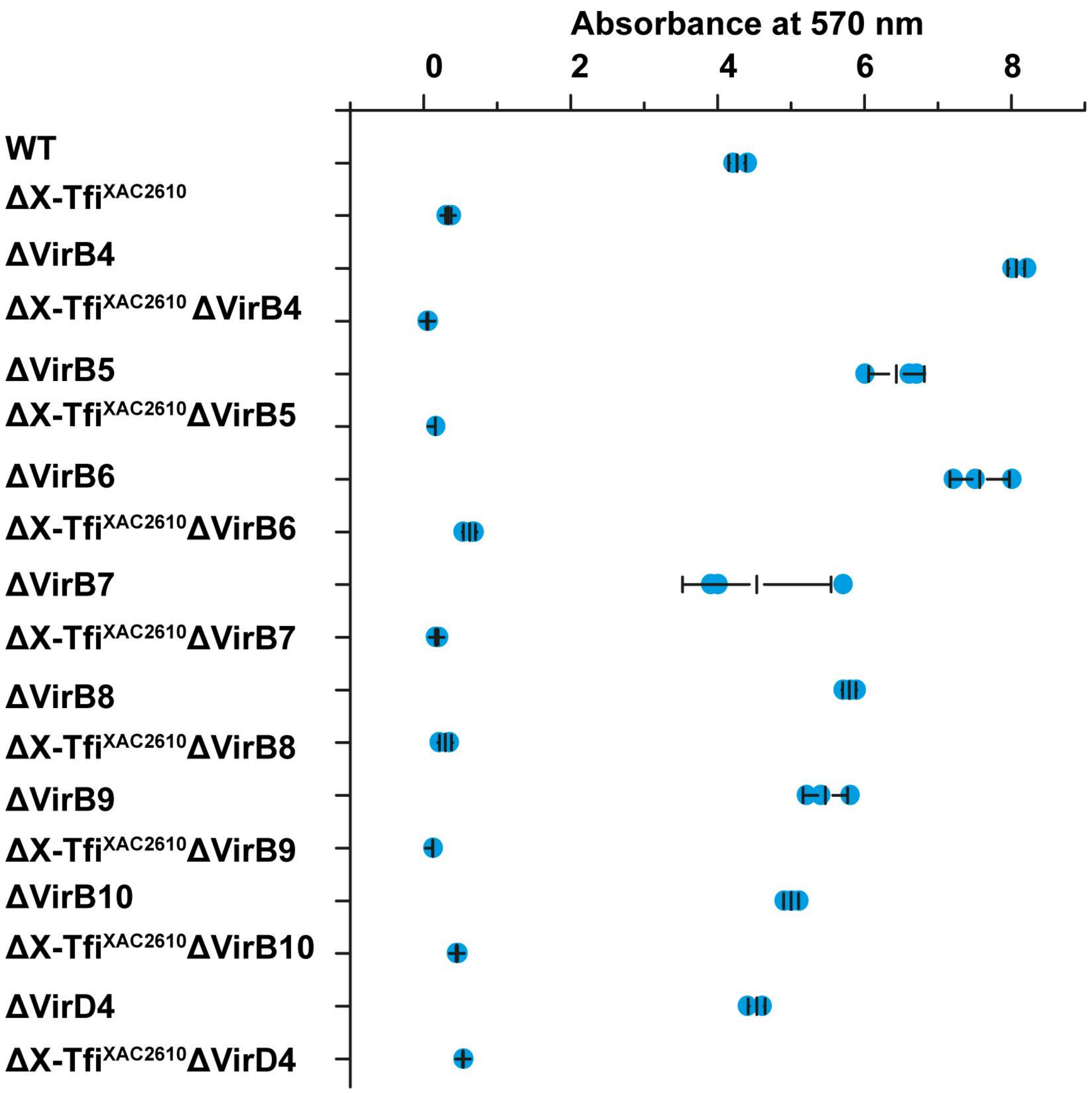

Appendix Figure S5. ***X. citri*** **biofilm quantification using crystal violet.**

X. citri strains used in the assay are indicated in the Figure. *X. citri* cells were grown using 24 well-plates in 2xTY media for seven days at 22 ^{o}C without shaking. *X. citri* biofilm was quantified using a crystal violet-based assay measuring absorbance at 570 nm as described in Materials and Methods.

Data information: Mean absorbance values (vertical lines) +/- SD (horizontal lines) of n=3 technical replicates. Blue dots show the data points from each replicate.

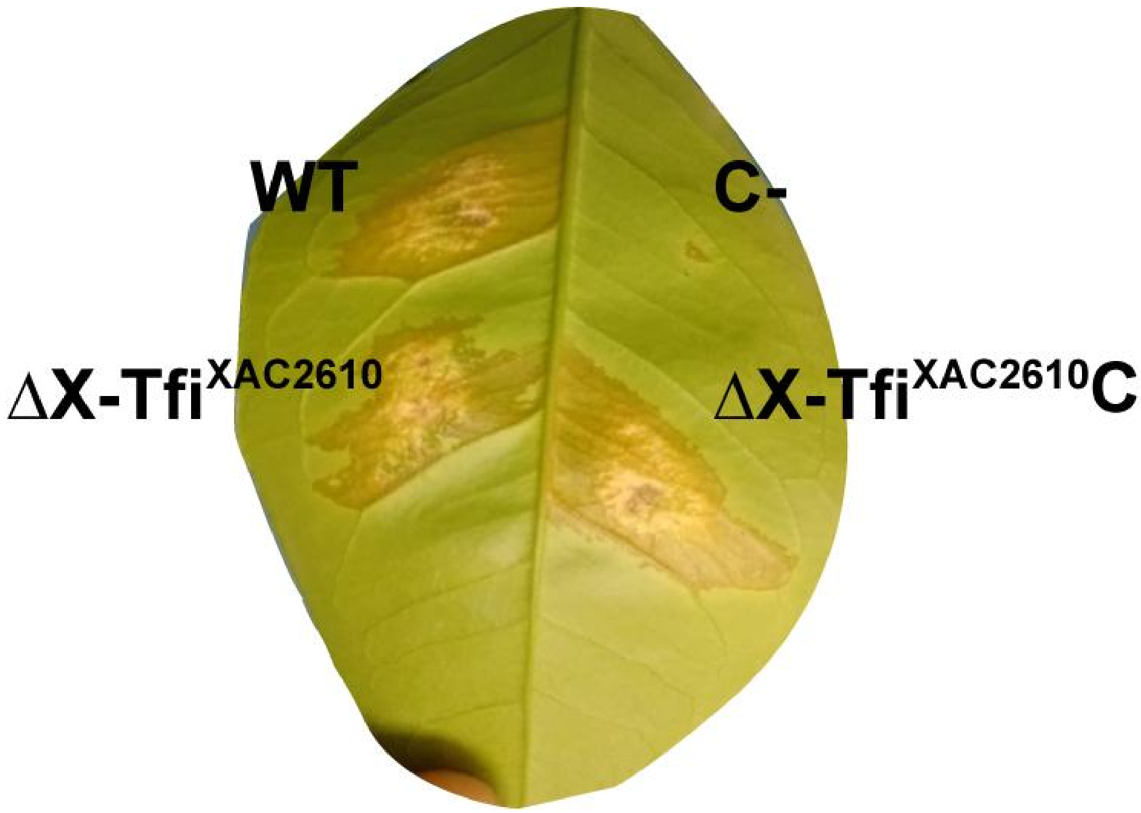

Appendix Figure S6. **Citrus canker development assay on sweet orange leaves after 14 days of infection.**

X. citri strains used: wild-type (WT), ΔX-Tfi^{XAC2610}, ΔX-Tfi^{XAC2610} complemented with pBRAXAC2610 vector (ΔX-Tfi^{XAC2610}C). C-: mock infection with distilled water.

Data information: Shown are typical results of experiments performed on multiple leaves (n=3-5) of four different plants.

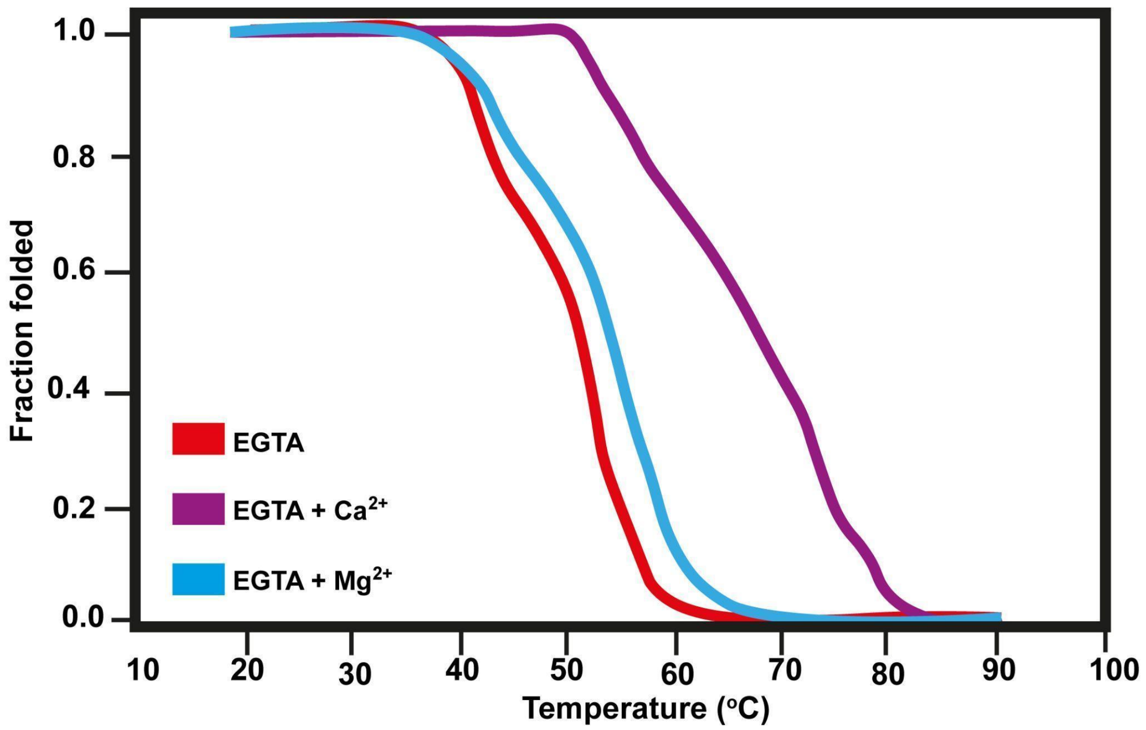

Appendix Figure S7. **Ca^{2+} binding stabilizes X-Tfi^{XAC2610}**.

Thermal denaturation experiments were conducted with 0.5 µM purified recombinant X-Tfi^{XAC2610}(55-267) in 20 mM Tris-HCl (pH 7.5) and 50 mM NaCl) in the presence of EGTA (red), EGTA and Ca^{2+} (purple), EGTA and Mg^{2+} (cyan). EGTA and divalent metal salts ($MgCl_2$ and $CaCl_2$) were added with final concentrations of 0.5 mM and 0.75 mM, respectively. The fraction of folded protein was calculated by monitoring the intrinsic tryptophan fluorescence as described in the Materials and Methods (Pace and Scholtz, 1997).

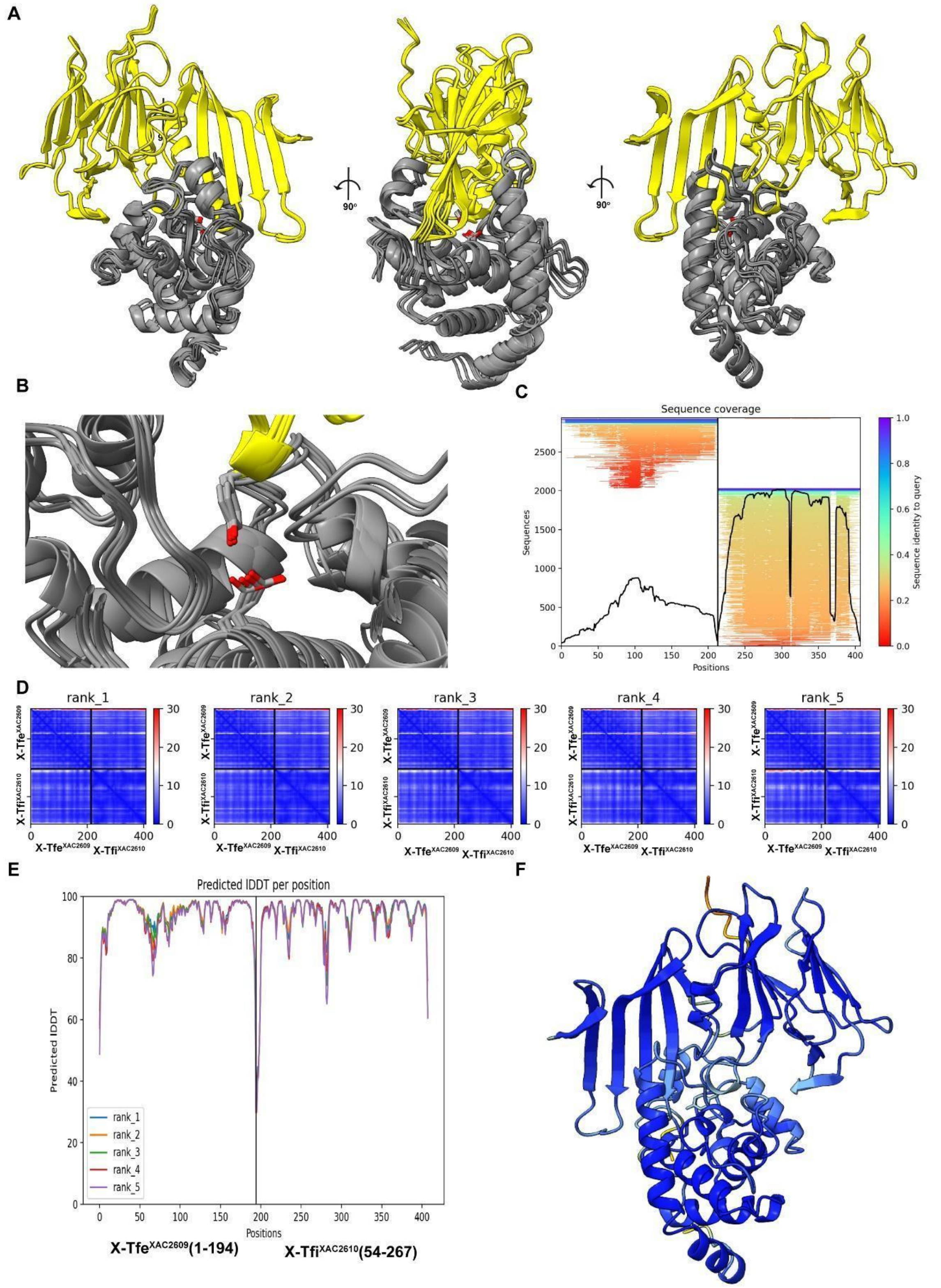

Appendix Figure S8. AlphaFold 2 models of the X-Tfe^{XAC2609}-X-Tfi^{XAC2610} complex.

(A) Superposition of 5 models of the X-Tfe^{XAC2609}(1-194)-X-Tfi^{XAC2610}(54-267) complex predicted by ColabFold-AlphaFold 2 (Mirdita et al. 2022; Varadi et al. 2022; Jumper et al. 2021). X-Tfi^{XAC2610}(54-267) (yellow); X-Tfe^{XAC2609}(1-194) (gray).

(B) Interaction between the conserved Y170 of X-Tfi^{XAC2610} and E48 of X-Tfe^{XAC2609} from the ensemble shown in (**A**). Y170 and E48 are shown as stick models.

(C) Multiple Sequence coverage of the X-Tfe^{XAC2609}(1-194)-X-Tfi^{XAC2610}(54-267) complex.

(D) Predicted Aligned Error file (PAE) of 5 models of the X-Tfe^{XAC2609}(1-194)-X-Tfi^{XAC2610}(54-267) complex.

(E) Predicted local Distance Difference Test (lDDT) score.

(F) Final model of the X-Tfe^{XAC2609}(1-194)-X-Tfi^{XAC2610}(54-267) complex colored according pLDDT values.
