# Supplementary material for: Xanthomonas immunity proteins protect against the cis-toxic effects of their cognate T4SS effectors
Source: EMBO Rep. 2024 Feb 8;25(3):27. doi: 10.1038/s44319-024-00060-6 (PMC10933484; doi:10.1038/s44319-024-00060-6)
Supplement: Supplementary file 10 — Source Data Fig. 2 [file 44319_2024_60_MOESM10_ESM.zip › Fig 2/2A numerical data/2A readme.docx]

Raw data for CPRG-based bacterial competition curves shown in Fig 2A

column A: time minutes

other columns: Absorbance at 572 nm

The data includes mean and SD values from replicates r1 r2 r3.

Mean columns are named according to the Figure legend.

type (WT), ΔvirB7 (ΔVirB7), ∆X-TfeXAC2609∆X-TfiXAC2610
