# Supplementary material for: Xanthomonas immunity proteins protect against the cis-toxic effects of their cognate T4SS effectors
Source: EMBO Rep. 2024 Feb 8;25(3):27. doi: 10.1038/s44319-024-00060-6 (PMC10933484; doi:10.1038/s44319-024-00060-6)
Supplement: Supplementary file 10 — Source Data Fig. 2 [file 44319_2024_60_MOESM10_ESM.zip › Fig 2/2B numerical data/2B readme.docx]

CFU ratio of X.citri (gentR)/X.citri (kanR) co-cultures after 40 hours

Numerical data includes five replicates (R1, R2, R3, R4, R5), cell viability in kanamycin and gentamicin, and the ratio of viability (gent/kan) mean and SD.
