# Supplementary material for: Xanthomonas immunity proteins protect against the cis-toxic effects of their cognate T4SS effectors
Source: EMBO Rep. 2024 Feb 8;25(3):27. doi: 10.1038/s44319-024-00060-6 (PMC10933484; doi:10.1038/s44319-024-00060-6)
Supplement: Supplementary file 10 — Source Data Fig. 2 [file 44319_2024_60_MOESM10_ESM.zip › Fig 2/2C numerical data/readme Fig 2C.docx]

Cell viability data for specific X. citri strains measured after 48 h, 72 h, 96 h

Numerical data includes four replicates (R1,R2,R3,R4), mean and SD.
