# Supplementary figures and images for: Xanthomonas immunity proteins protect against the cis-toxic effects of their cognate T4SS effectors (part 1 of 2)
Source: EMBO Rep. 2024 Feb 8;25(3):27. doi: 10.1038/s44319-024-00060-6 (PMC10933484; doi:10.1038/s44319-024-00060-6)

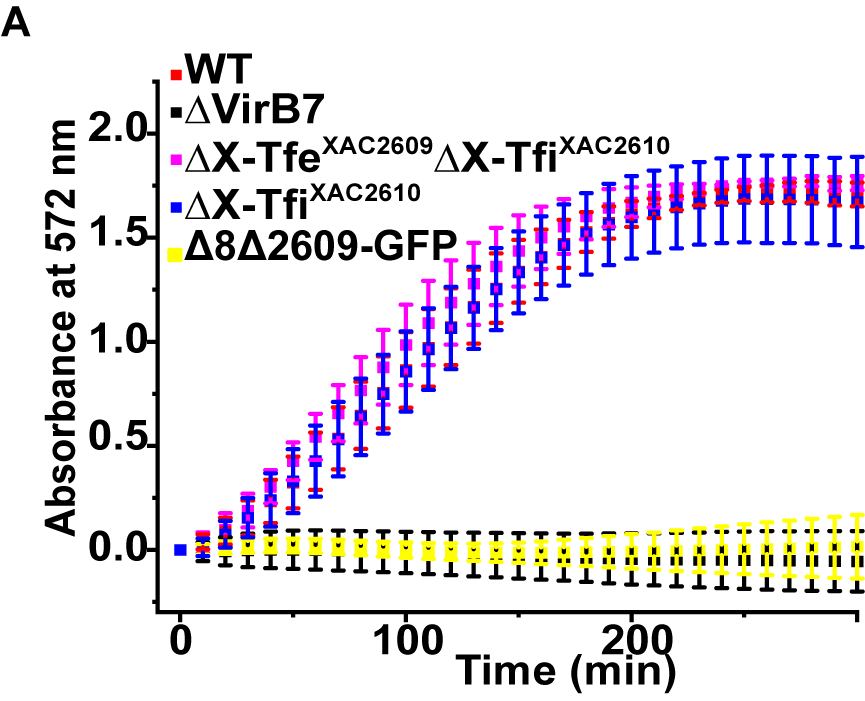

Supplement: Supplementary file 10 — Source Data Fig. 2 [file 44319_2024_60_MOESM10_ESM.zip › Fig 2/2A numerical data/2A.tif]

A

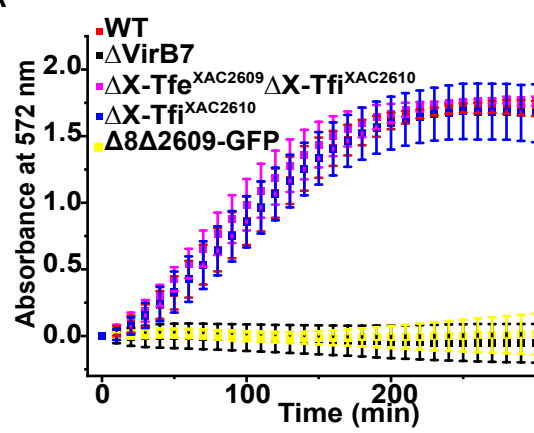

Supplement: Supplementary file 10 — Source Data Fig. 2 [file 44319_2024_60_MOESM10_ESM.zip › Fig 2/2A numerical data/2A.pdf]

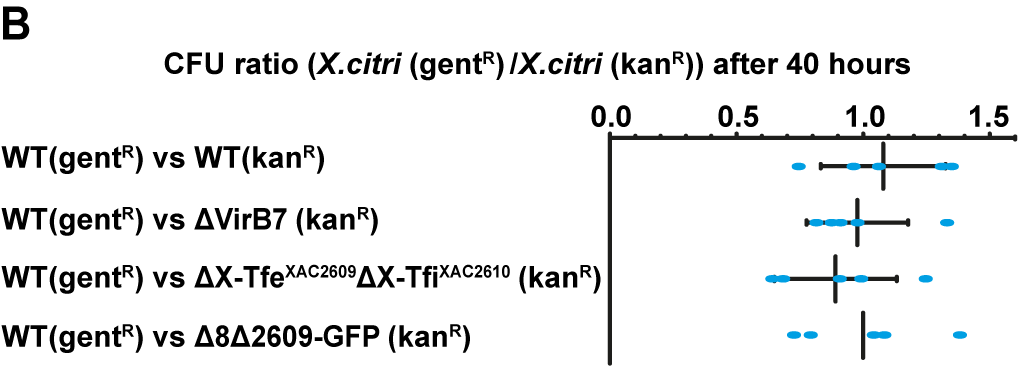

Supplement: Supplementary file 10 — Source Data Fig. 2 [file 44319_2024_60_MOESM10_ESM.zip › Fig 2/2B numerical data/2B.tif]

**B**

CFU ratio (*X.citri* (gent<sup>R</sup>) / *X.citri* (kan<sup>R</sup>)) after 40 hours

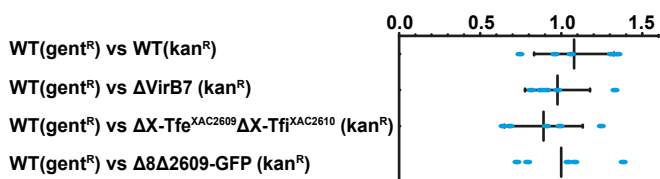

Supplement: Supplementary file 10 — Source Data Fig. 2 [file 44319_2024_60_MOESM10_ESM.zip › Fig 2/2B numerical data/2B.pdf]

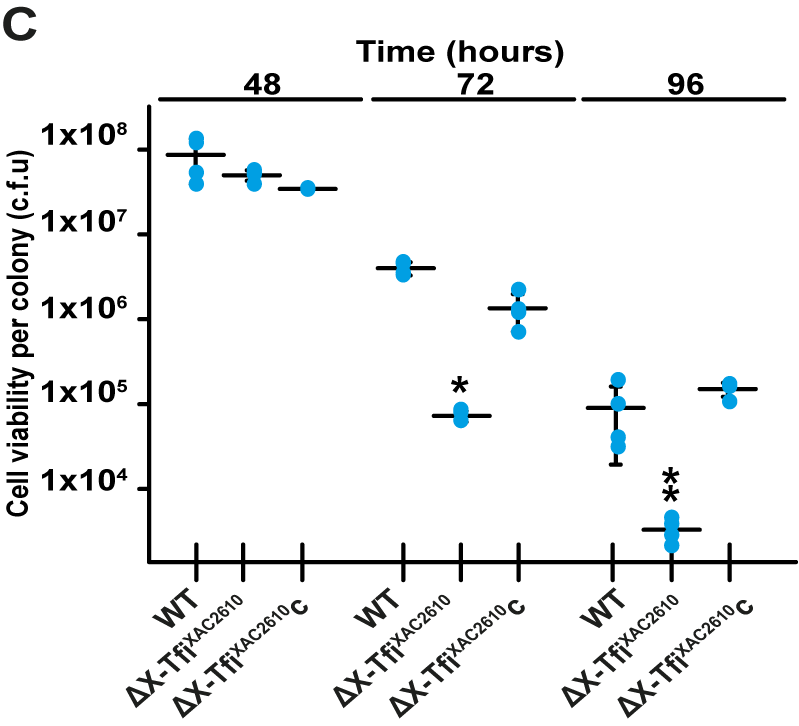

Supplement: Supplementary file 10 — Source Data Fig. 2 [file 44319_2024_60_MOESM10_ESM.zip › Fig 2/2C numerical data/2C.tif]

**C**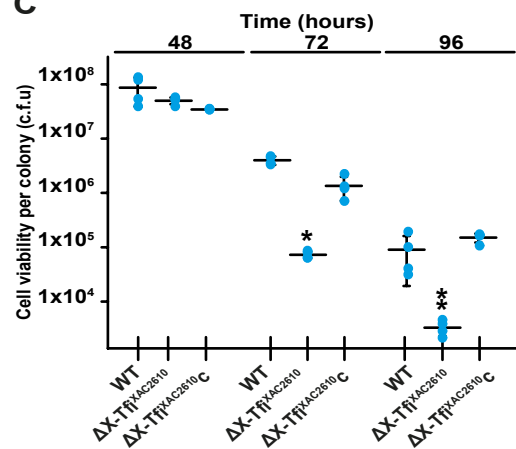

Supplement: Supplementary file 10 — Source Data Fig. 2 [file 44319_2024_60_MOESM10_ESM.zip › Fig 2/2C numerical data/2C.pdf]

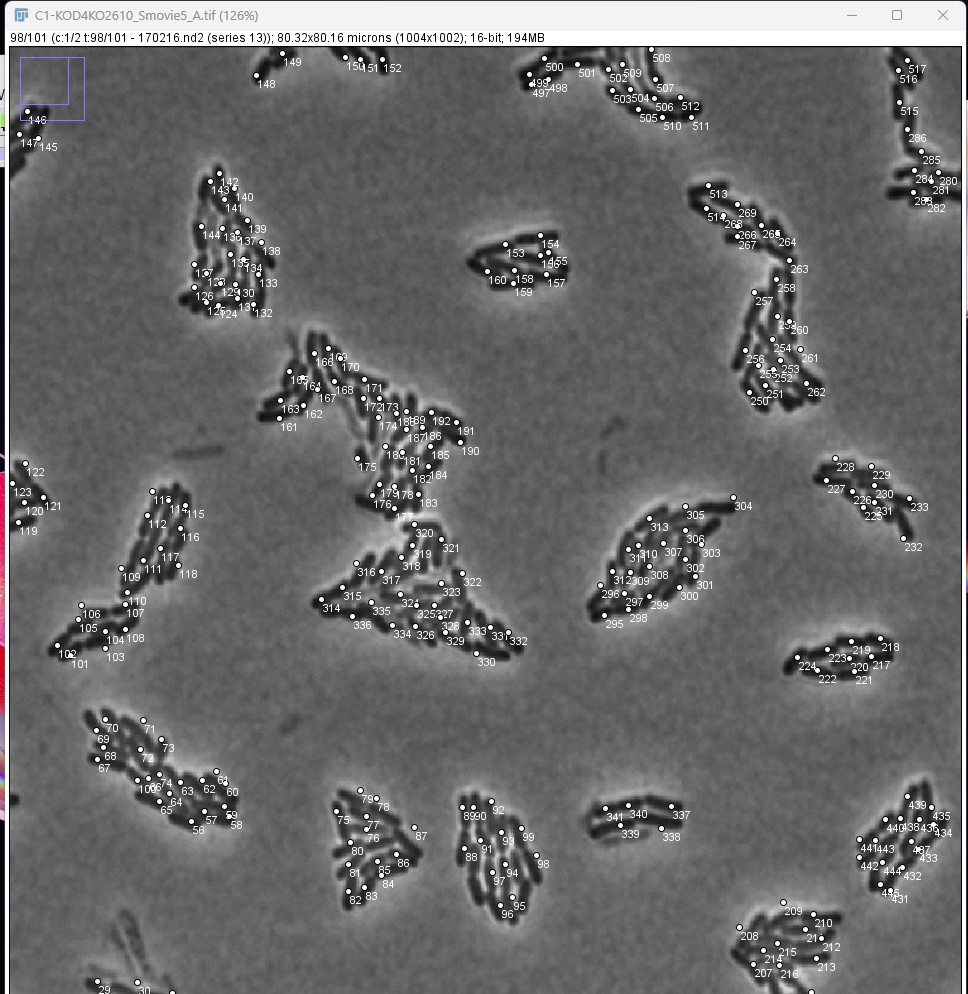

Supplement: Supplementary file 11 — Source Data Fig. 3 [file 44319_2024_60_MOESM11_ESM.zip › Fig3 no micrographs/Movie raw data analysis/ KO2610KOD4_video1_tot530.jpg]

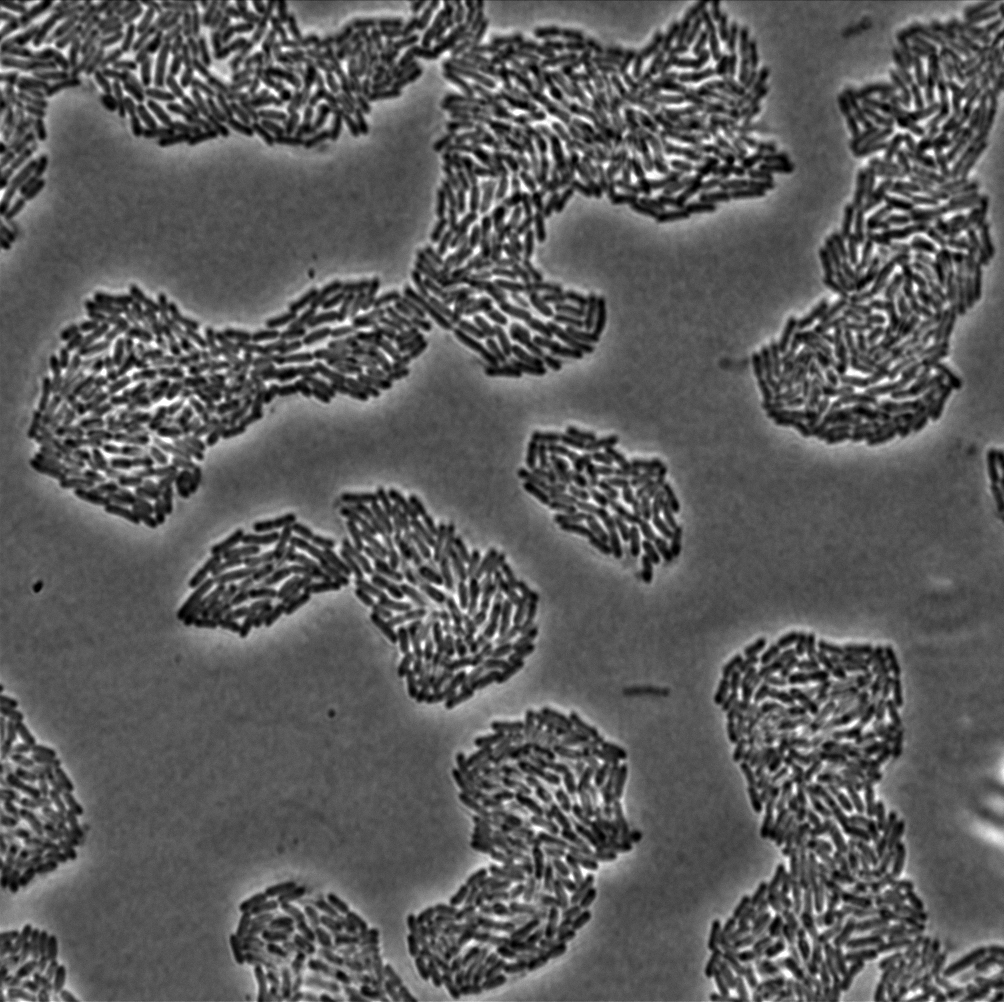

Supplement: Supplementary file 11 — Source Data Fig. 3 [file 44319_2024_60_MOESM11_ESM.zip › Fig3 no micrographs/Movie raw data analysis/KO2610_video2_total_1189.tif]

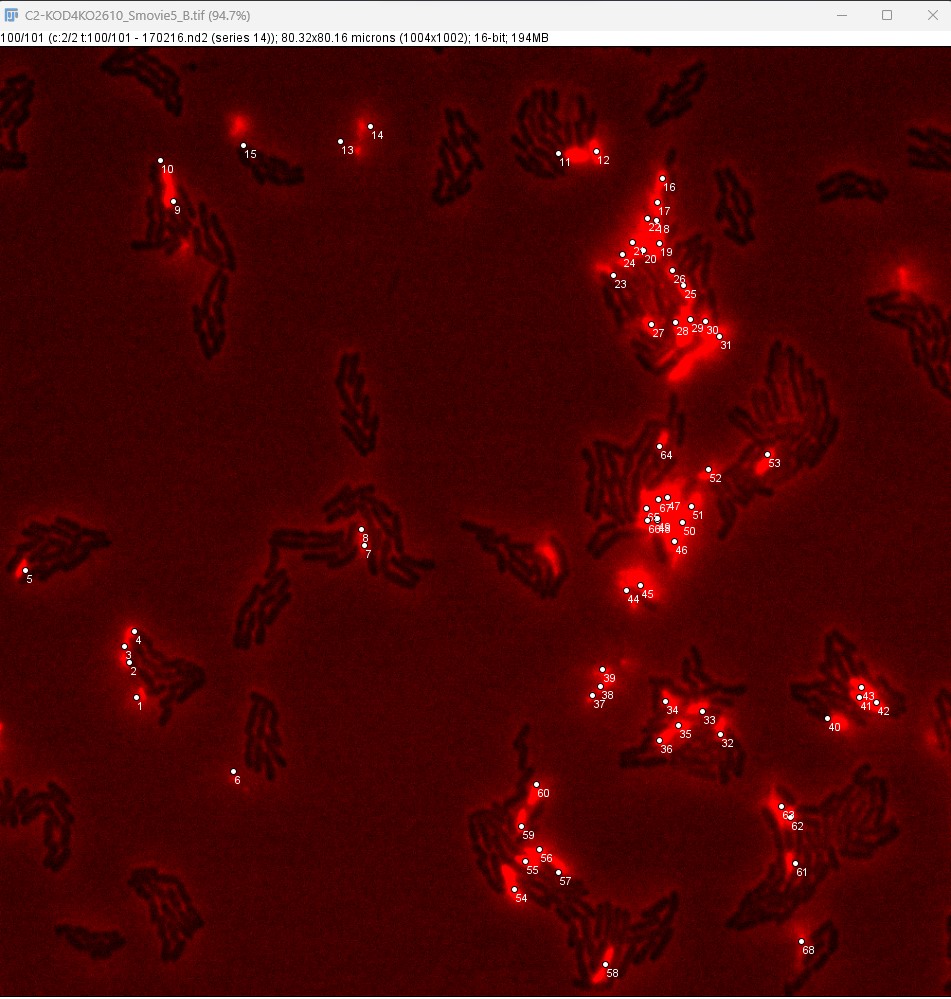

Supplement: Supplementary file 11 — Source Data Fig. 3 [file 44319_2024_60_MOESM11_ESM.zip › Fig3 no micrographs/Movie raw data analysis/KO26010KOD4_video2_IP68.jpg]

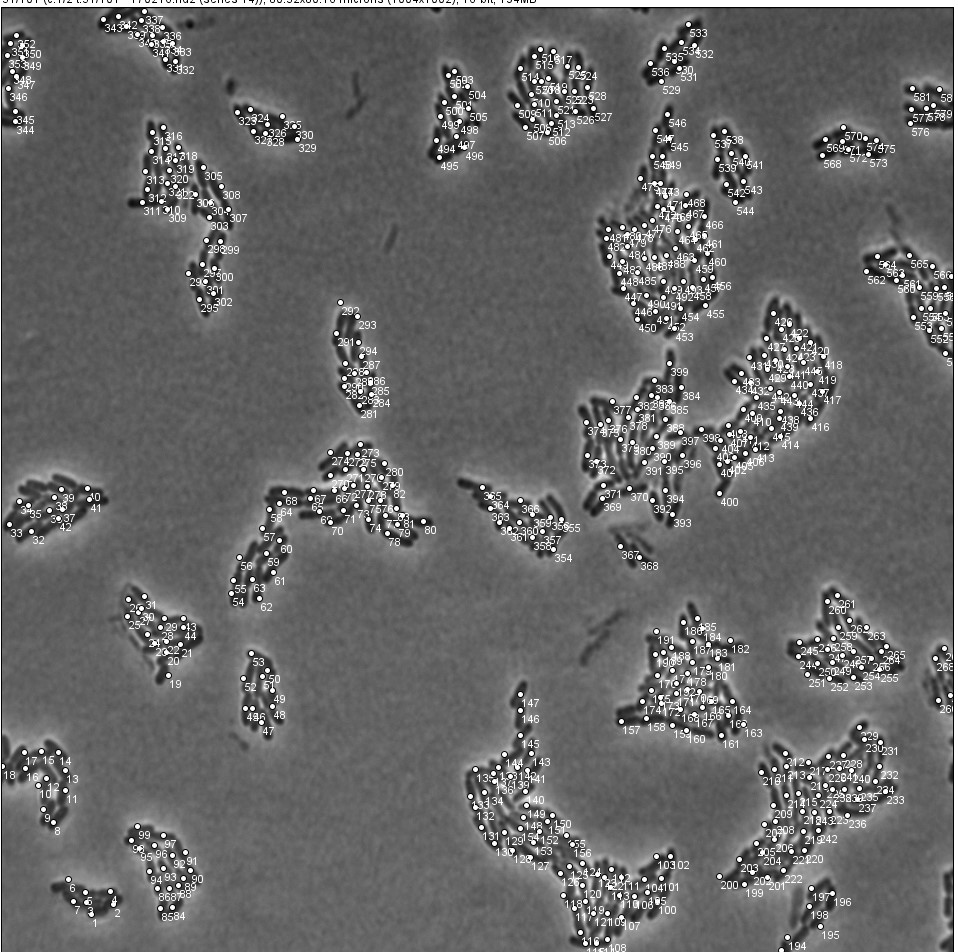

Supplement: Supplementary file 11 — Source Data Fig. 3 [file 44319_2024_60_MOESM11_ESM.zip › Fig3 no micrographs/Movie raw data analysis/KO2610KOD4_video2_tot588.jpg]

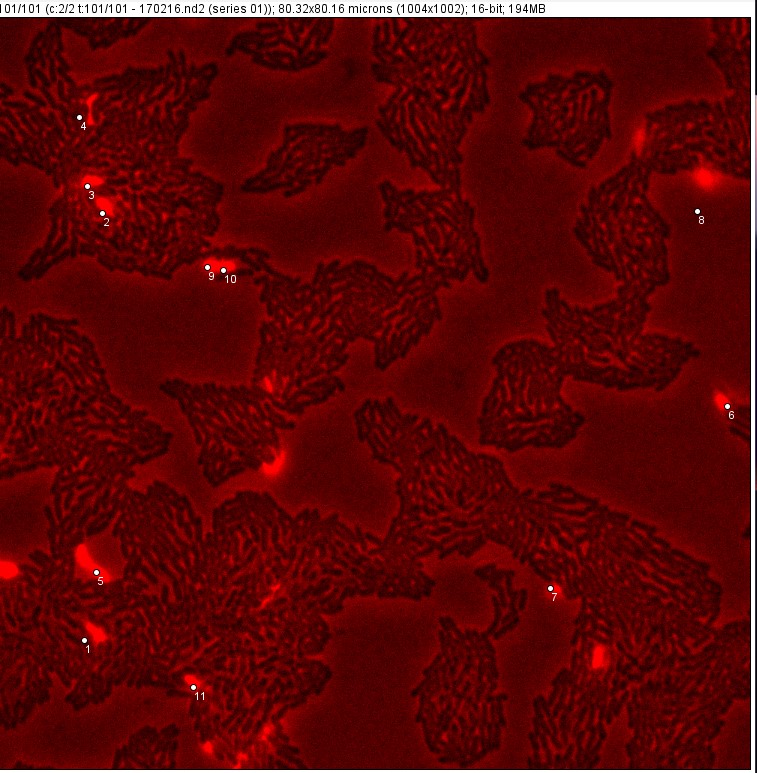

Supplement: Supplementary file 11 — Source Data Fig. 3 [file 44319_2024_60_MOESM11_ESM.zip › Fig3 no micrographs/Movie raw data analysis/WT_movieS1A_PIP_11.jpg]

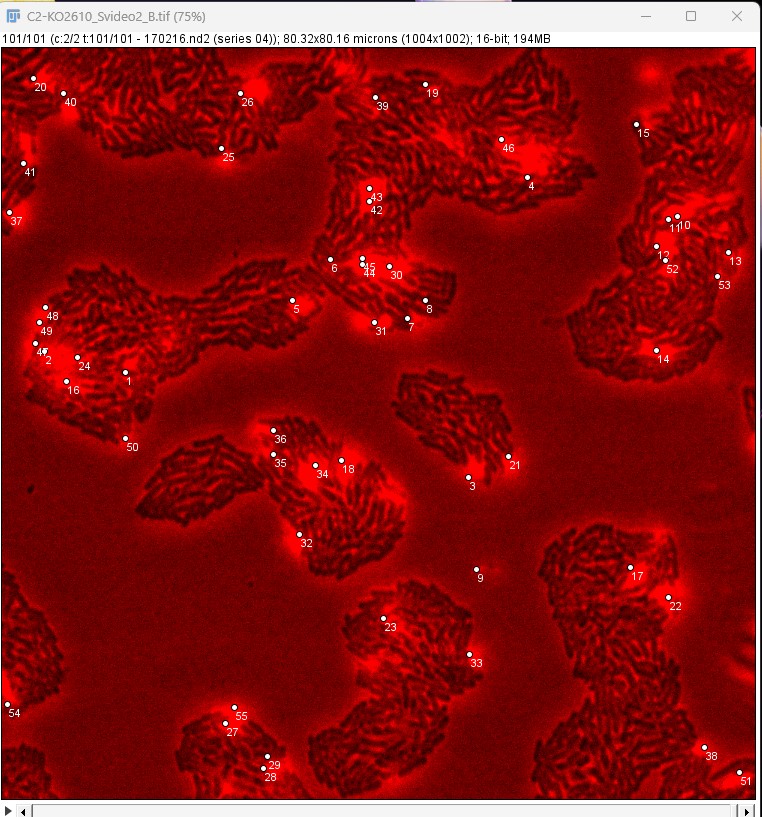

Supplement: Supplementary file 11 — Source Data Fig. 3 [file 44319_2024_60_MOESM11_ESM.zip › Fig3 no micrographs/Movie raw data analysis/KO2610_video2_IP.jpg]

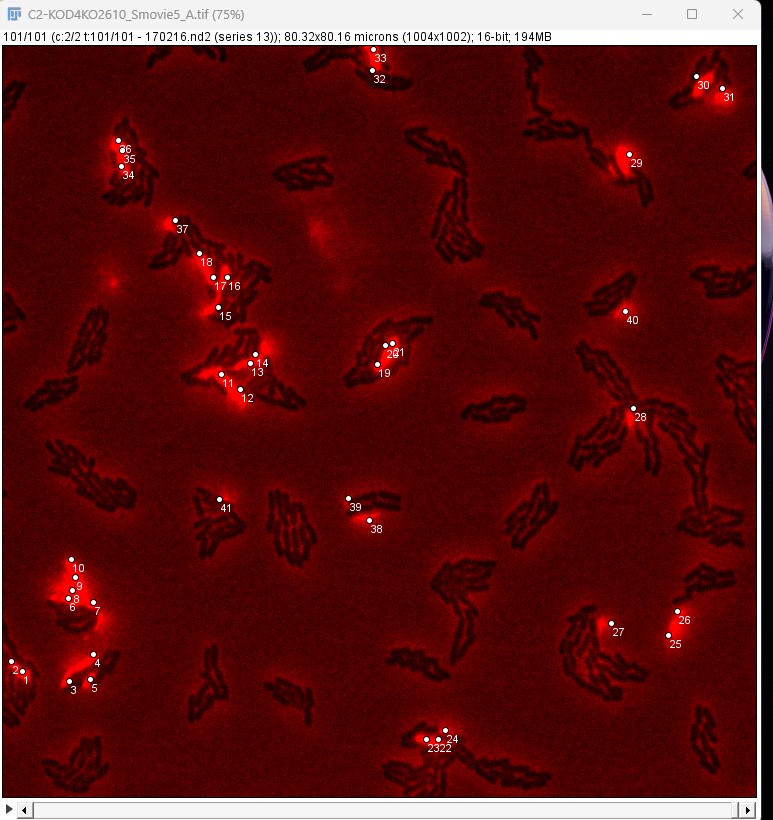

Supplement: Supplementary file 11 — Source Data Fig. 3 [file 44319_2024_60_MOESM11_ESM.zip › Fig3 no micrographs/Movie raw data analysis/KO26010KOD4_video1_IP41.jpg]

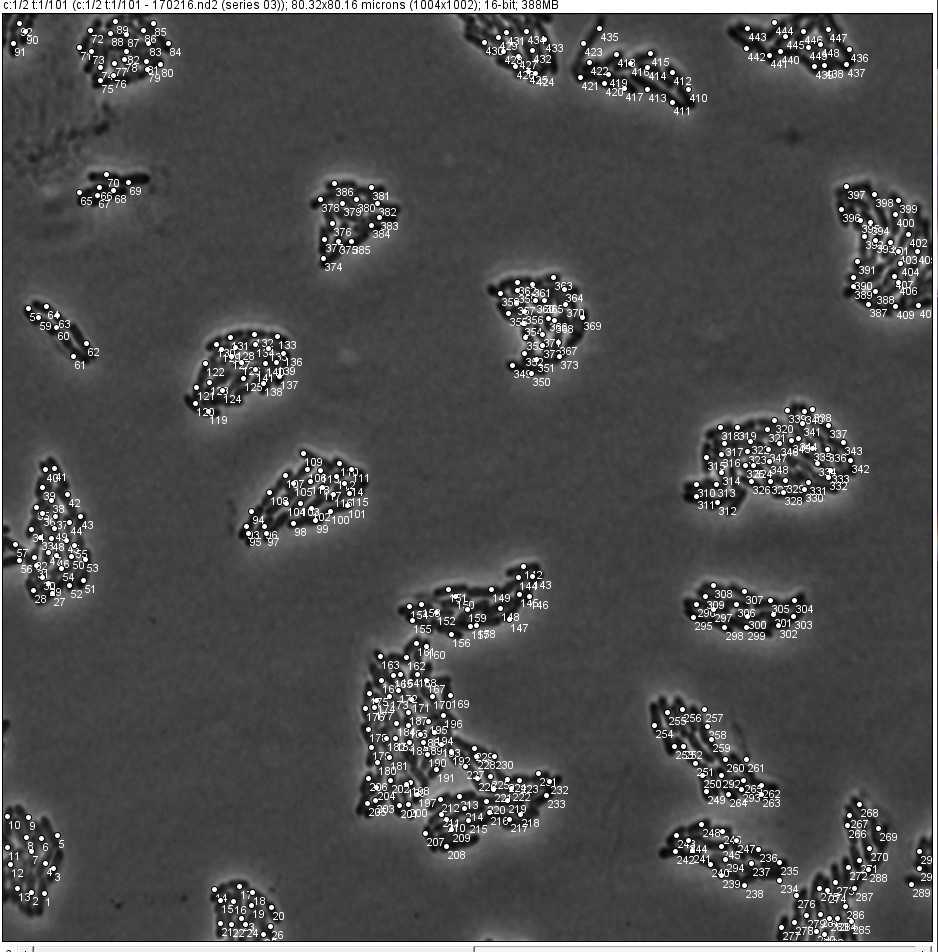

Supplement: Supplementary file 11 — Source Data Fig. 3 [file 44319_2024_60_MOESM11_ESM.zip › Fig3 no micrographs/Movie raw data analysis/KO2610_movieS2A_total_549_inicio.jpg]

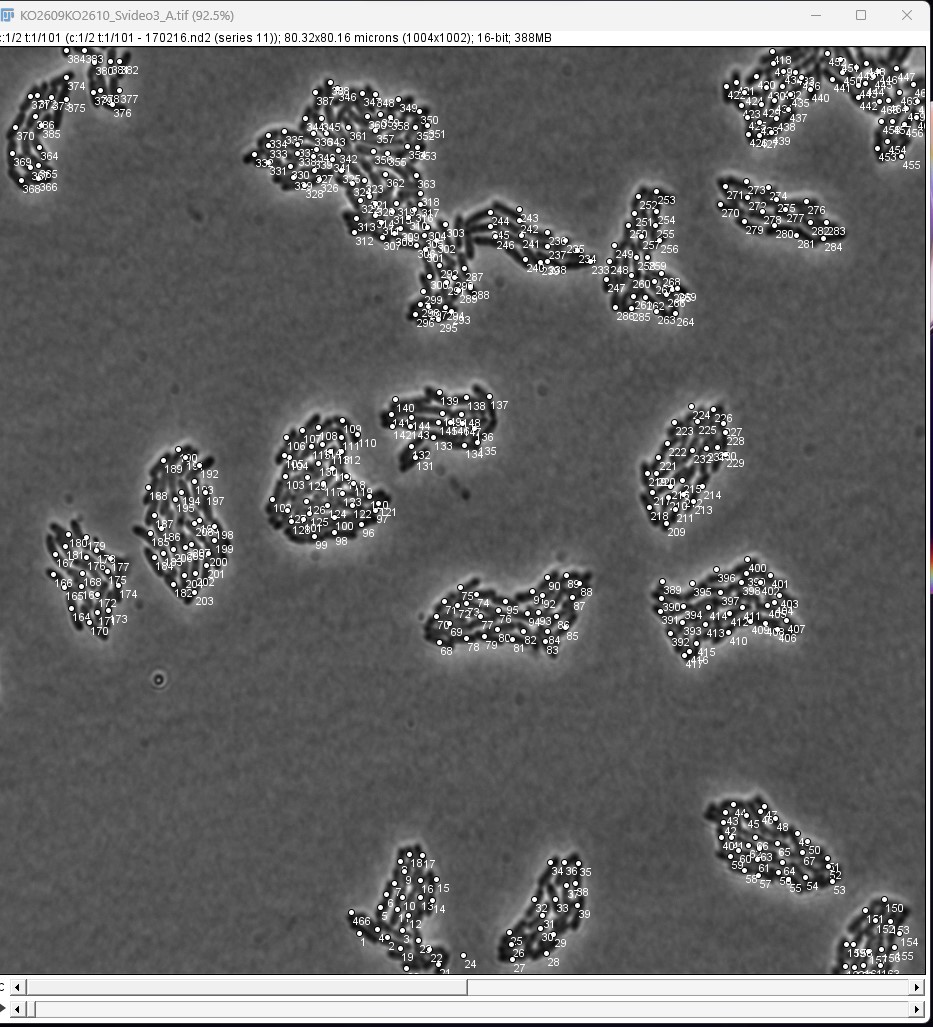

Supplement: Supplementary file 11 — Source Data Fig. 3 [file 44319_2024_60_MOESM11_ESM.zip › Fig3 no micrographs/Movie raw data analysis/KO260KO2609_smovie3A_total_466_initio.jpg]

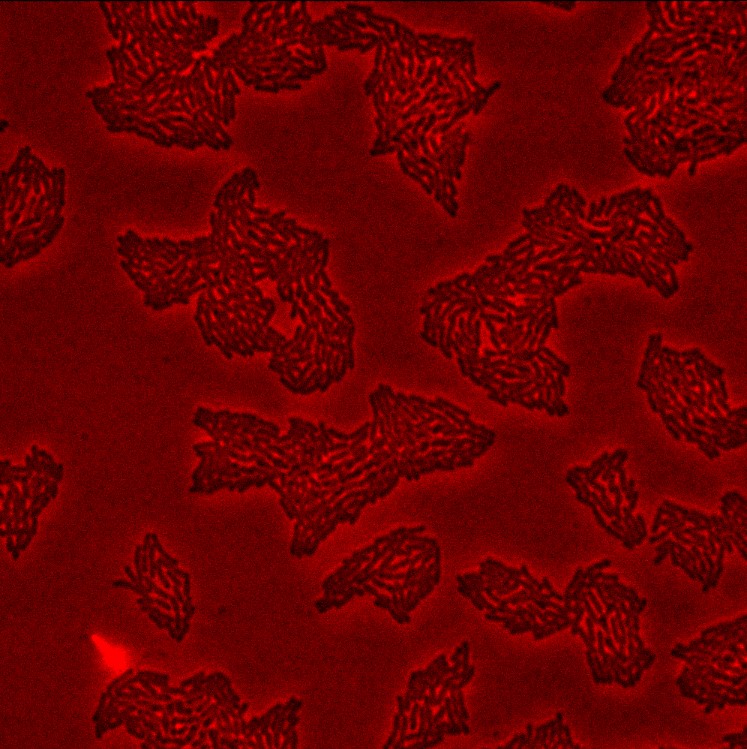

Supplement: Supplementary file 11 — Source Data Fig. 3 [file 44319_2024_60_MOESM11_ESM.zip › Fig3 no micrographs/Movie raw data analysis/ KO260KO2609_movieS3B_PlP_0.jpg]

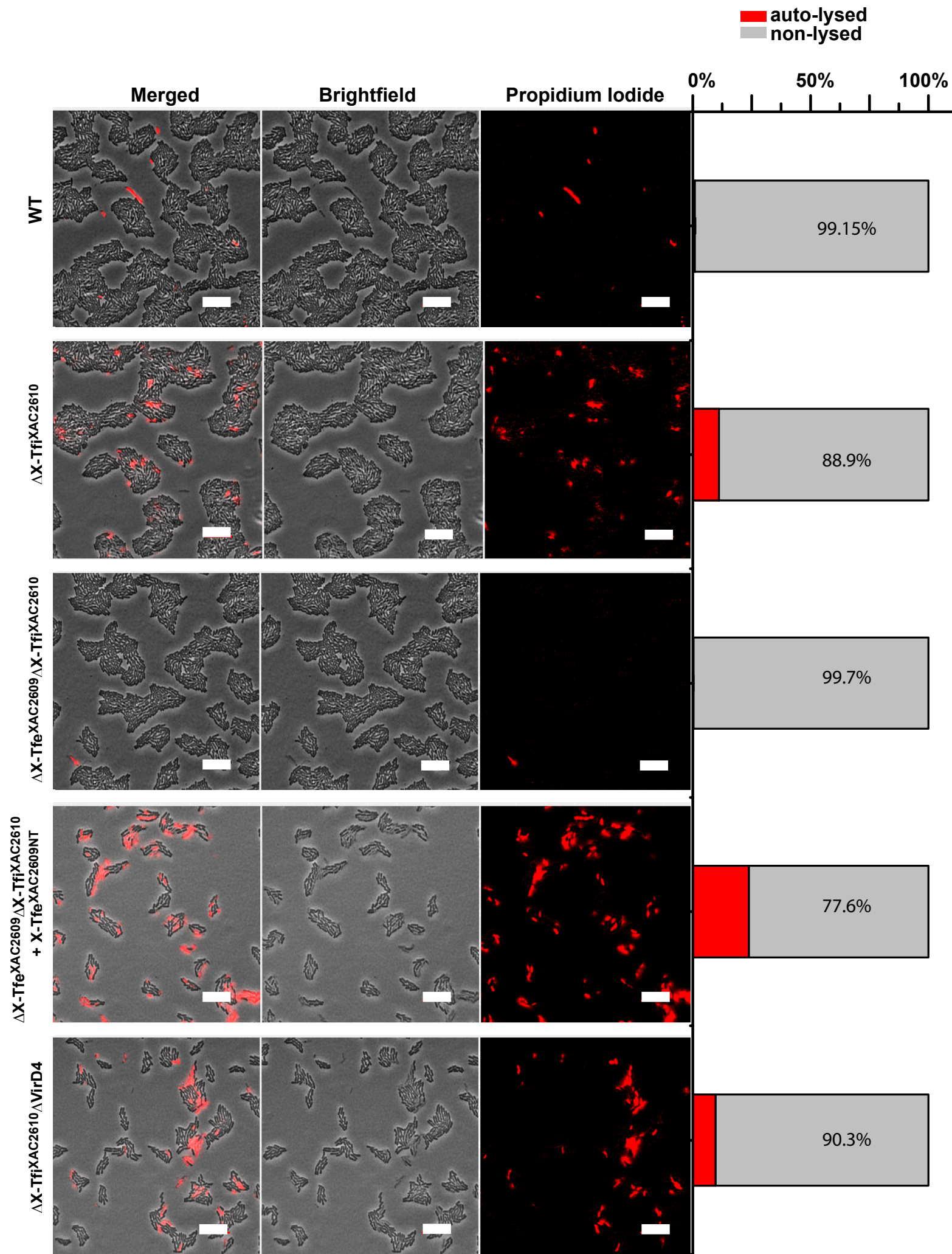

Supplement: Supplementary file 11 — Source Data Fig. 3 [file 44319_2024_60_MOESM11_ESM.zip › Fig3 no micrographs/FIg3.pdf]

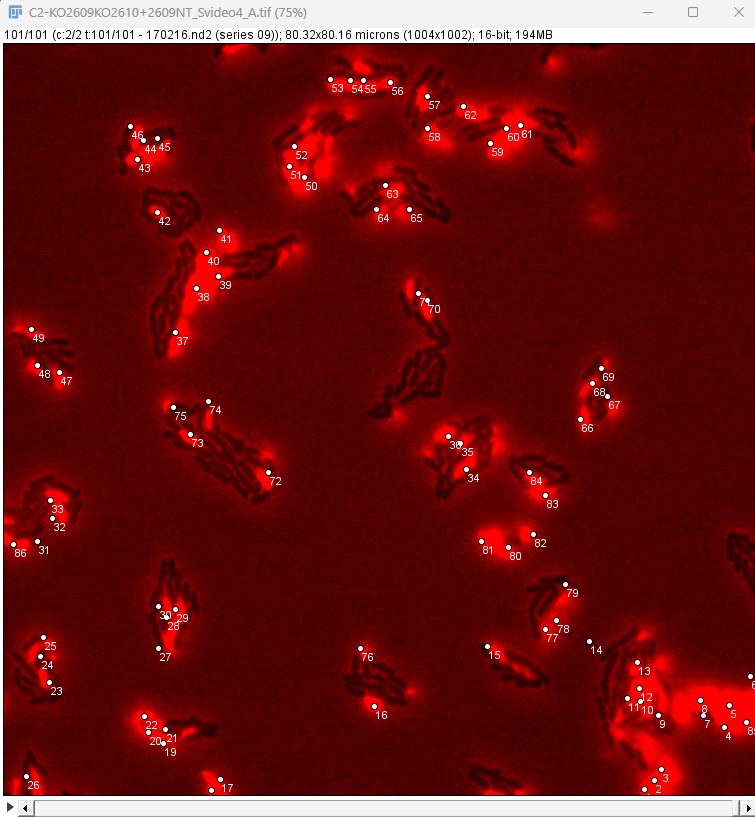

Supplement: Supplementary file 11 — Source Data Fig. 3 [file 44319_2024_60_MOESM11_ESM.zip › Fig3 no micrographs/Movie raw data analysis/KO260KO2609+2609NT_smovie4A_PIP_85.jpg]

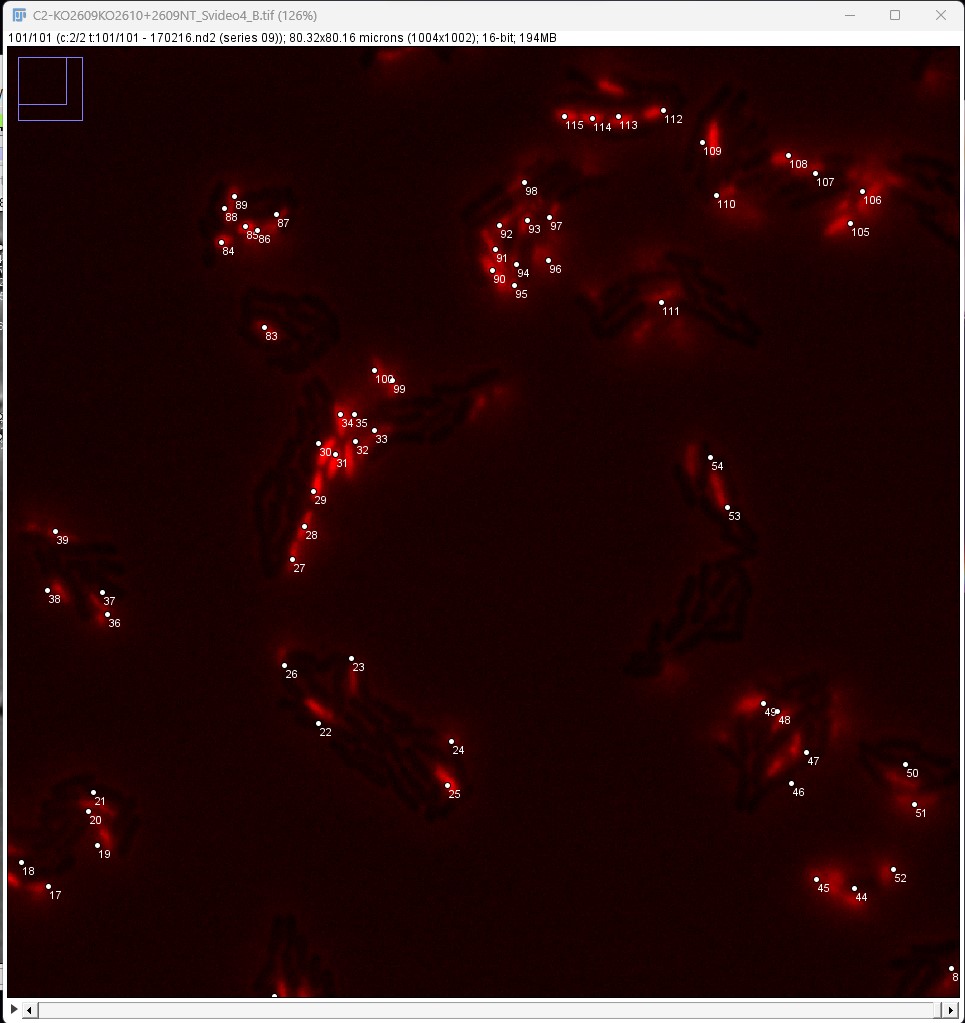

Supplement: Supplementary file 11 — Source Data Fig. 3 [file 44319_2024_60_MOESM11_ESM.zip › Fig3 no micrographs/Movie raw data analysis/KO2610_movieS2A_PIP_72.jpg]

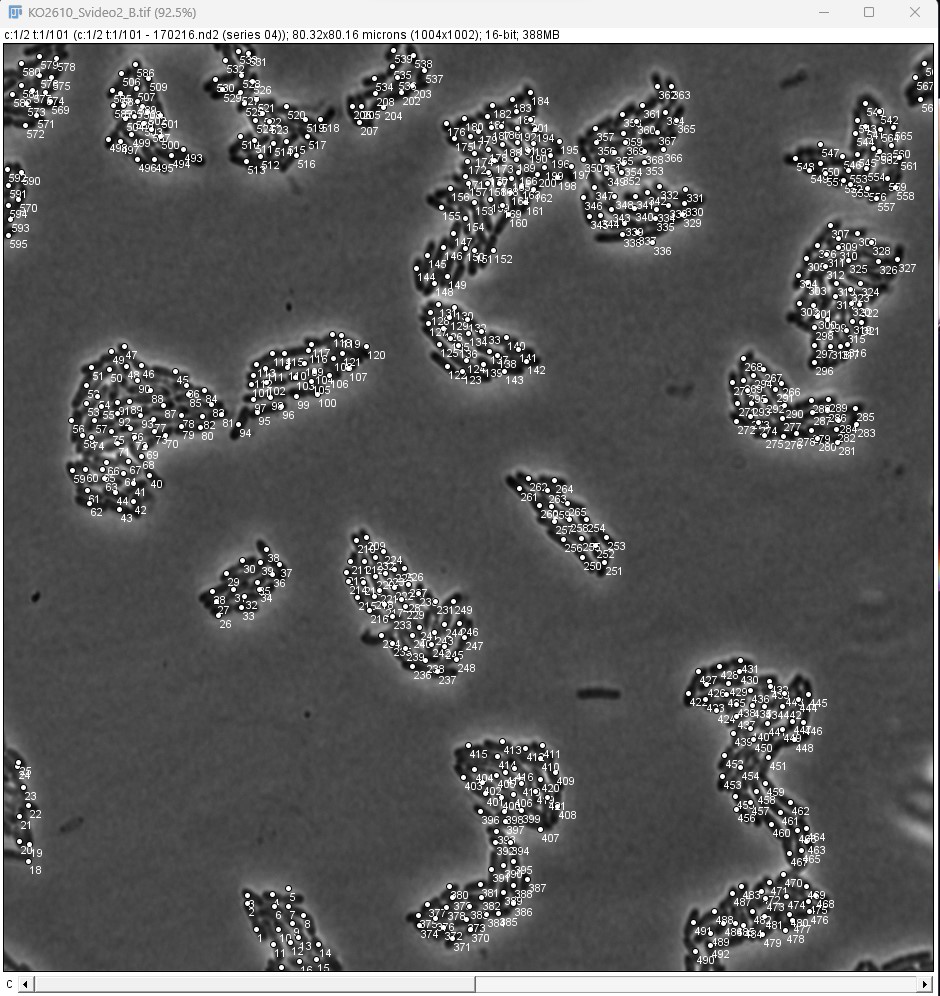

Supplement: Supplementary file 11 — Source Data Fig. 3 [file 44319_2024_60_MOESM11_ESM.zip › Fig3 no micrographs/Movie raw data analysis/KO2610_movieS2B_total_595_inicio.jpg]

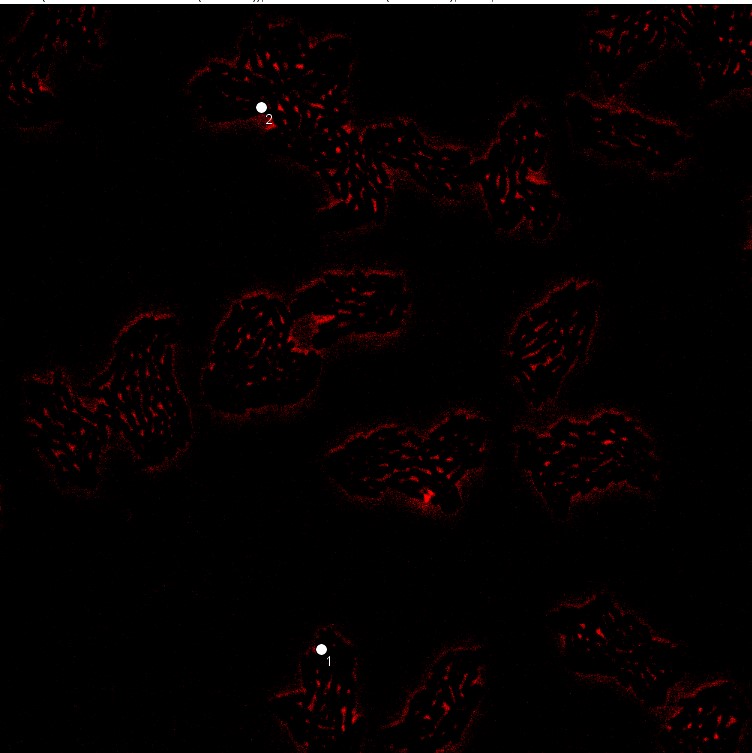

Supplement: Supplementary file 11 — Source Data Fig. 3 [file 44319_2024_60_MOESM11_ESM.zip › Fig3 no micrographs/Movie raw data analysis/KO260KO2609_smovie3A_PlP_2.jpg]

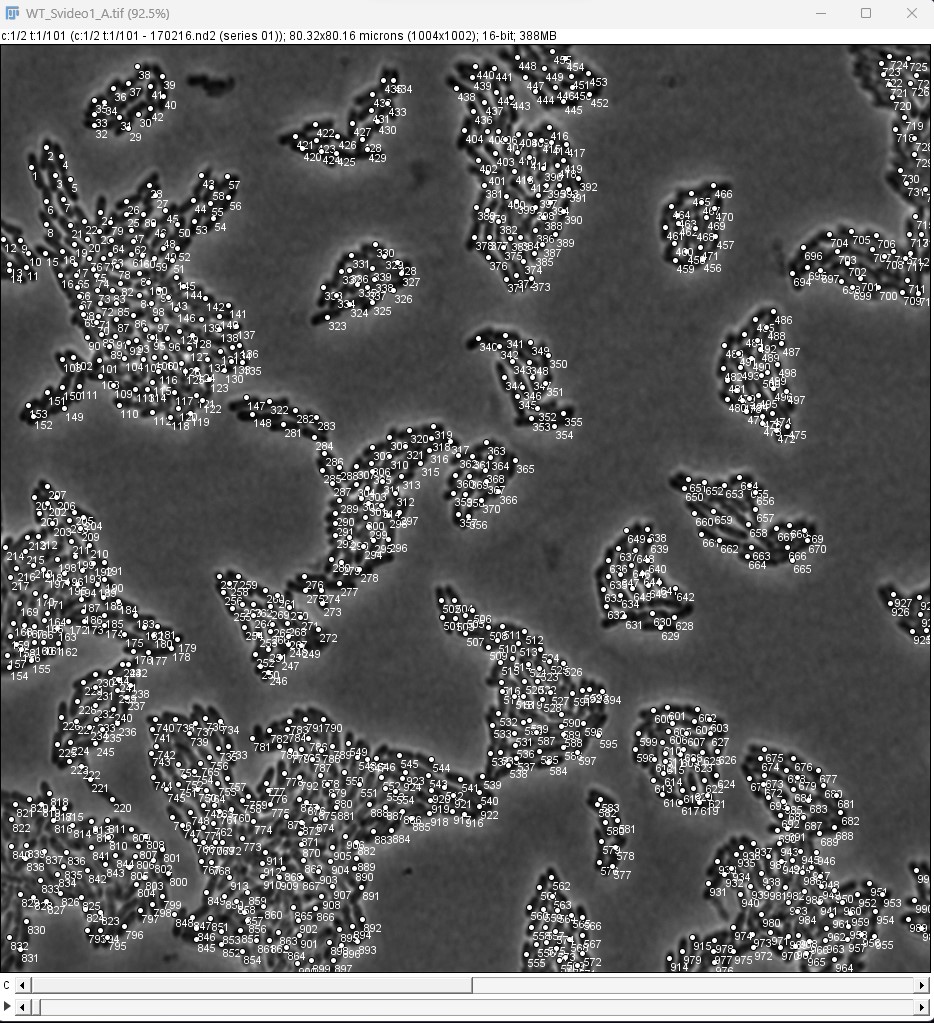

Supplement: Supplementary file 11 — Source Data Fig. 3 [file 44319_2024_60_MOESM11_ESM.zip › Fig3 no micrographs/Movie raw data analysis/WT_movieS1A_tot_991_T=0.jpg]

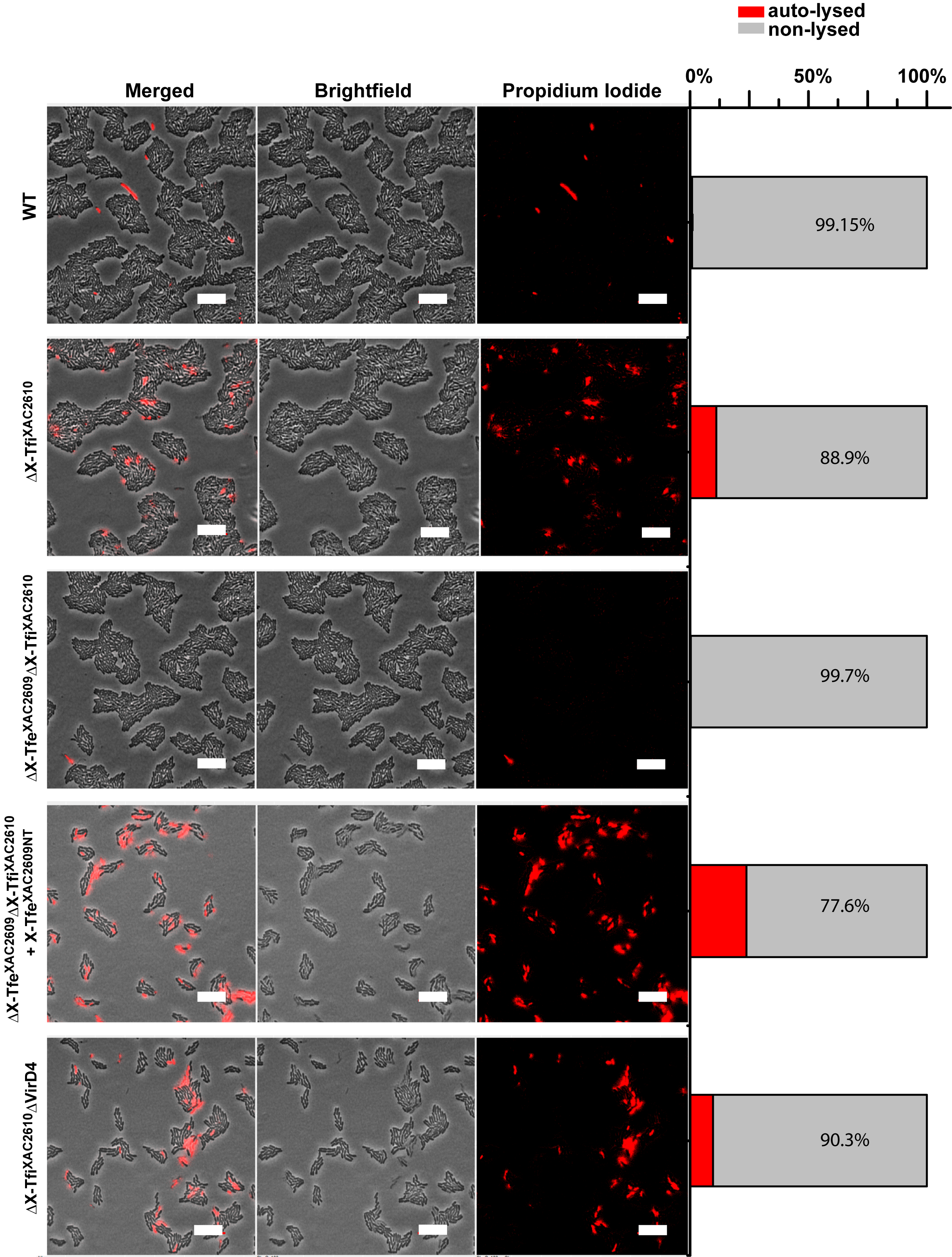

Supplement: Supplementary file 11 — Source Data Fig. 3 [file 44319_2024_60_MOESM11_ESM.zip › Fig3 no micrographs/Fig3.tif]

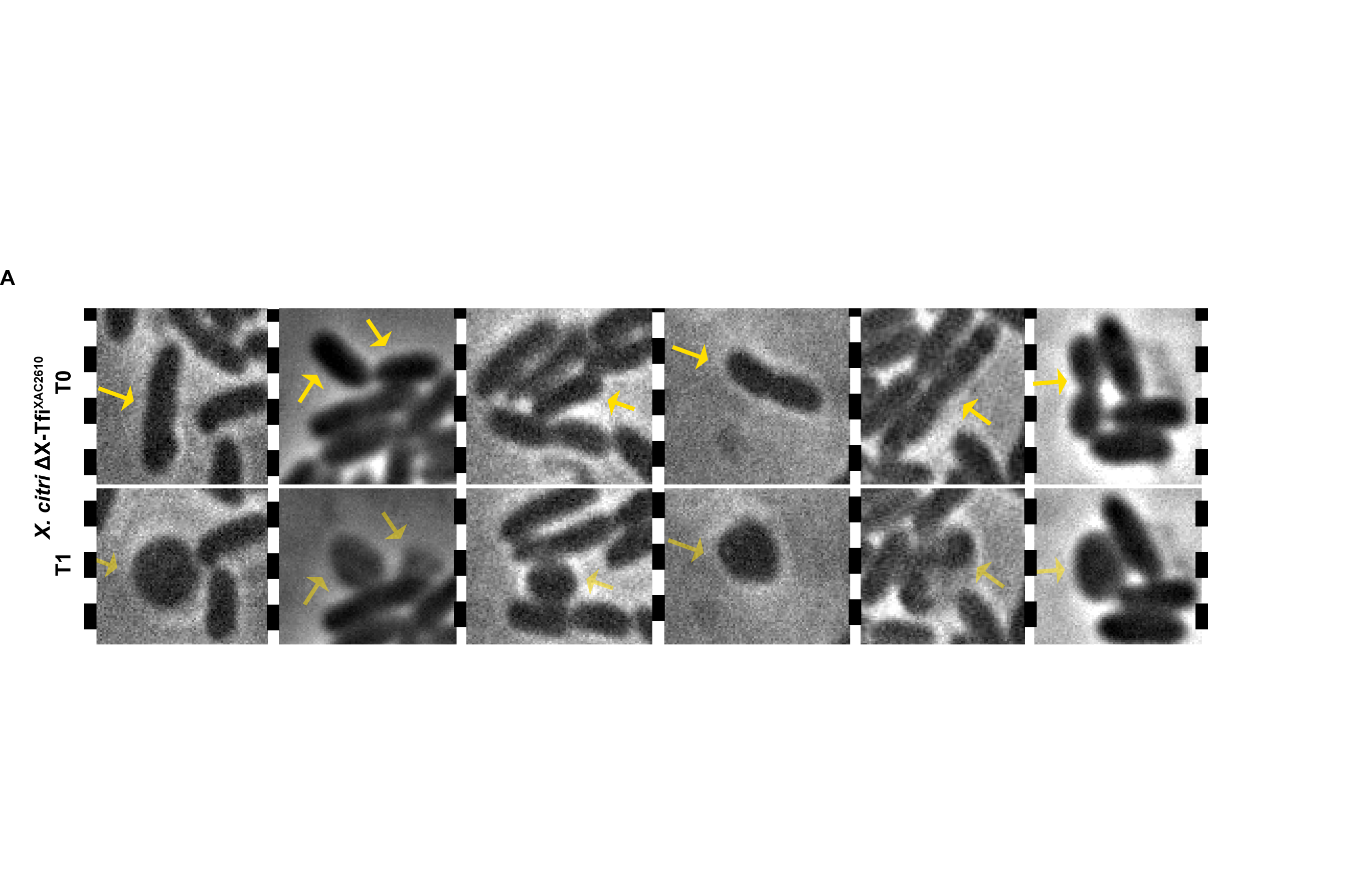

Supplement: Supplementary file 12 — Source Data Fig. 4 [file 44319_2024_60_MOESM12_ESM.zip › Fig 4/4A/4A.tif]

A

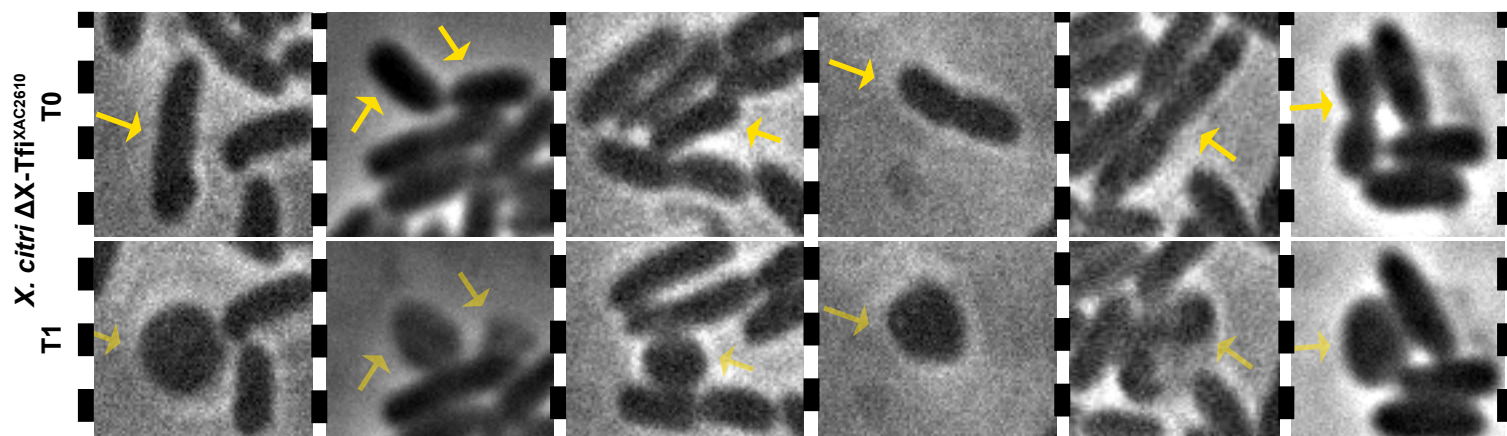

Supplement: Supplementary file 12 — Source Data Fig. 4 [file 44319_2024_60_MOESM12_ESM.zip › Fig 4/4A/4A.pdf]

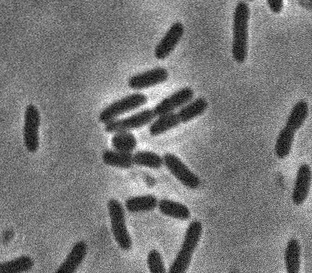

Supplement: Supplementary file 12 — Source Data Fig. 4 [file 44319_2024_60_MOESM12_ESM.zip › Fig 4/4A/micrographs/T=1 Example_C_.tif]

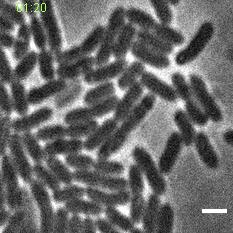

Supplement: Supplementary file 12 — Source Data Fig. 4 [file 44319_2024_60_MOESM12_ESM.zip › Fig 4/4A/micrographs/T=0 Example_F_.tif]

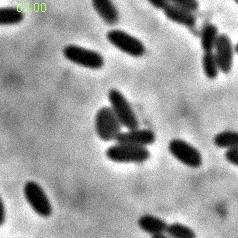

Supplement: Supplementary file 12 — Source Data Fig. 4 [file 44319_2024_60_MOESM12_ESM.zip › Fig 4/4A/micrographs/T=1 Example_E_.tif]

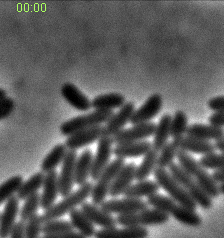

Supplement: Supplementary file 12 — Source Data Fig. 4 [file 44319_2024_60_MOESM12_ESM.zip › Fig 4/4A/micrographs/T=0 Example_B_.tif]

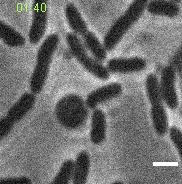

Supplement: Supplementary file 12 — Source Data Fig. 4 [file 44319_2024_60_MOESM12_ESM.zip › Fig 4/4A/micrographs/T=1 Example_A_.tif]

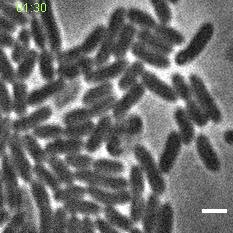

Supplement: Supplementary file 12 — Source Data Fig. 4 [file 44319_2024_60_MOESM12_ESM.zip › Fig 4/4A/micrographs/T=1 Example_F_.tif]

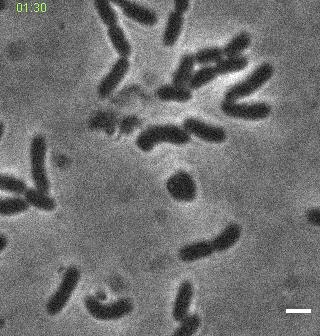

Supplement: Supplementary file 12 — Source Data Fig. 4 [file 44319_2024_60_MOESM12_ESM.zip › Fig 4/4A/micrographs/T=1 Example_D_.tif]

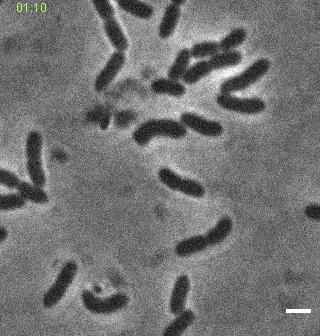

Supplement: Supplementary file 12 — Source Data Fig. 4 [file 44319_2024_60_MOESM12_ESM.zip › Fig 4/4A/micrographs/T=0 Example_D_.tif]

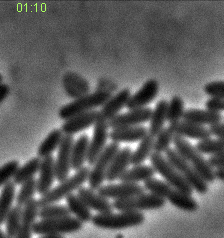

Supplement: Supplementary file 12 — Source Data Fig. 4 [file 44319_2024_60_MOESM12_ESM.zip › Fig 4/4A/micrographs/T=1 Example_B_.tif]

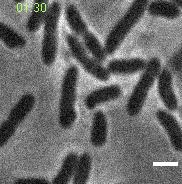

Supplement: Supplementary file 12 — Source Data Fig. 4 [file 44319_2024_60_MOESM12_ESM.zip › Fig 4/4A/micrographs/T=0 Example_A_.tif]

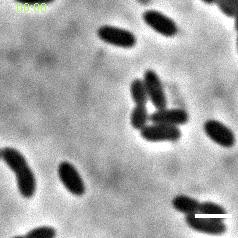

Supplement: Supplementary file 12 — Source Data Fig. 4 [file 44319_2024_60_MOESM12_ESM.zip › Fig 4/4A/micrographs/T=0 Example_E_.tif]

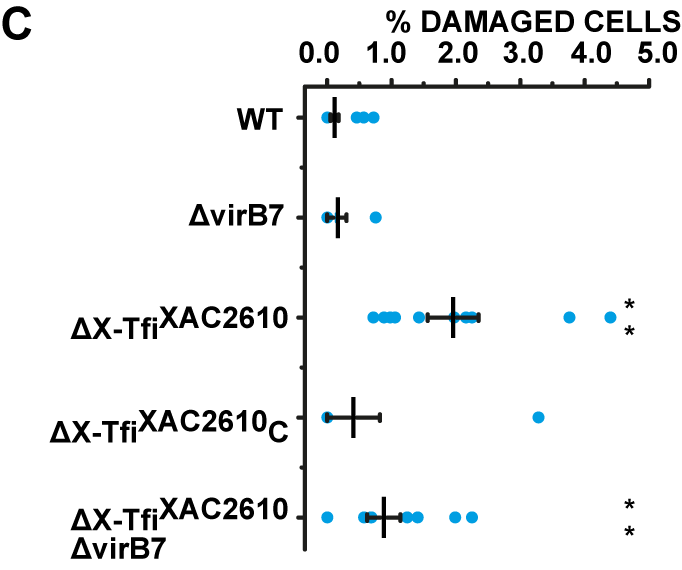

Supplement: Supplementary file 12 — Source Data Fig. 4 [file 44319_2024_60_MOESM12_ESM.zip › Fig 4/4C/4C.tif]

**C**

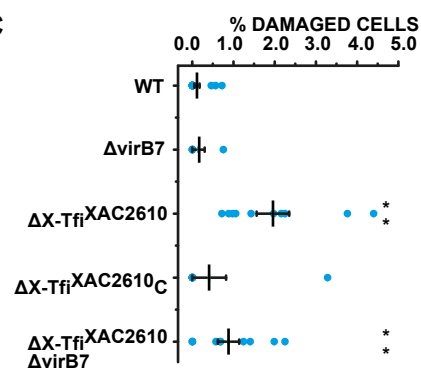

Supplement: Supplementary file 12 — Source Data Fig. 4 [file 44319_2024_60_MOESM12_ESM.zip › Fig 4/4C/4C.pdf]

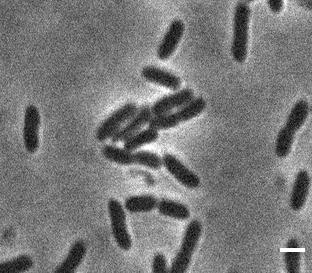

Supplement: Supplementary file 12 — Source Data Fig. 4 [file 44319_2024_60_MOESM12_ESM.zip › Fig 4/4A/micrographs/T=0 Example_C_.tif]

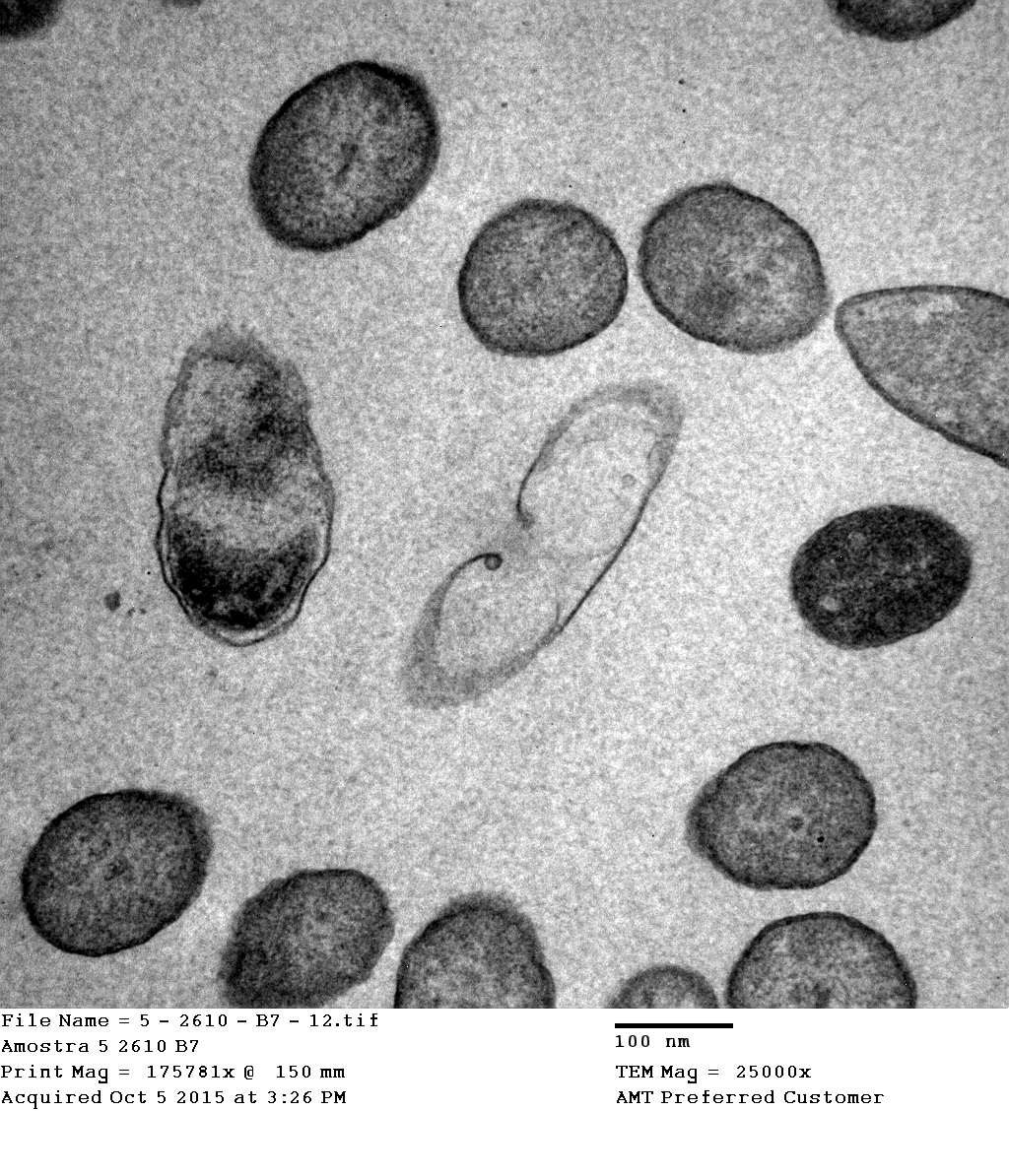

Supplement: Supplementary file 12 — Source Data Fig. 4 [file 44319_2024_60_MOESM12_ESM.zip › Fig 4/4C/raw images/5 - 2610 - B7 - 12.tif]

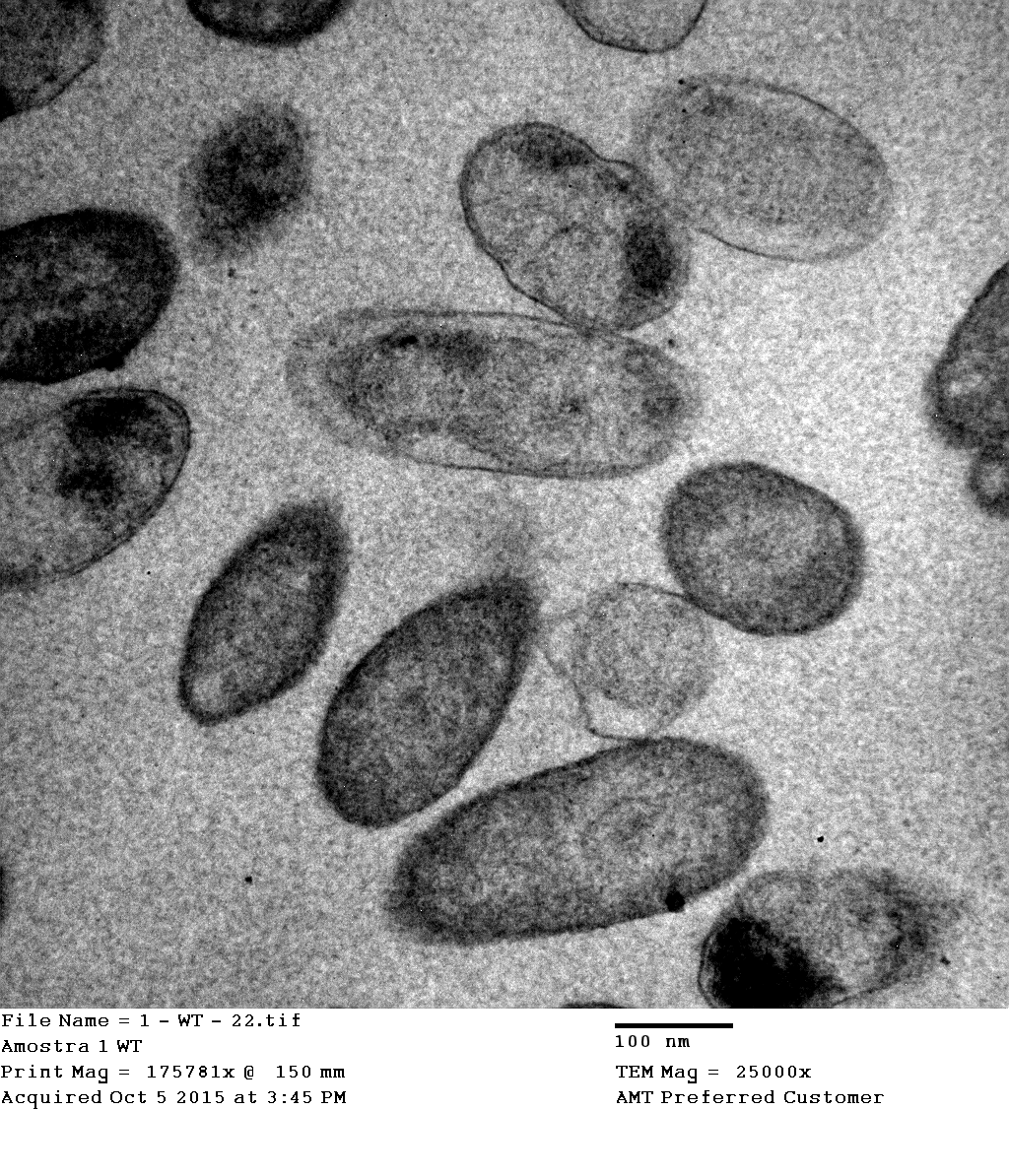

Supplement: Supplementary file 12 — Source Data Fig. 4 [file 44319_2024_60_MOESM12_ESM.zip › Fig 4/4C/raw images/1 - WT - 22.tif]

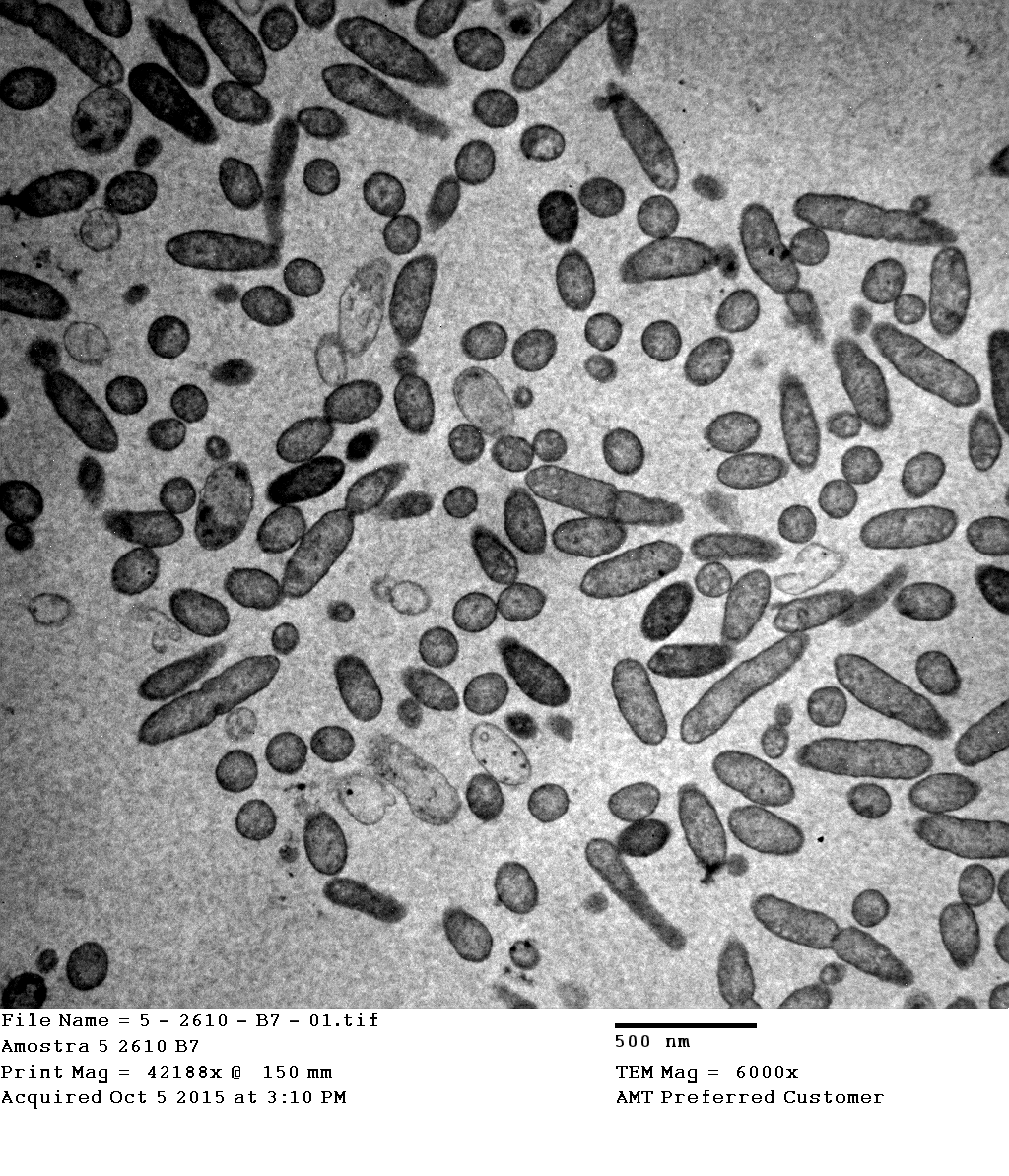

Supplement: Supplementary file 12 — Source Data Fig. 4 [file 44319_2024_60_MOESM12_ESM.zip › Fig 4/4C/raw images/5 - 2610 - B7 - 01.tif]

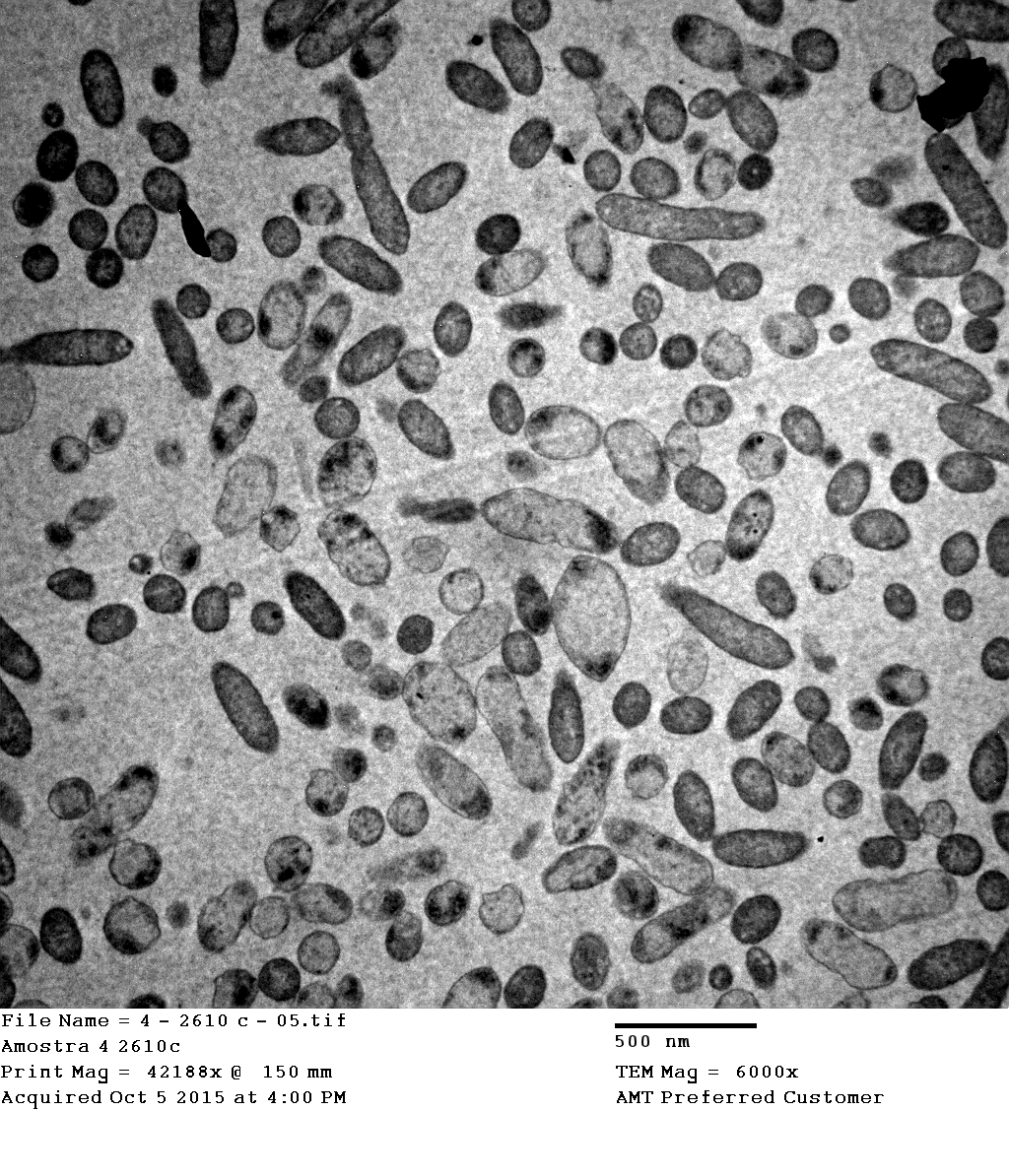

Supplement: Supplementary file 12 — Source Data Fig. 4 [file 44319_2024_60_MOESM12_ESM.zip › Fig 4/4C/raw images/4 - 2610 c - 05.tif]

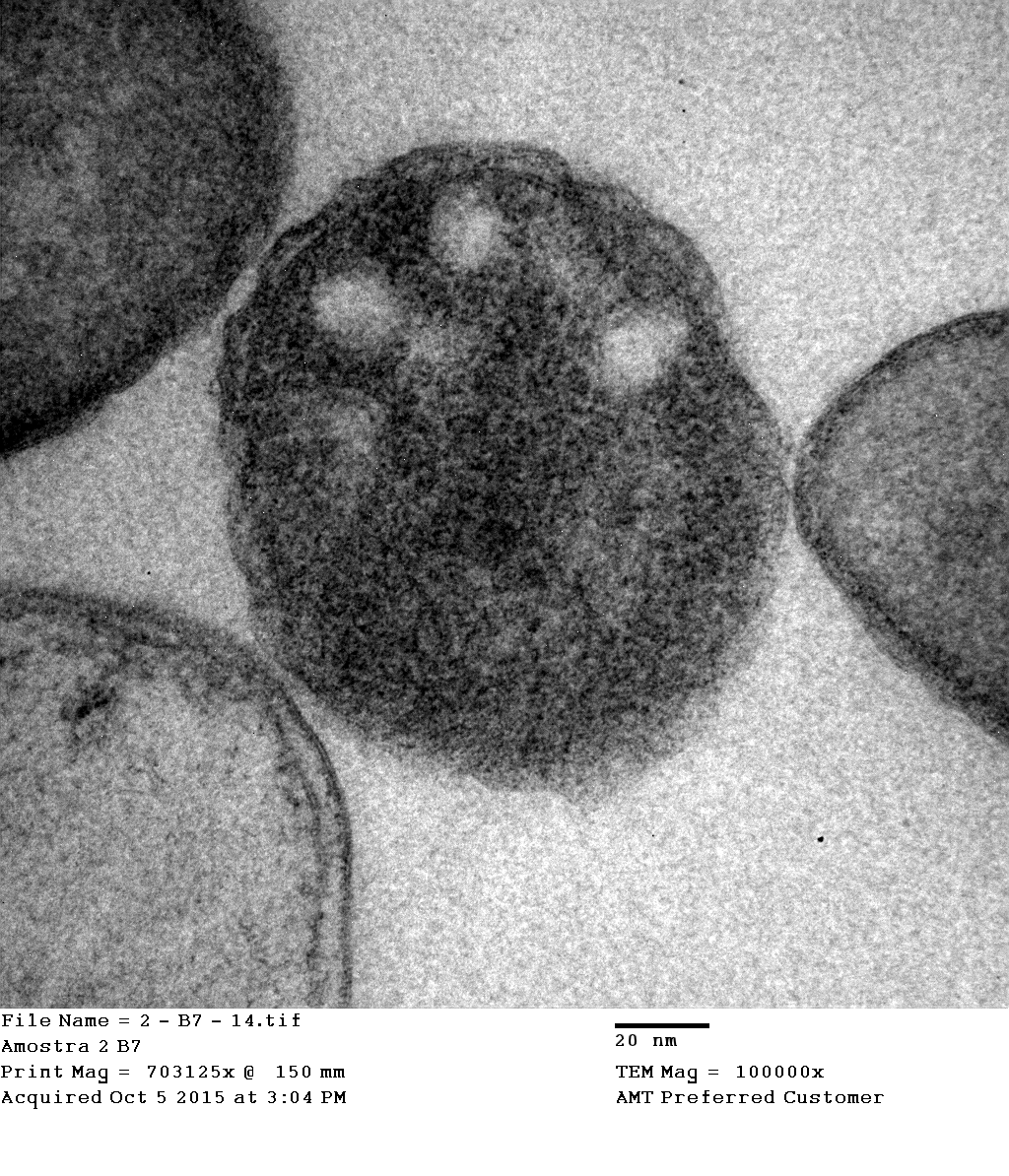

Supplement: Supplementary file 12 — Source Data Fig. 4 [file 44319_2024_60_MOESM12_ESM.zip › Fig 4/4C/raw images/2 - B7 - 14.tif]

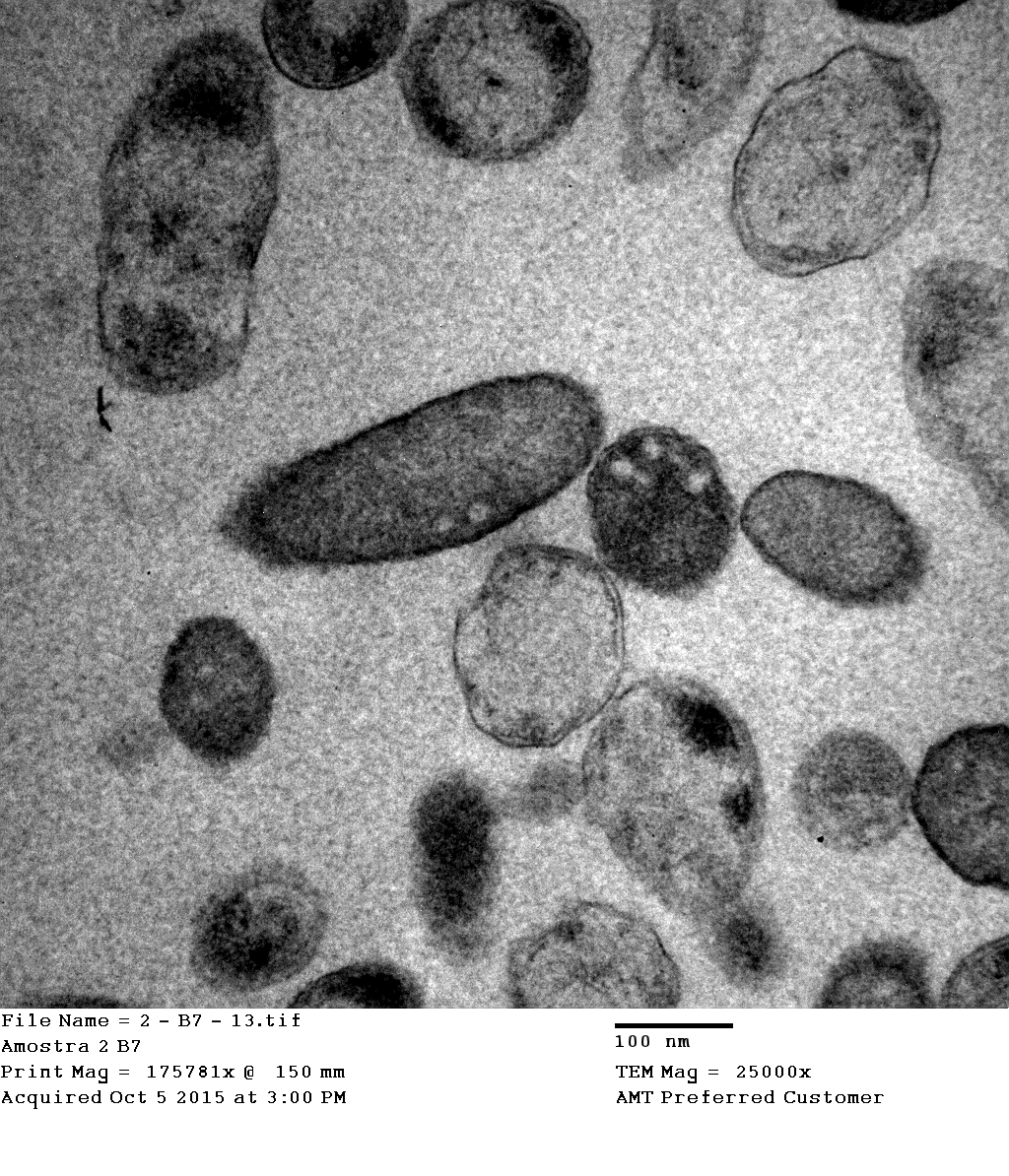

Supplement: Supplementary file 12 — Source Data Fig. 4 [file 44319_2024_60_MOESM12_ESM.zip › Fig 4/4C/raw images/2 - B7 - 13.tif]

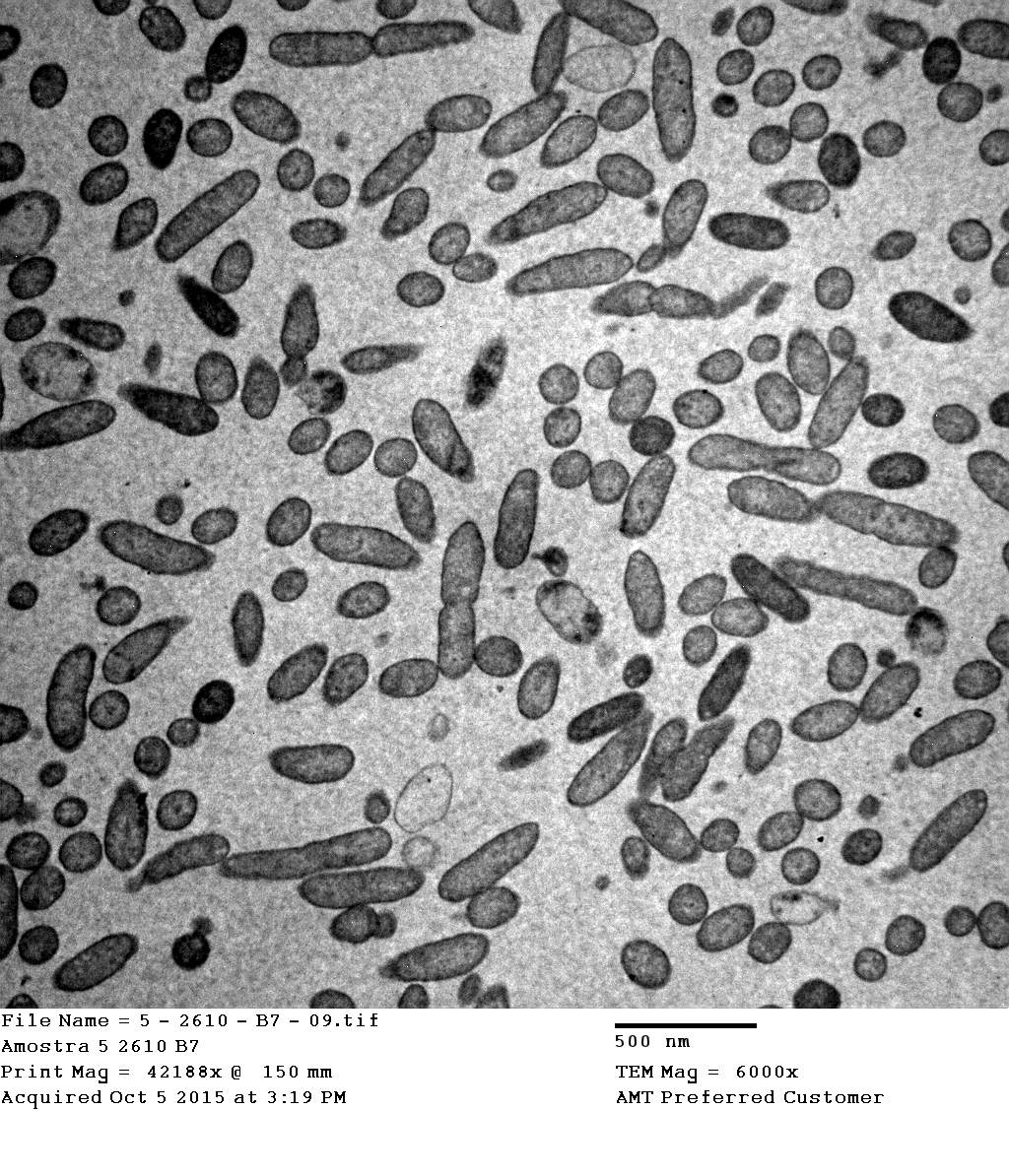

Supplement: Supplementary file 12 — Source Data Fig. 4 [file 44319_2024_60_MOESM12_ESM.zip › Fig 4/4C/raw images/5 - 2610 - B7 - 09.tif]

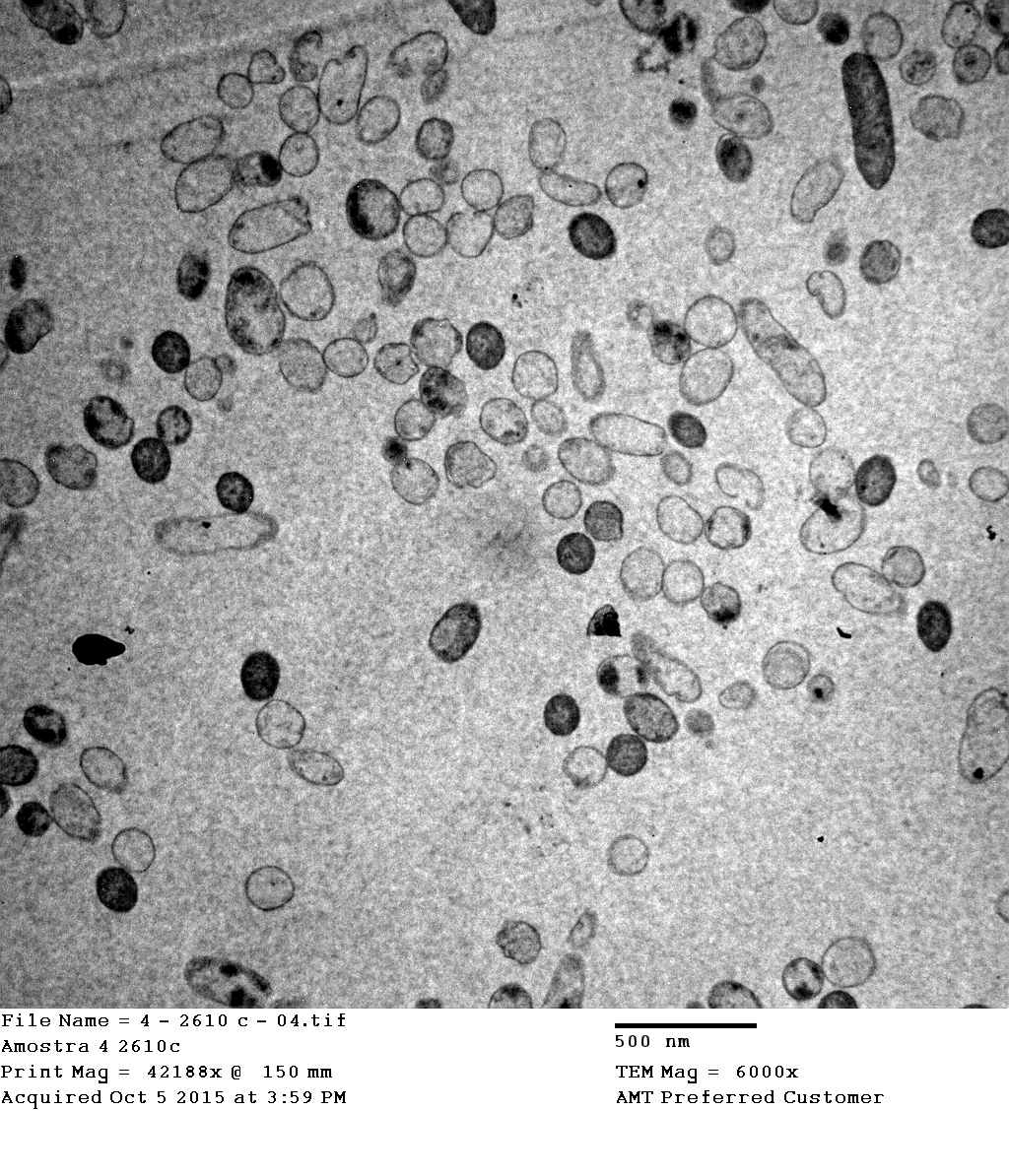

Supplement: Supplementary file 12 — Source Data Fig. 4 [file 44319_2024_60_MOESM12_ESM.zip › Fig 4/4C/raw images/4 - 2610 c - 04.tif]

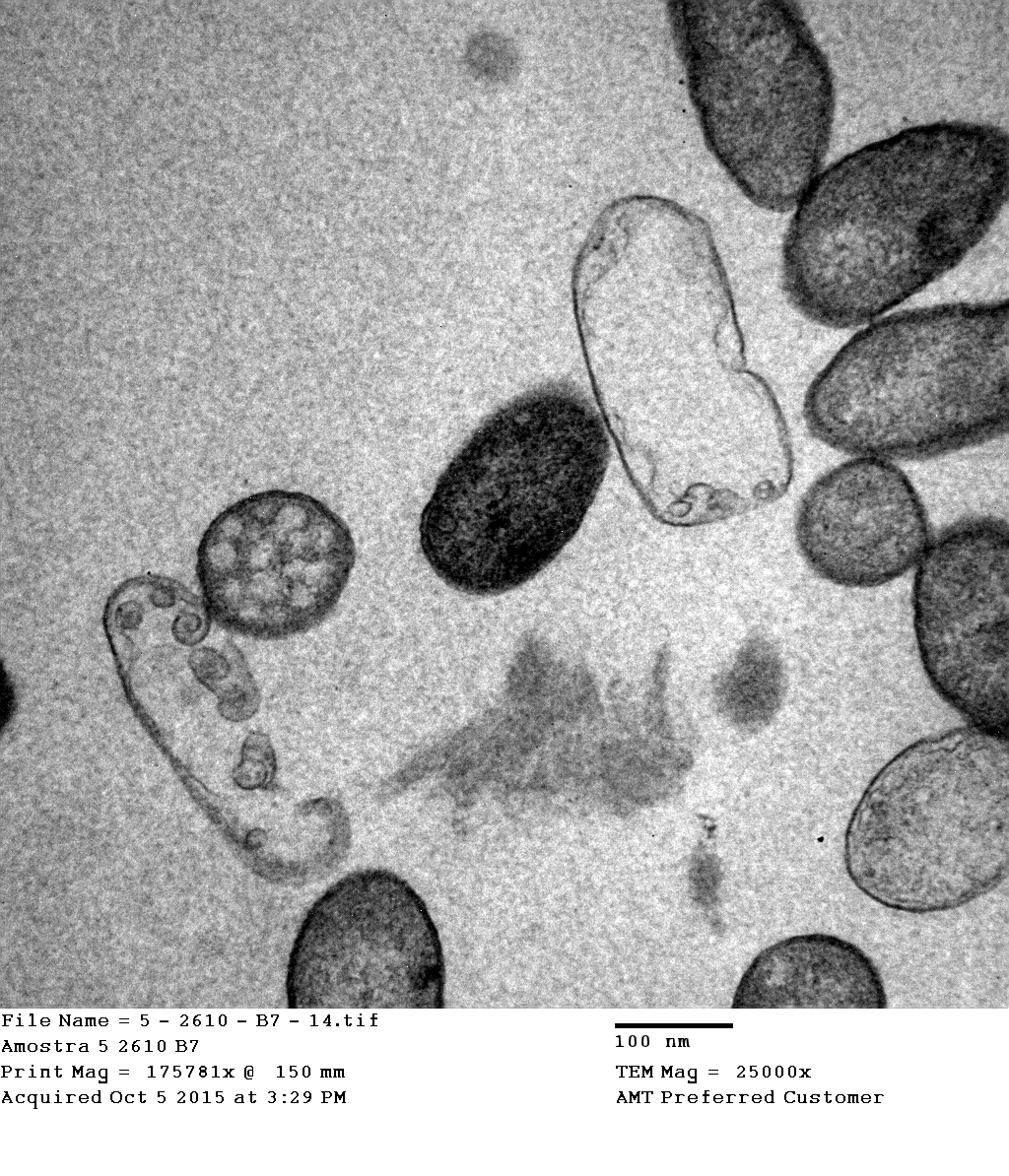

Supplement: Supplementary file 12 — Source Data Fig. 4 [file 44319_2024_60_MOESM12_ESM.zip › Fig 4/4C/raw images/5 - 2610 - B7 - 14.tif]

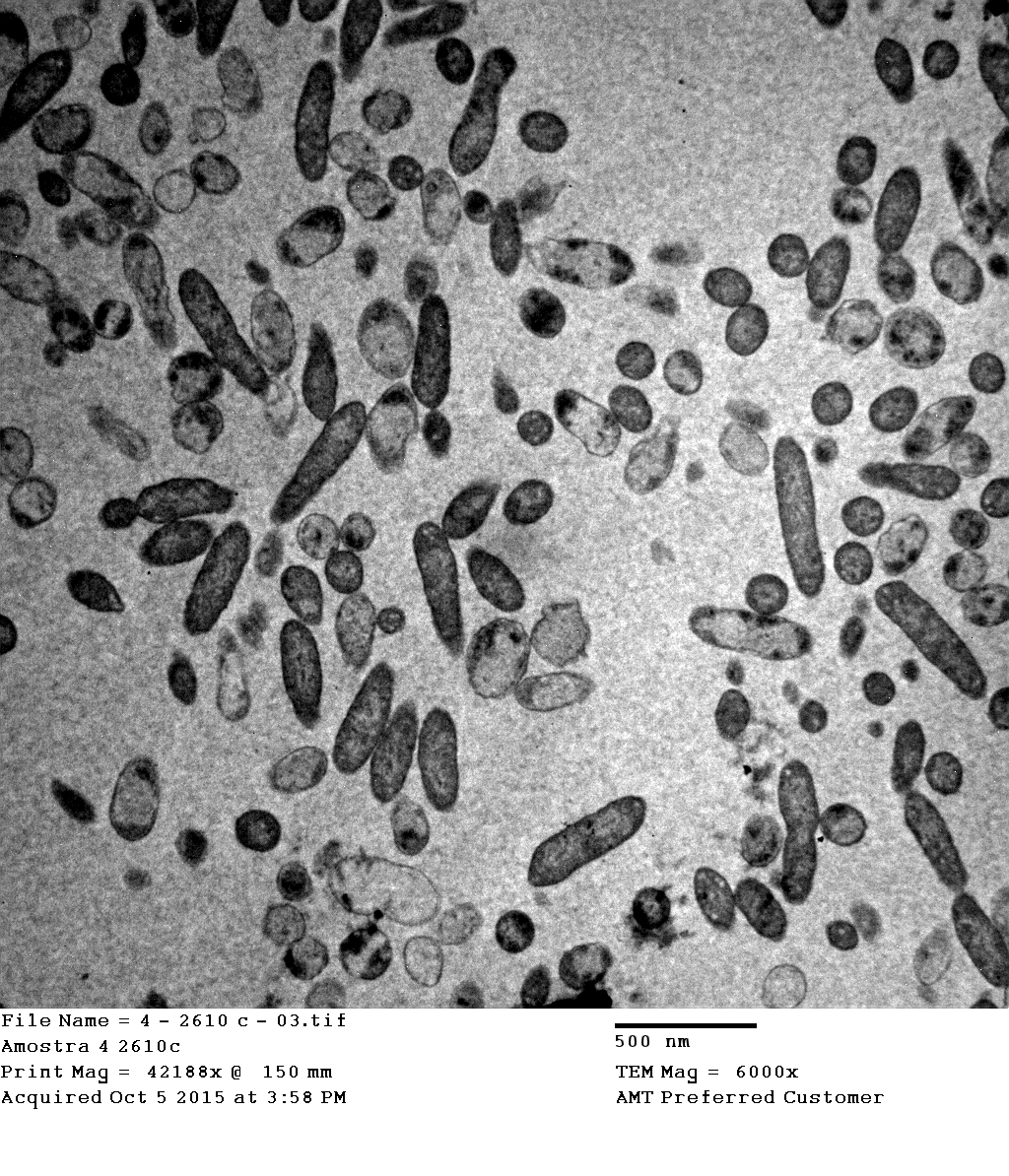

Supplement: Supplementary file 12 — Source Data Fig. 4 [file 44319_2024_60_MOESM12_ESM.zip › Fig 4/4C/raw images/4 - 2610 c - 03.tif]

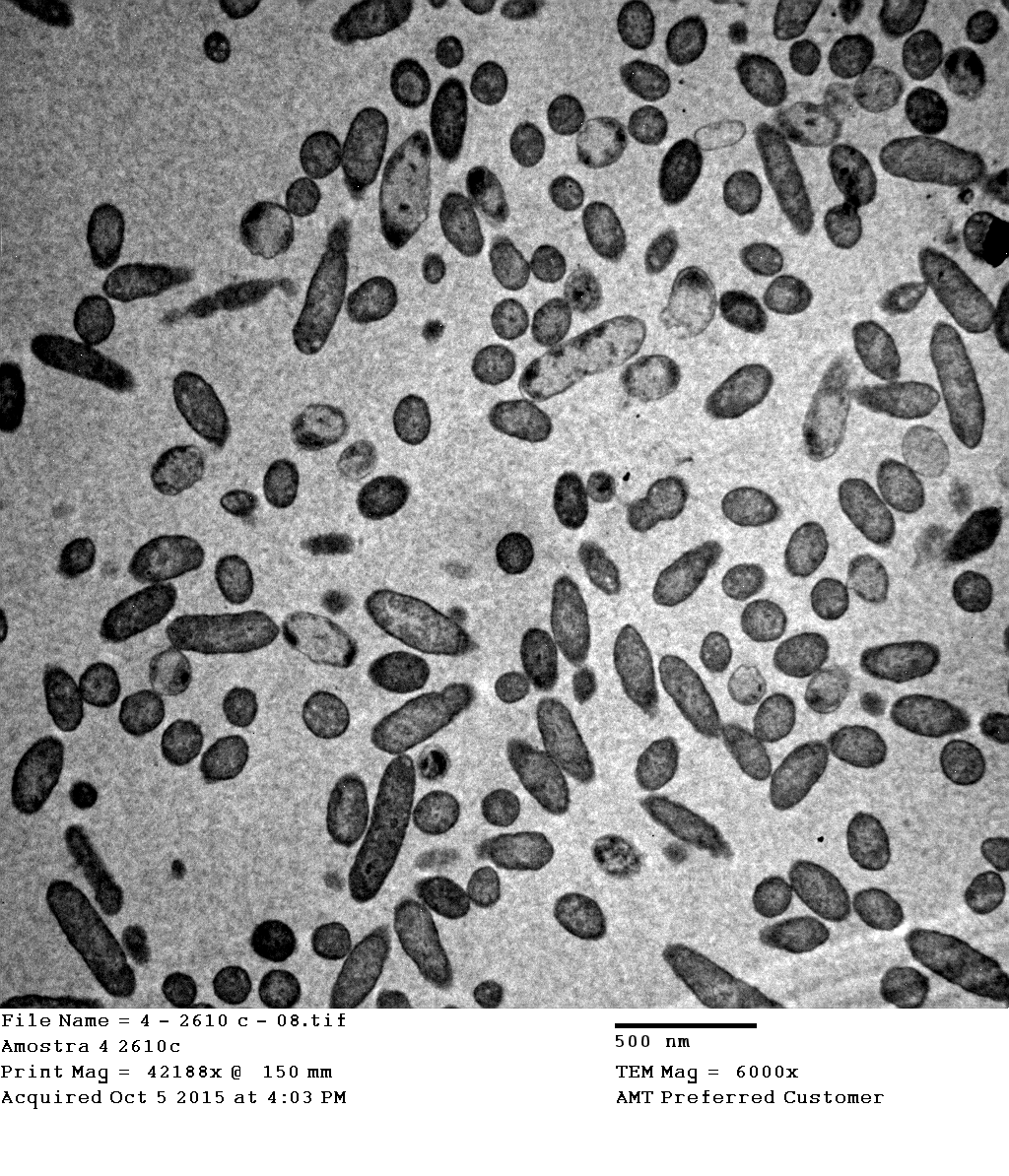

Supplement: Supplementary file 12 — Source Data Fig. 4 [file 44319_2024_60_MOESM12_ESM.zip › Fig 4/4C/raw images/4 - 2610 c - 08.tif]

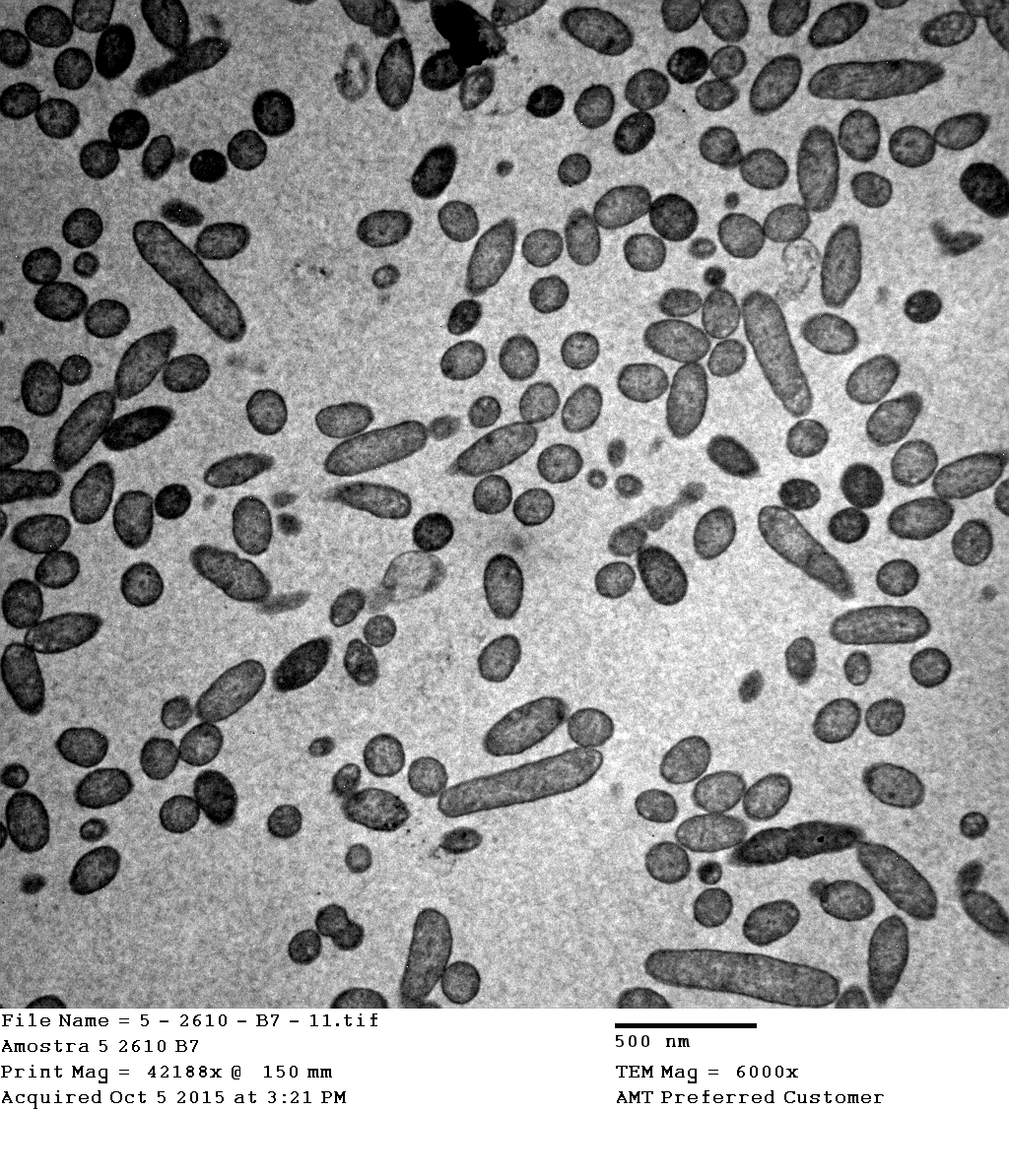

Supplement: Supplementary file 12 — Source Data Fig. 4 [file 44319_2024_60_MOESM12_ESM.zip › Fig 4/4C/raw images/5 - 2610 - B7 - 11.tif]

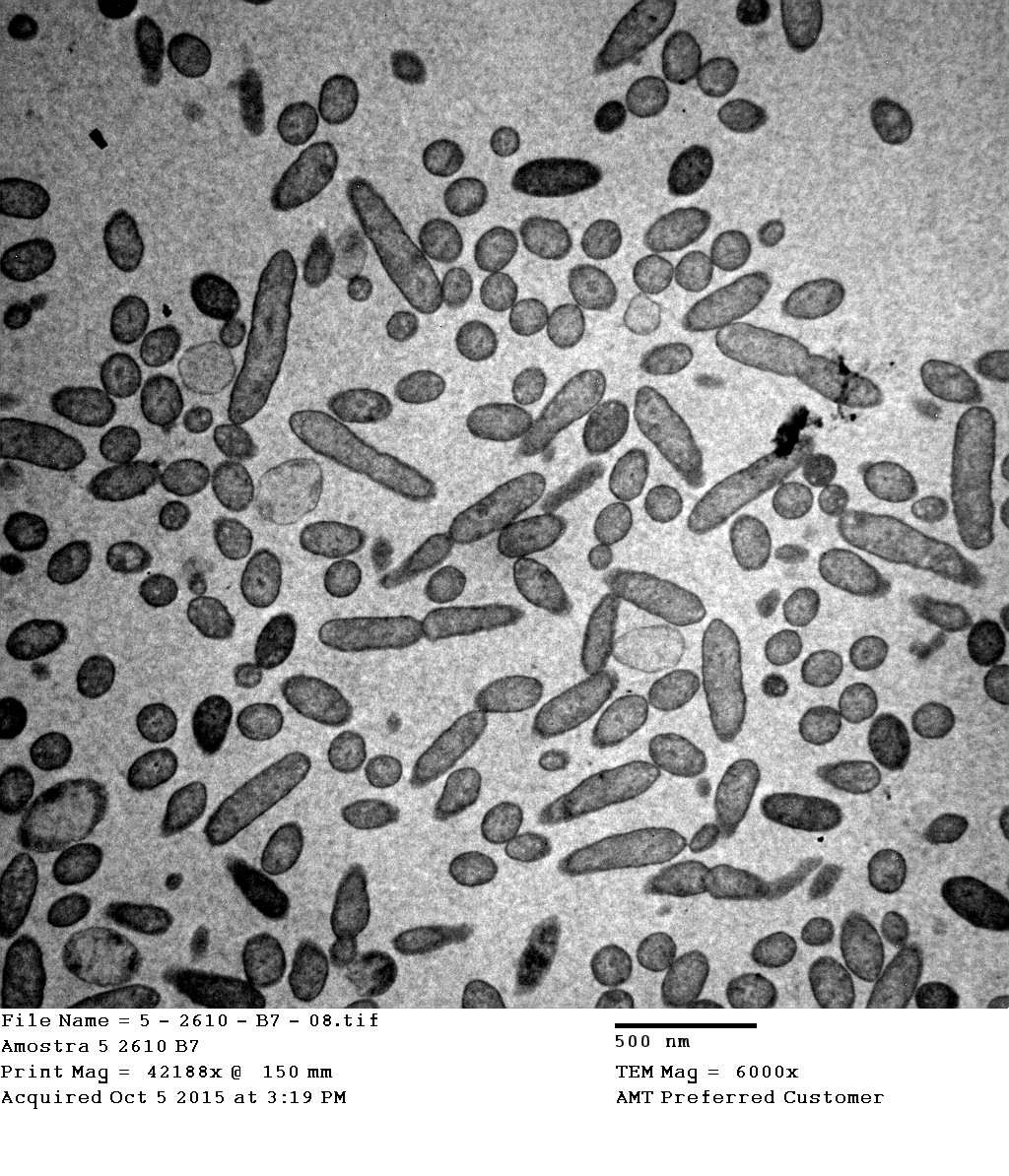

Supplement: Supplementary file 12 — Source Data Fig. 4 [file 44319_2024_60_MOESM12_ESM.zip › Fig 4/4C/raw images/5 - 2610 - B7 - 08.tif]

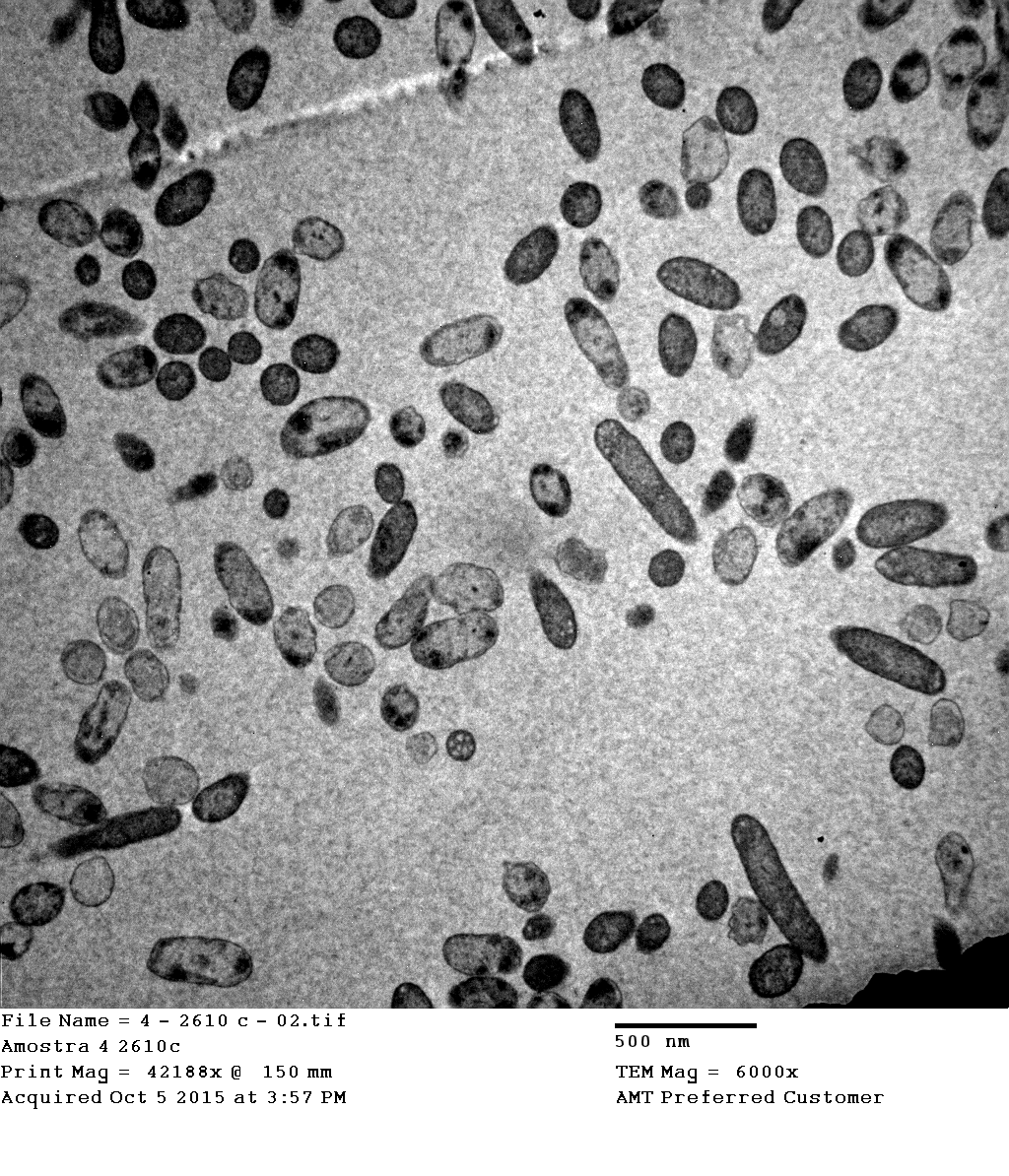

Supplement: Supplementary file 12 — Source Data Fig. 4 [file 44319_2024_60_MOESM12_ESM.zip › Fig 4/4C/raw images/4 - 2610 c - 02.tif]

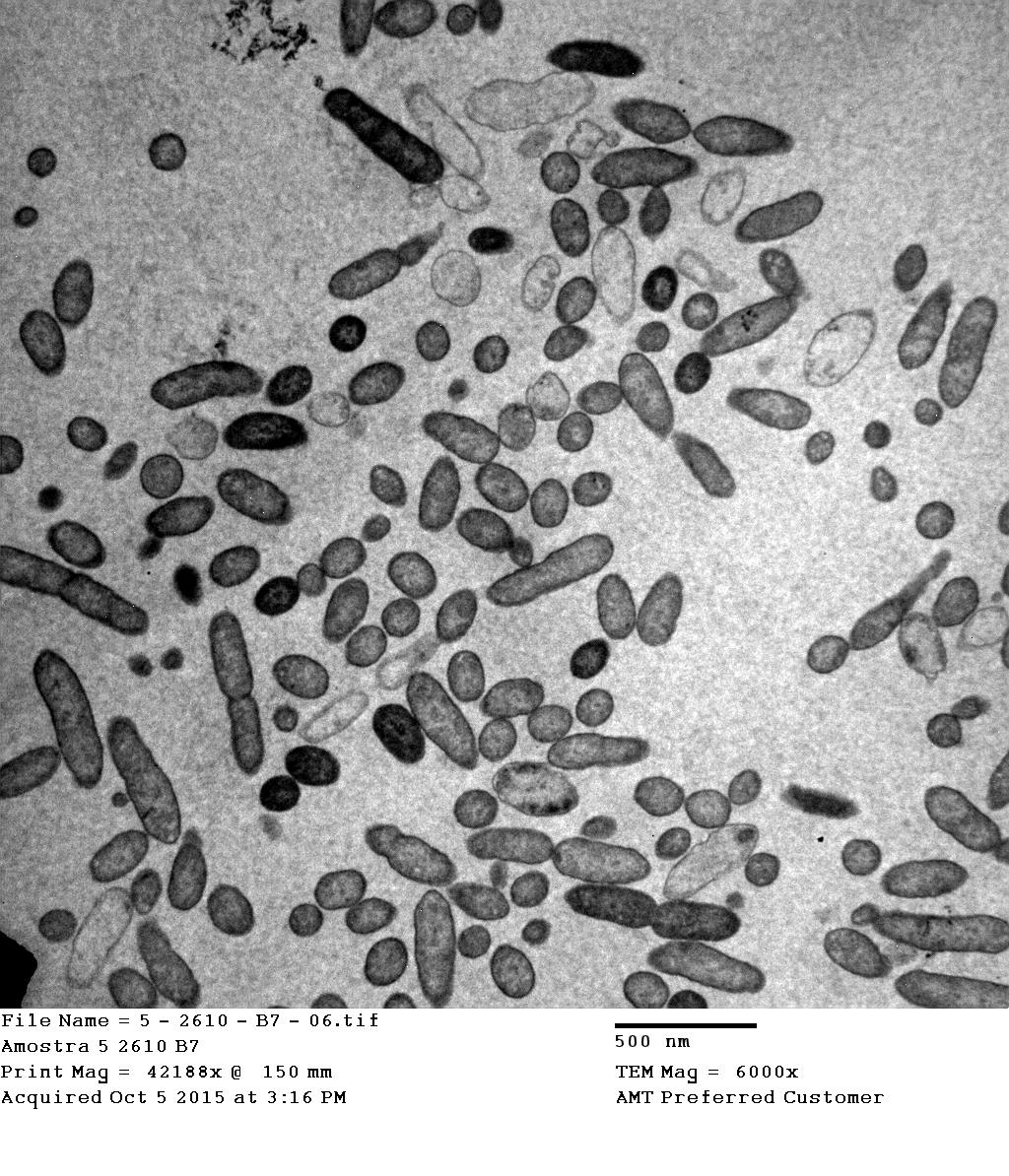

Supplement: Supplementary file 12 — Source Data Fig. 4 [file 44319_2024_60_MOESM12_ESM.zip › Fig 4/4C/raw images/5 - 2610 - B7 - 06.tif]

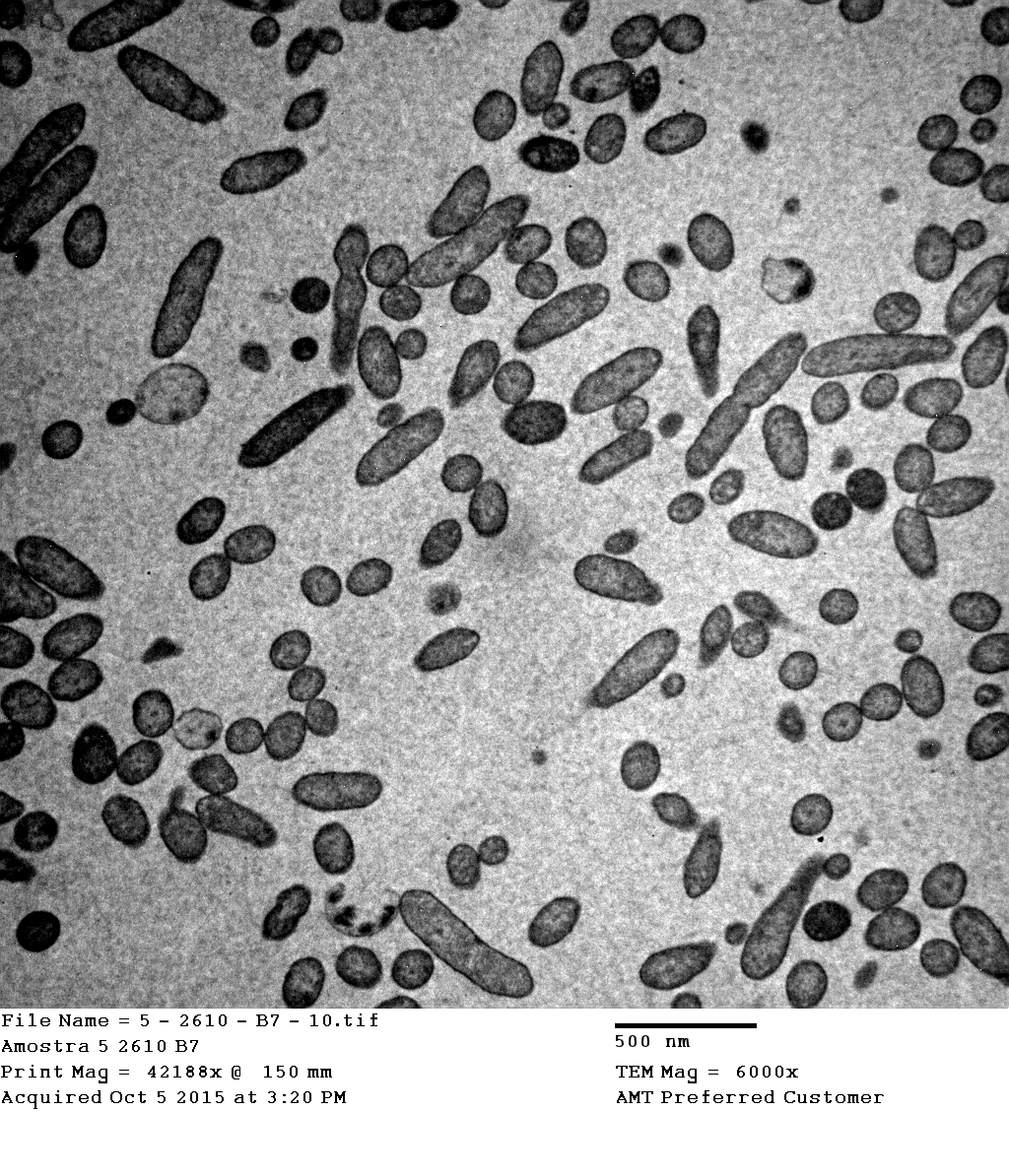

Supplement: Supplementary file 12 — Source Data Fig. 4 [file 44319_2024_60_MOESM12_ESM.zip › Fig 4/4C/raw images/5 - 2610 - B7 - 10.tif]

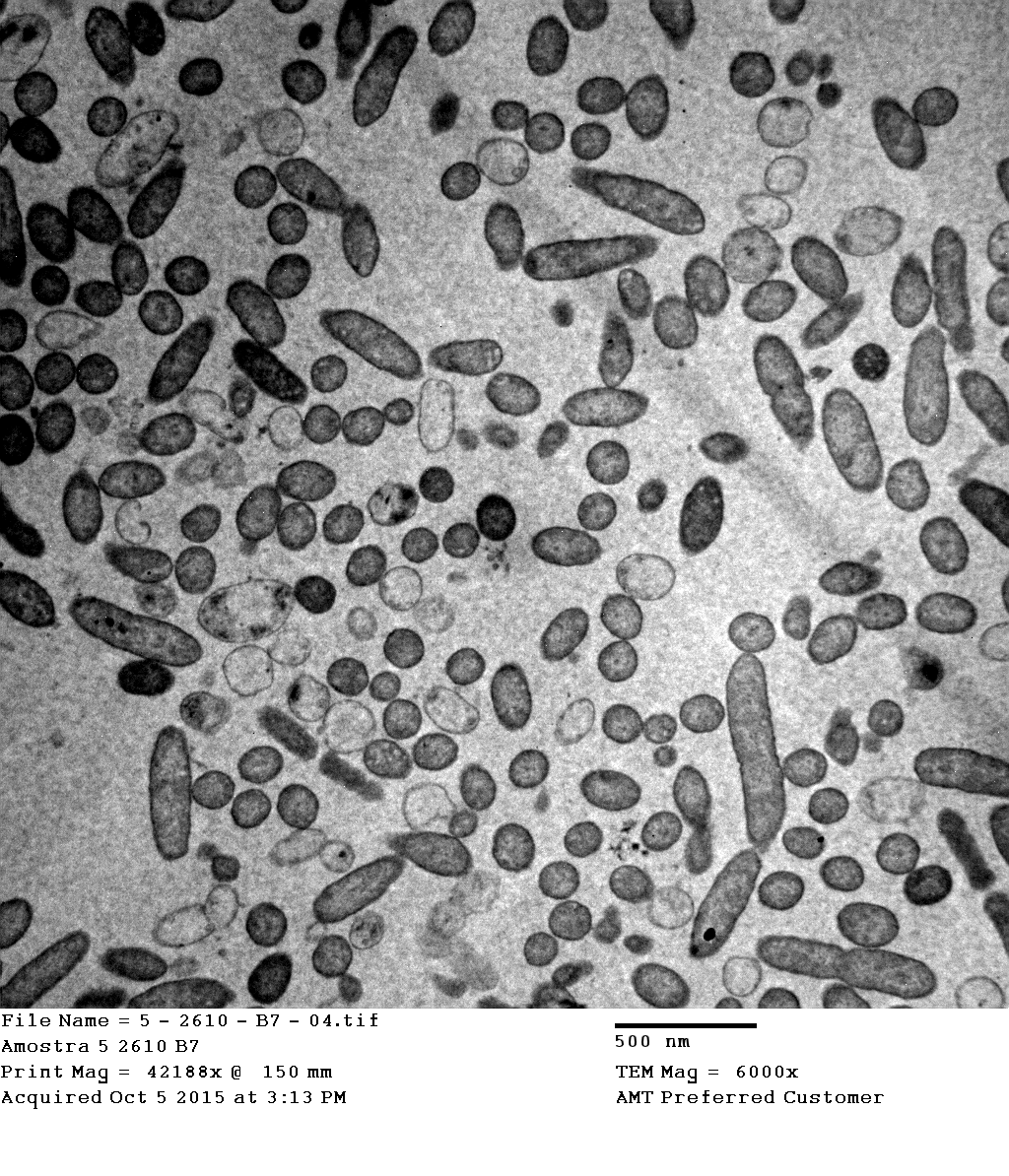

Supplement: Supplementary file 12 — Source Data Fig. 4 [file 44319_2024_60_MOESM12_ESM.zip › Fig 4/4C/raw images/5 - 2610 - B7 - 04.tif]

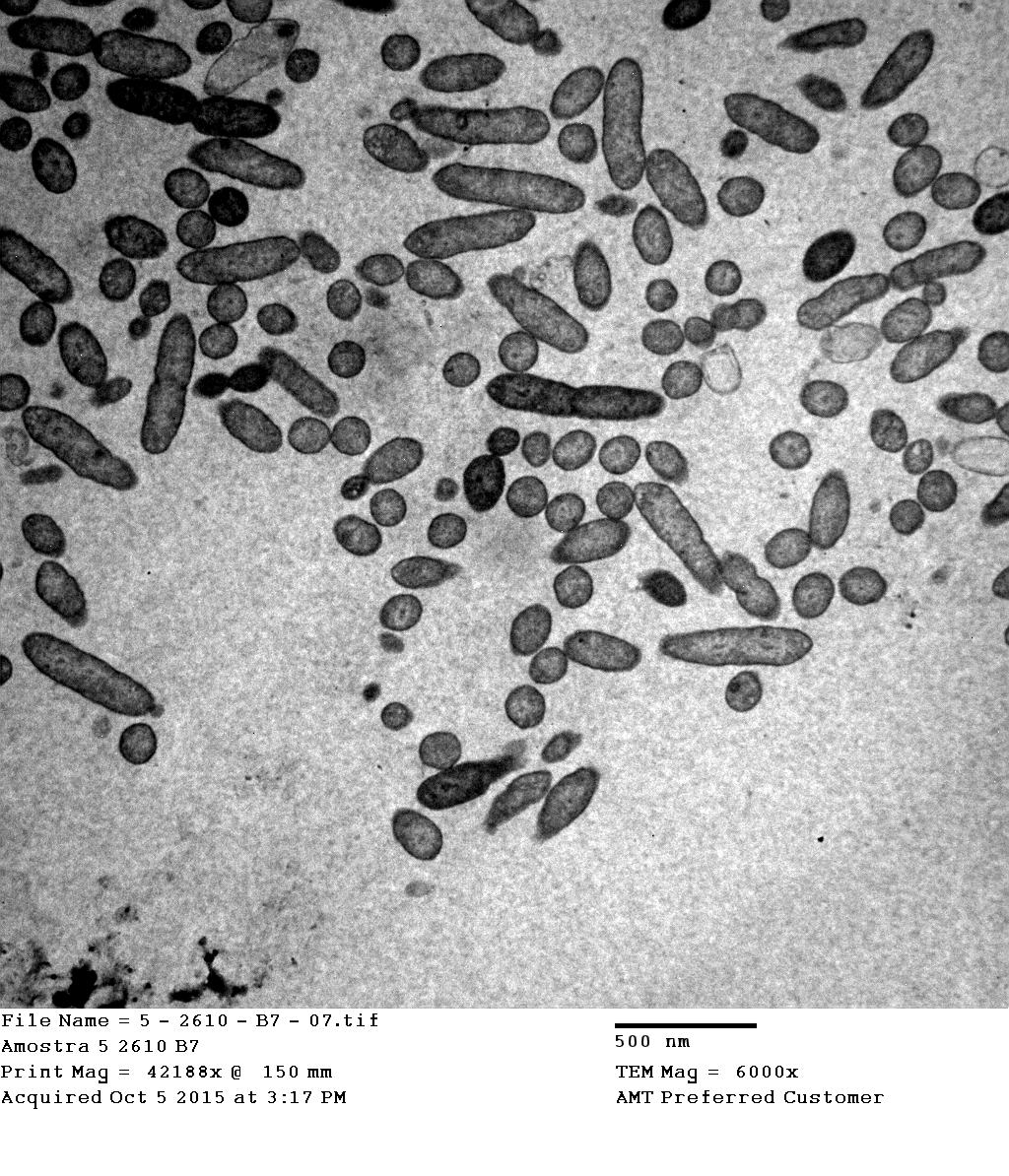

Supplement: Supplementary file 12 — Source Data Fig. 4 [file 44319_2024_60_MOESM12_ESM.zip › Fig 4/4C/raw images/5 - 2610 - B7 - 07.tif]

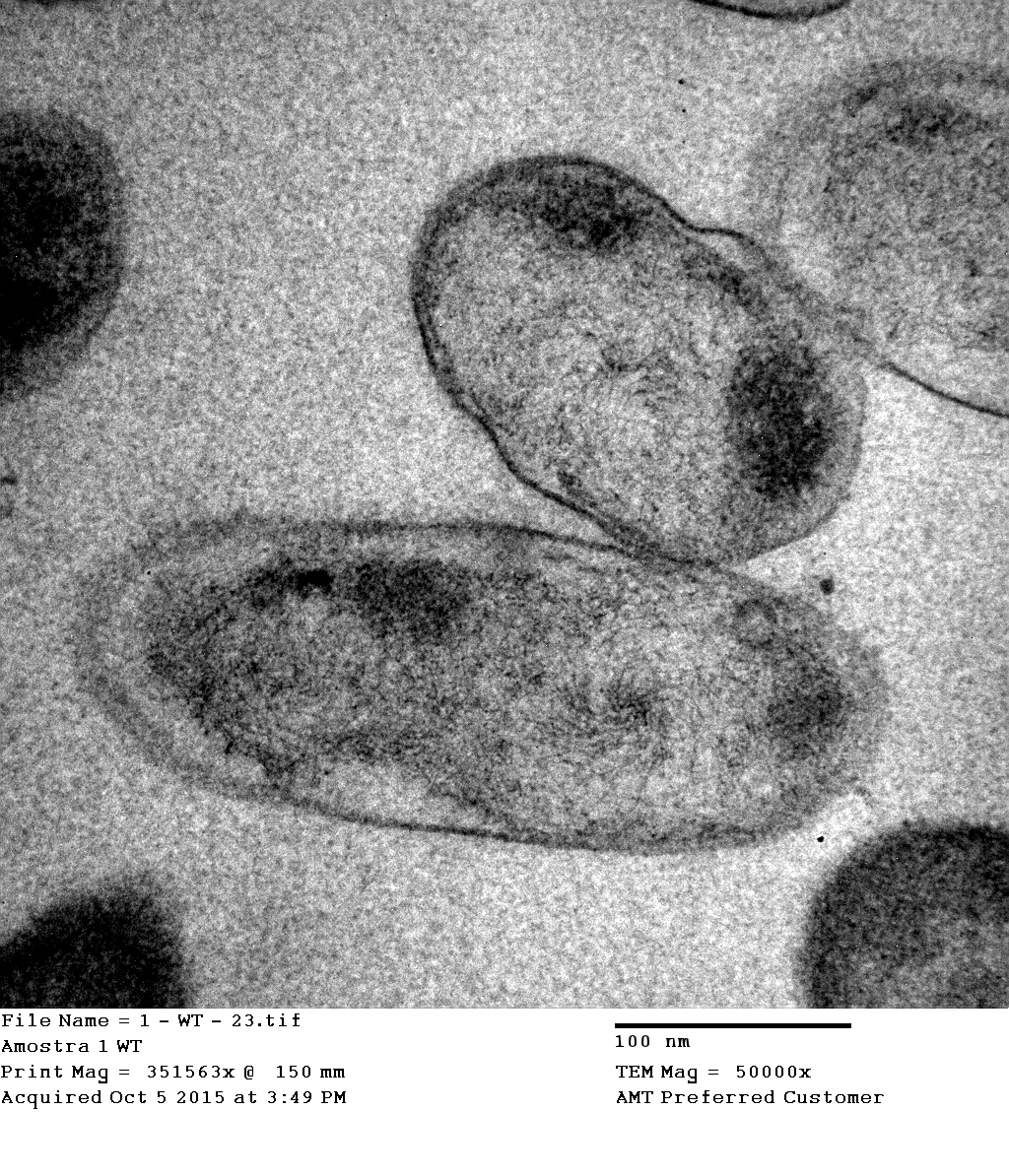

Supplement: Supplementary file 12 — Source Data Fig. 4 [file 44319_2024_60_MOESM12_ESM.zip › Fig 4/4C/raw images/1 - WT - 23.tif]

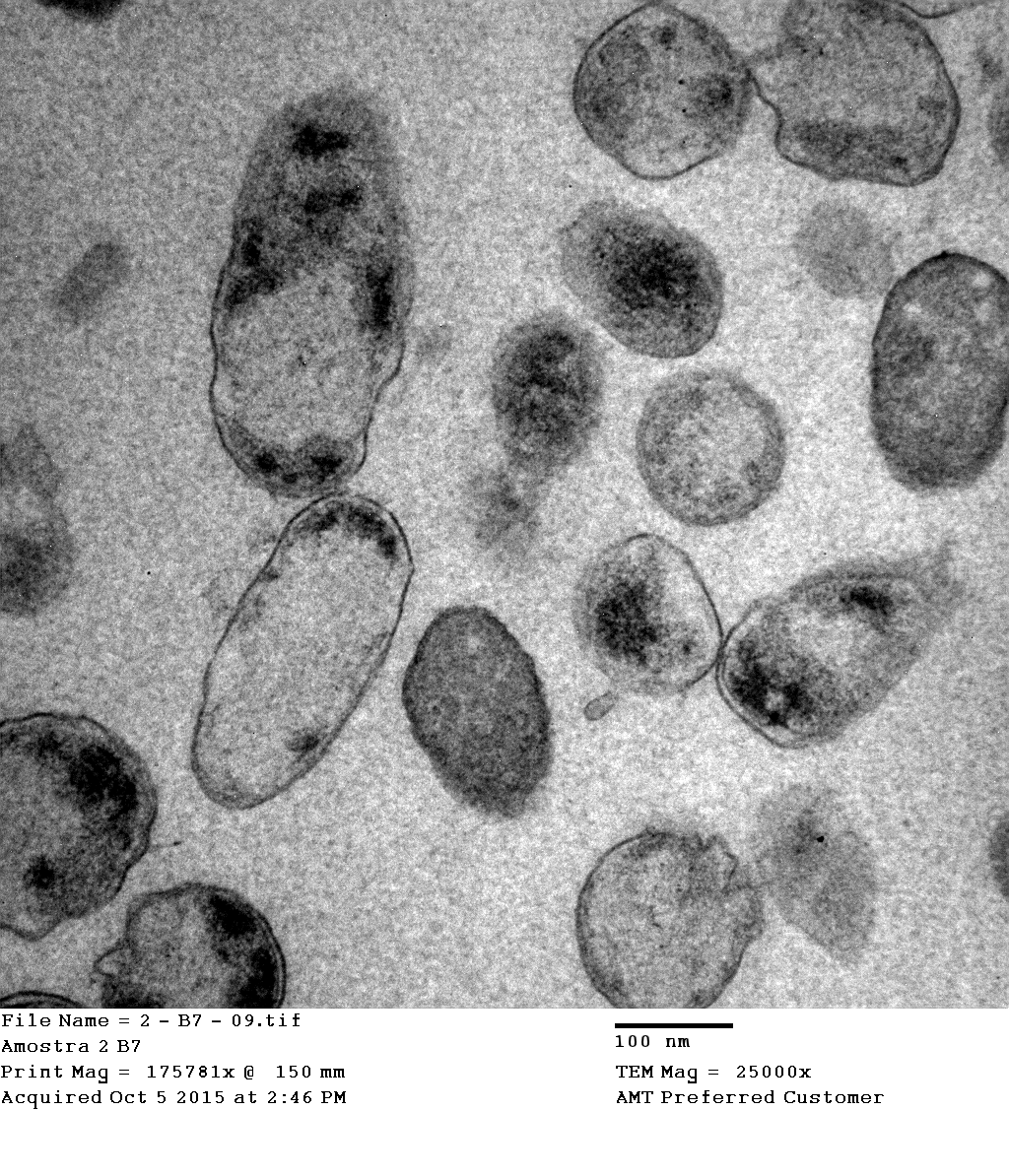

Supplement: Supplementary file 12 — Source Data Fig. 4 [file 44319_2024_60_MOESM12_ESM.zip › Fig 4/4C/raw images/2 - B7 - 09.tif]

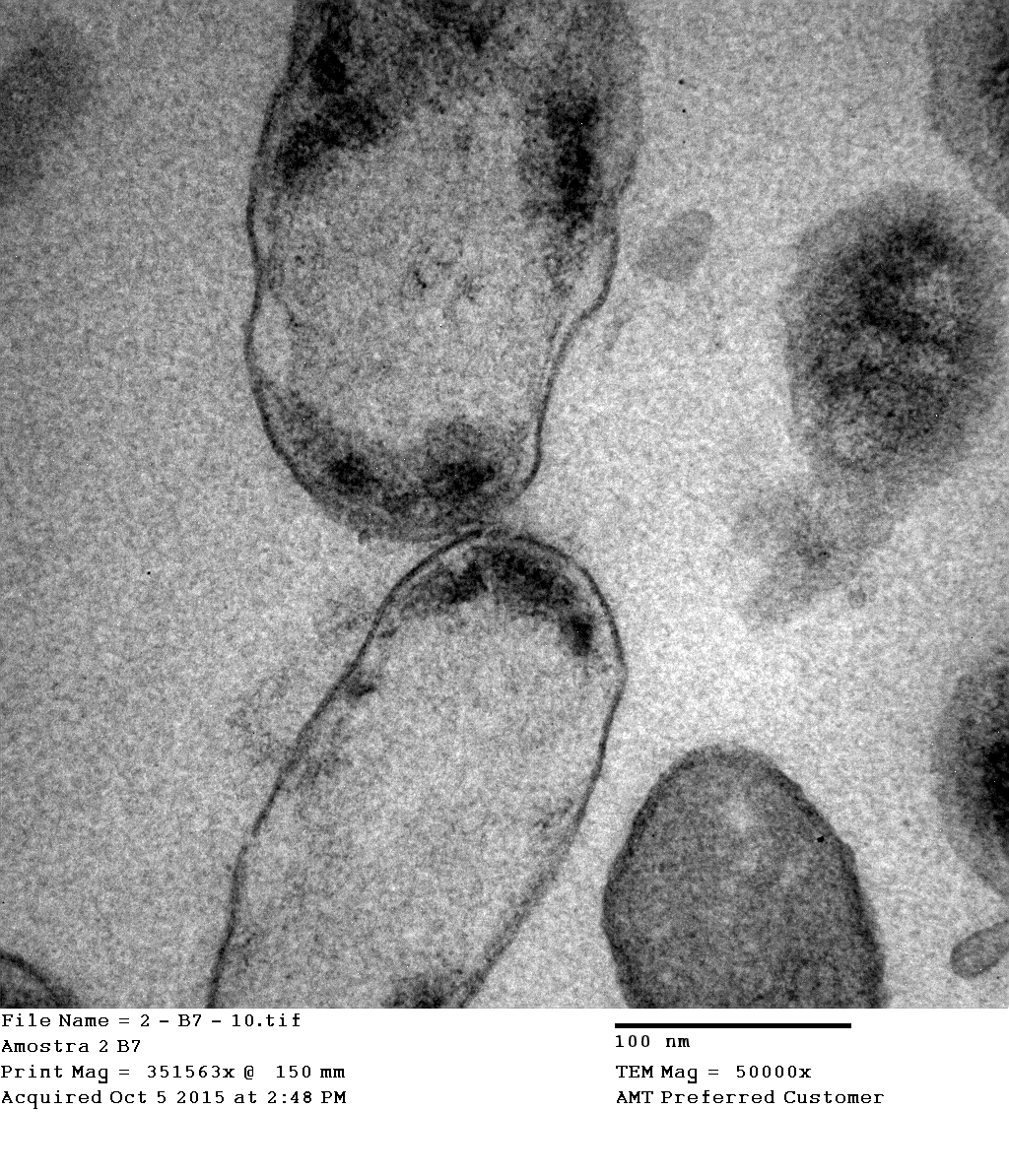

Supplement: Supplementary file 12 — Source Data Fig. 4 [file 44319_2024_60_MOESM12_ESM.zip › Fig 4/4C/raw images/2 - B7 - 10.tif]

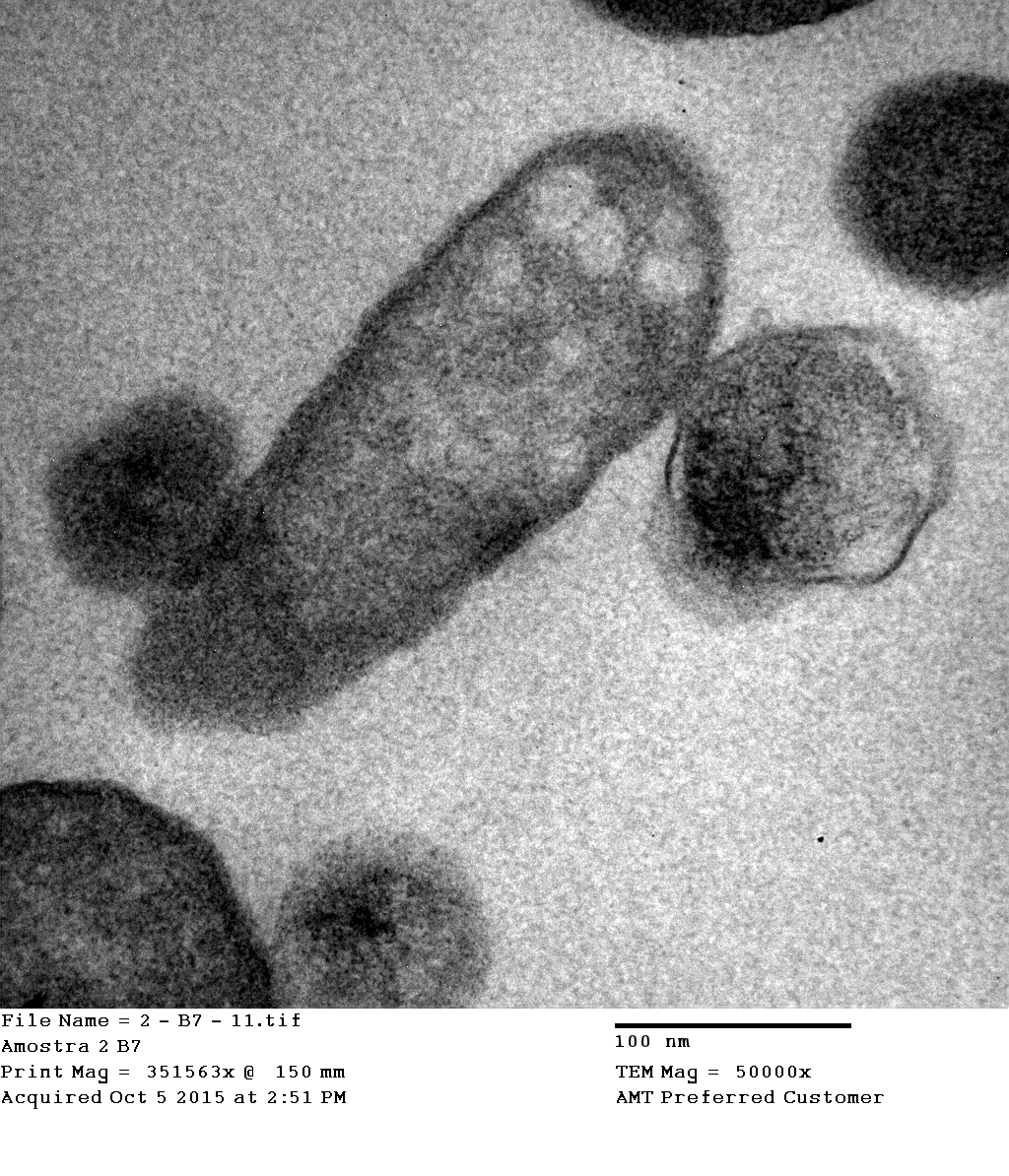

Supplement: Supplementary file 12 — Source Data Fig. 4 [file 44319_2024_60_MOESM12_ESM.zip › Fig 4/4C/raw images/2 - B7 - 11.tif]

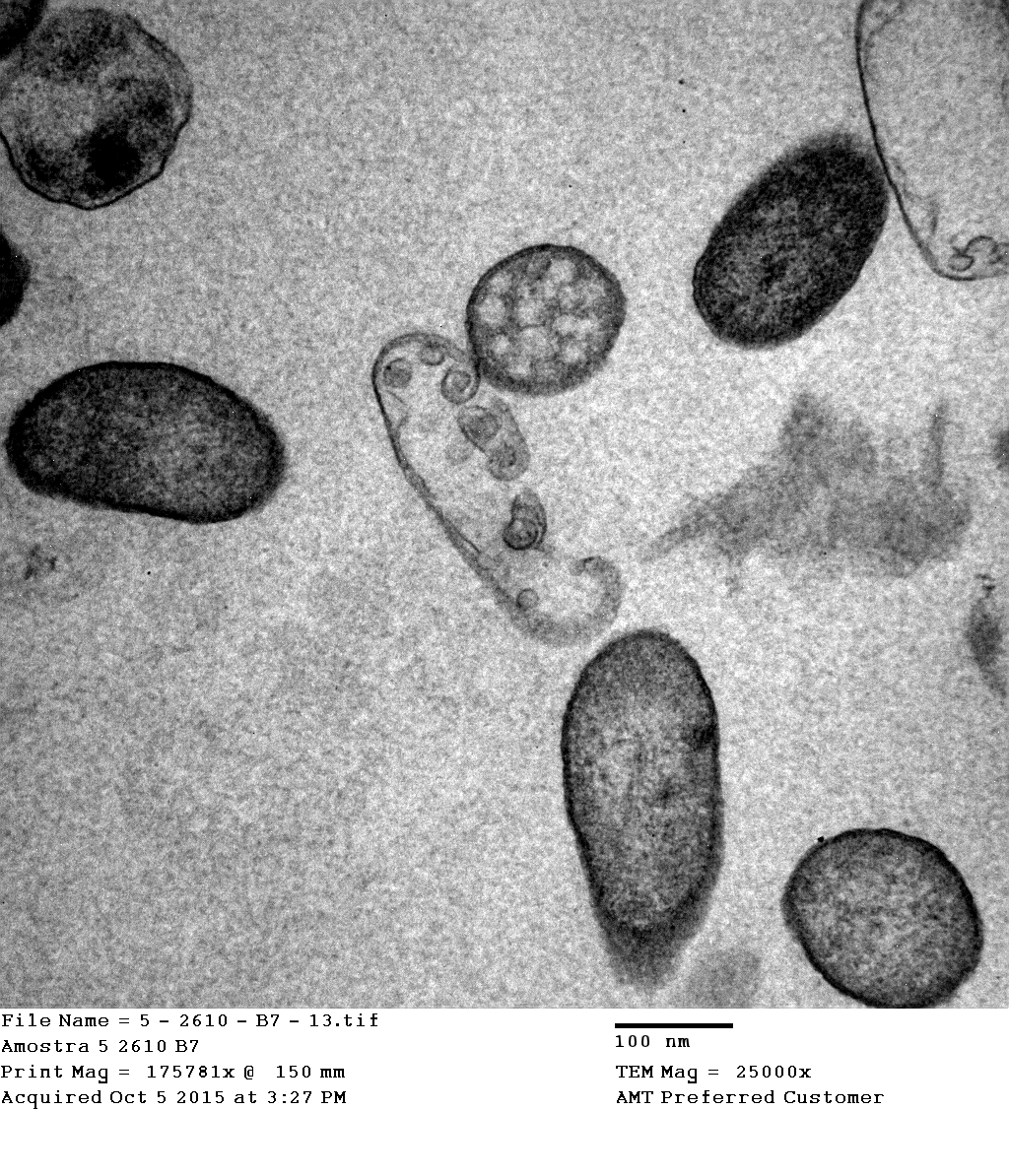

Supplement: Supplementary file 12 — Source Data Fig. 4 [file 44319_2024_60_MOESM12_ESM.zip › Fig 4/4C/raw images/5 - 2610 - B7 - 13.tif]

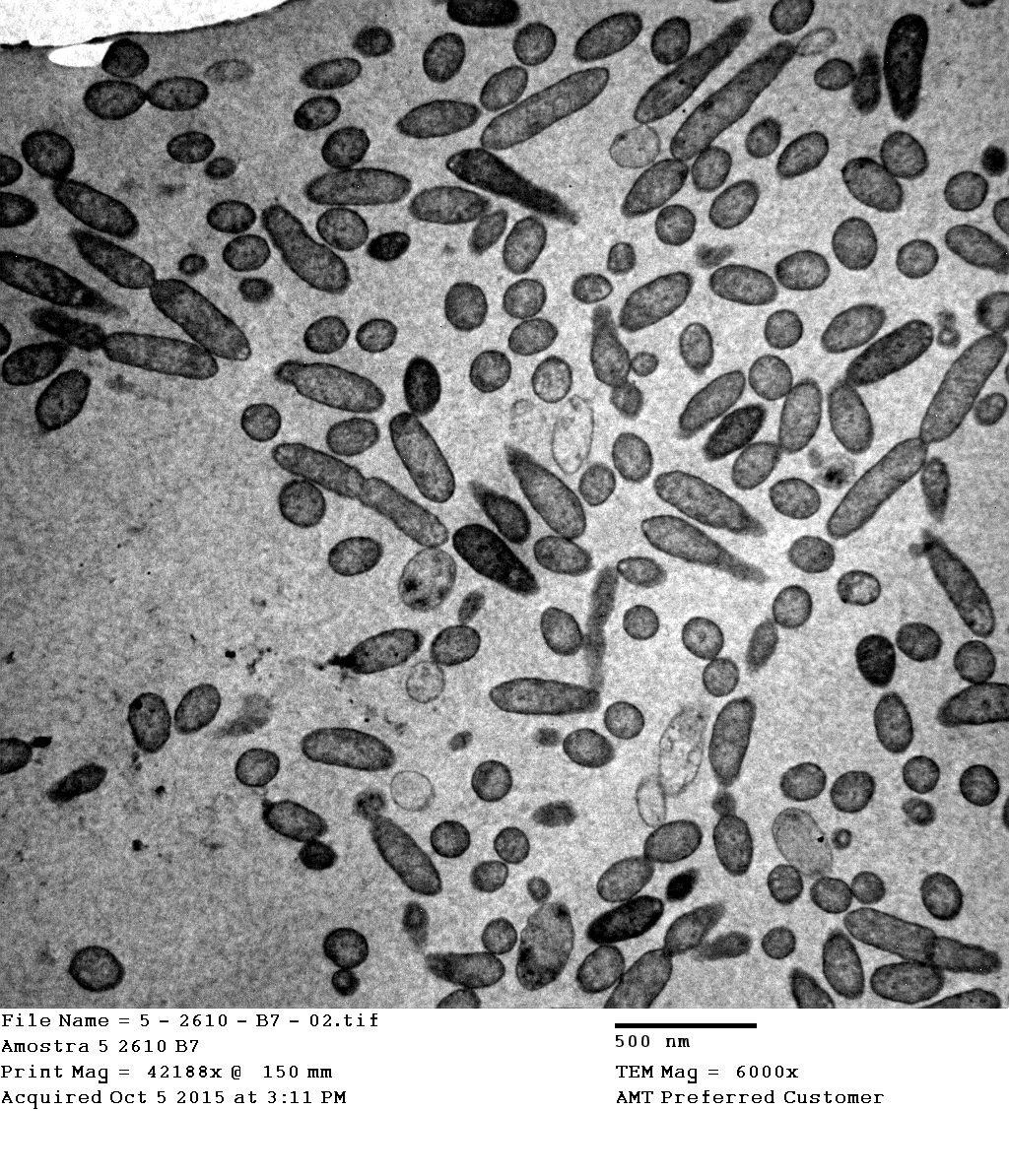

Supplement: Supplementary file 12 — Source Data Fig. 4 [file 44319_2024_60_MOESM12_ESM.zip › Fig 4/4C/raw images/5 - 2610 - B7 - 02.tif]

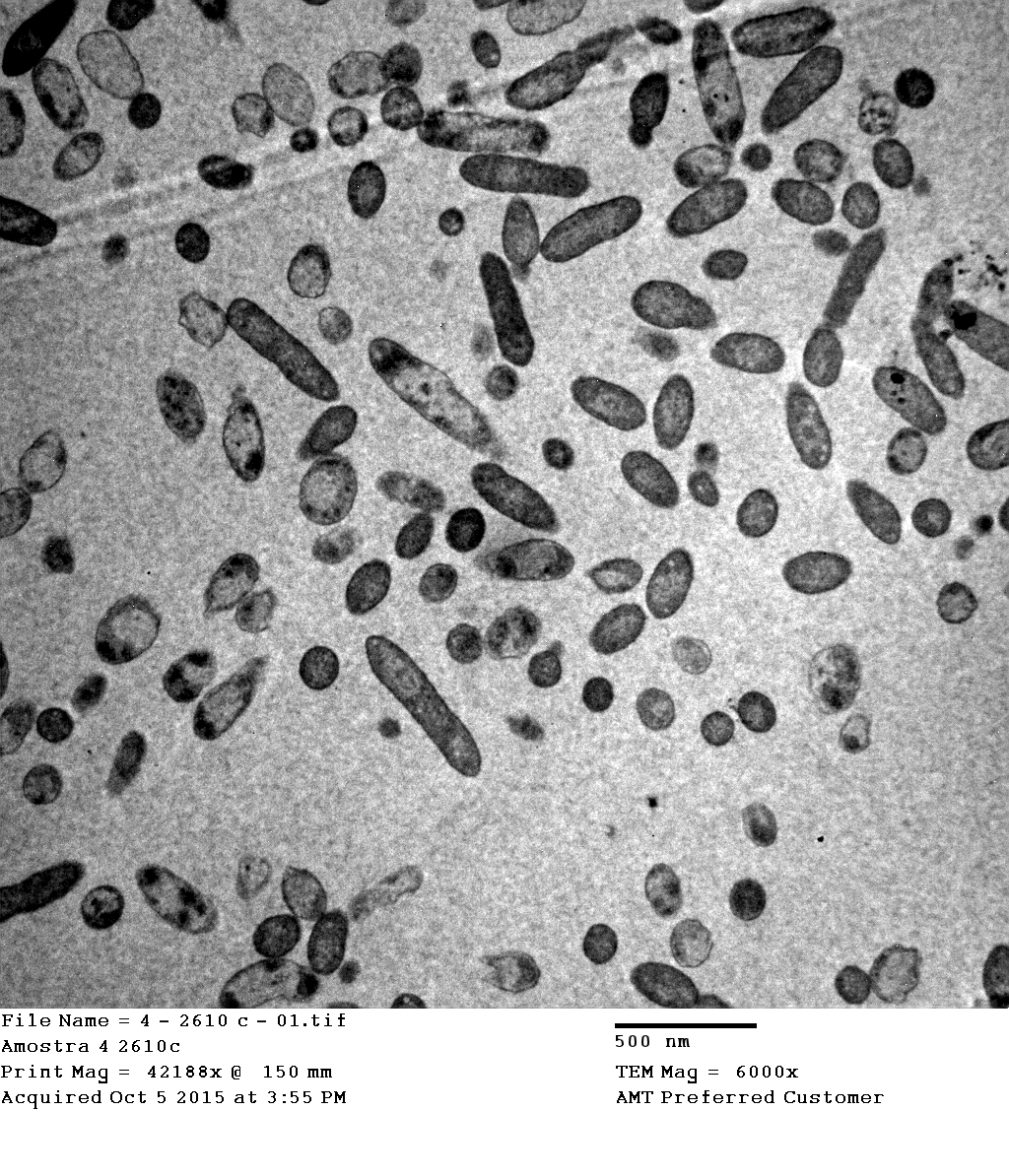

Supplement: Supplementary file 12 — Source Data Fig. 4 [file 44319_2024_60_MOESM12_ESM.zip › Fig 4/4C/raw images/4 - 2610 c - 01.tif]

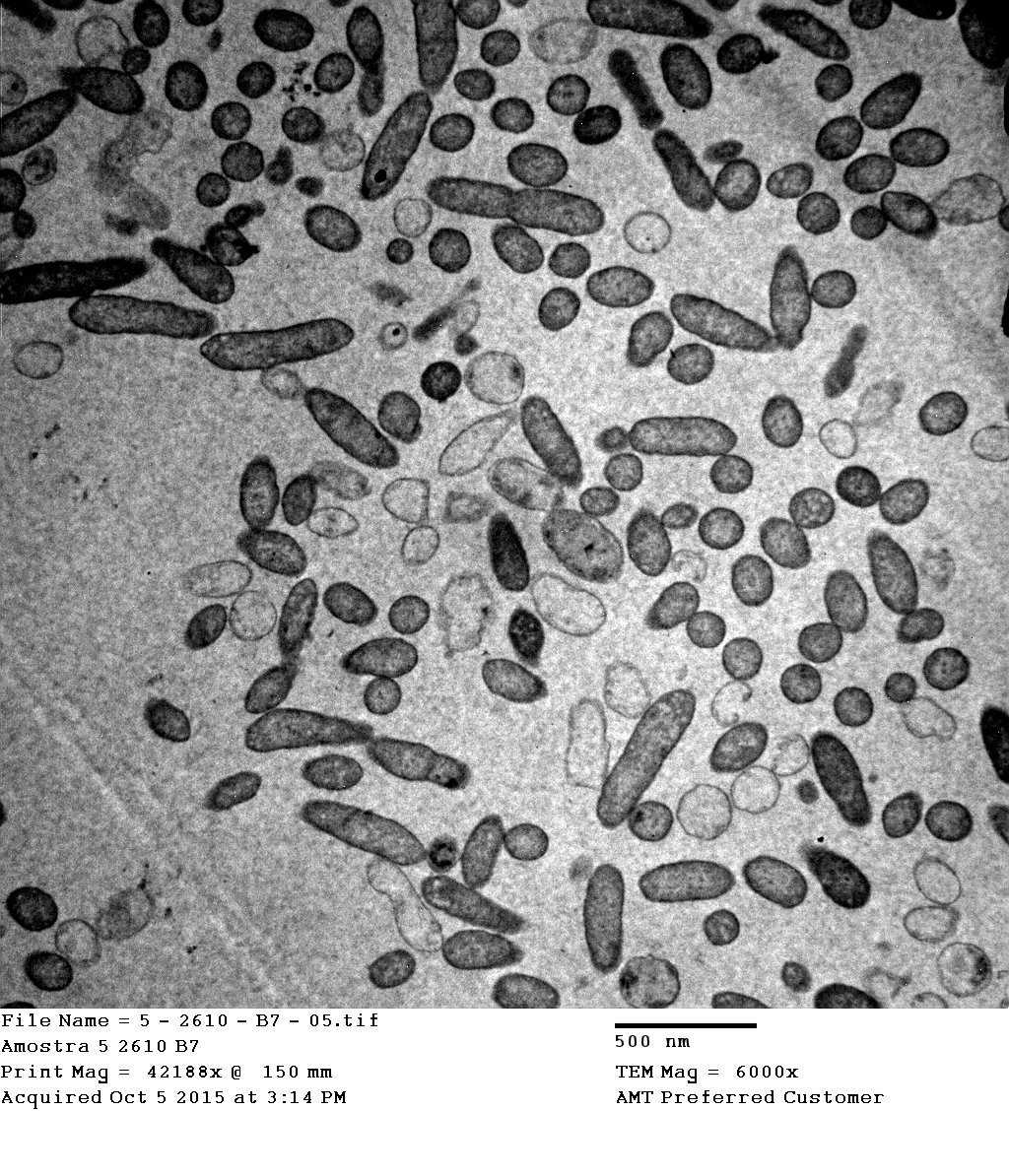

Supplement: Supplementary file 12 — Source Data Fig. 4 [file 44319_2024_60_MOESM12_ESM.zip › Fig 4/4C/raw images/5 - 2610 - B7 - 05.tif]

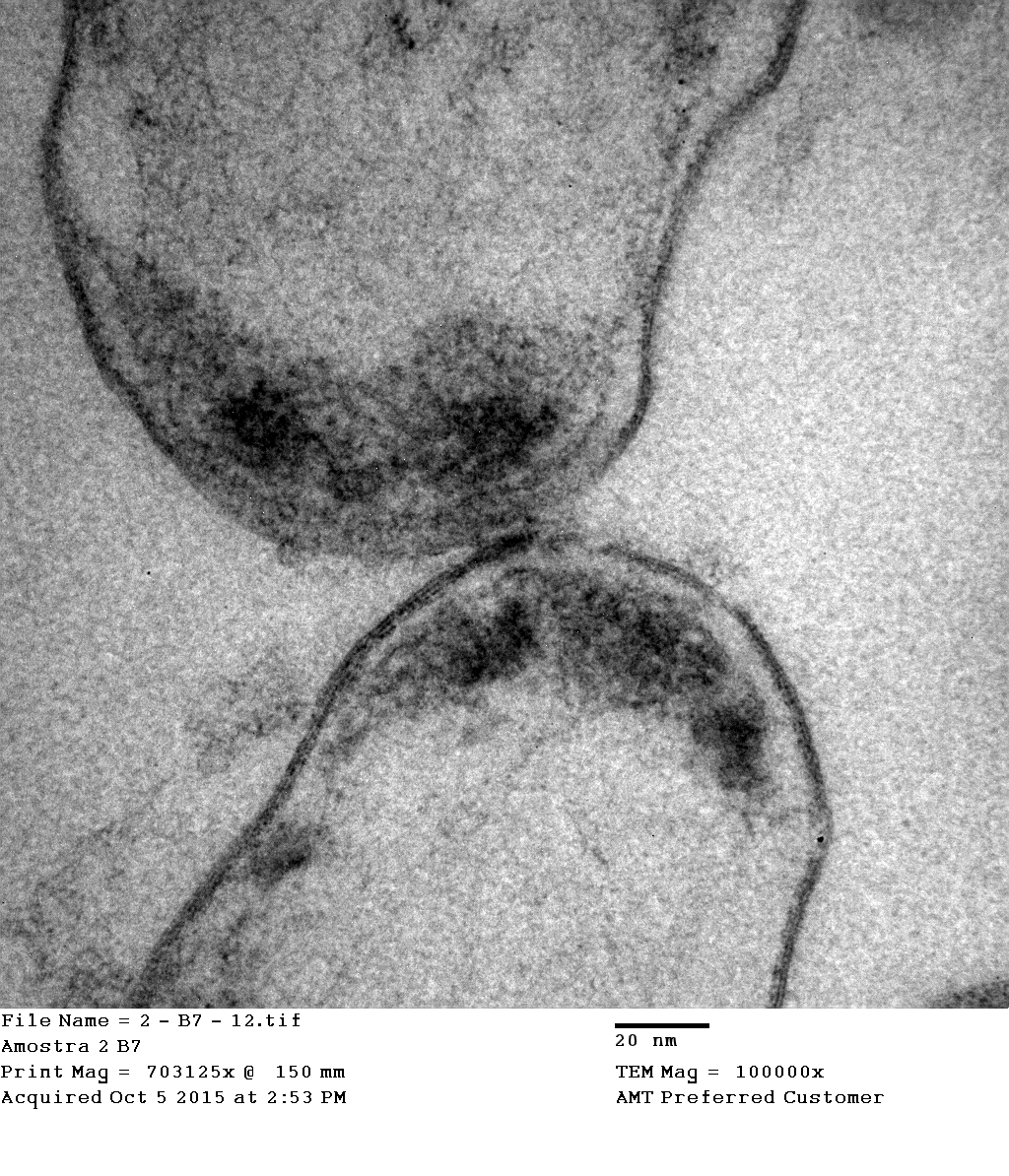

Supplement: Supplementary file 12 — Source Data Fig. 4 [file 44319_2024_60_MOESM12_ESM.zip › Fig 4/4C/raw images/2 - B7 - 12.tif]

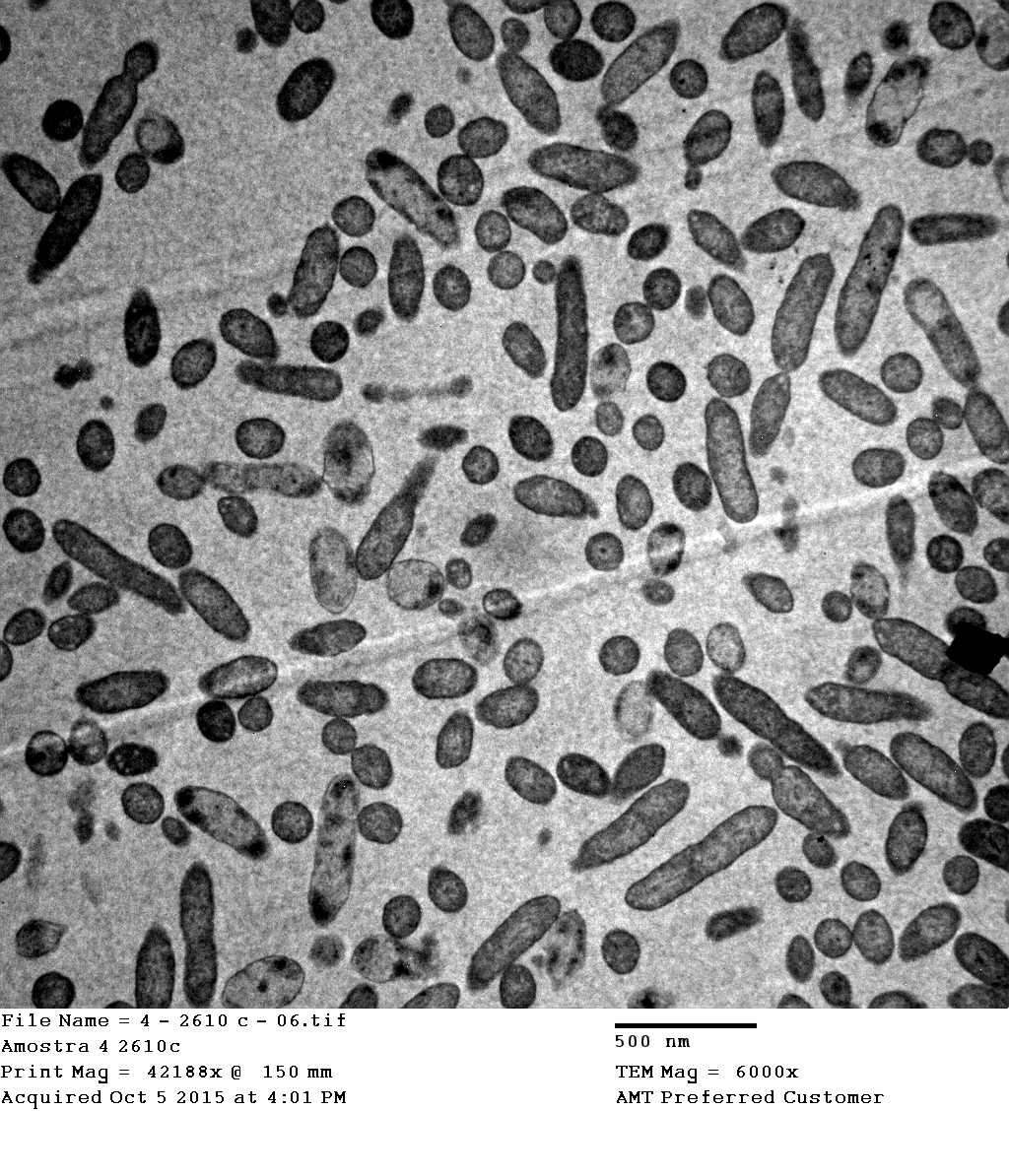

Supplement: Supplementary file 12 — Source Data Fig. 4 [file 44319_2024_60_MOESM12_ESM.zip › Fig 4/4C/raw images/4 - 2610 c - 06.tif]

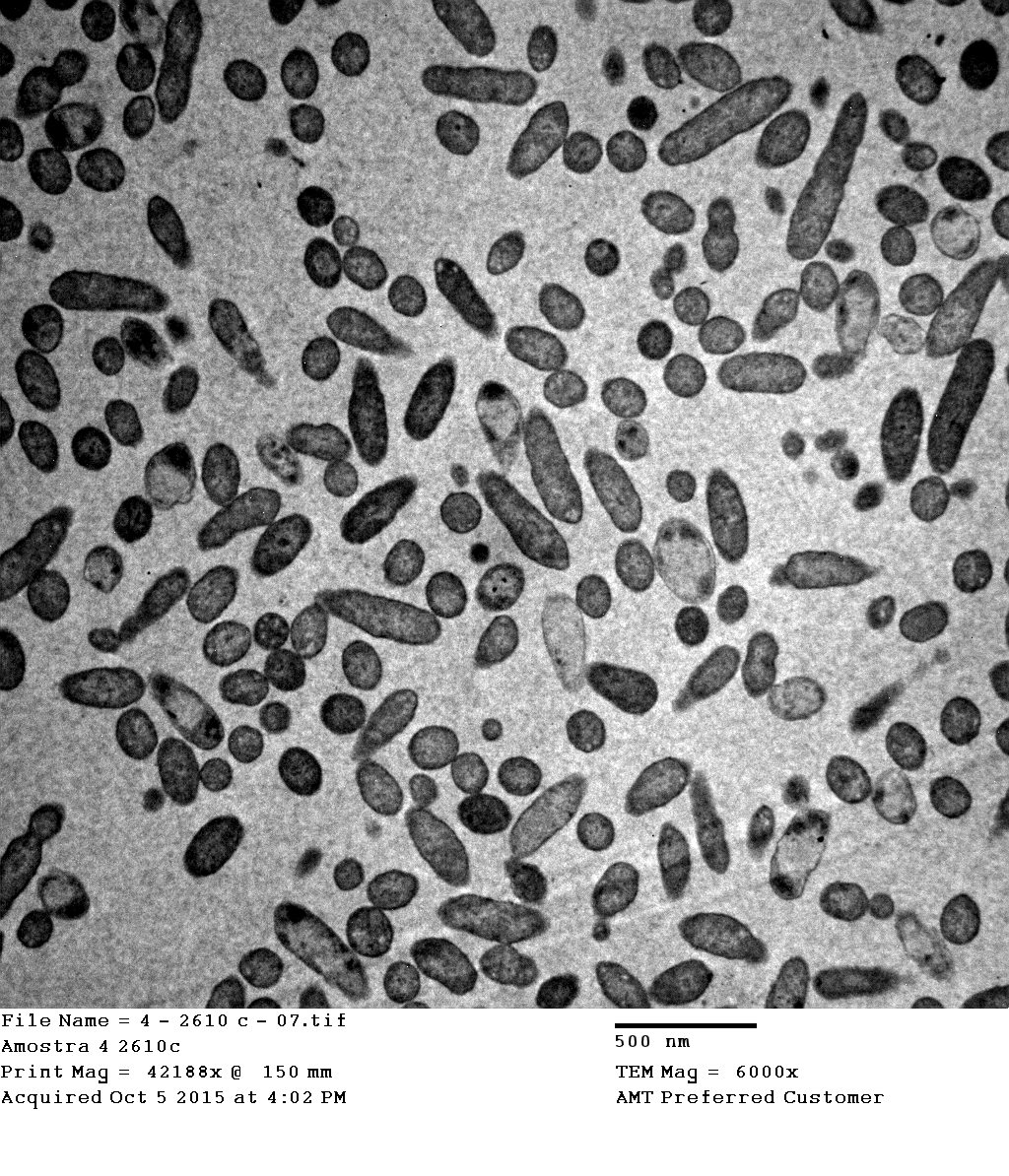

Supplement: Supplementary file 12 — Source Data Fig. 4 [file 44319_2024_60_MOESM12_ESM.zip › Fig 4/4C/raw images/4 - 2610 c - 07.tif]

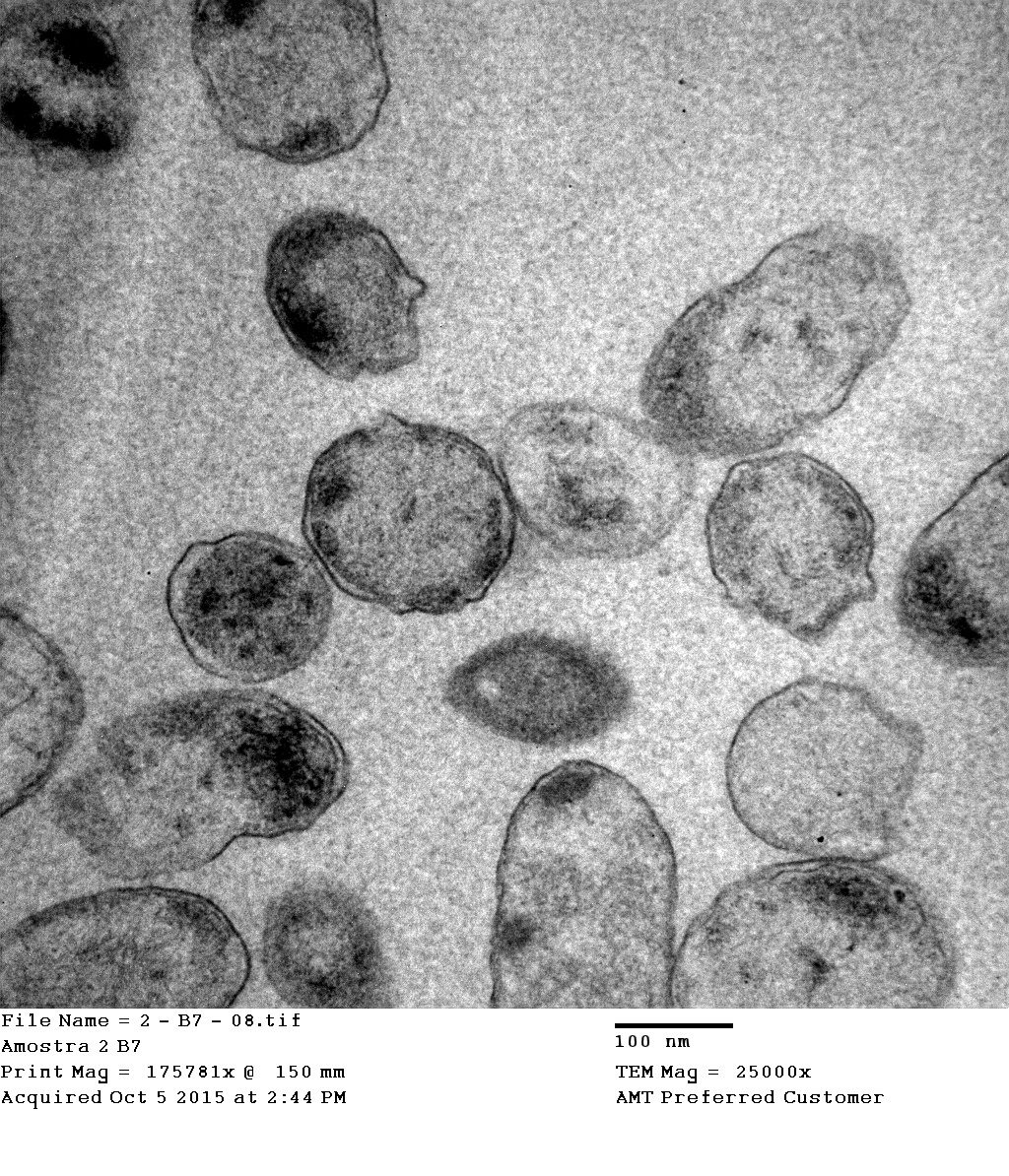

Supplement: Supplementary file 12 — Source Data Fig. 4 [file 44319_2024_60_MOESM12_ESM.zip › Fig 4/4C/raw images/2 - B7 - 08.tif]

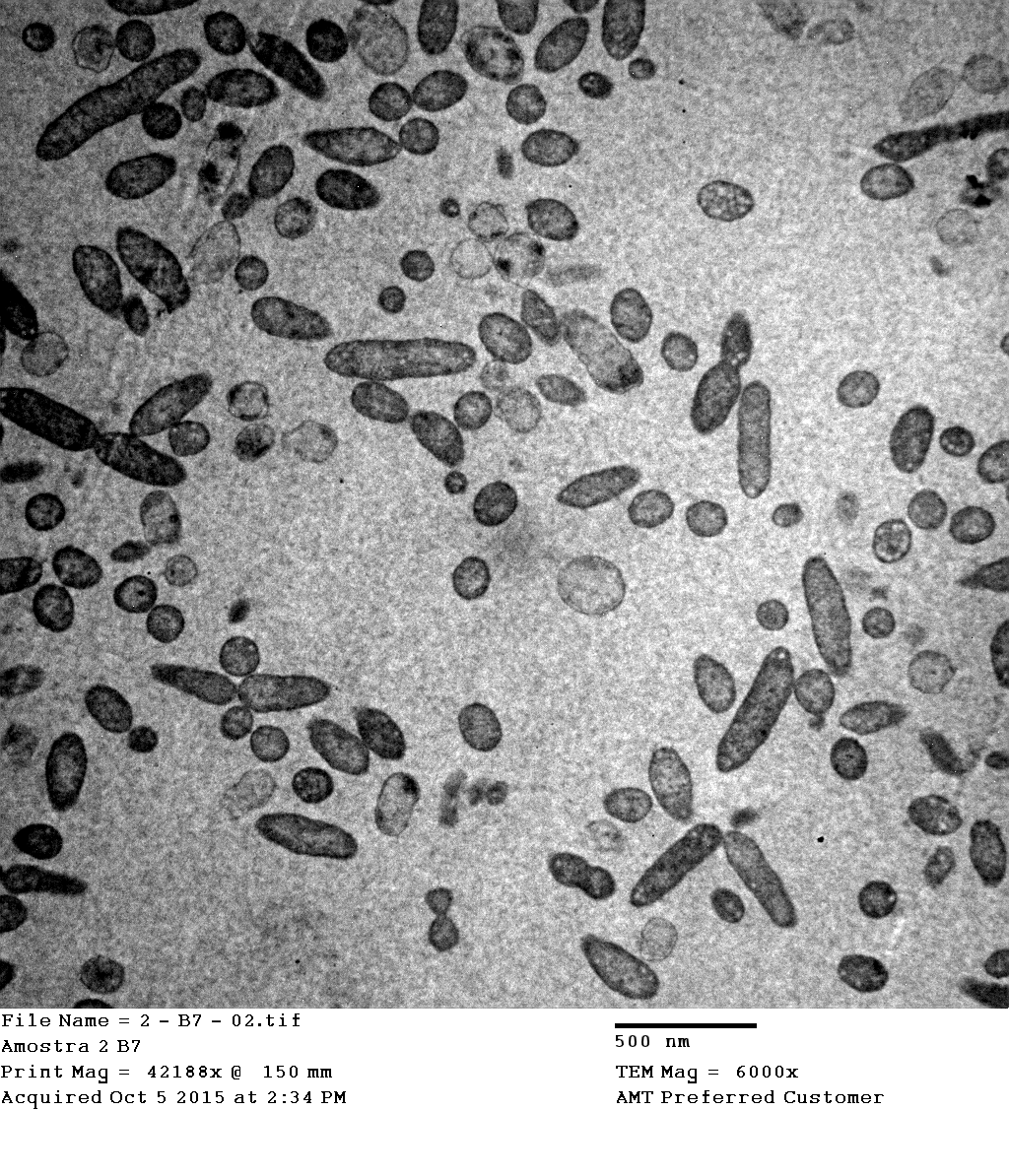

Supplement: Supplementary file 12 — Source Data Fig. 4 [file 44319_2024_60_MOESM12_ESM.zip › Fig 4/4C/raw images/2 - B7 - 02.tif]

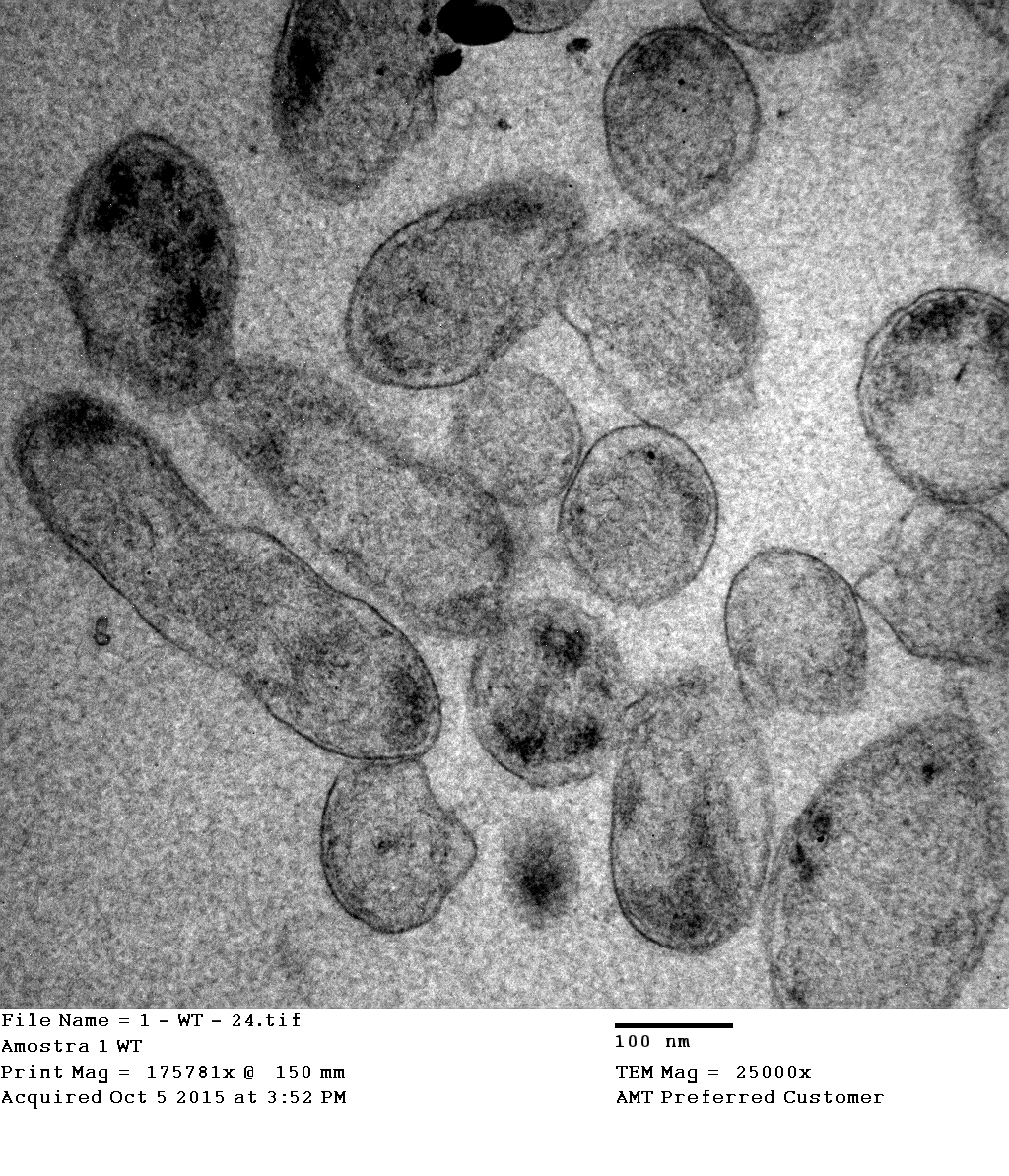

Supplement: Supplementary file 12 — Source Data Fig. 4 [file 44319_2024_60_MOESM12_ESM.zip › Fig 4/4C/raw images/1 - WT - 24.tif]

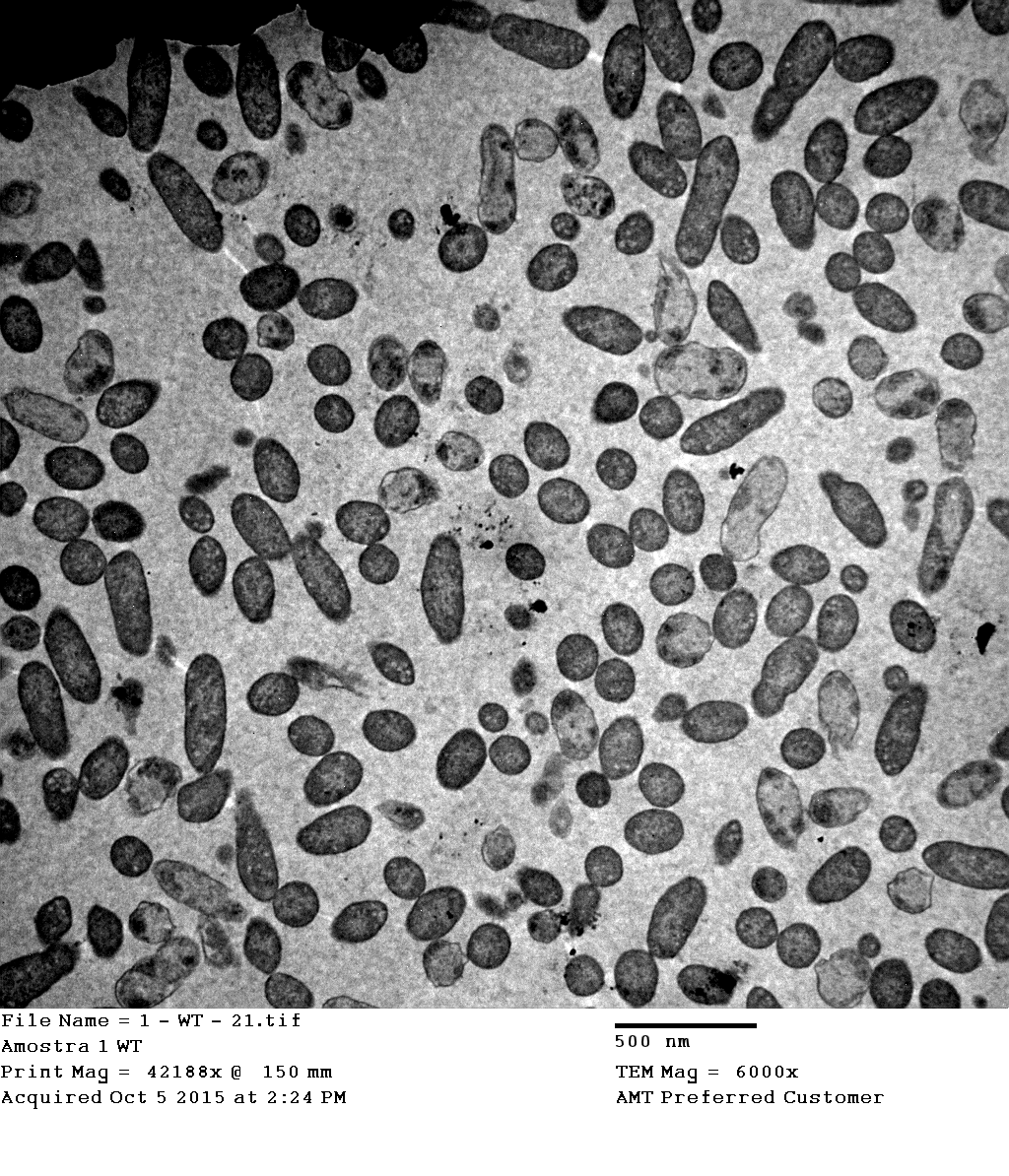

Supplement: Supplementary file 12 — Source Data Fig. 4 [file 44319_2024_60_MOESM12_ESM.zip › Fig 4/4C/raw images/1 - WT - 21.tif]

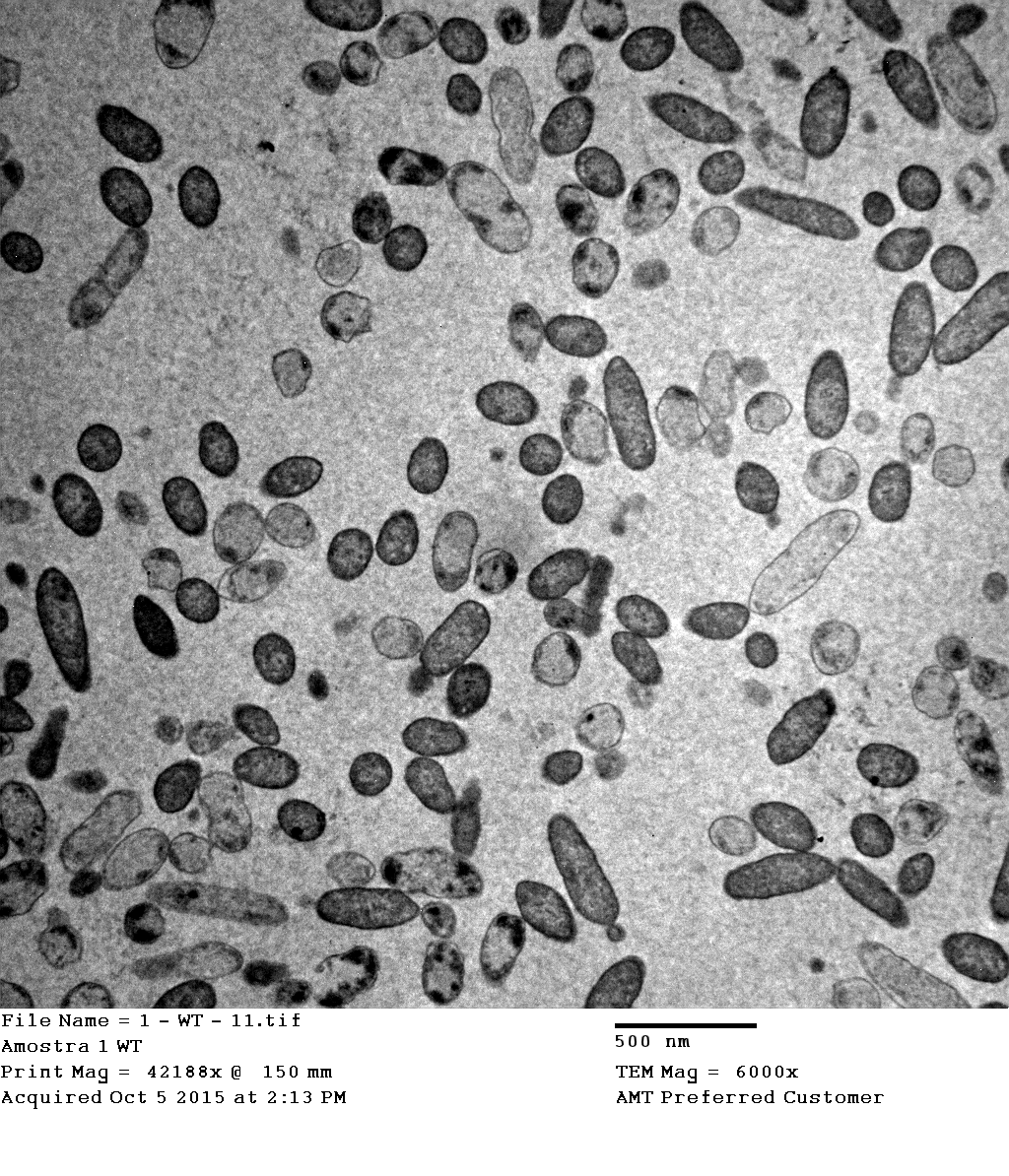

Supplement: Supplementary file 12 — Source Data Fig. 4 [file 44319_2024_60_MOESM12_ESM.zip › Fig 4/4C/raw images/1 - WT - 11.tif]

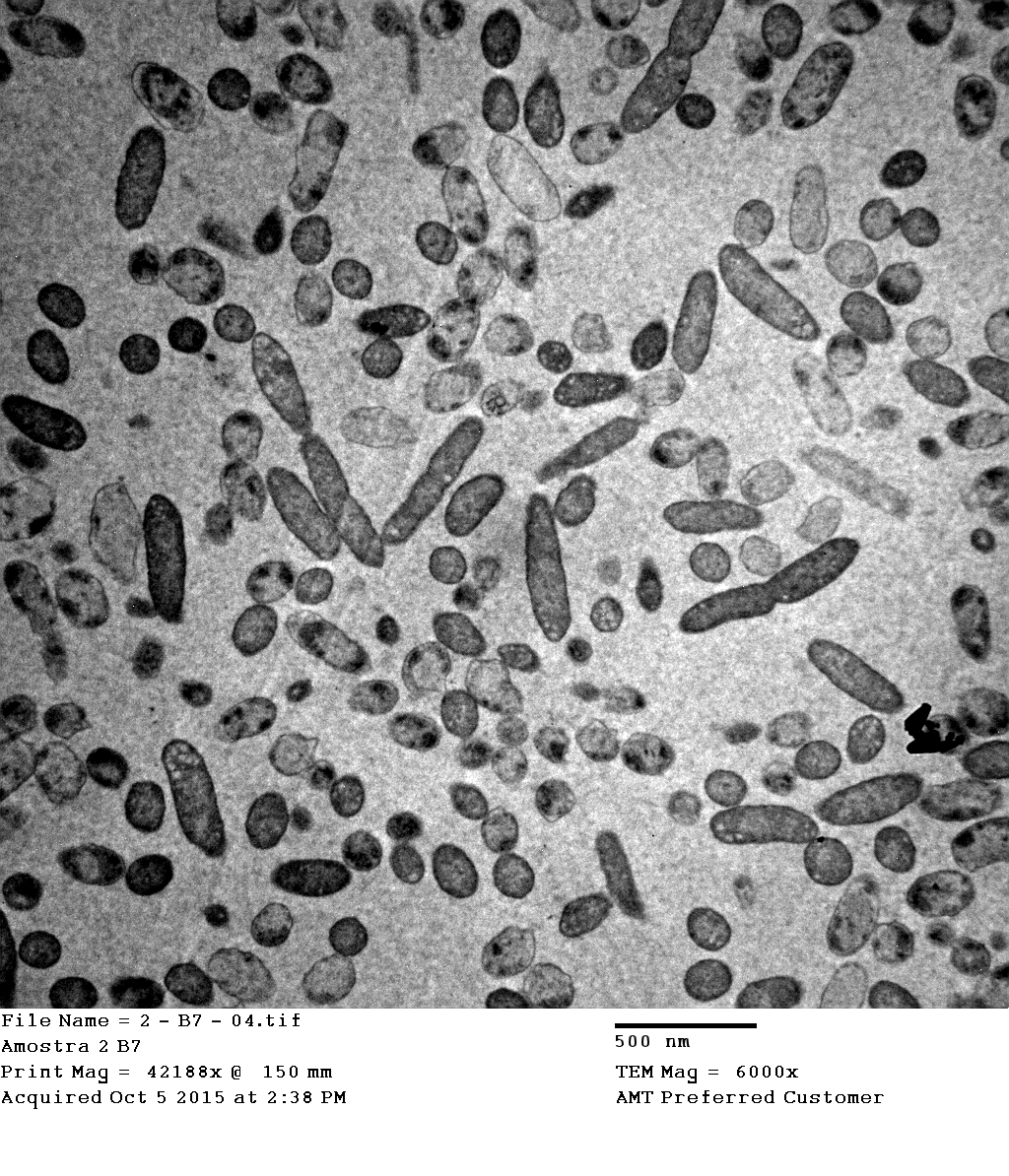

Supplement: Supplementary file 12 — Source Data Fig. 4 [file 44319_2024_60_MOESM12_ESM.zip › Fig 4/4C/raw images/2 - B7 - 04.tif]

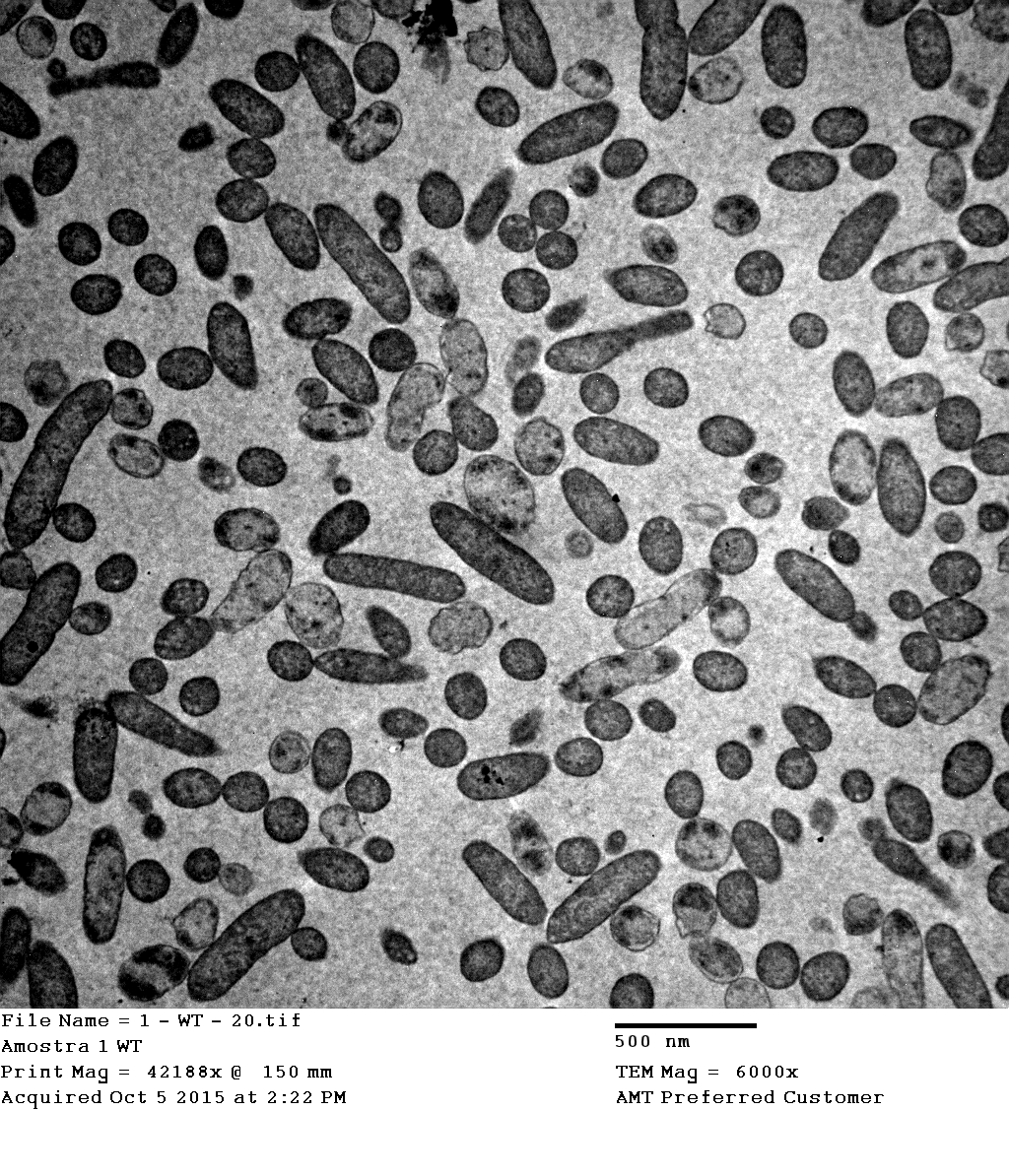

Supplement: Supplementary file 12 — Source Data Fig. 4 [file 44319_2024_60_MOESM12_ESM.zip › Fig 4/4C/raw images/1 - WT - 20.tif]

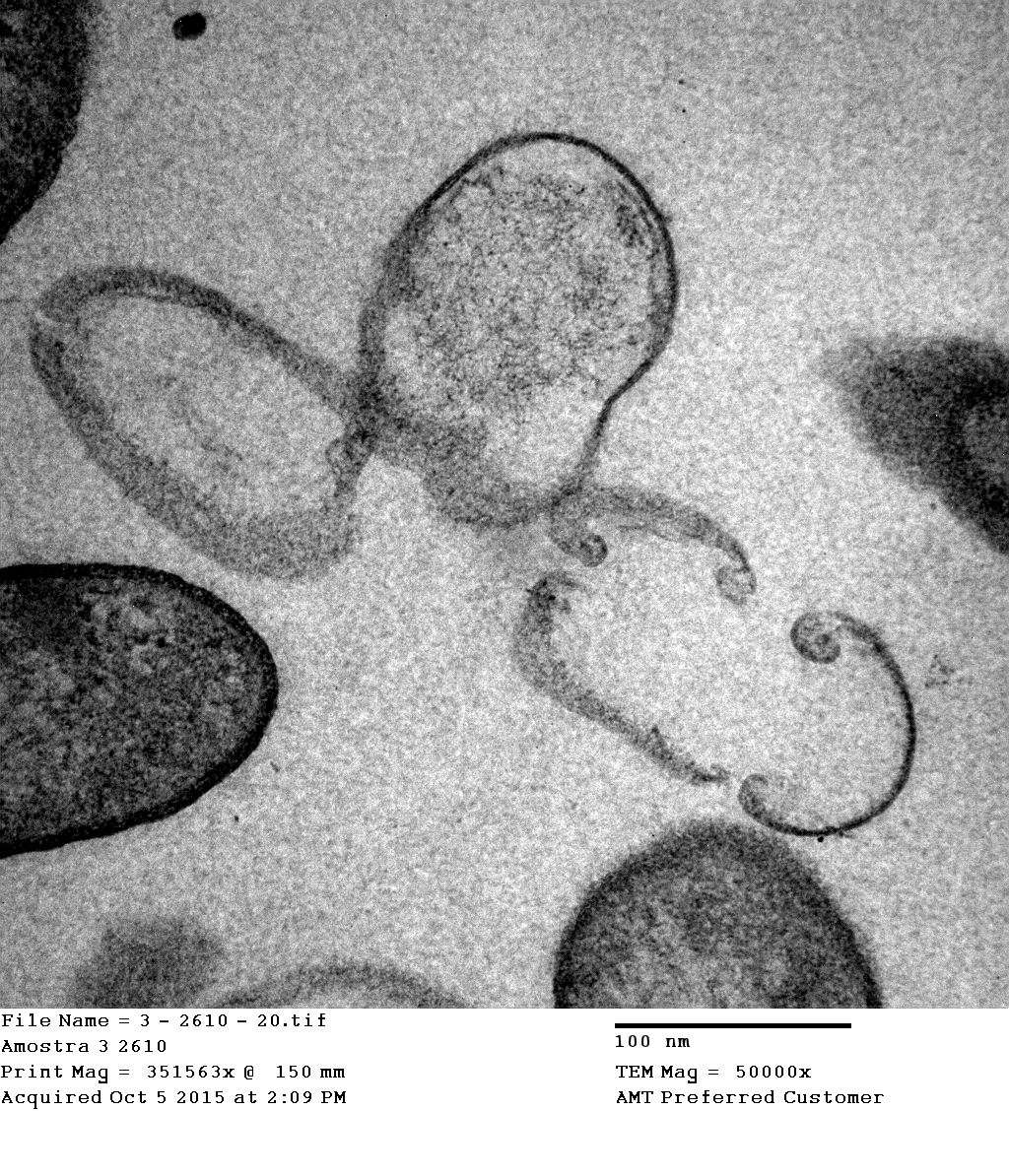

Supplement: Supplementary file 12 — Source Data Fig. 4 [file 44319_2024_60_MOESM12_ESM.zip › Fig 4/4C/raw images/3 - 2610 - 20.tif]

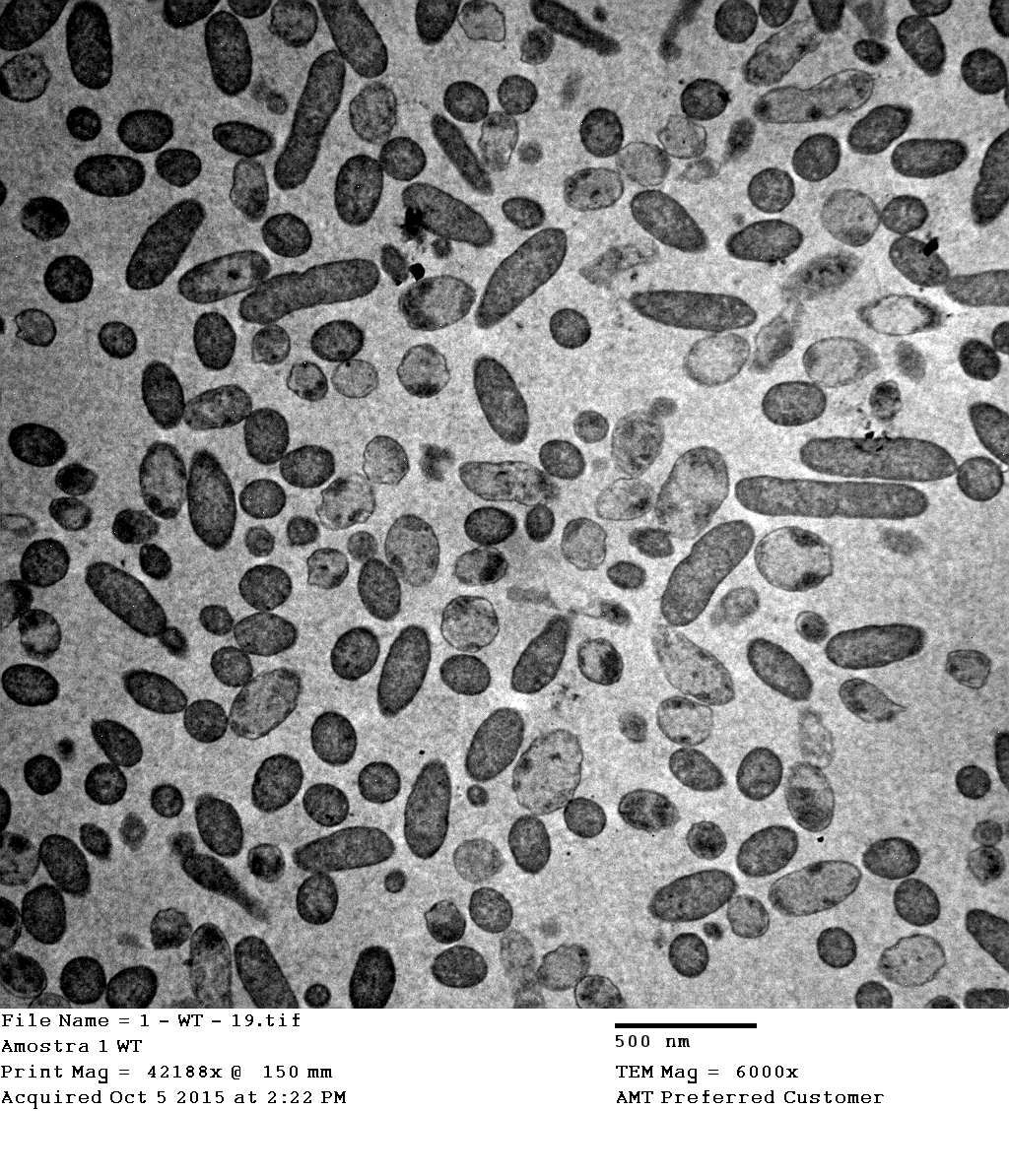

Supplement: Supplementary file 12 — Source Data Fig. 4 [file 44319_2024_60_MOESM12_ESM.zip › Fig 4/4C/raw images/1 - WT - 19.tif]

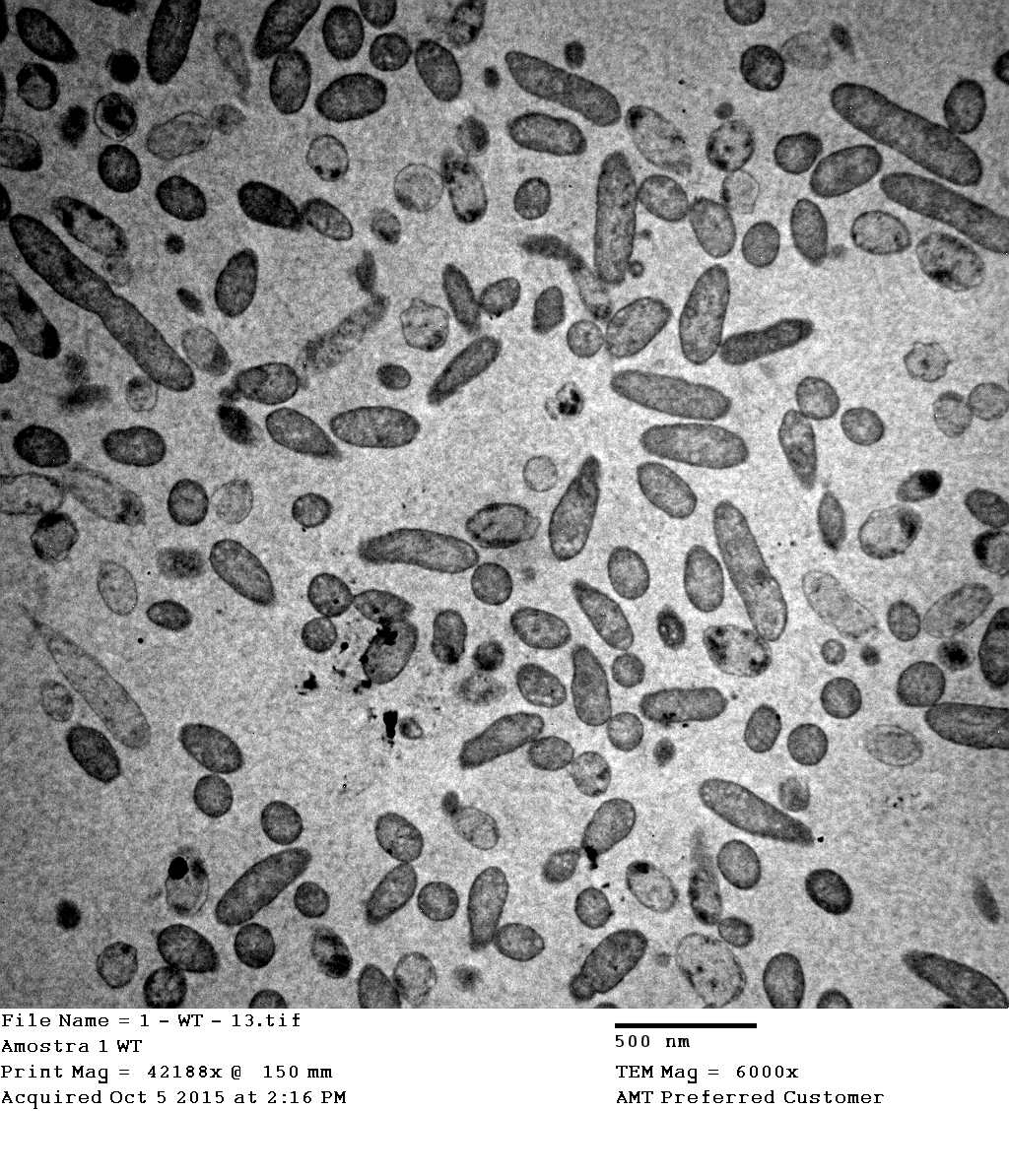

Supplement: Supplementary file 12 — Source Data Fig. 4 [file 44319_2024_60_MOESM12_ESM.zip › Fig 4/4C/raw images/1 - WT - 13.tif]

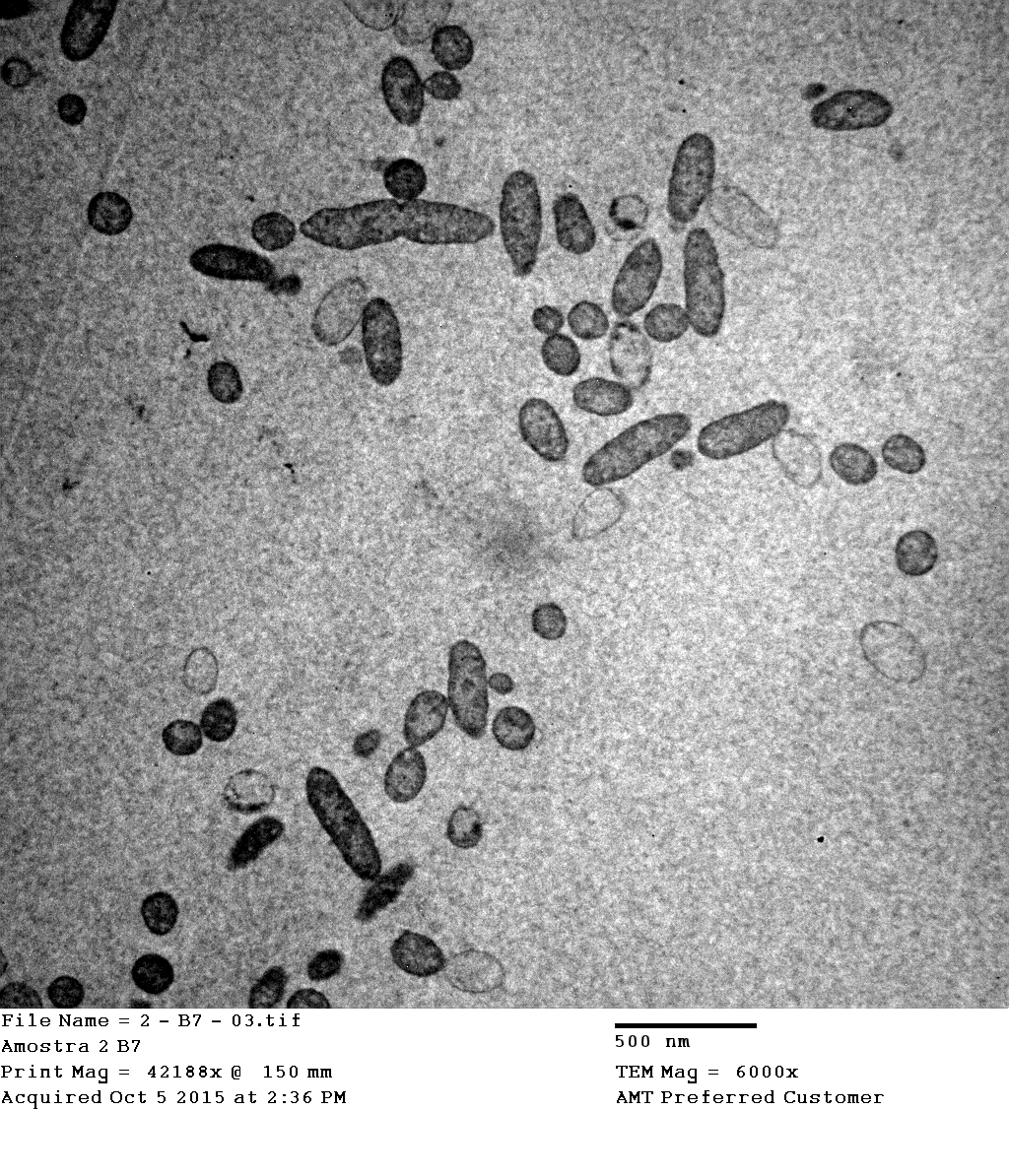

Supplement: Supplementary file 12 — Source Data Fig. 4 [file 44319_2024_60_MOESM12_ESM.zip › Fig 4/4C/raw images/2 - B7 - 03.tif]

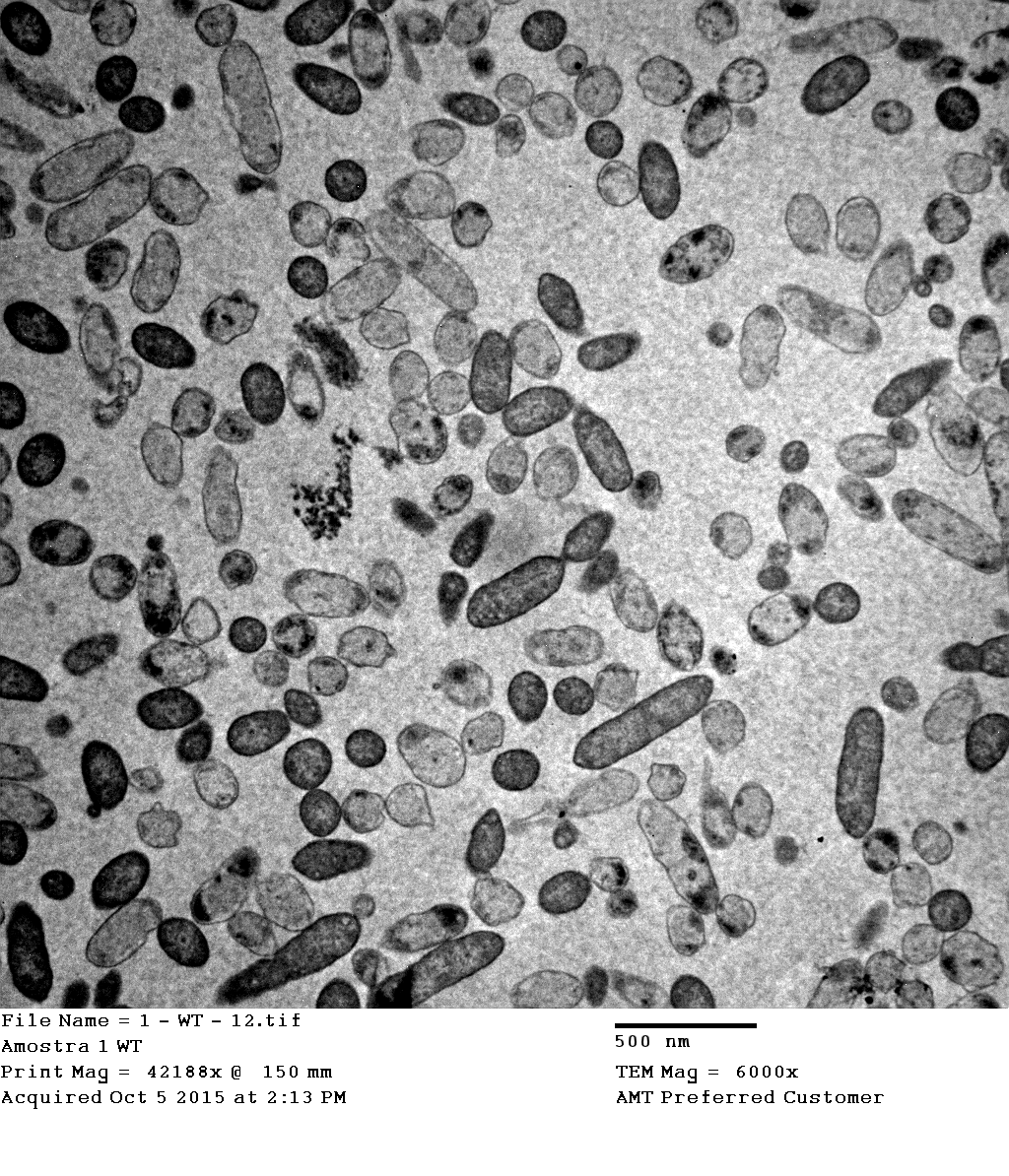

Supplement: Supplementary file 12 — Source Data Fig. 4 [file 44319_2024_60_MOESM12_ESM.zip › Fig 4/4C/raw images/1 - WT - 12.tif]

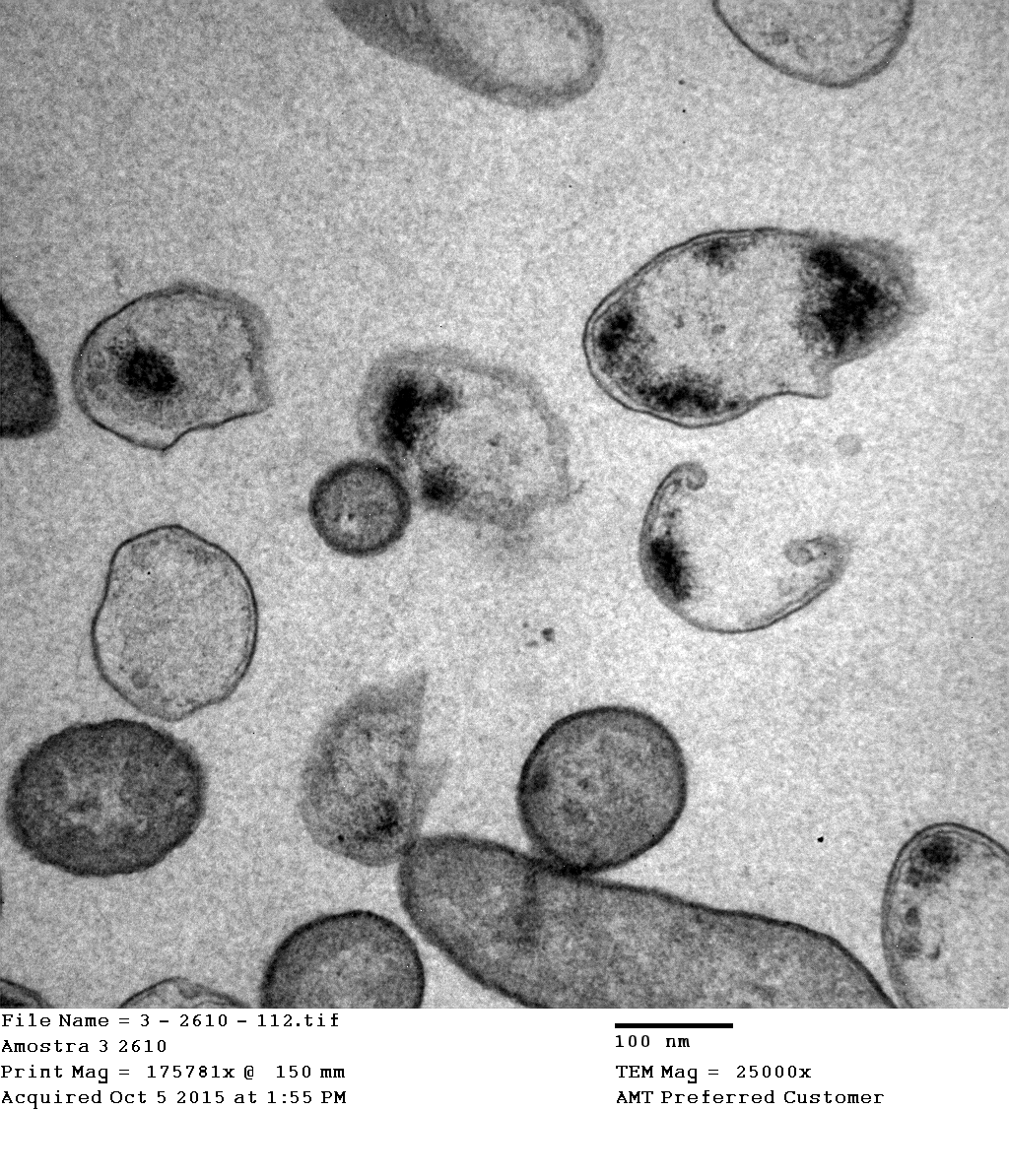

Supplement: Supplementary file 12 — Source Data Fig. 4 [file 44319_2024_60_MOESM12_ESM.zip › Fig 4/4C/raw images/3 - 2610 - 12.tif]

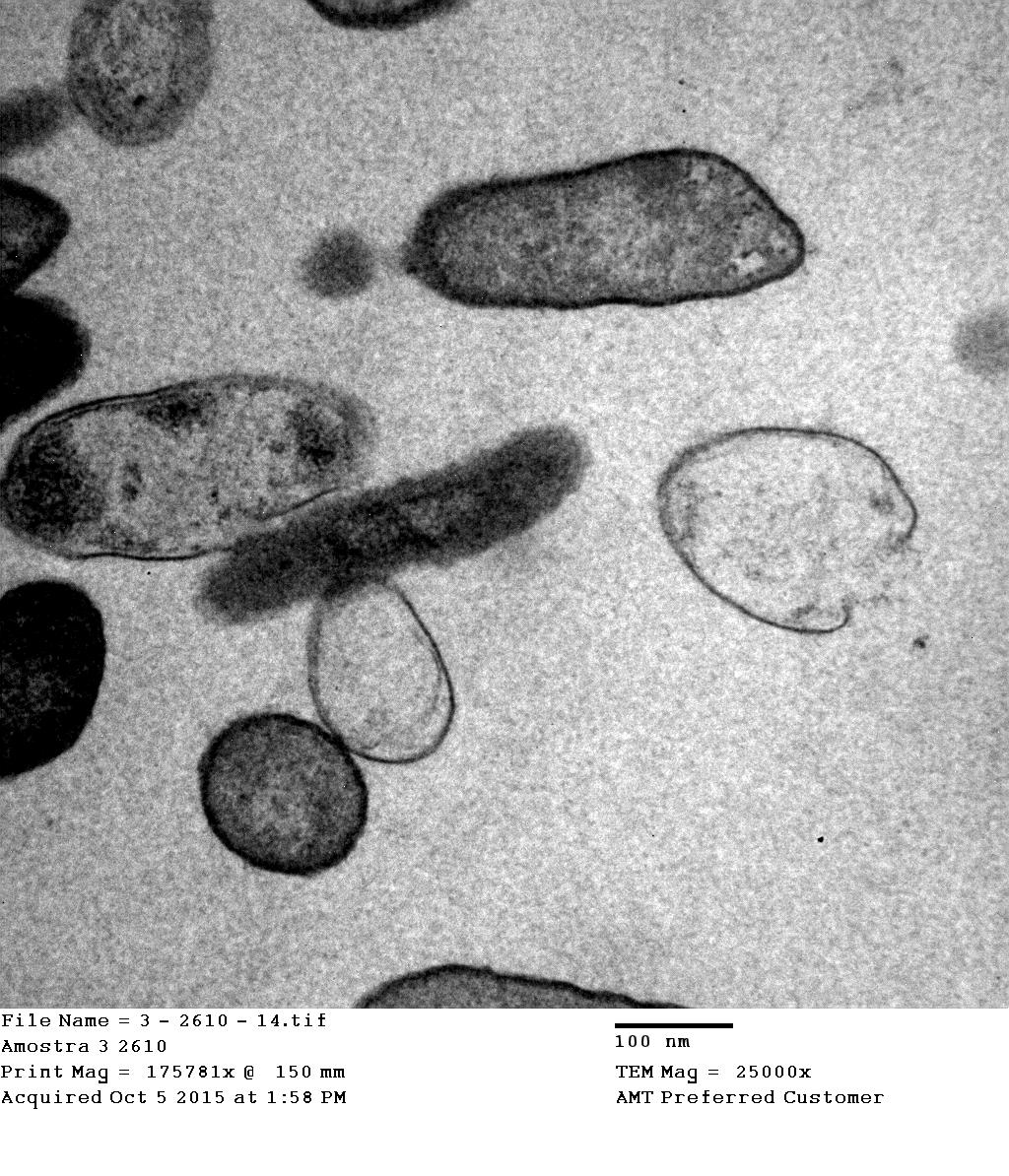

Supplement: Supplementary file 12 — Source Data Fig. 4 [file 44319_2024_60_MOESM12_ESM.zip › Fig 4/4C/raw images/3 - 2610 - 14.tif]

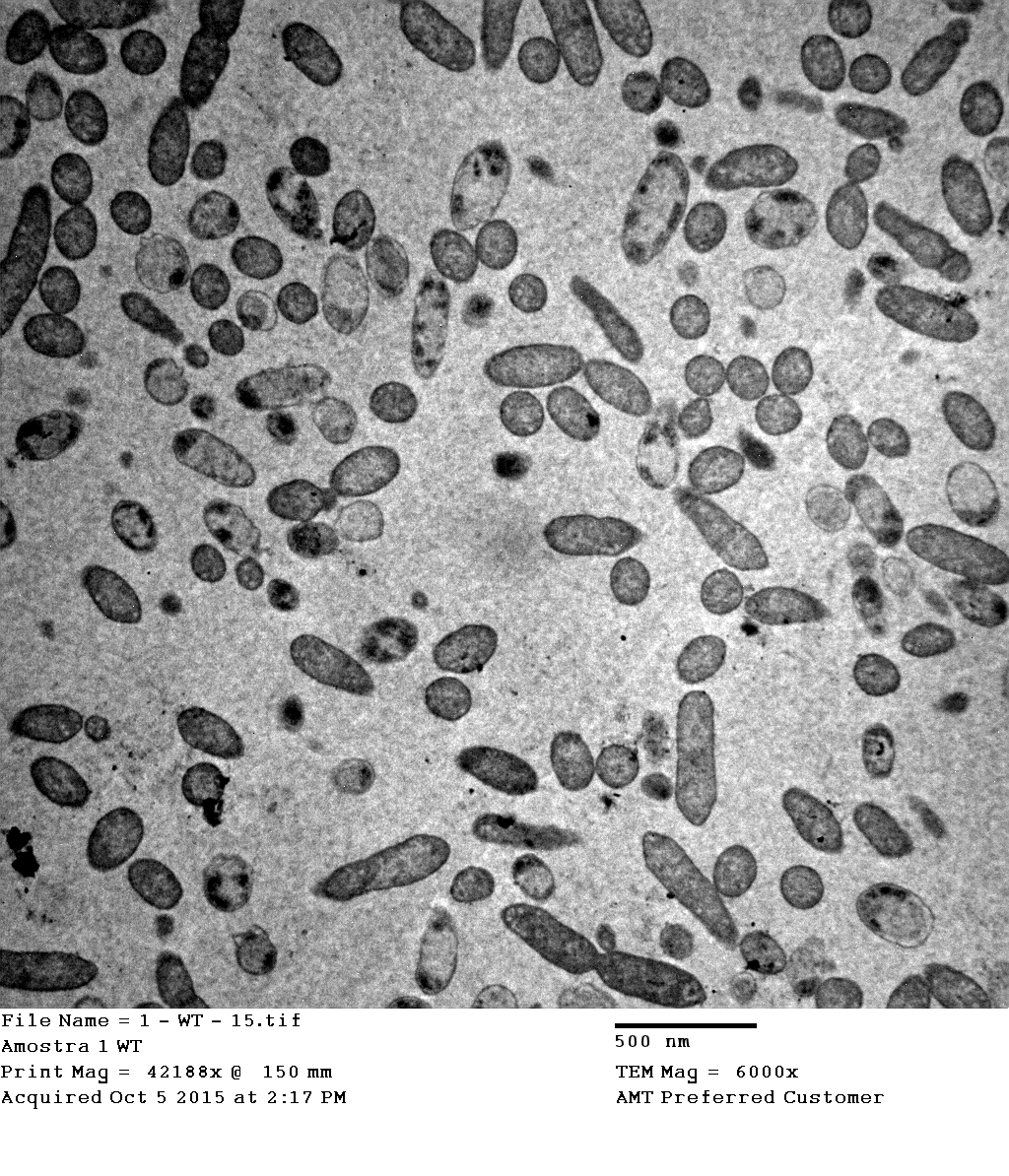

Supplement: Supplementary file 12 — Source Data Fig. 4 [file 44319_2024_60_MOESM12_ESM.zip › Fig 4/4C/raw images/1 - WT - 15.tif]

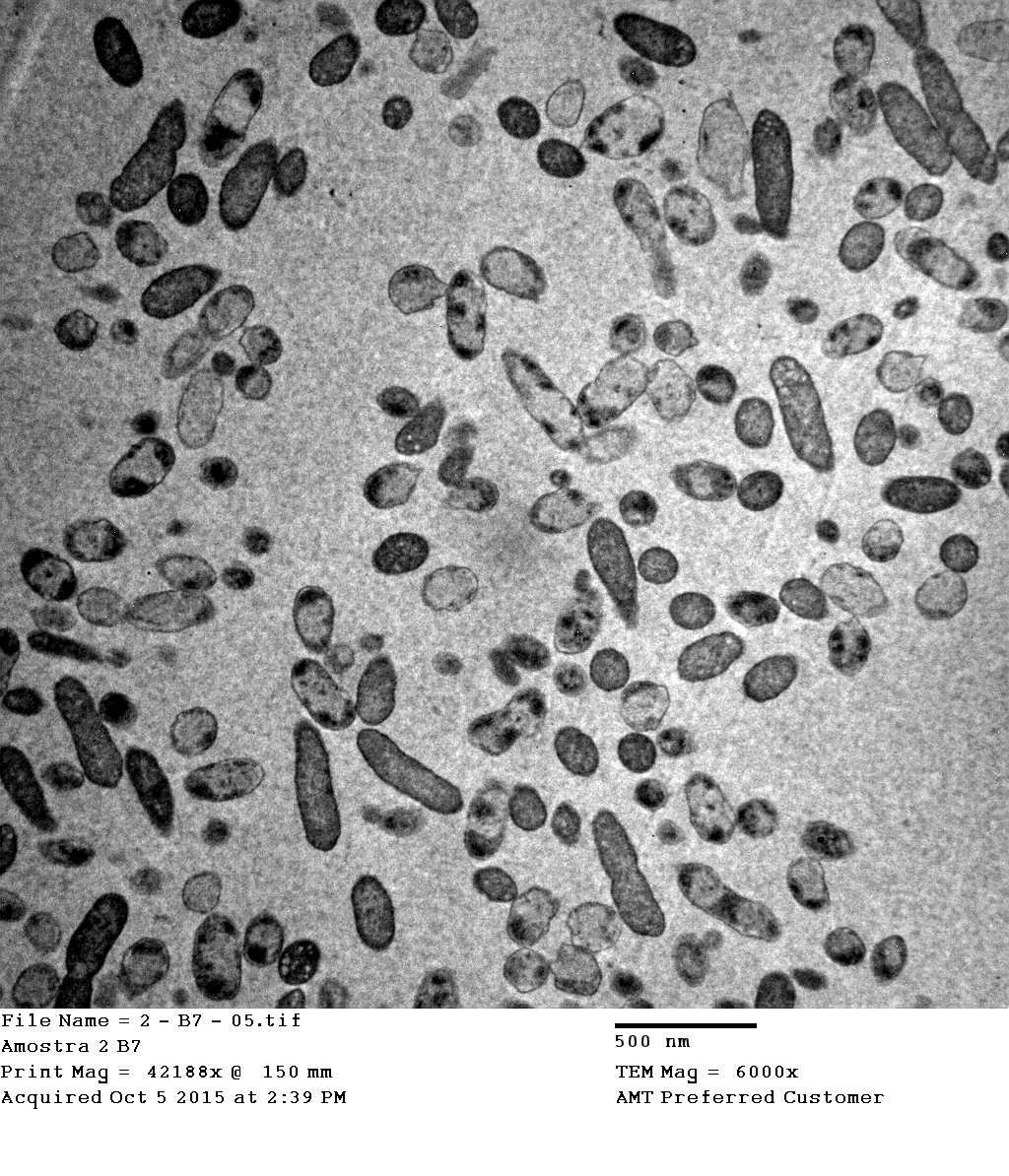

Supplement: Supplementary file 12 — Source Data Fig. 4 [file 44319_2024_60_MOESM12_ESM.zip › Fig 4/4C/raw images/2 - B7 - 05.tif]

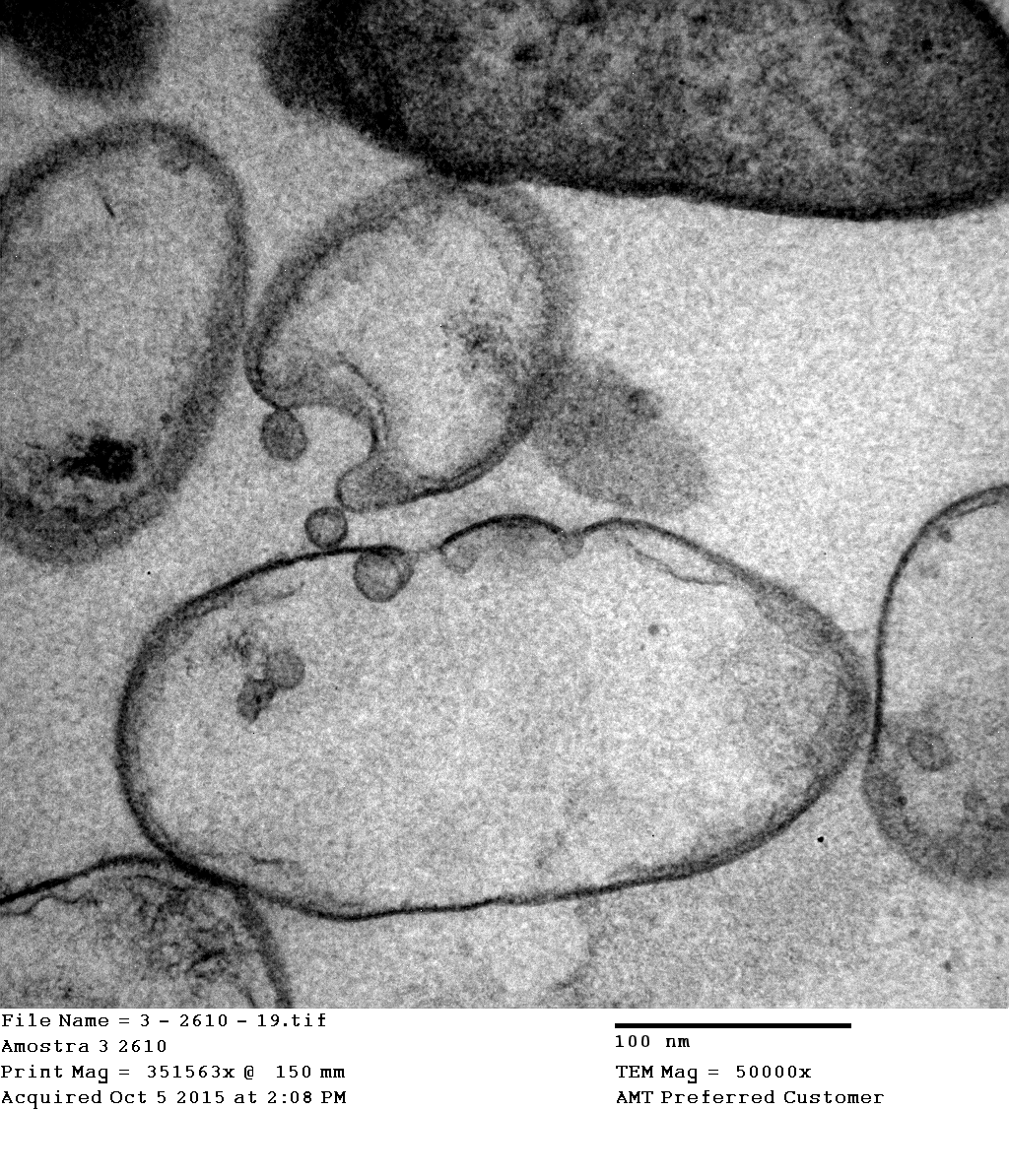

Supplement: Supplementary file 12 — Source Data Fig. 4 [file 44319_2024_60_MOESM12_ESM.zip › Fig 4/4C/raw images/3 - 2610 - 19.tif]

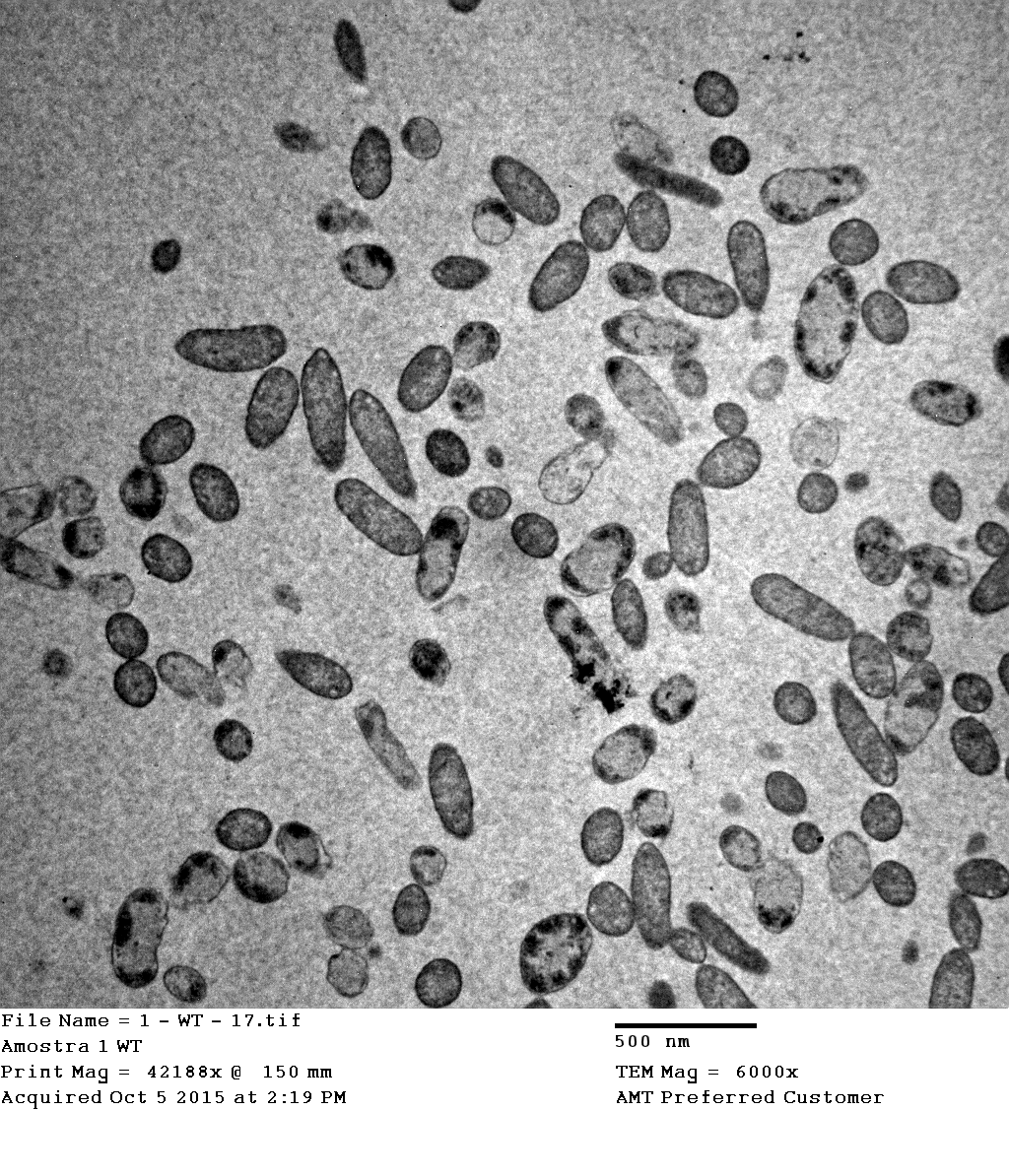

Supplement: Supplementary file 12 — Source Data Fig. 4 [file 44319_2024_60_MOESM12_ESM.zip › Fig 4/4C/raw images/1 - WT - 17.tif]

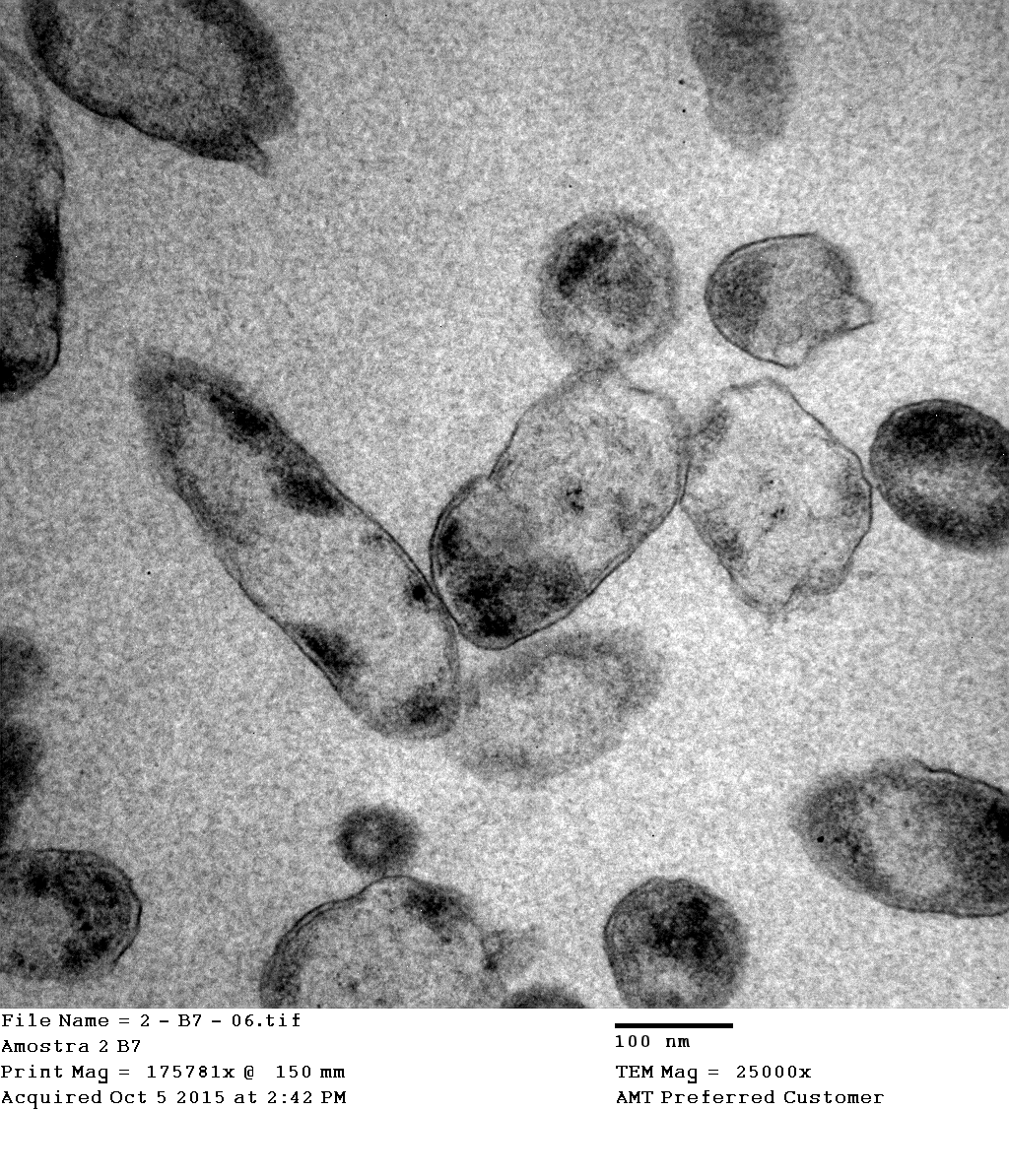

Supplement: Supplementary file 12 — Source Data Fig. 4 [file 44319_2024_60_MOESM12_ESM.zip › Fig 4/4C/raw images/2 - B7 - 06.tif]

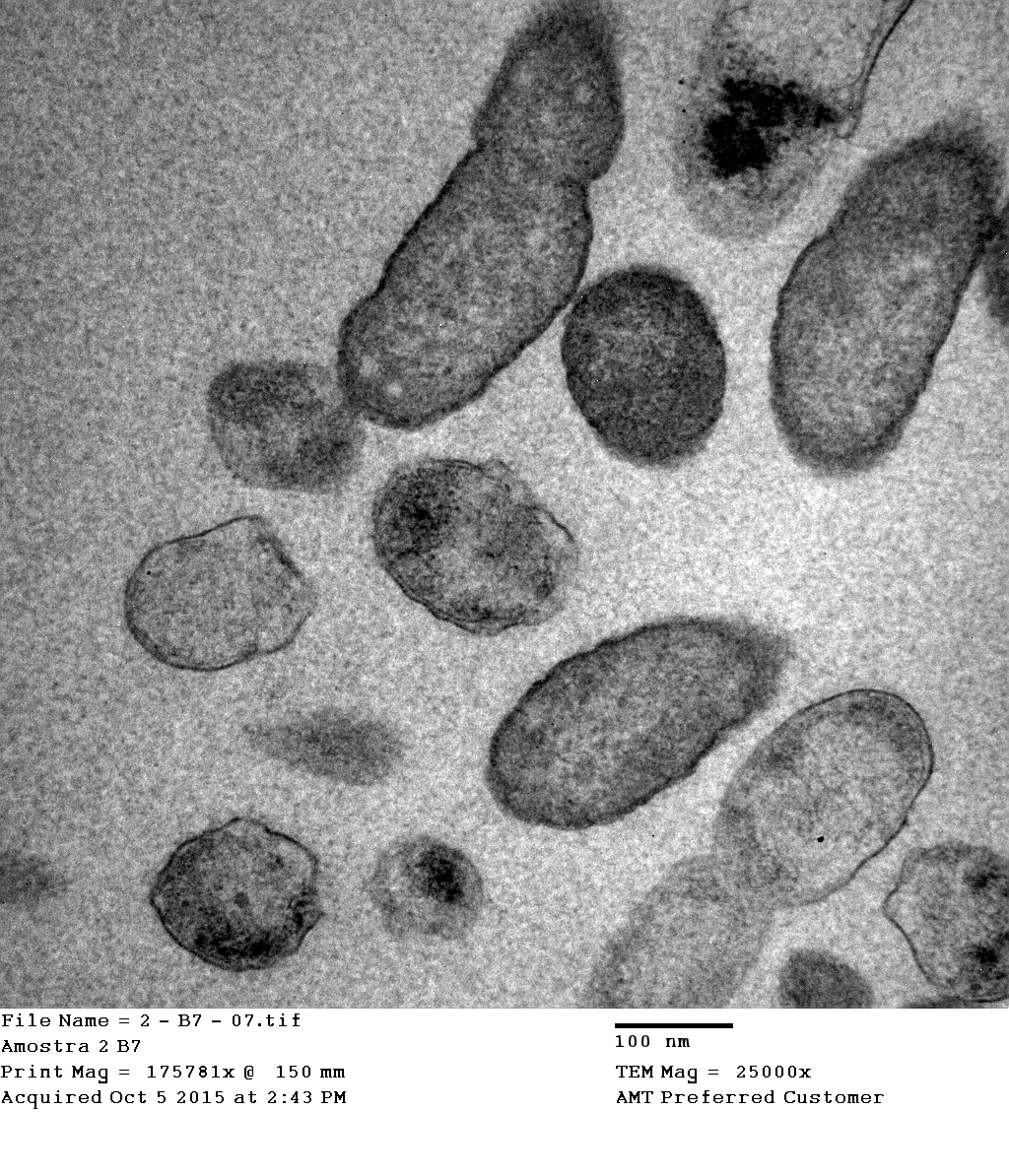

Supplement: Supplementary file 12 — Source Data Fig. 4 [file 44319_2024_60_MOESM12_ESM.zip › Fig 4/4C/raw images/2 - B7 - 07.tif]

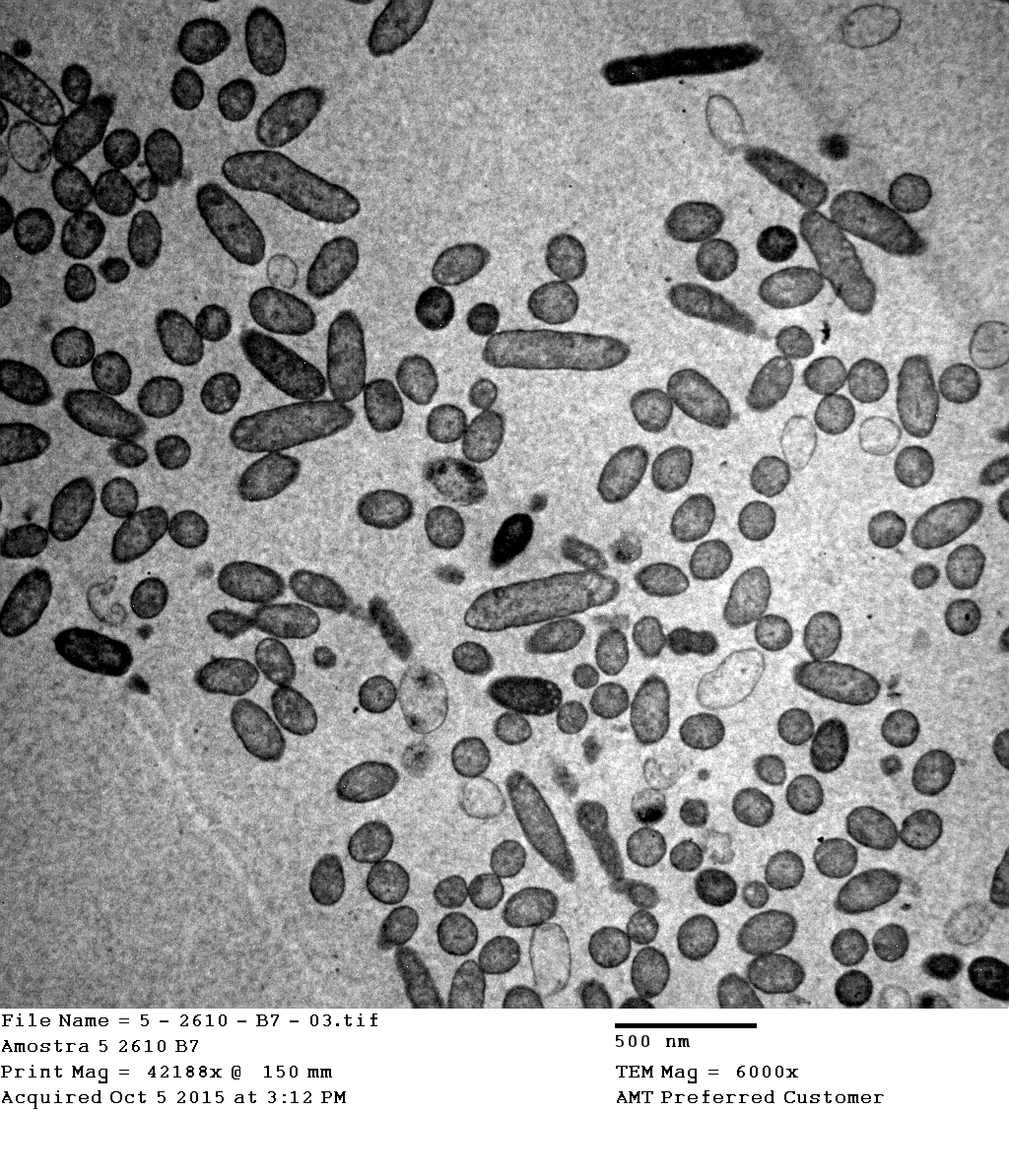

Supplement: Supplementary file 12 — Source Data Fig. 4 [file 44319_2024_60_MOESM12_ESM.zip › Fig 4/4C/raw images/5 - 2610 - B7 - 03.tif]

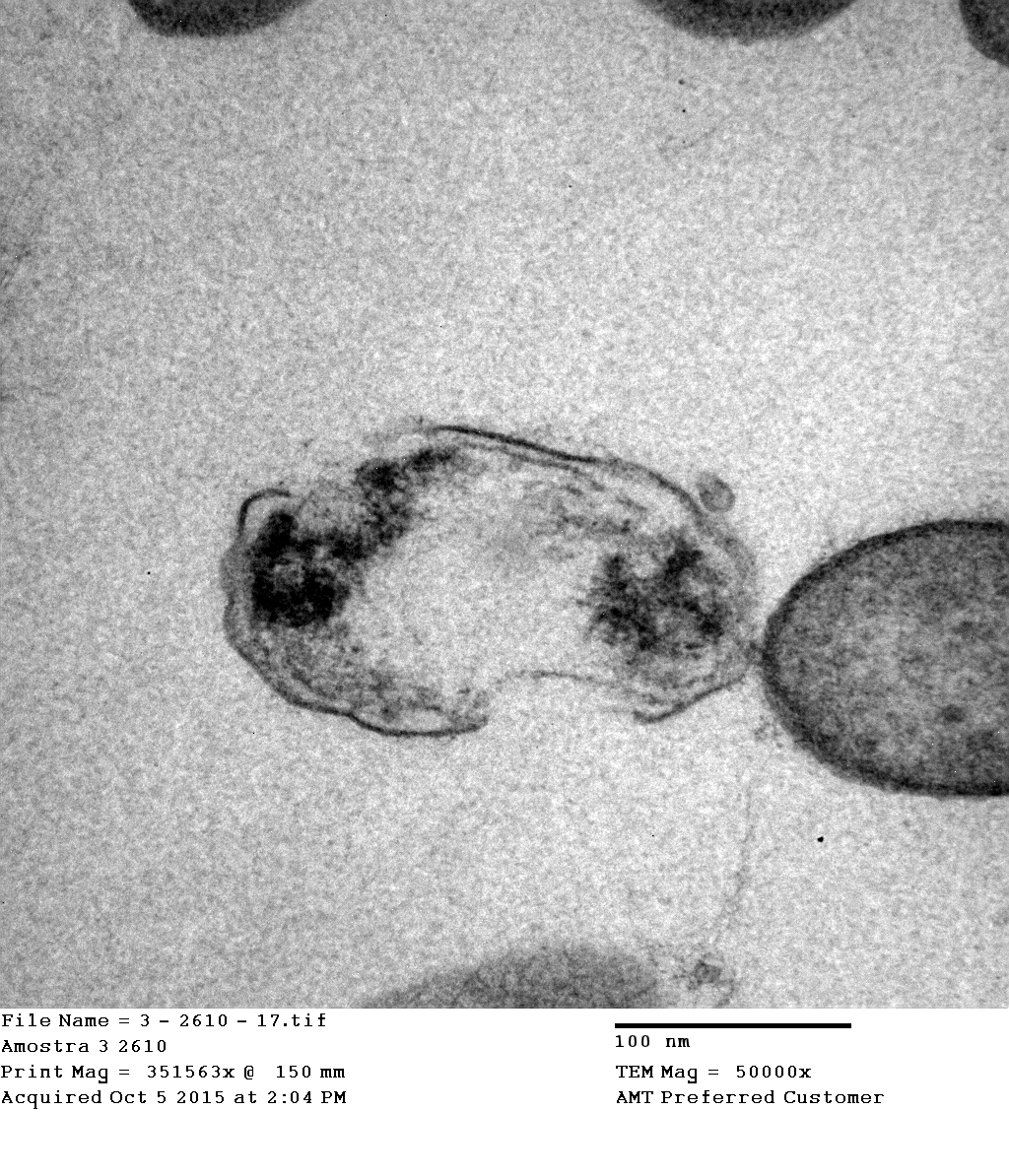

Supplement: Supplementary file 12 — Source Data Fig. 4 [file 44319_2024_60_MOESM12_ESM.zip › Fig 4/4C/raw images/3 - 2610 - 17.tif]

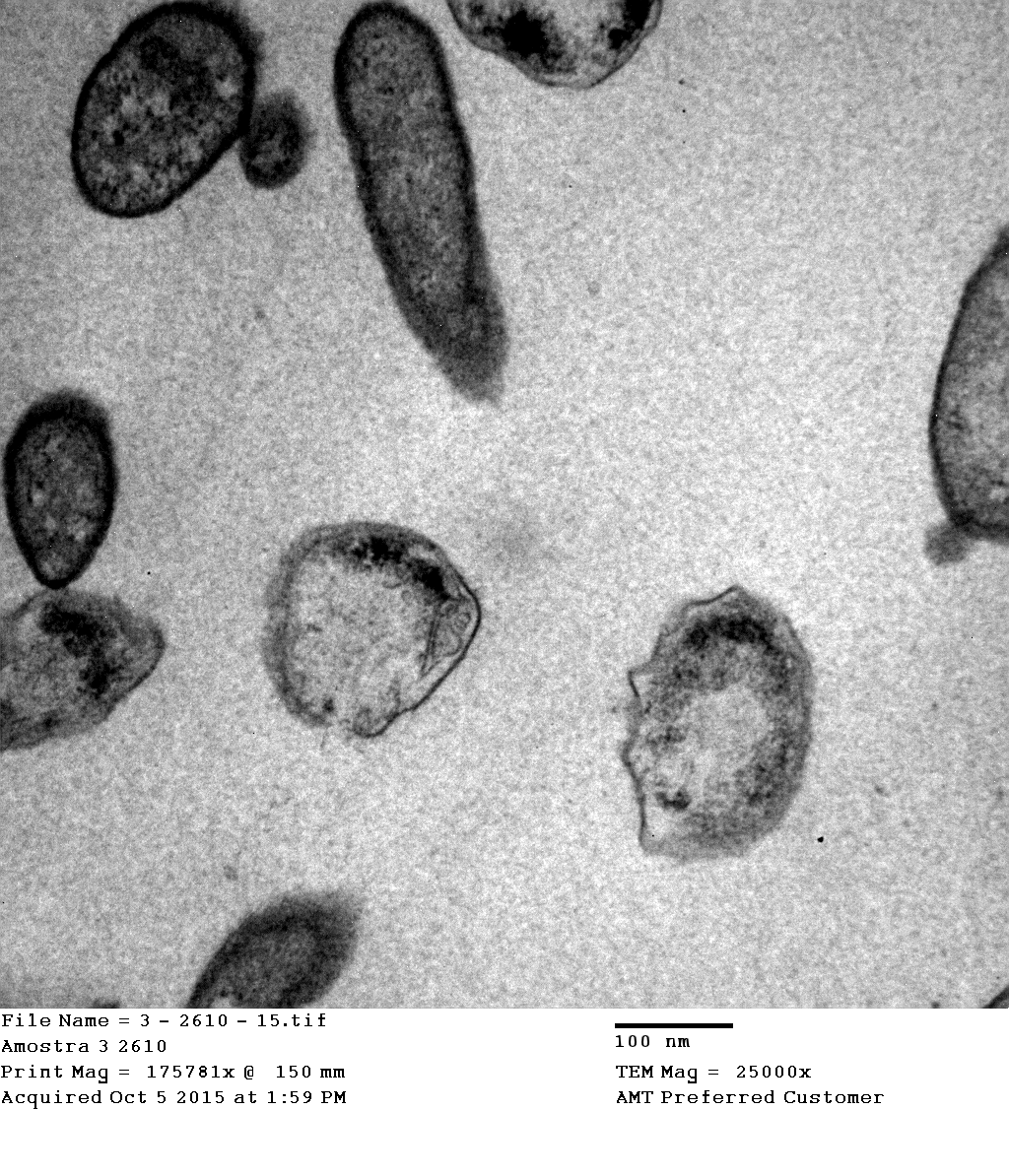

Supplement: Supplementary file 12 — Source Data Fig. 4 [file 44319_2024_60_MOESM12_ESM.zip › Fig 4/4C/raw images/3 - 2610 - 15.tif]

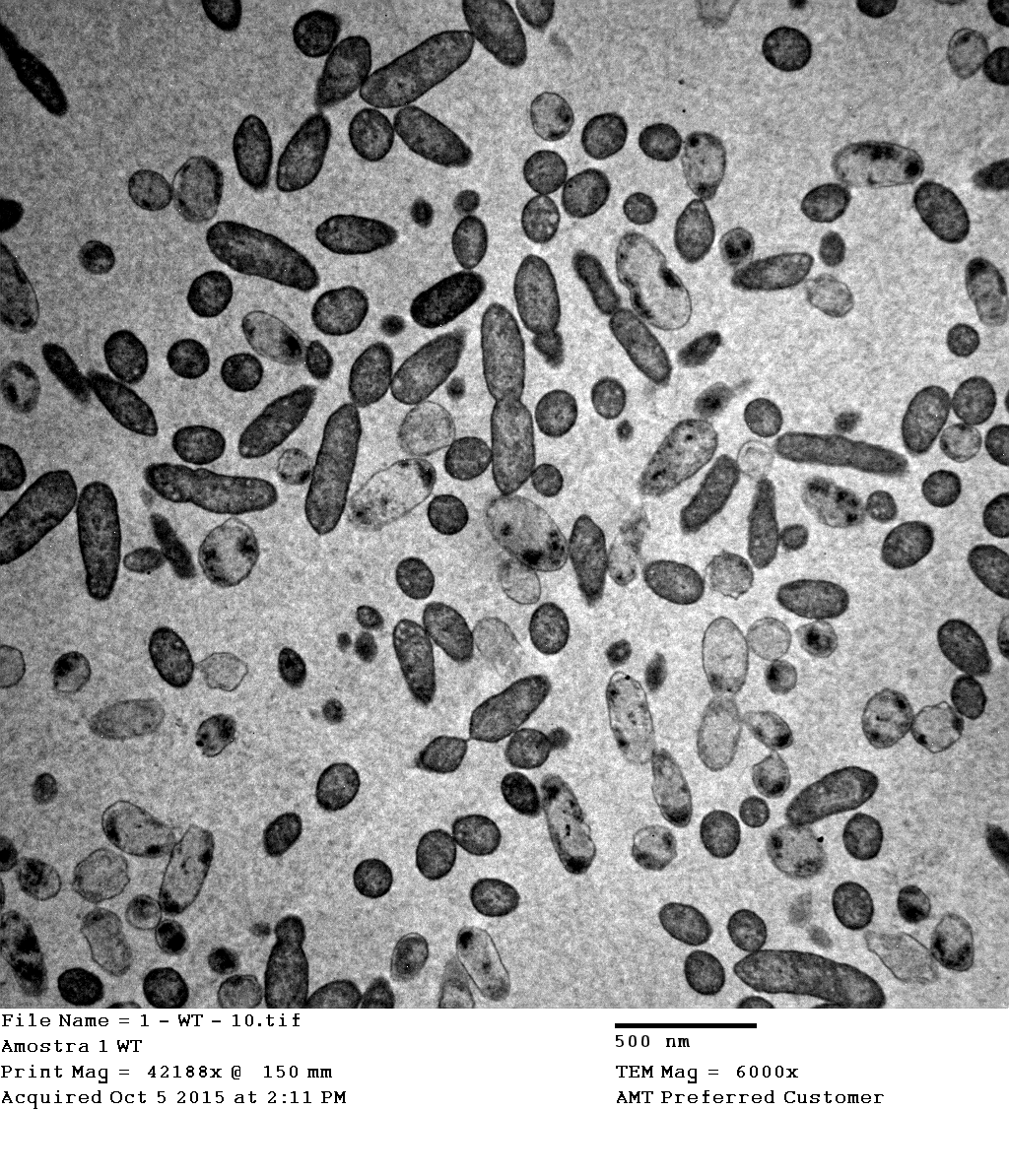

Supplement: Supplementary file 12 — Source Data Fig. 4 [file 44319_2024_60_MOESM12_ESM.zip › Fig 4/4C/raw images/1 - WT - 10.tif]

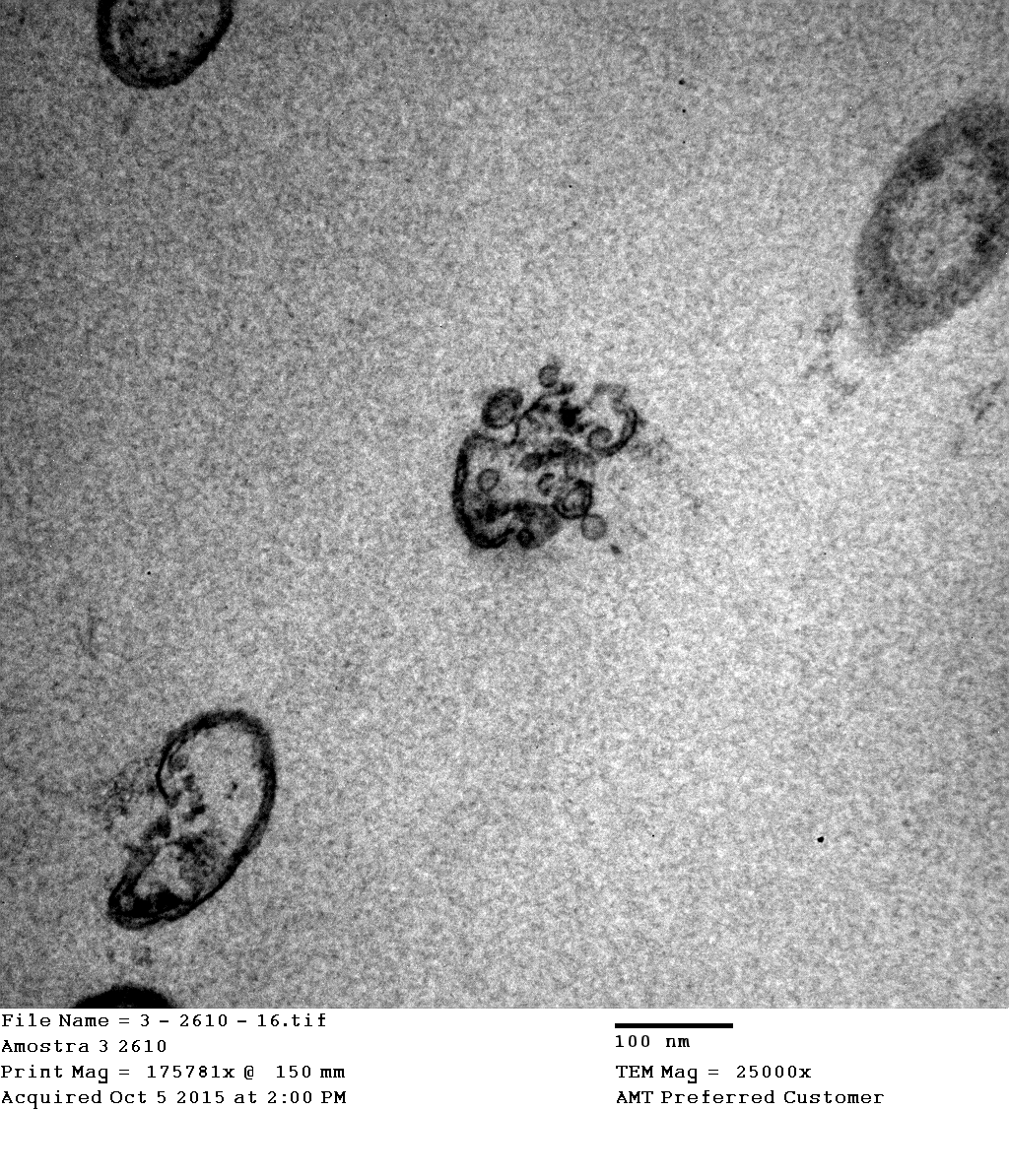

Supplement: Supplementary file 12 — Source Data Fig. 4 [file 44319_2024_60_MOESM12_ESM.zip › Fig 4/4C/raw images/3 - 2610 - 16.tif]

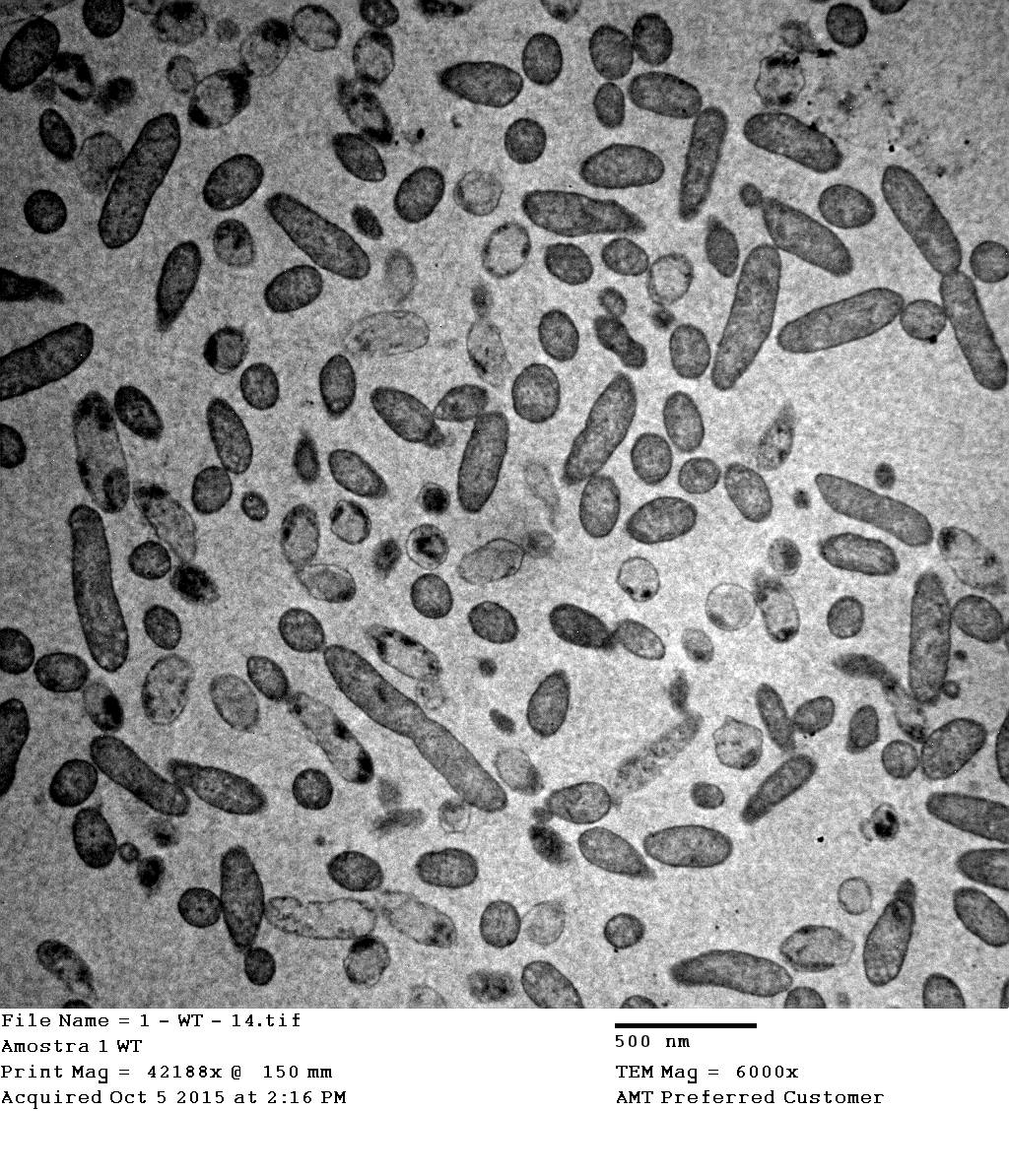

Supplement: Supplementary file 12 — Source Data Fig. 4 [file 44319_2024_60_MOESM12_ESM.zip › Fig 4/4C/raw images/1 - WT - 14.tif]

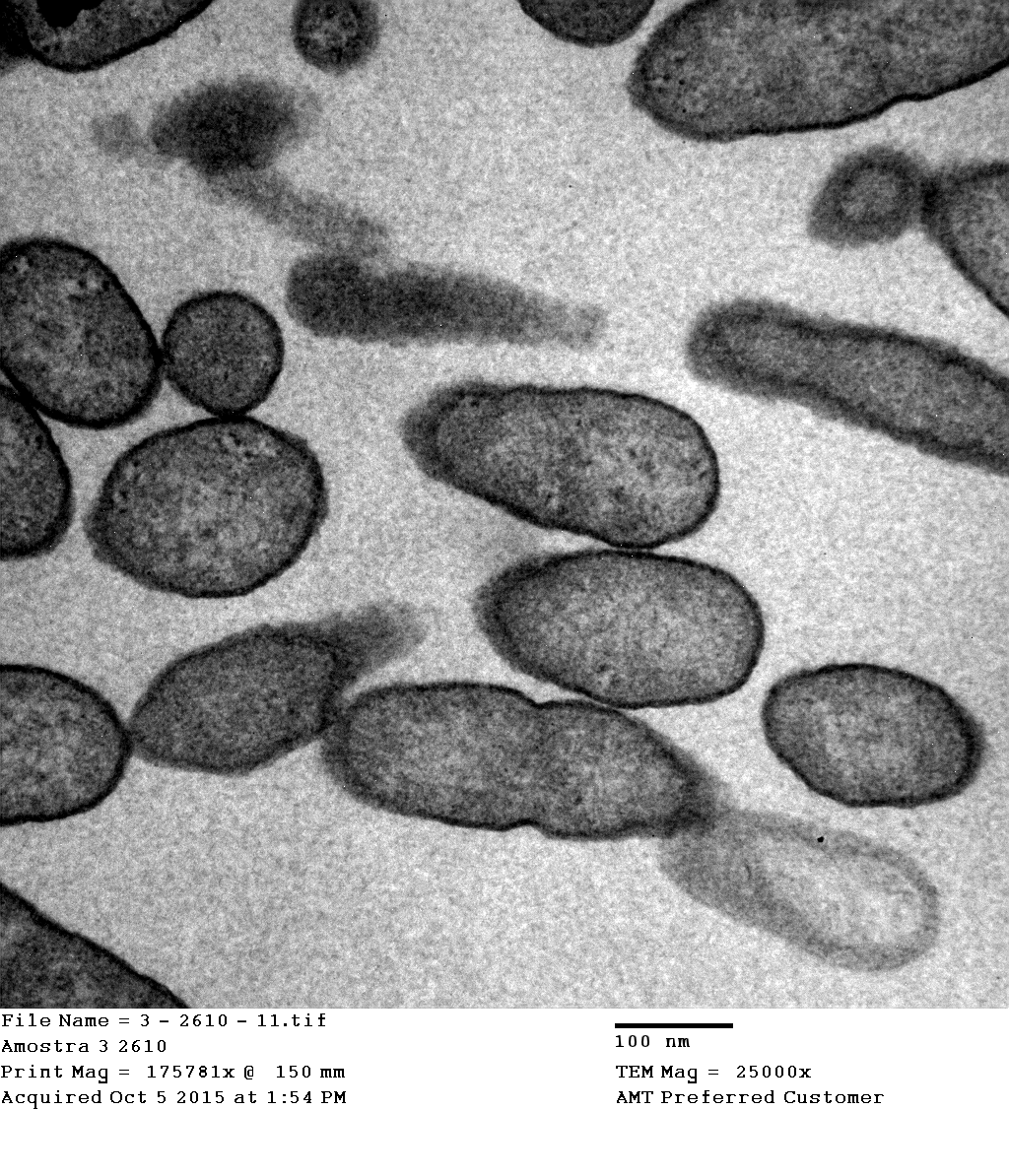

Supplement: Supplementary file 12 — Source Data Fig. 4 [file 44319_2024_60_MOESM12_ESM.zip › Fig 4/4C/raw images/3 - 2610 - 11.tif]

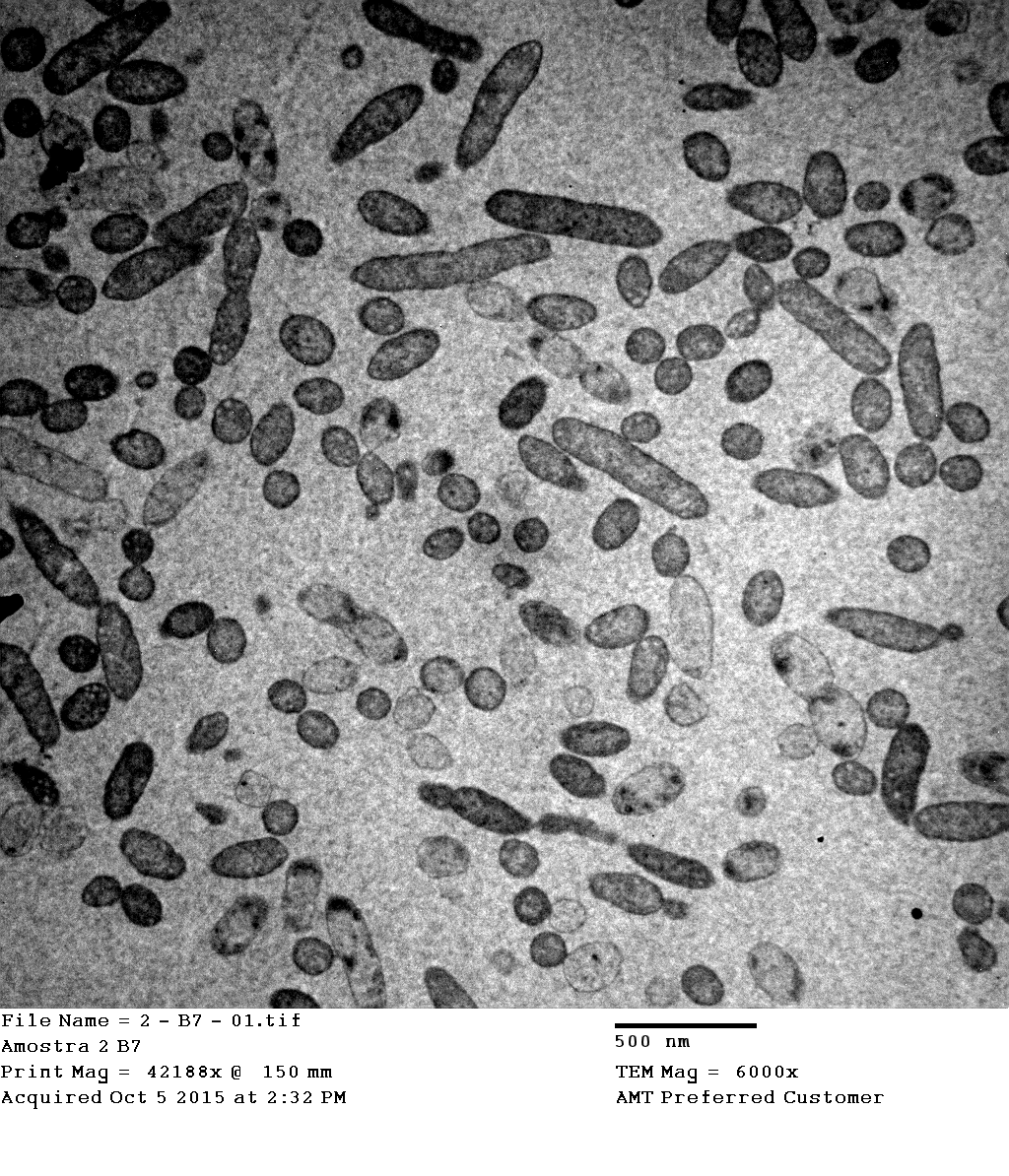

Supplement: Supplementary file 12 — Source Data Fig. 4 [file 44319_2024_60_MOESM12_ESM.zip › Fig 4/4C/raw images/2 - B7 - 01.tif]

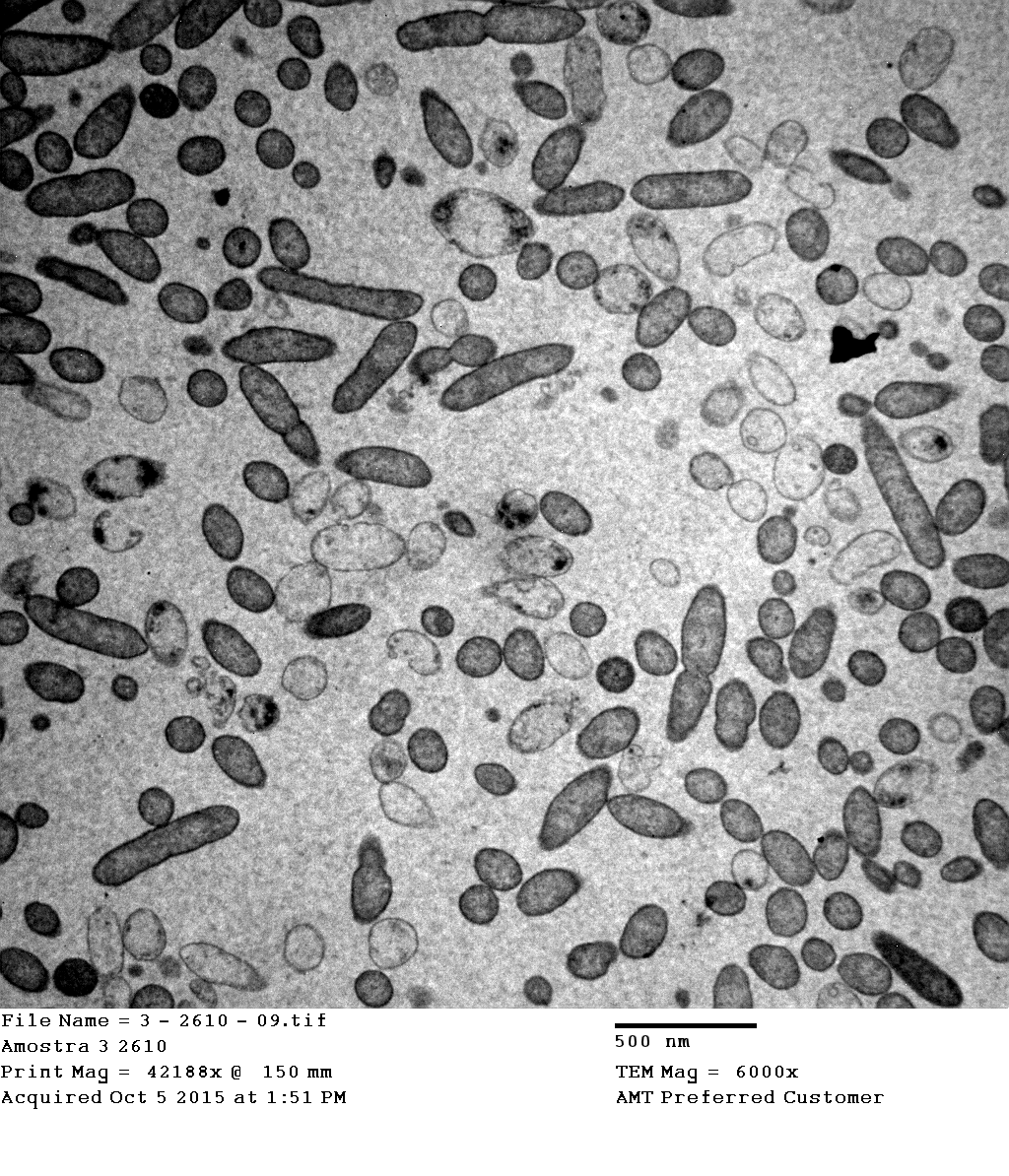

Supplement: Supplementary file 12 — Source Data Fig. 4 [file 44319_2024_60_MOESM12_ESM.zip › Fig 4/4C/raw images/3 - 2610 - 09.tif]

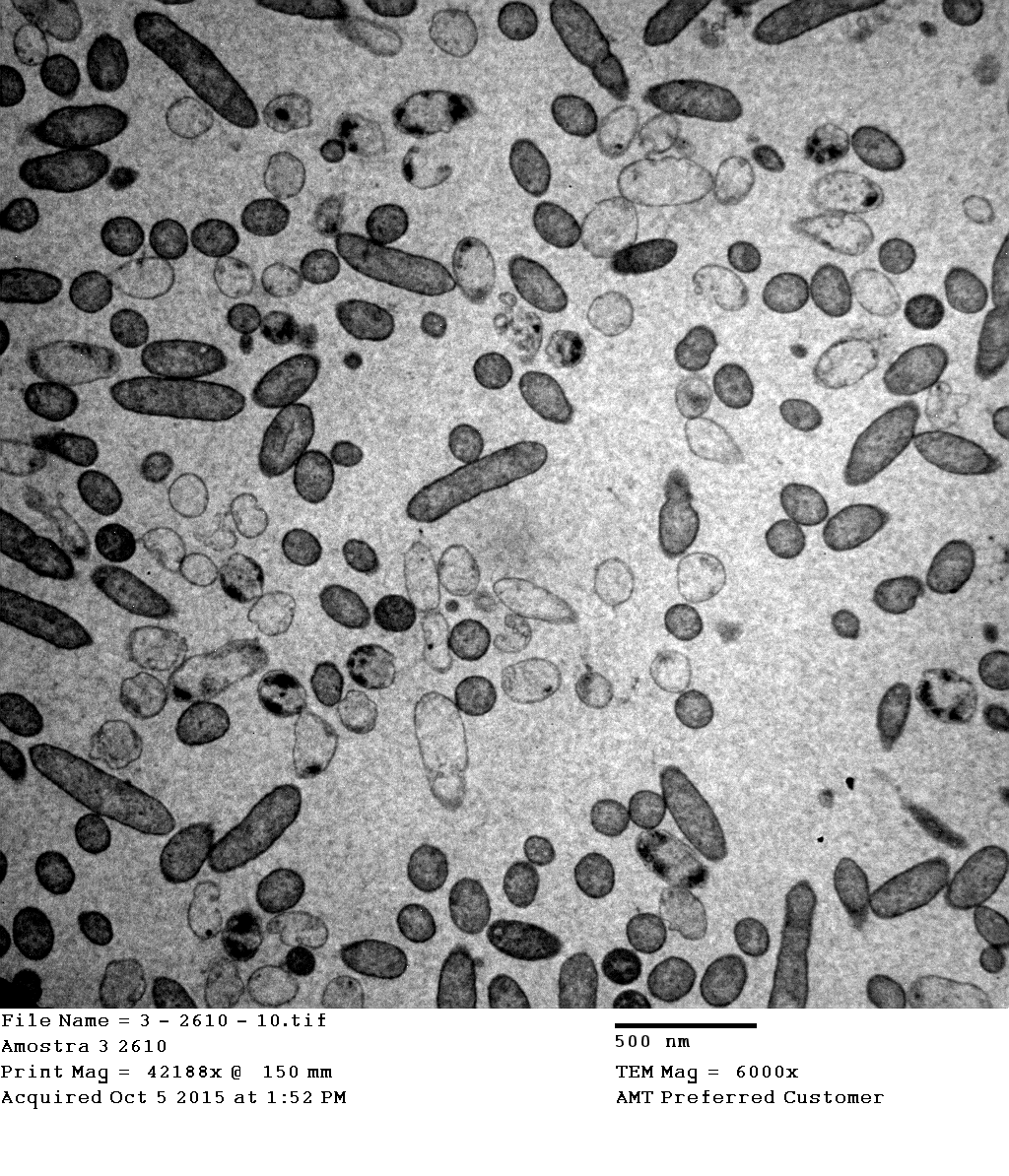

Supplement: Supplementary file 12 — Source Data Fig. 4 [file 44319_2024_60_MOESM12_ESM.zip › Fig 4/4C/raw images/3 - 2610 - 10.tif]

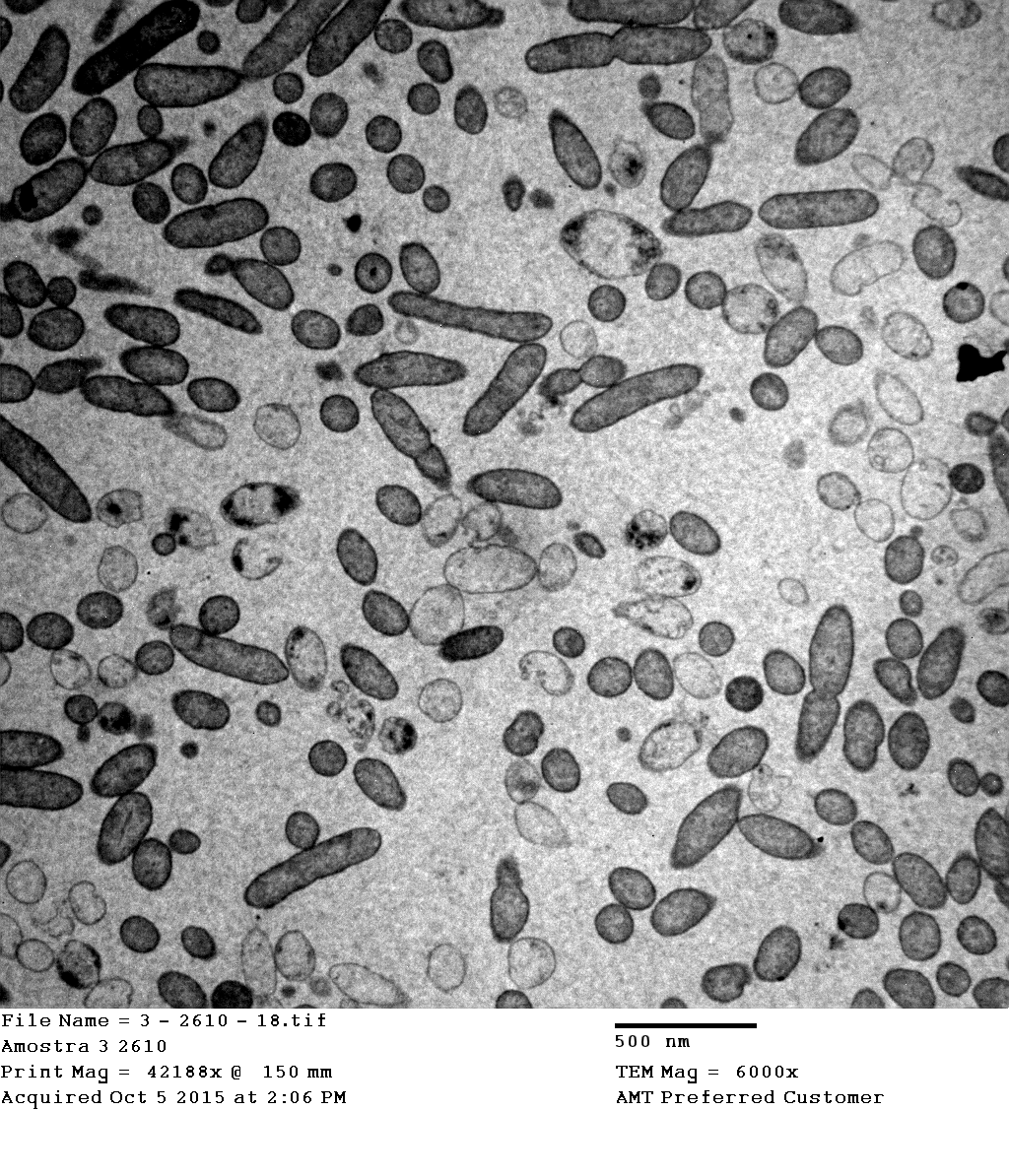

Supplement: Supplementary file 12 — Source Data Fig. 4 [file 44319_2024_60_MOESM12_ESM.zip › Fig 4/4C/raw images/3 - 2610 - 18.tif]

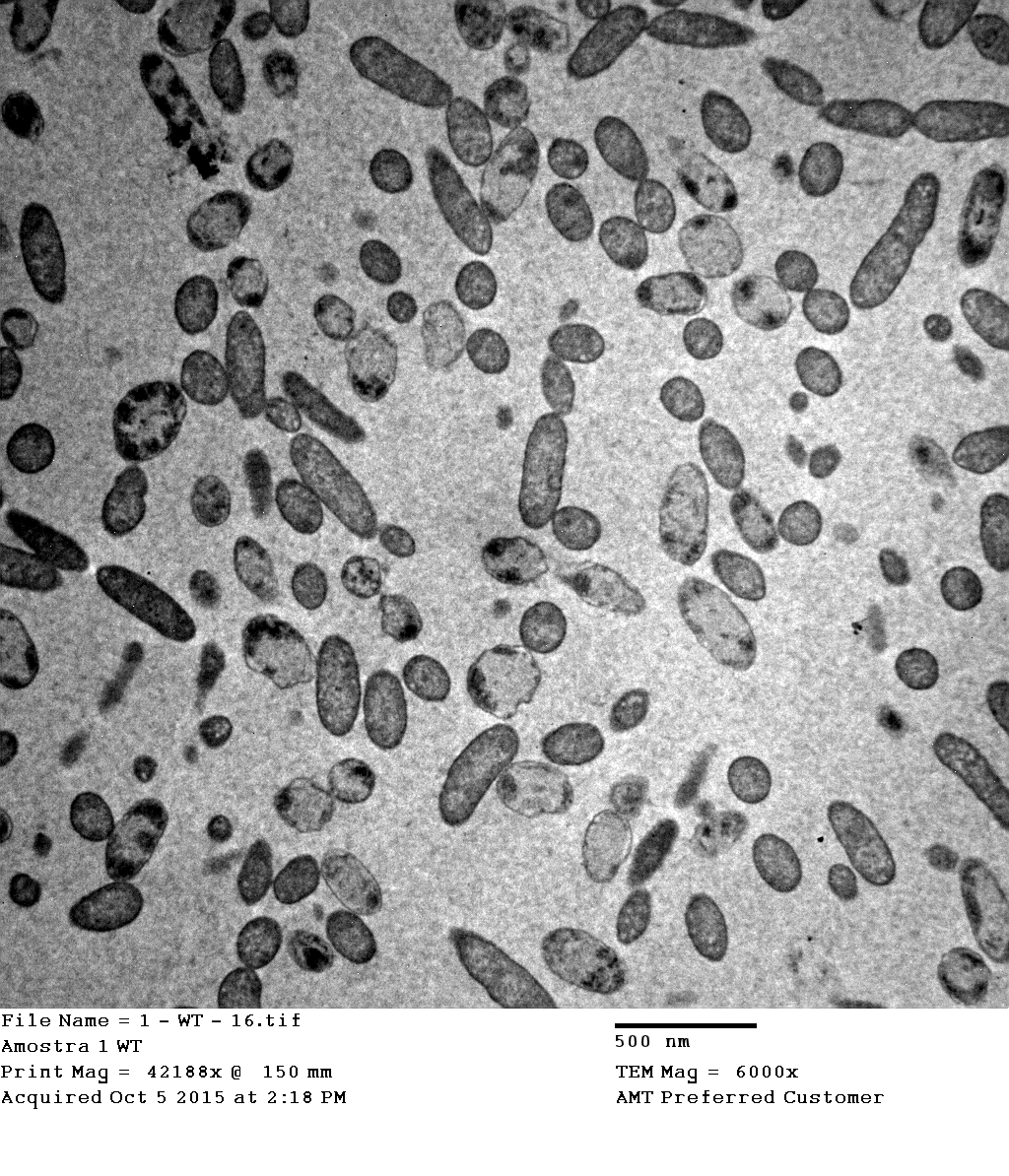

Supplement: Supplementary file 12 — Source Data Fig. 4 [file 44319_2024_60_MOESM12_ESM.zip › Fig 4/4C/raw images/1 - WT - 16.tif]
