# Supplementary material for: Xanthomonas immunity proteins protect against the cis-toxic effects of their cognate T4SS effectors
Source: EMBO Rep. 2024 Feb 8;25(3):27. doi: 10.1038/s44319-024-00060-6 (PMC10933484; doi:10.1038/s44319-024-00060-6)
Supplement: Supplementary file 11 — Source Data Fig. 3 [file 44319_2024_60_MOESM11_ESM.zip › Fig3 no micrographs/Read me Fig 3.docx]

The Microscopy Movies folder containing raw data (tif files) of the movies that were analyzed in Figure 3 and presented in the Movies S1-S5 can be accessed at BioStudies accession number S-BSST1204 (<https://www.ebi.ac.uk/biostudies/studies/S-BSST1204>). Each movie has 10 min per frame and two channels.

The Movie Raw Data Analysis folder contains images used to count total number of cells (bright field) and to count the number of cells undergoing lysis as determined by propidium iodide permeability (PIP).

The Fig 3 Numerical Data spreadsheet contains the data derived from the movies used to produce the bar graphs in Figure 3 and the data in Table S4
