# Supplementary material for: Xanthomonas immunity proteins protect against the cis-toxic effects of their cognate T4SS effectors
Source: EMBO Rep. 2024 Feb 8;25(3):27. doi: 10.1038/s44319-024-00060-6 (PMC10933484; doi:10.1038/s44319-024-00060-6)
Supplement: Supplementary file 12 — Source Data Fig. 4 [file 44319_2024_60_MOESM12_ESM.zip › Fig 4/4B /readme Fig4B.docx]

Transmission electron micrographs of X. citri wild type and ΔXAC2610 cells
