# Supplementary material for: Xanthomonas immunity proteins protect against the cis-toxic effects of their cognate T4SS effectors
Source: EMBO Rep. 2024 Feb 8;25(3):27. doi: 10.1038/s44319-024-00060-6 (PMC10933484; doi:10.1038/s44319-024-00060-6)
Supplement: Supplementary file 12 — Source Data Fig. 4 [file 44319_2024_60_MOESM12_ESM.zip › Fig 4/4A/micrographs/readme Fig4A.docx]

Micrographs of X. citri ΔXAC2610 cells at time zero (T=0) and time of spheroplast formation event at T=1.

Examples A,B,C,D,E,F at T=0 and T=1.
