# Supplementary material for: Xanthomonas immunity proteins protect against the cis-toxic effects of their cognate T4SS effectors
Source: EMBO Rep. 2024 Feb 8;25(3):27. doi: 10.1038/s44319-024-00060-6 (PMC10933484; doi:10.1038/s44319-024-00060-6)
Supplement: Supplementary file 12 — Source Data Fig. 4 [file 44319_2024_60_MOESM12_ESM.zip › Fig 4/4C/readme Fig4C.docx]

Numerical data file contains total cell counts, damaged cells and % of damaged cells observed in transmission electron micrographs of specific X. citri strains (r1,r2,r3..). Each column is named according to the strain analyzed. Note that KO2610c indicates X. citri ΔXAC2610 complemented with a plasmid expressing the xac2610 gene..

Mean and standard error of replicates are shown at the end of the table in columns AX to BG.

The raw images directory contains all of the micrographs used to analyze the counts in this figure.
