# Supplementary figures and images for: Xanthomonas immunity proteins protect against the cis-toxic effects of their cognate T4SS effectors (part 2 of 2)
Source: EMBO Rep. 2024 Feb 8;25(3):27. doi: 10.1038/s44319-024-00060-6 (PMC10933484; doi:10.1038/s44319-024-00060-6)

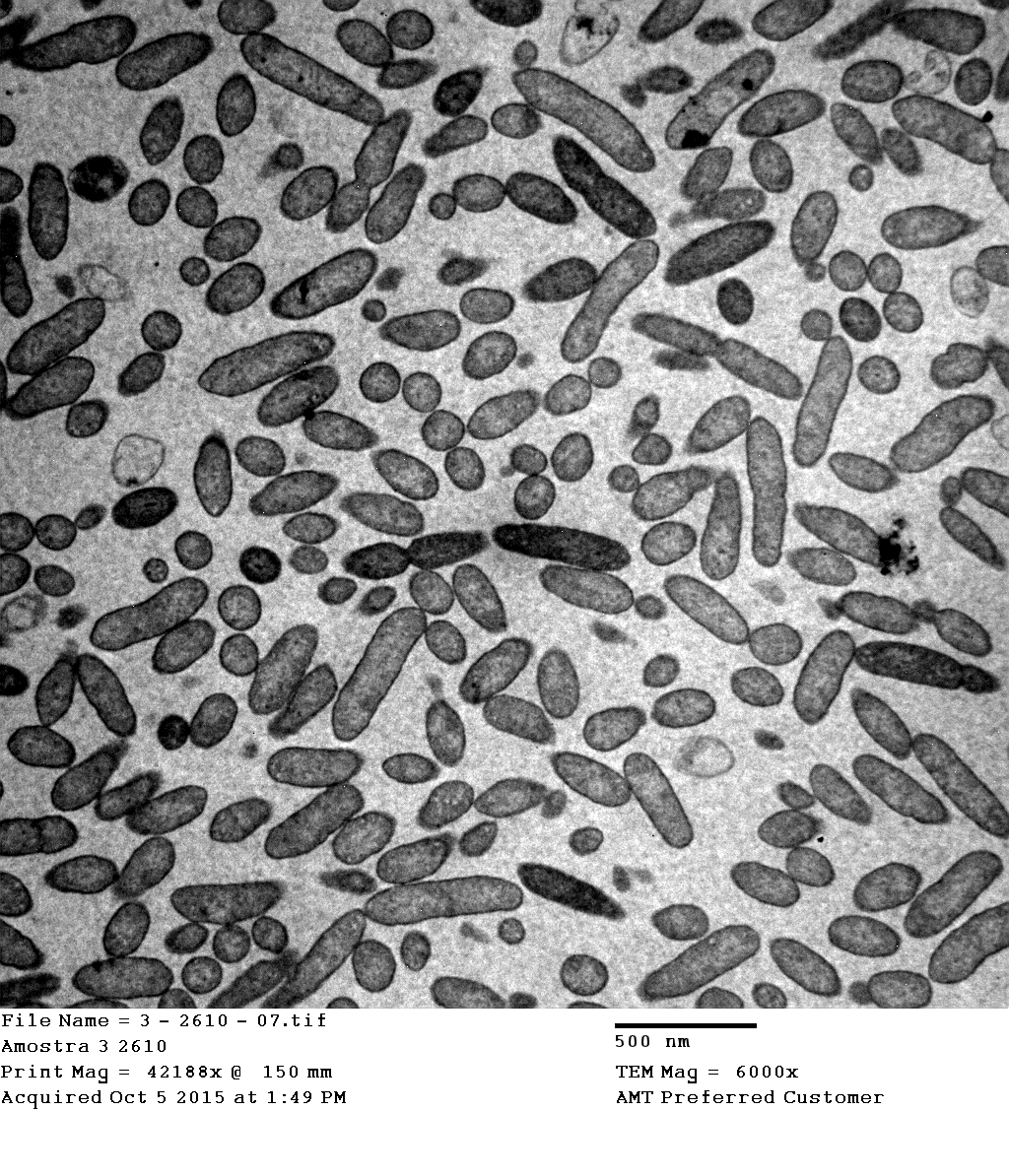

Supplement: Supplementary file 12 — Source Data Fig. 4 [file 44319_2024_60_MOESM12_ESM.zip › Fig 4/4C/raw images/3 - 2610 - 07.tif]

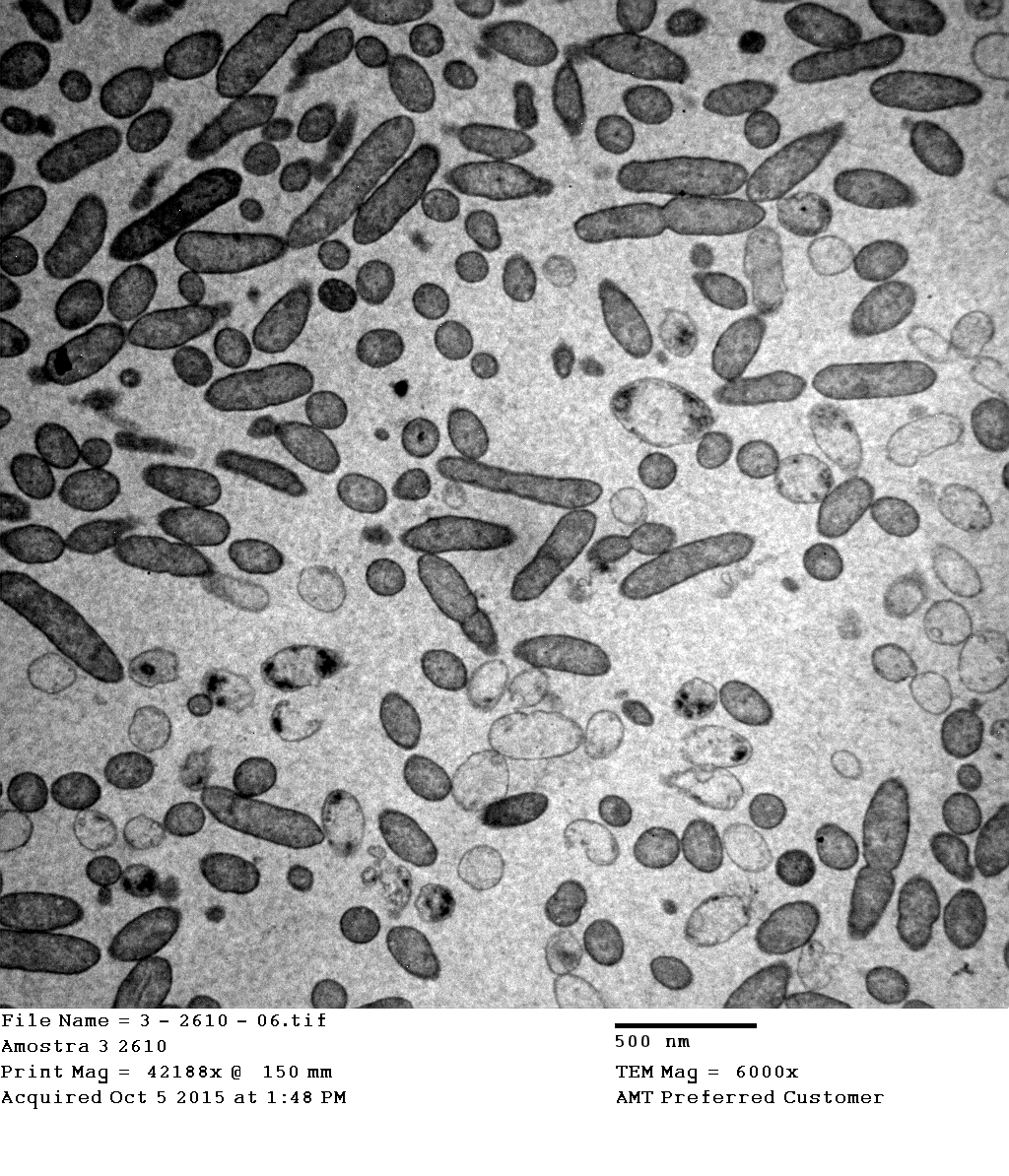

Supplement: Supplementary file 12 — Source Data Fig. 4 [file 44319_2024_60_MOESM12_ESM.zip › Fig 4/4C/raw images/3 - 2610 - 06.tif]

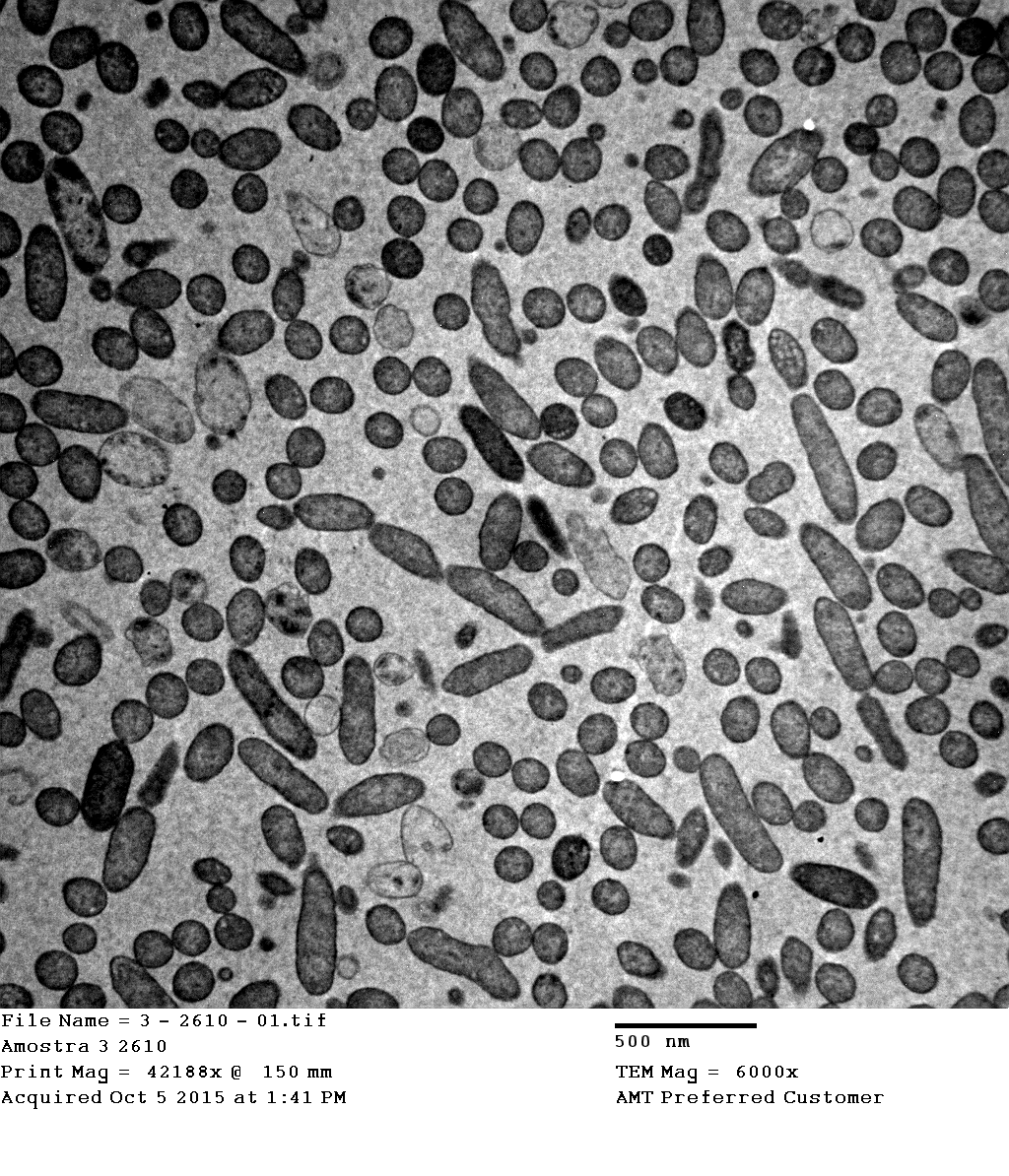

Supplement: Supplementary file 12 — Source Data Fig. 4 [file 44319_2024_60_MOESM12_ESM.zip › Fig 4/4C/raw images/3 - 2610 - 01.tif]

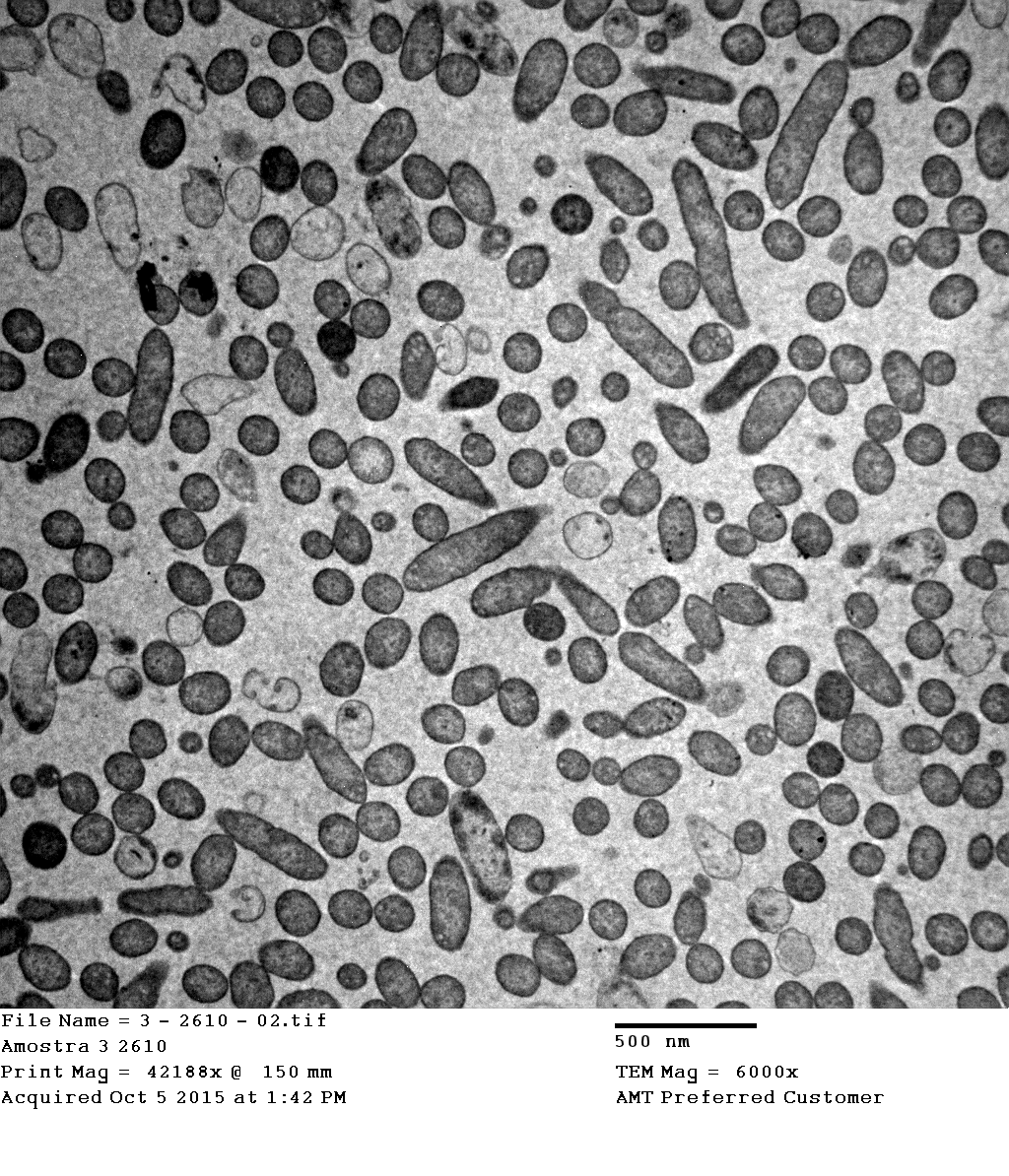

Supplement: Supplementary file 12 — Source Data Fig. 4 [file 44319_2024_60_MOESM12_ESM.zip › Fig 4/4C/raw images/3 - 2610 - 02.tif]

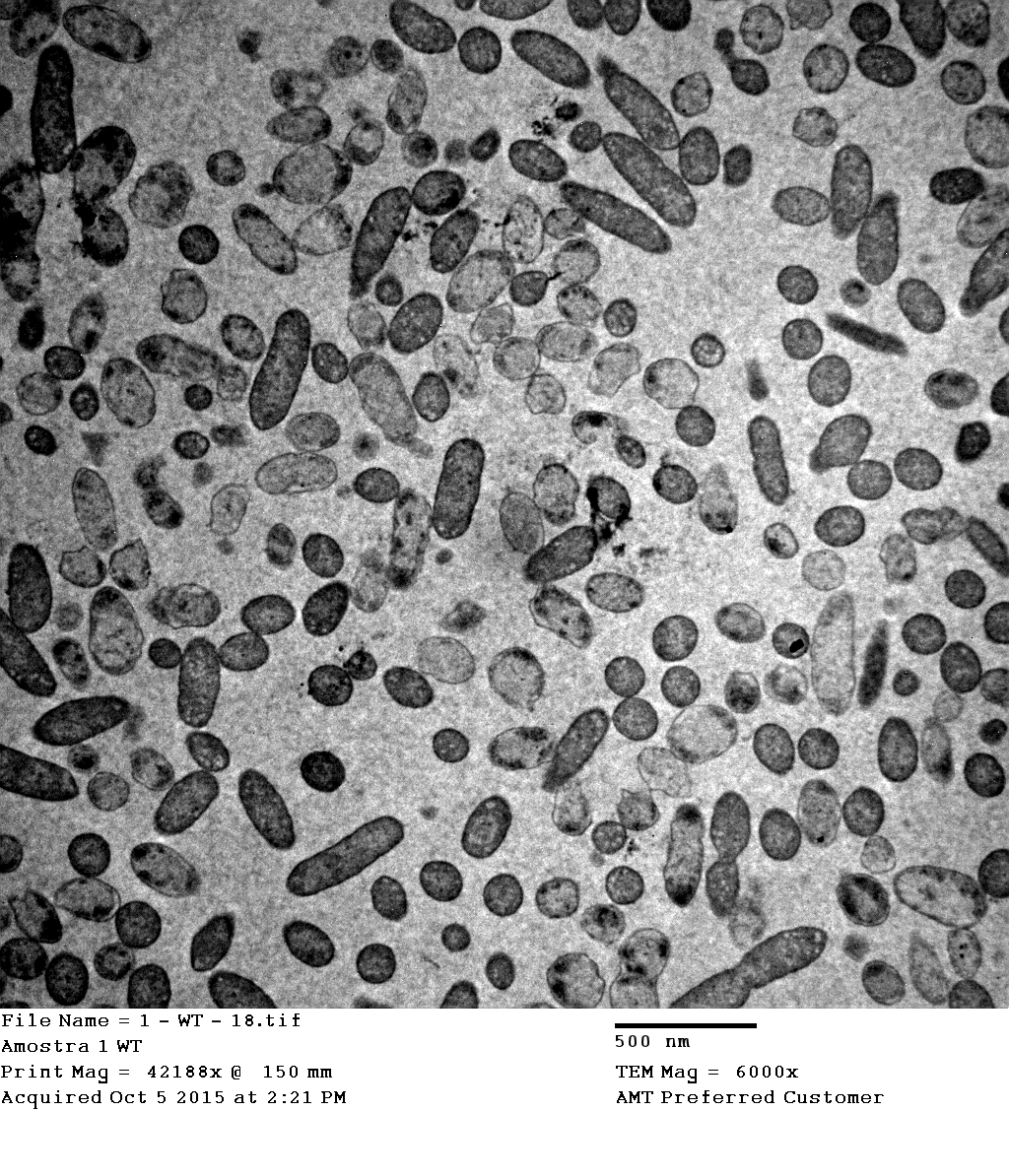

Supplement: Supplementary file 12 — Source Data Fig. 4 [file 44319_2024_60_MOESM12_ESM.zip › Fig 4/4C/raw images/1 - WT - 18.tif]

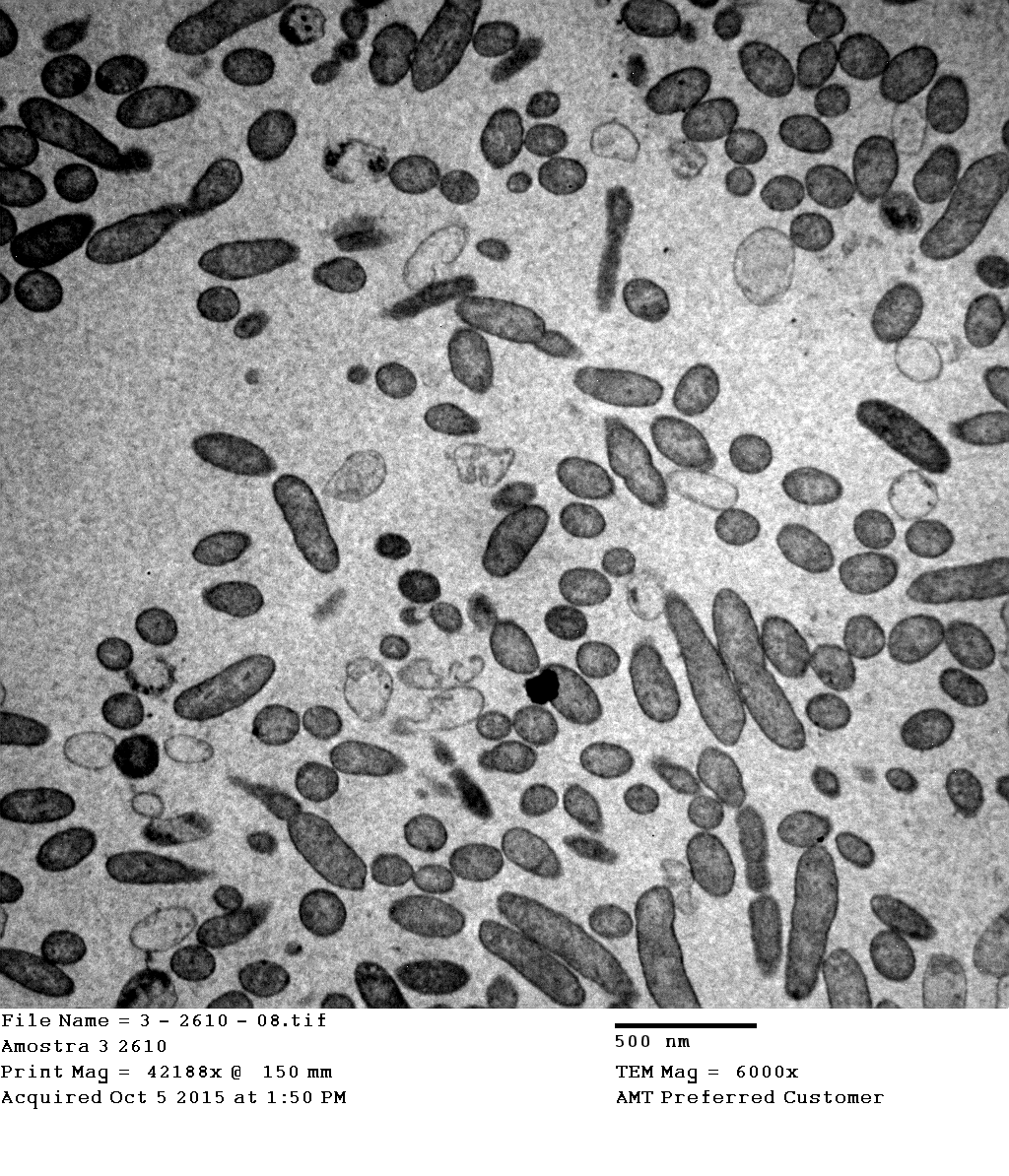

Supplement: Supplementary file 12 — Source Data Fig. 4 [file 44319_2024_60_MOESM12_ESM.zip › Fig 4/4C/raw images/3 - 2610 - 08.tif]

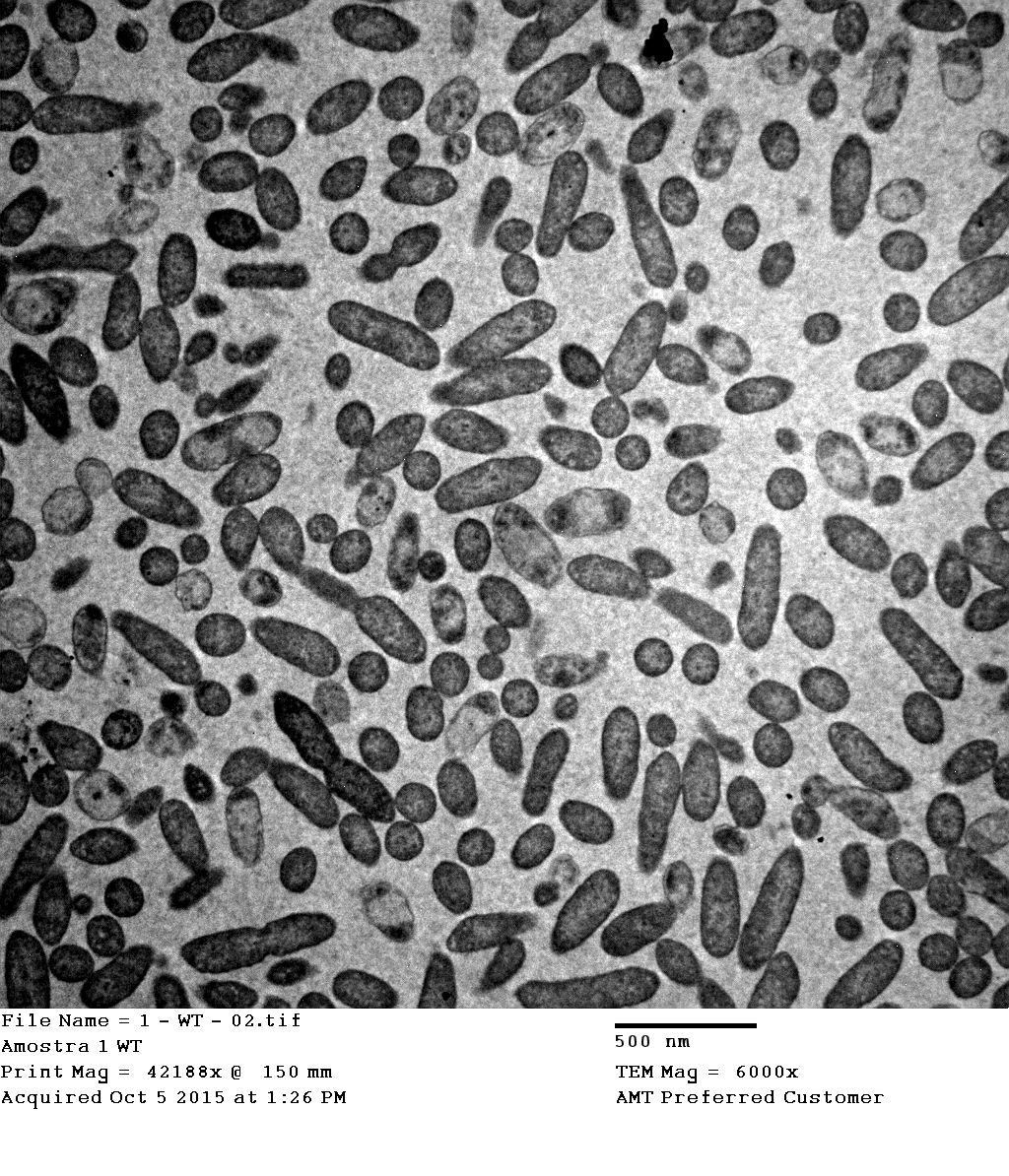

Supplement: Supplementary file 12 — Source Data Fig. 4 [file 44319_2024_60_MOESM12_ESM.zip › Fig 4/4C/raw images/1 - WT - 02.tif]

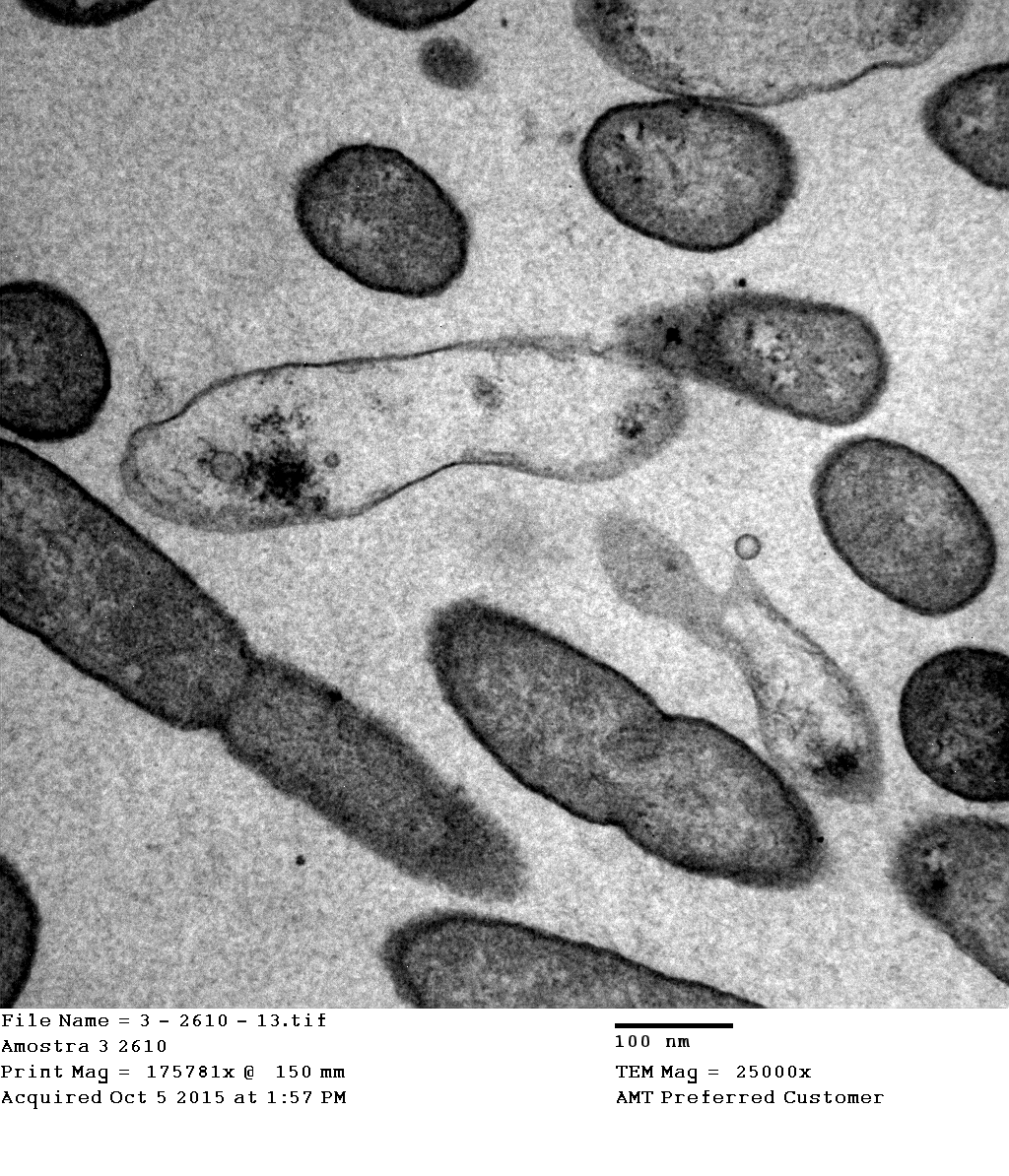

Supplement: Supplementary file 12 — Source Data Fig. 4 [file 44319_2024_60_MOESM12_ESM.zip › Fig 4/4C/raw images/3 - 2610 - 13.tif]

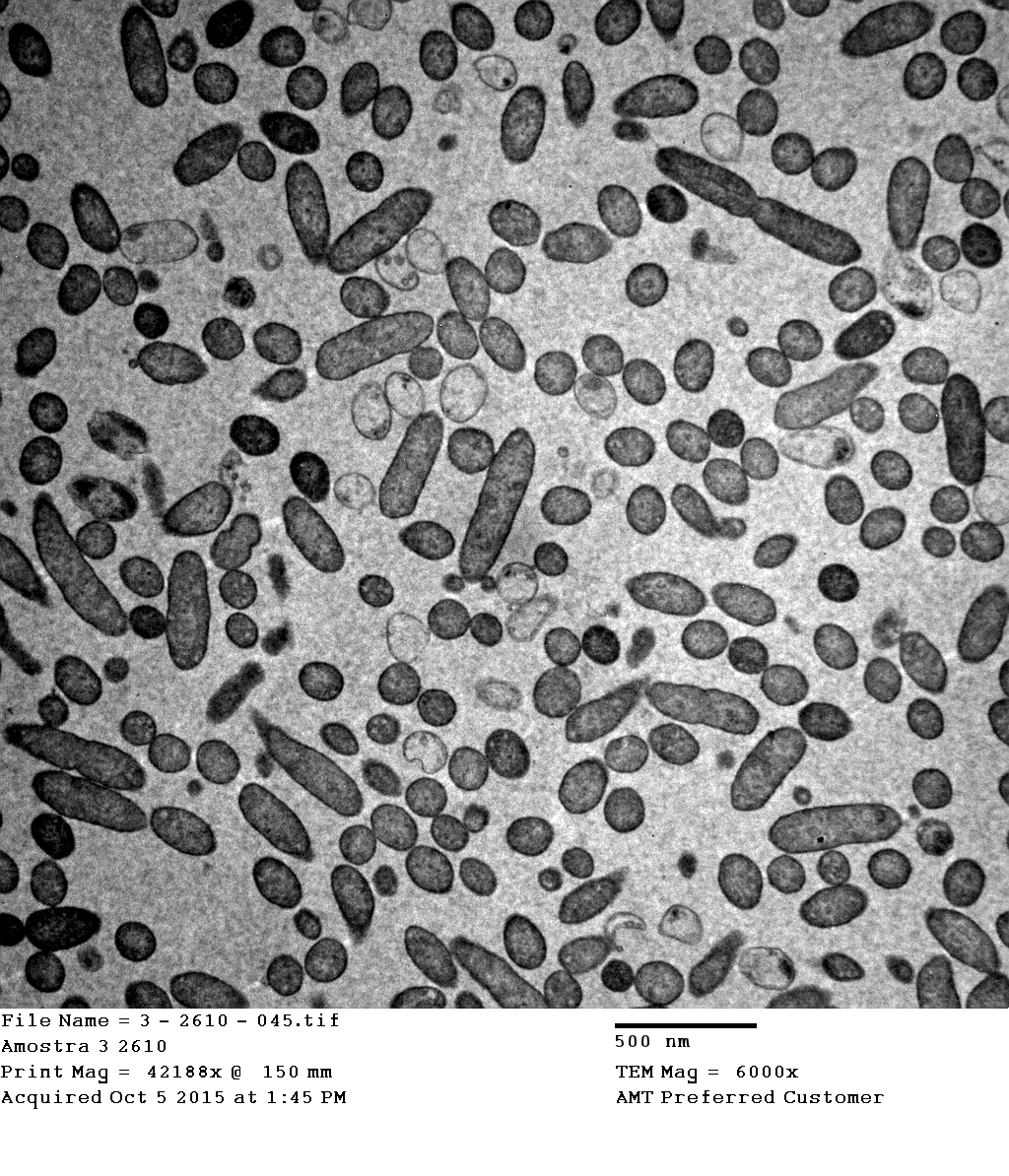

Supplement: Supplementary file 12 — Source Data Fig. 4 [file 44319_2024_60_MOESM12_ESM.zip › Fig 4/4C/raw images/3 - 2610 - 05.tif]

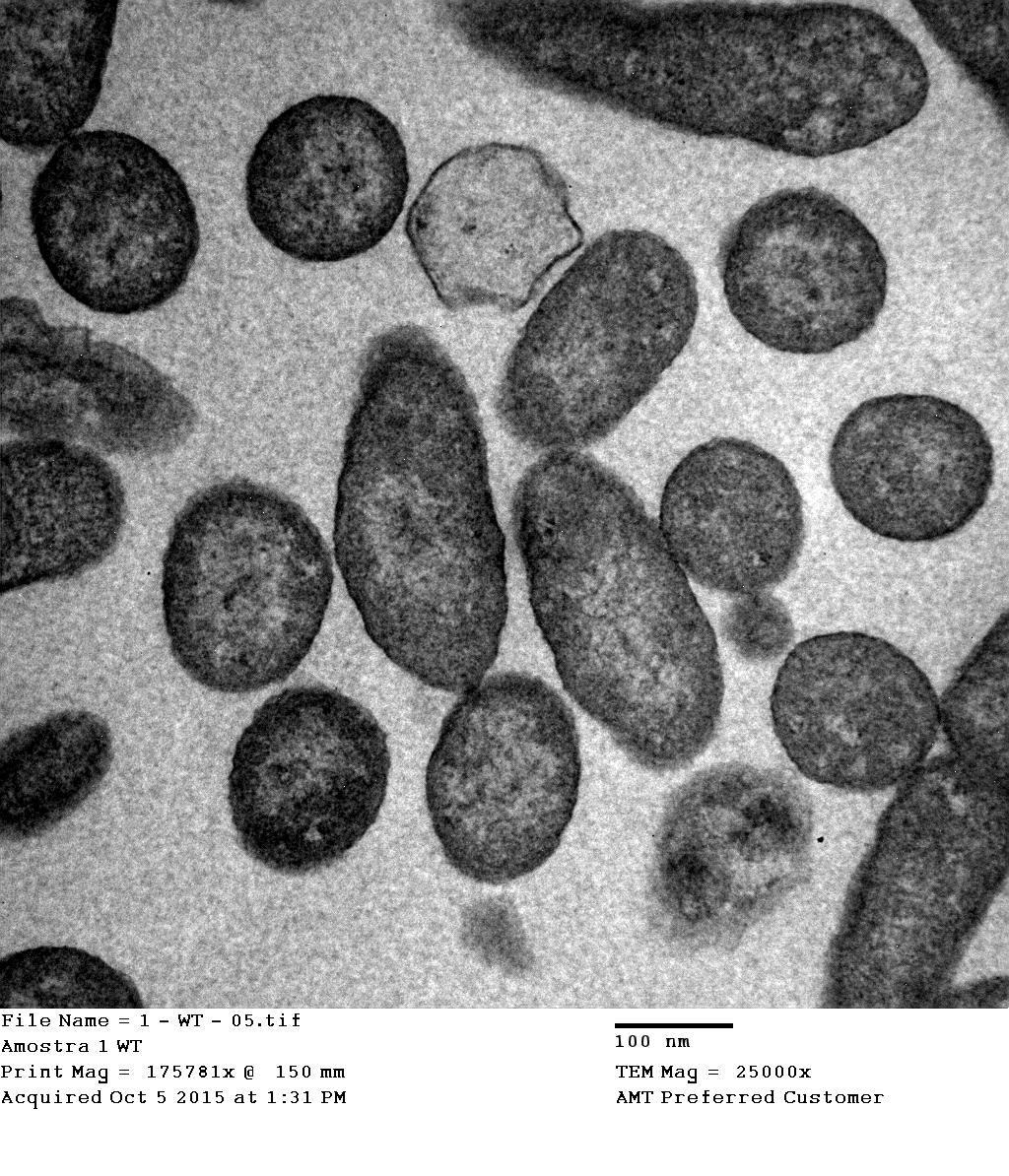

Supplement: Supplementary file 12 — Source Data Fig. 4 [file 44319_2024_60_MOESM12_ESM.zip › Fig 4/4C/raw images/1 - WT - 05.tif]

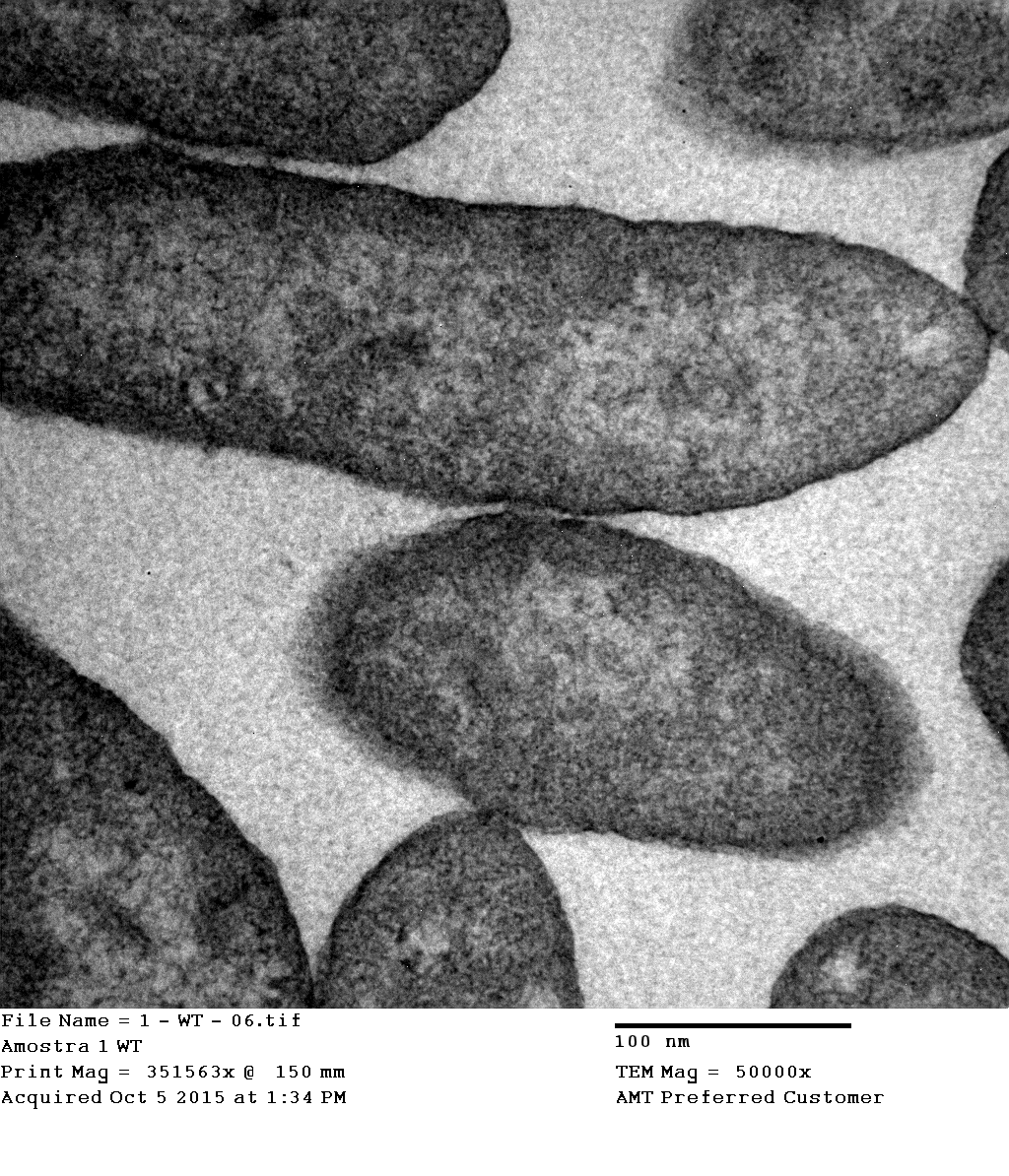

Supplement: Supplementary file 12 — Source Data Fig. 4 [file 44319_2024_60_MOESM12_ESM.zip › Fig 4/4C/raw images/1 - WT - 06.tif]

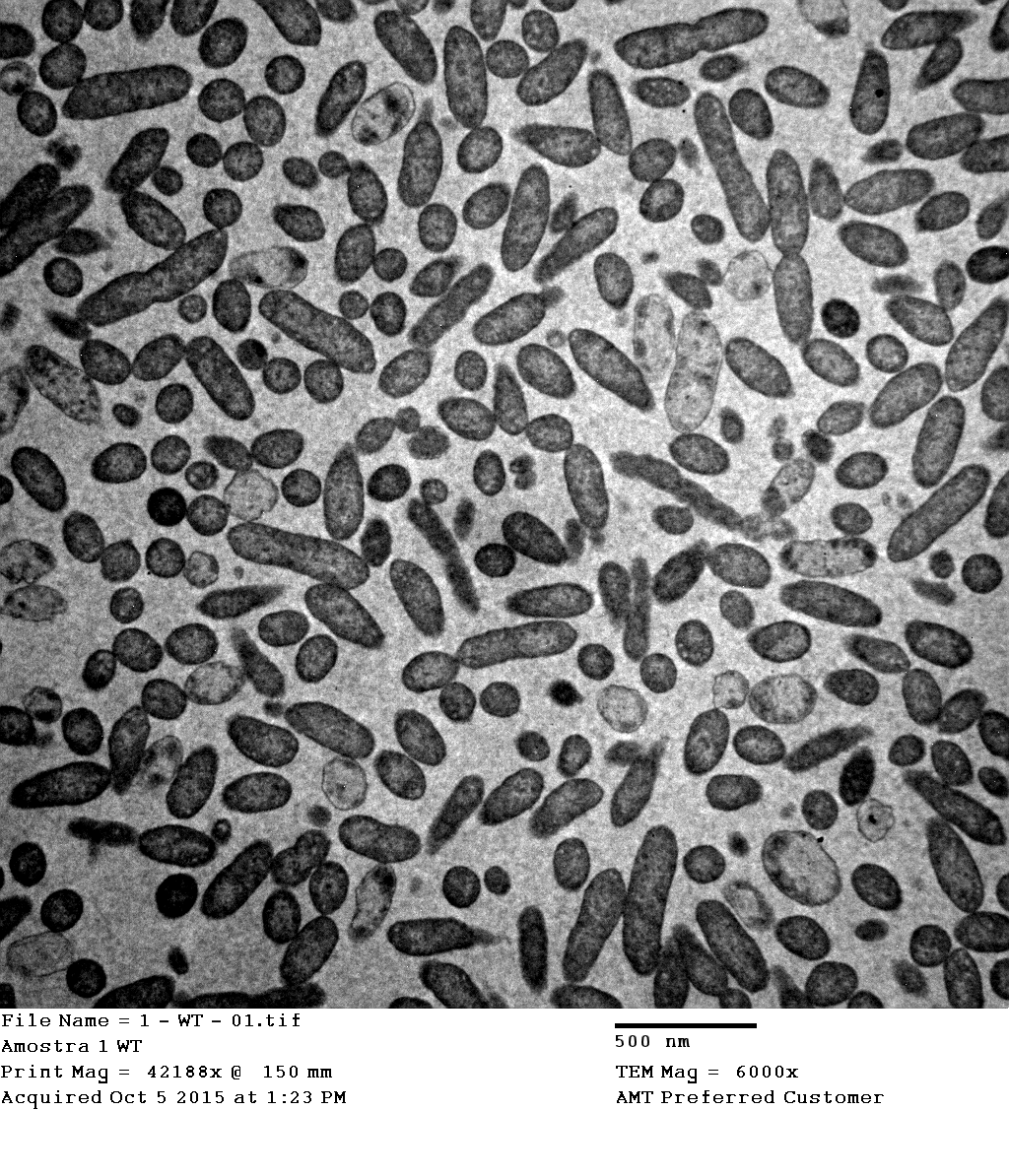

Supplement: Supplementary file 12 — Source Data Fig. 4 [file 44319_2024_60_MOESM12_ESM.zip › Fig 4/4C/raw images/1 - WT - 01.tif]

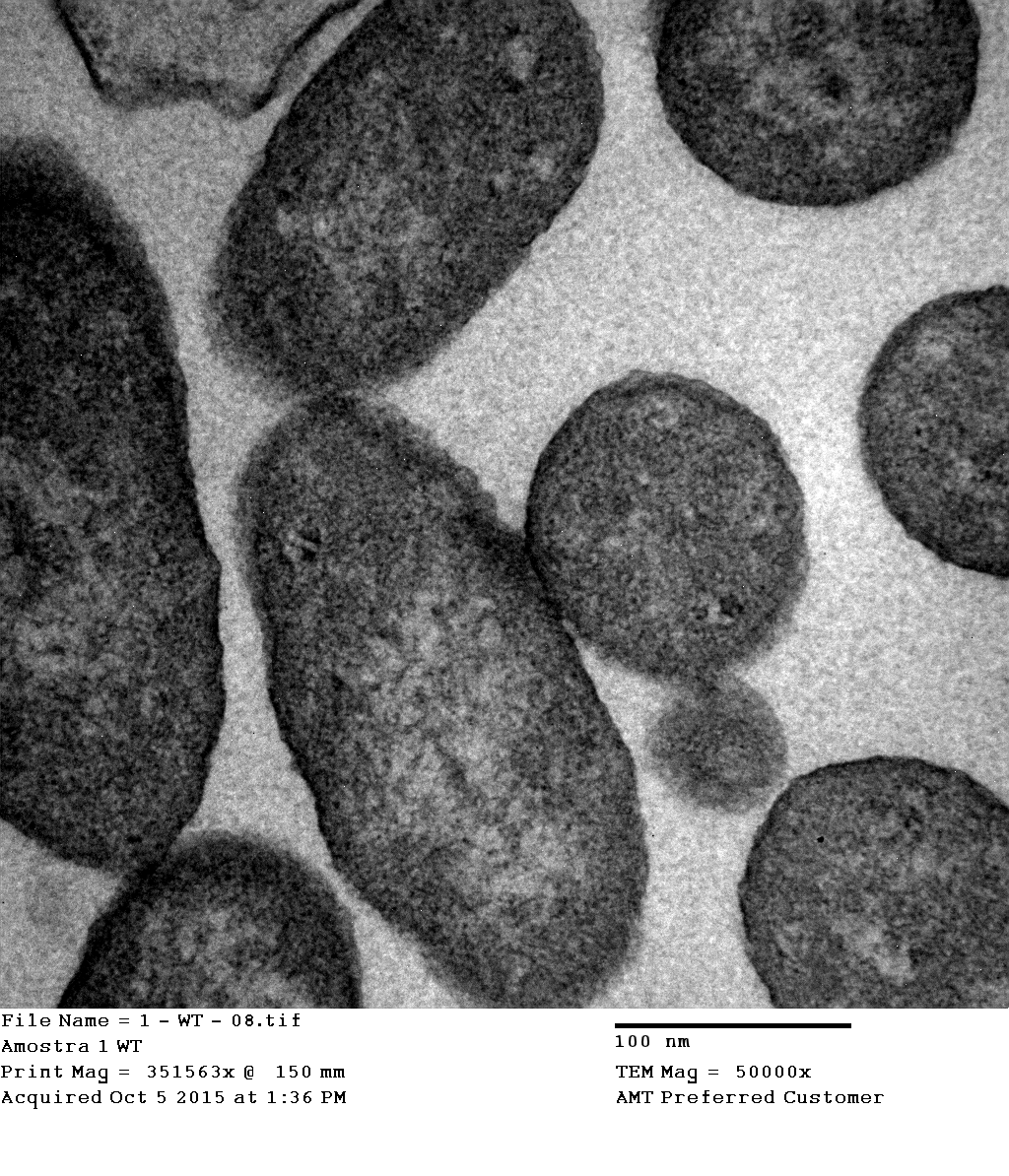

Supplement: Supplementary file 12 — Source Data Fig. 4 [file 44319_2024_60_MOESM12_ESM.zip › Fig 4/4C/raw images/1 - WT - 08.tif]

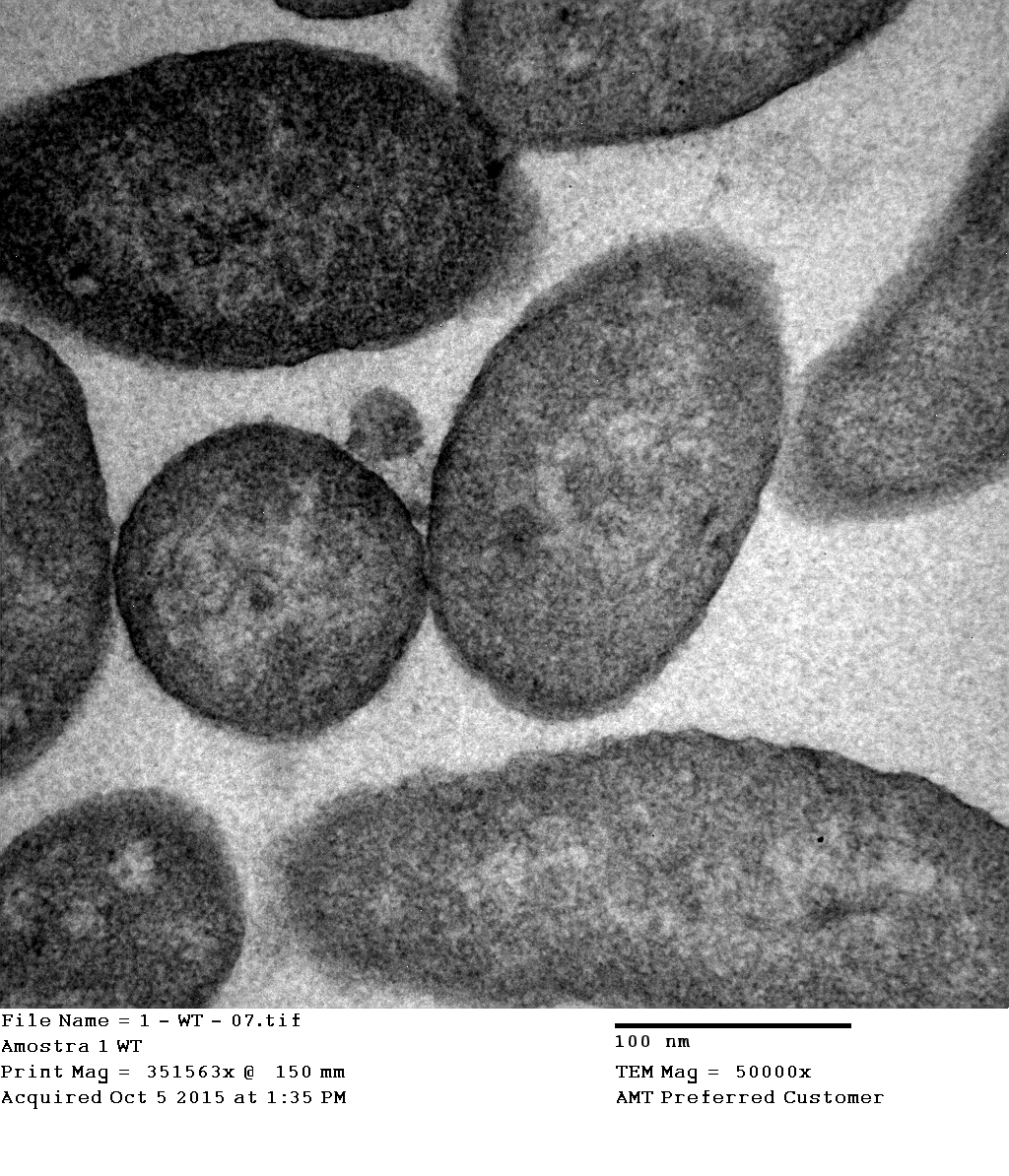

Supplement: Supplementary file 12 — Source Data Fig. 4 [file 44319_2024_60_MOESM12_ESM.zip › Fig 4/4C/raw images/1 - WT - 07.tif]

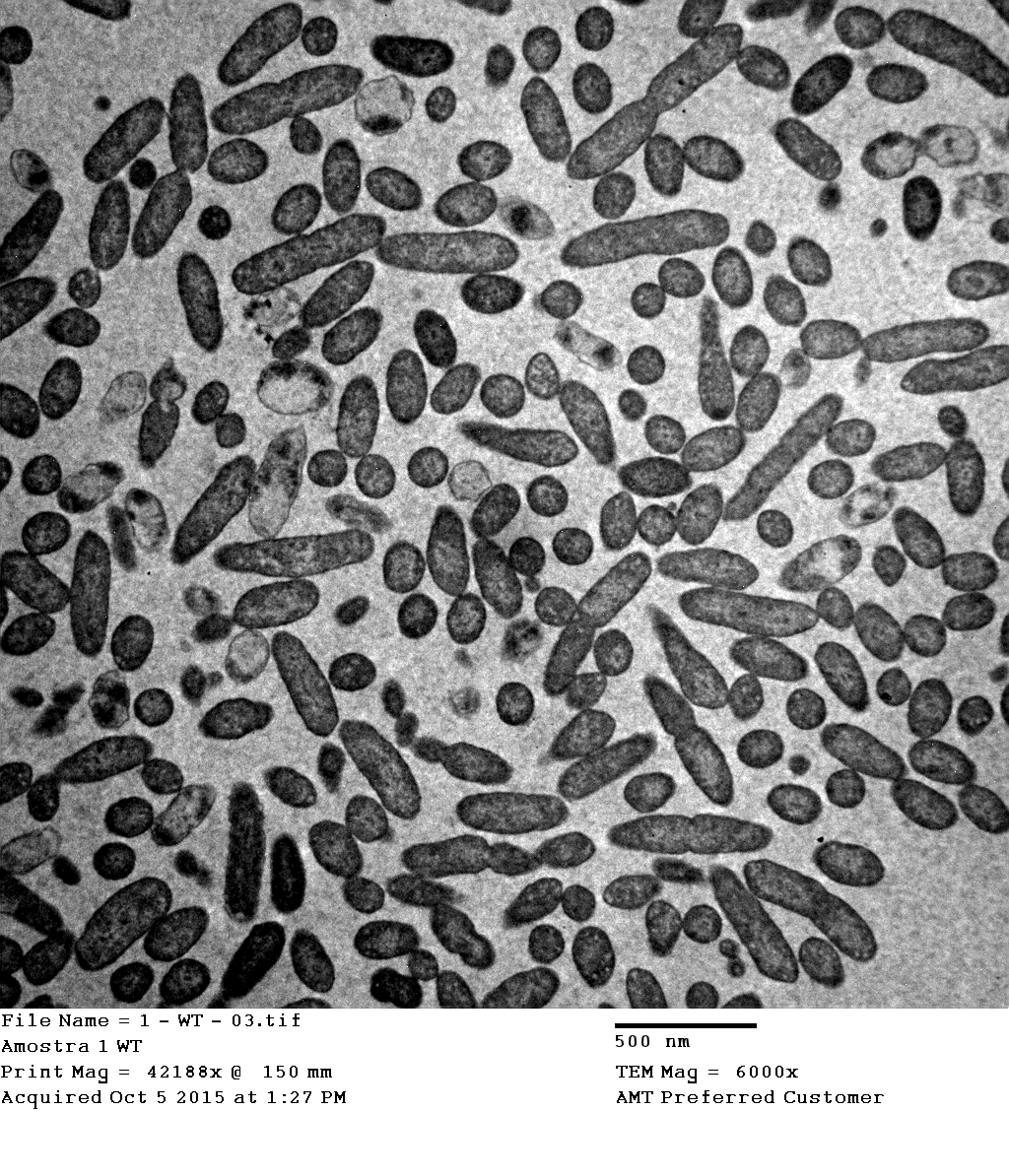

Supplement: Supplementary file 12 — Source Data Fig. 4 [file 44319_2024_60_MOESM12_ESM.zip › Fig 4/4C/raw images/1 - WT - 03.tif]

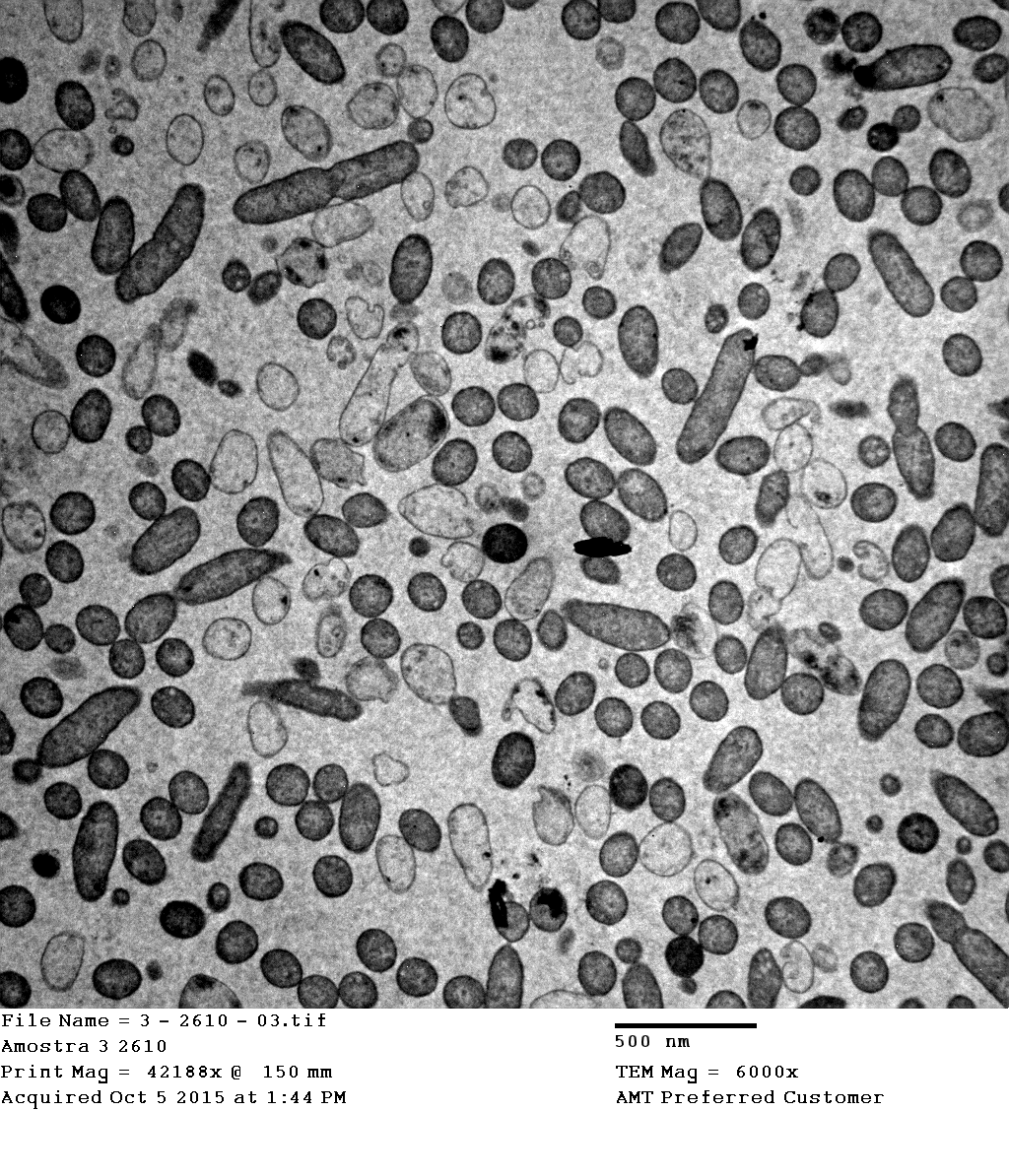

Supplement: Supplementary file 12 — Source Data Fig. 4 [file 44319_2024_60_MOESM12_ESM.zip › Fig 4/4C/raw images/3 - 2610 - 03.tif]

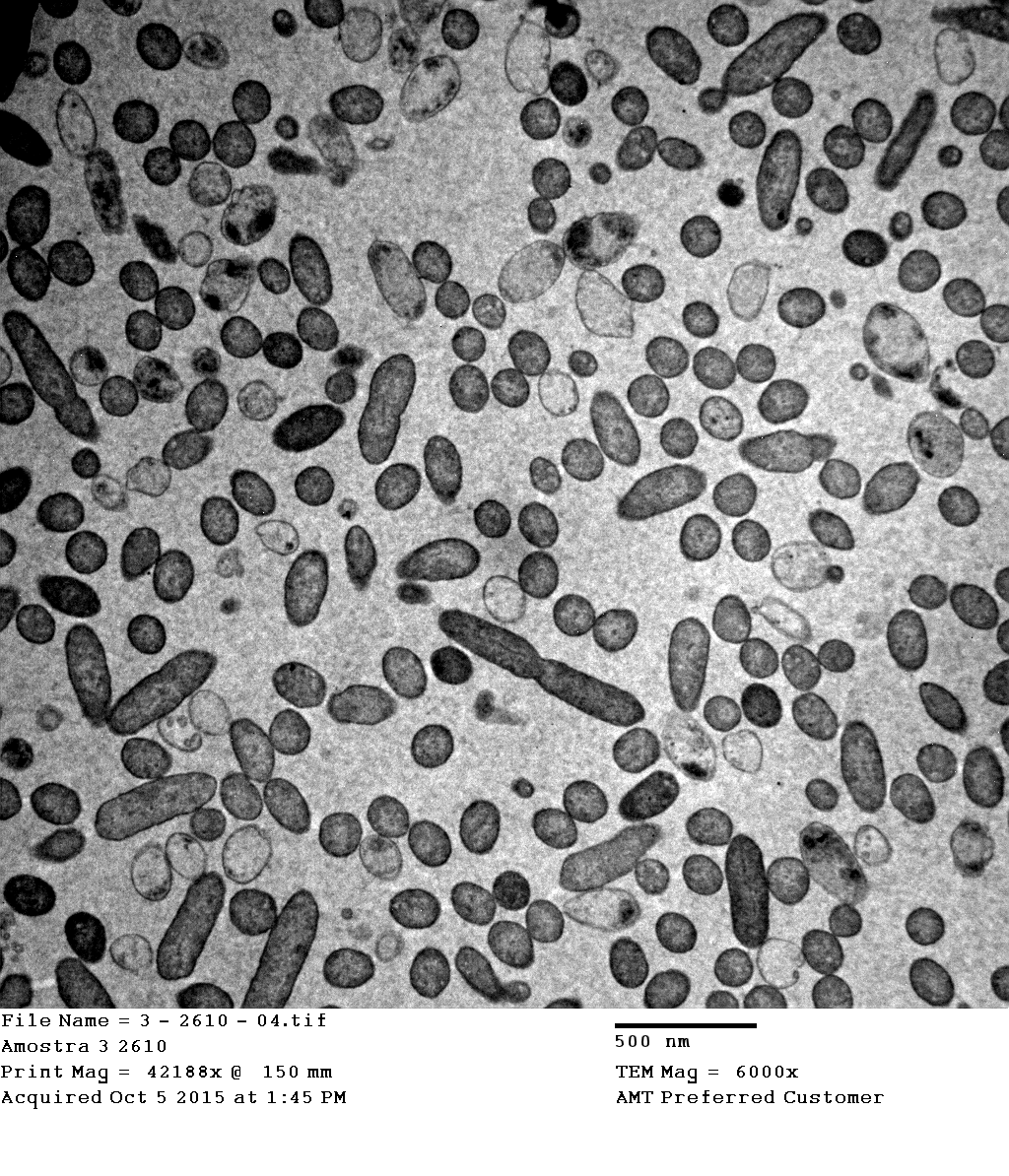

Supplement: Supplementary file 12 — Source Data Fig. 4 [file 44319_2024_60_MOESM12_ESM.zip › Fig 4/4C/raw images/3 - 2610 - 04.tif]

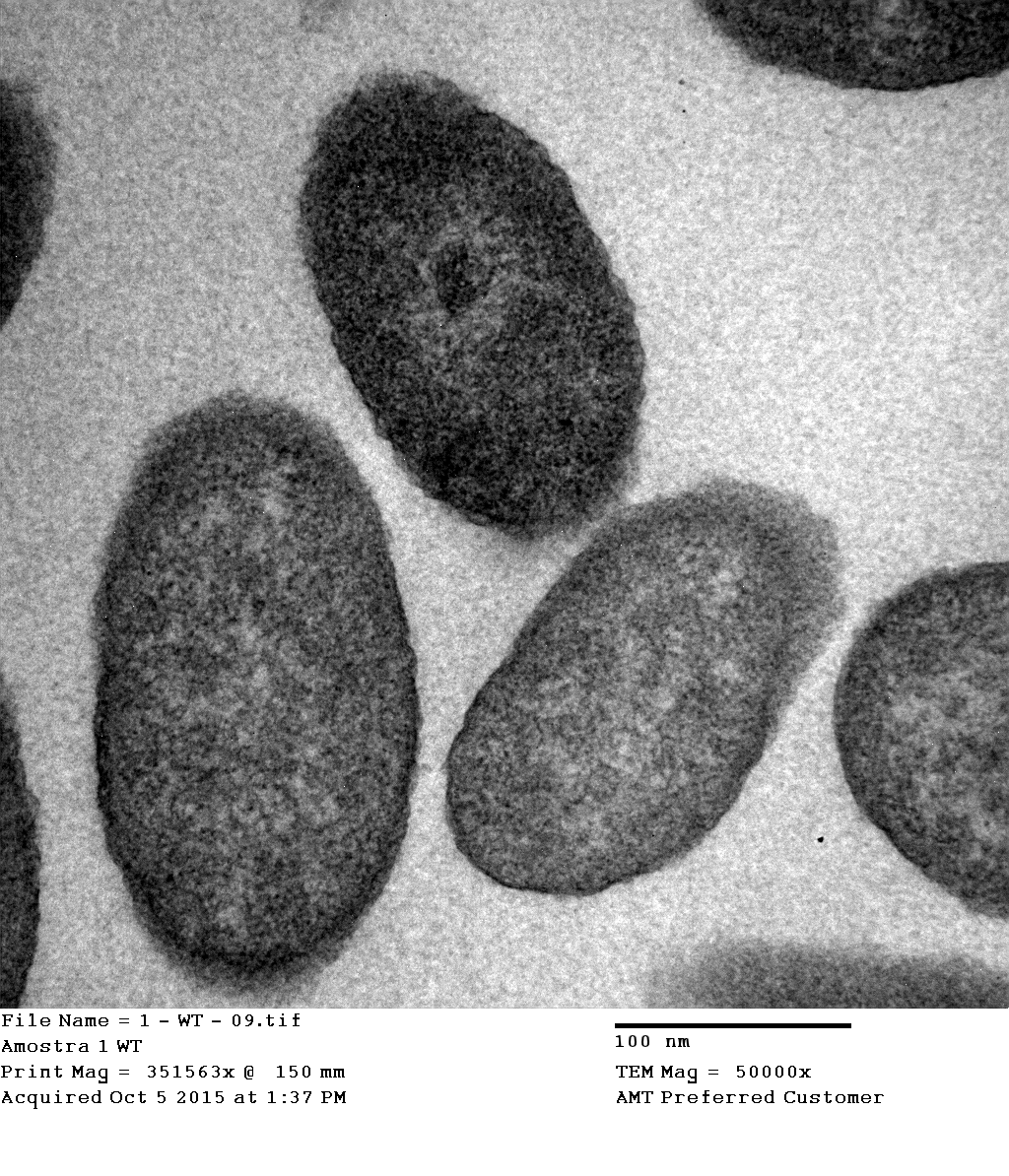

Supplement: Supplementary file 12 — Source Data Fig. 4 [file 44319_2024_60_MOESM12_ESM.zip › Fig 4/4C/raw images/1 - WT - 09.tif]

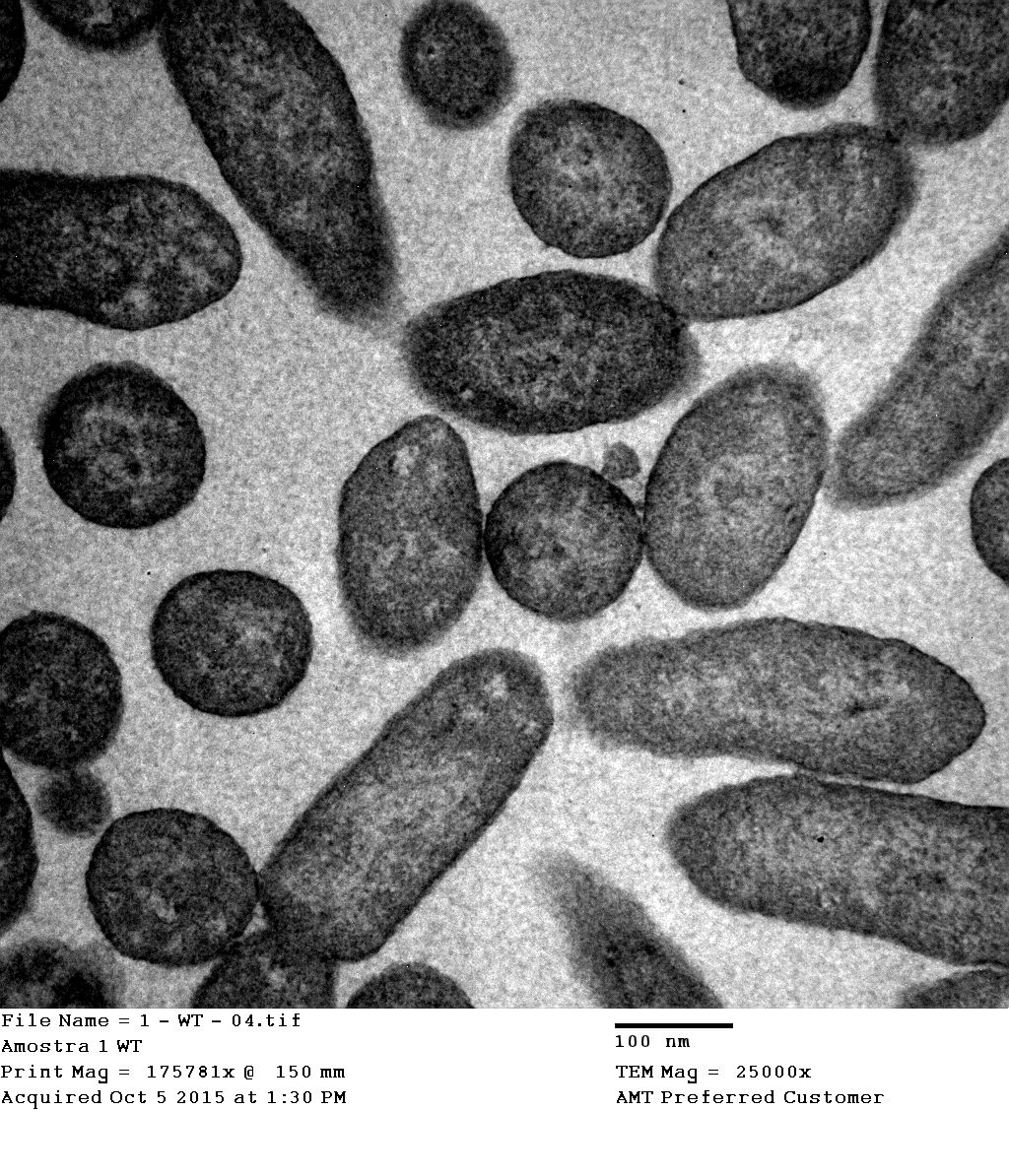

Supplement: Supplementary file 12 — Source Data Fig. 4 [file 44319_2024_60_MOESM12_ESM.zip › Fig 4/4C/raw images/1 - WT - 04.tif]

B

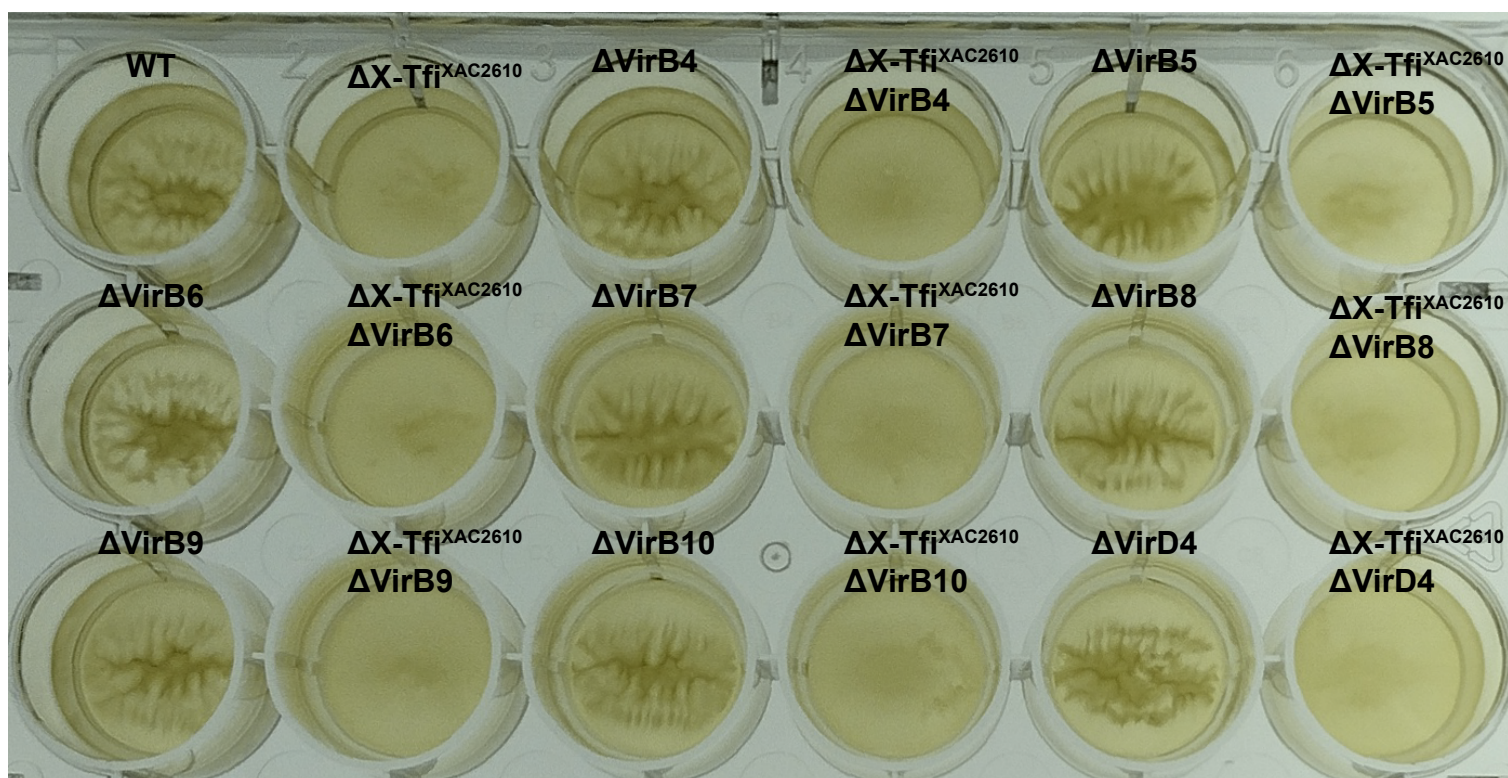

Supplement: Supplementary file 13 — Source Data Fig. 5 [file 44319_2024_60_MOESM13_ESM.zip › Fig 5 no micrographs/5B/Fig5B.pdf]

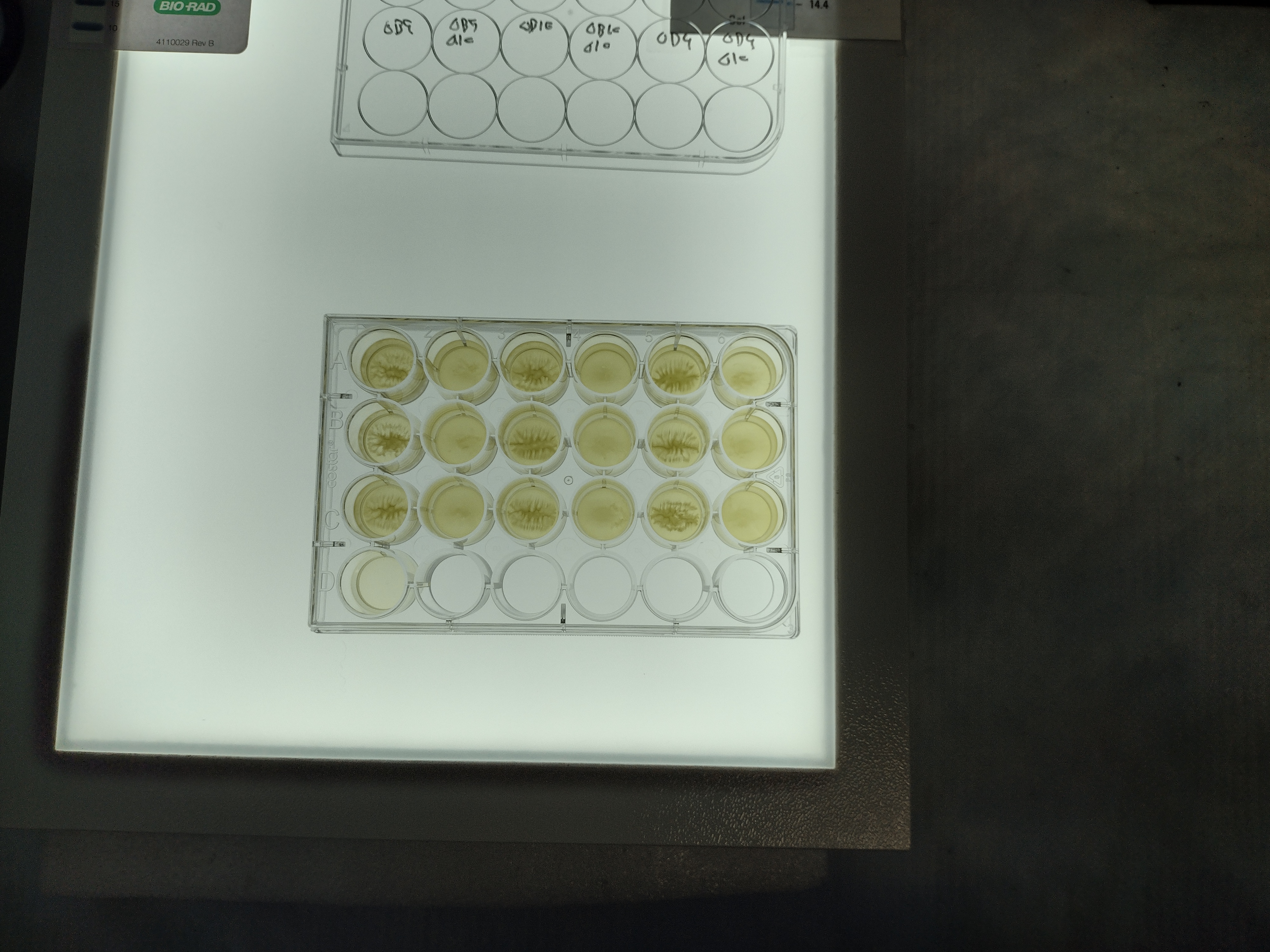

Supplement: Supplementary file 13 — Source Data Fig. 5 [file 44319_2024_60_MOESM13_ESM.zip › Fig 5 no micrographs/5B/Fig5B.jpg]

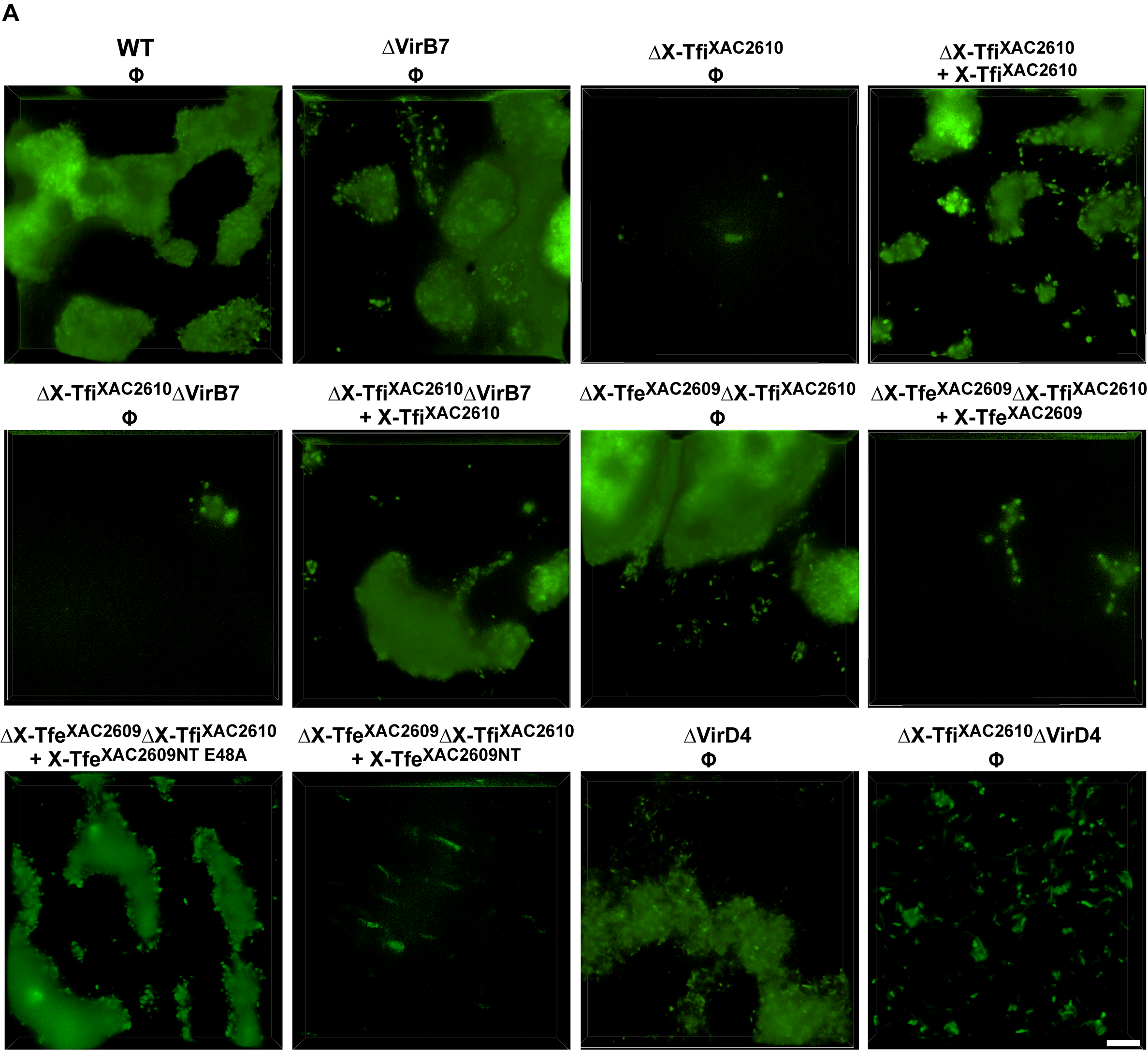

Supplement: Supplementary file 13 — Source Data Fig. 5 [file 44319_2024_60_MOESM13_ESM.zip › Fig 5 no micrographs/5A/Fig5A.tif]

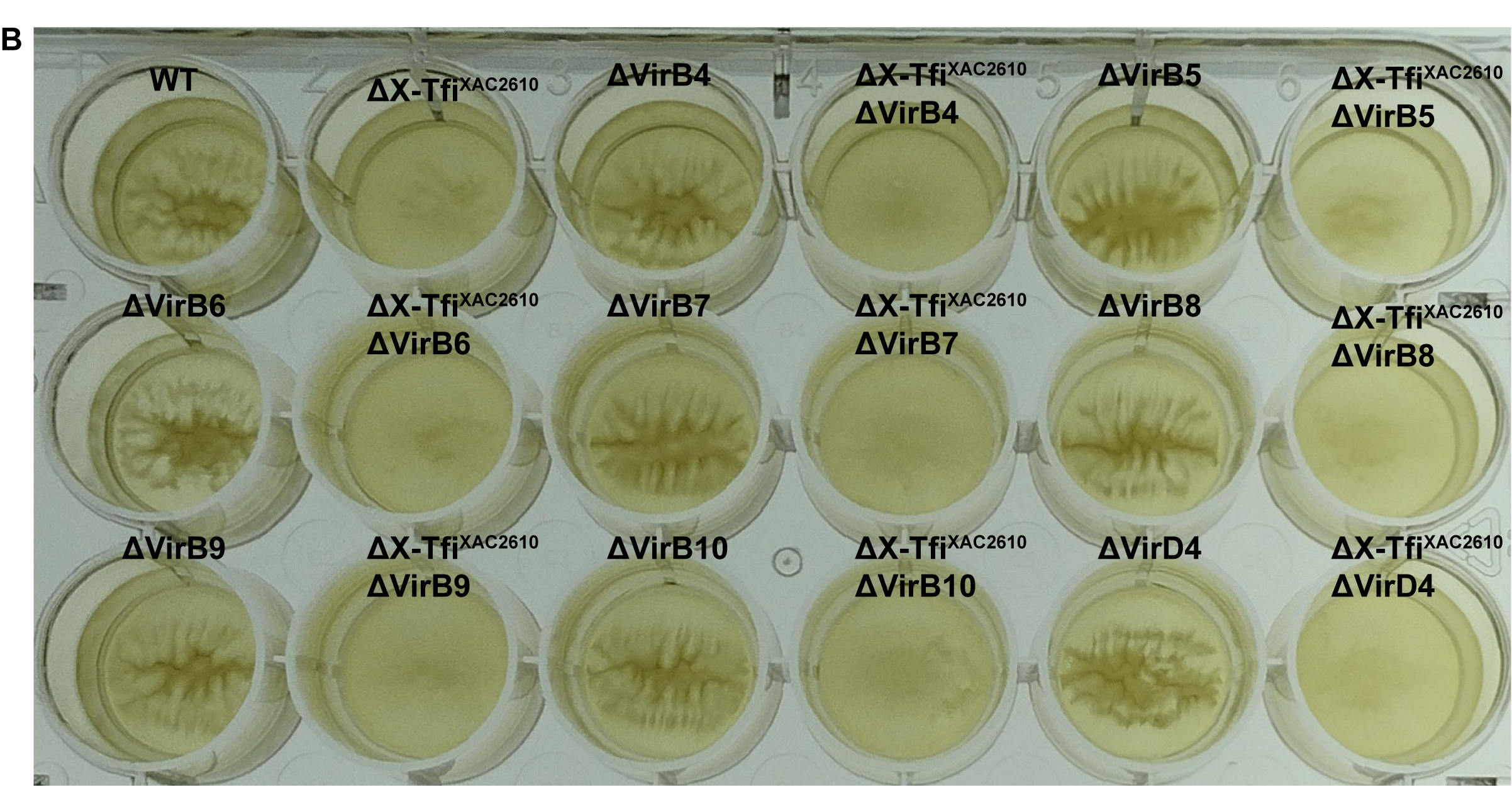

Supplement: Supplementary file 13 — Source Data Fig. 5 [file 44319_2024_60_MOESM13_ESM.zip › Fig 5 no micrographs/5B/Fig5B.tif]

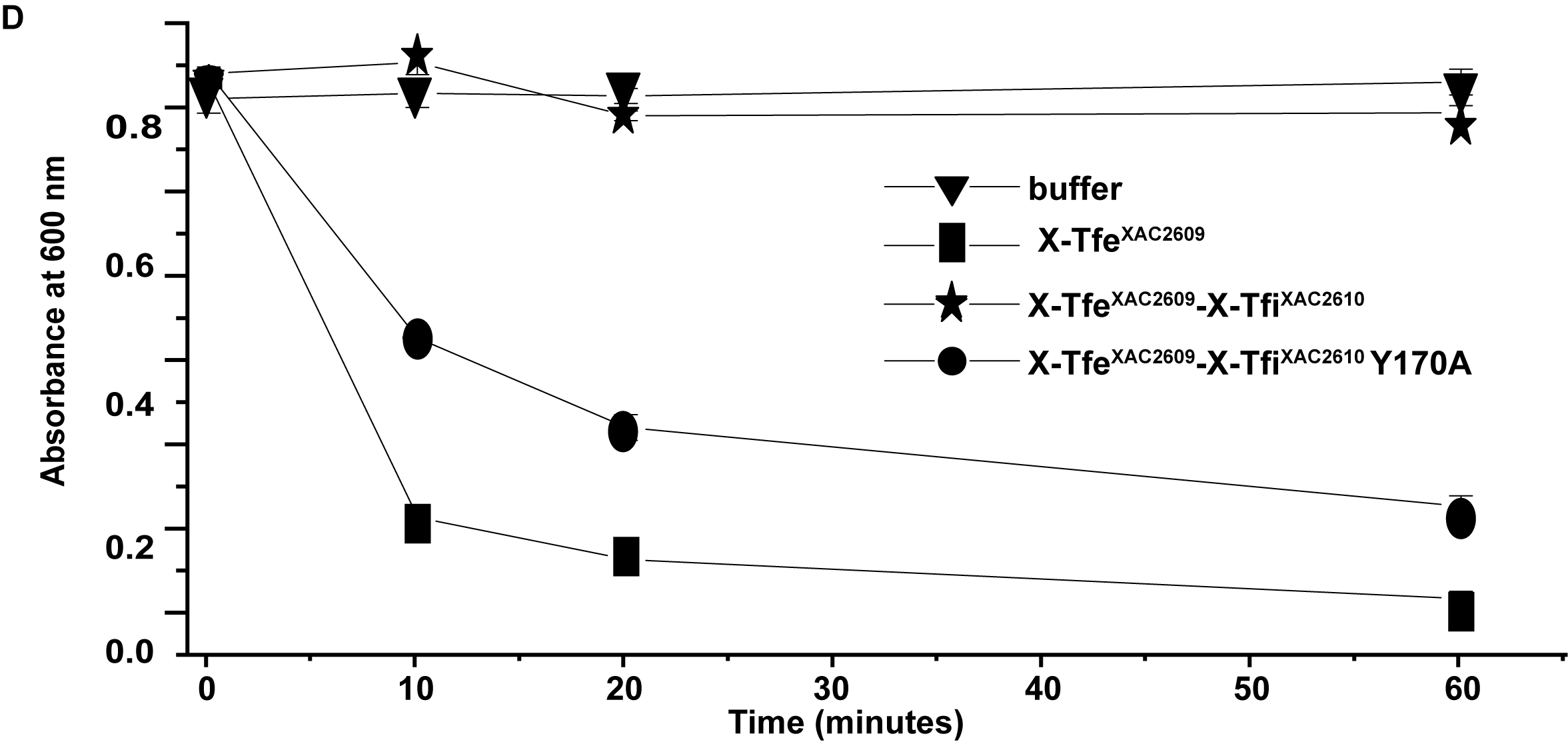

Supplement: Supplementary file 14 — Source Data Fig. 6 [file 44319_2024_60_MOESM14_ESM.zip › Fig 6/6D/Fig6D.tif]

D

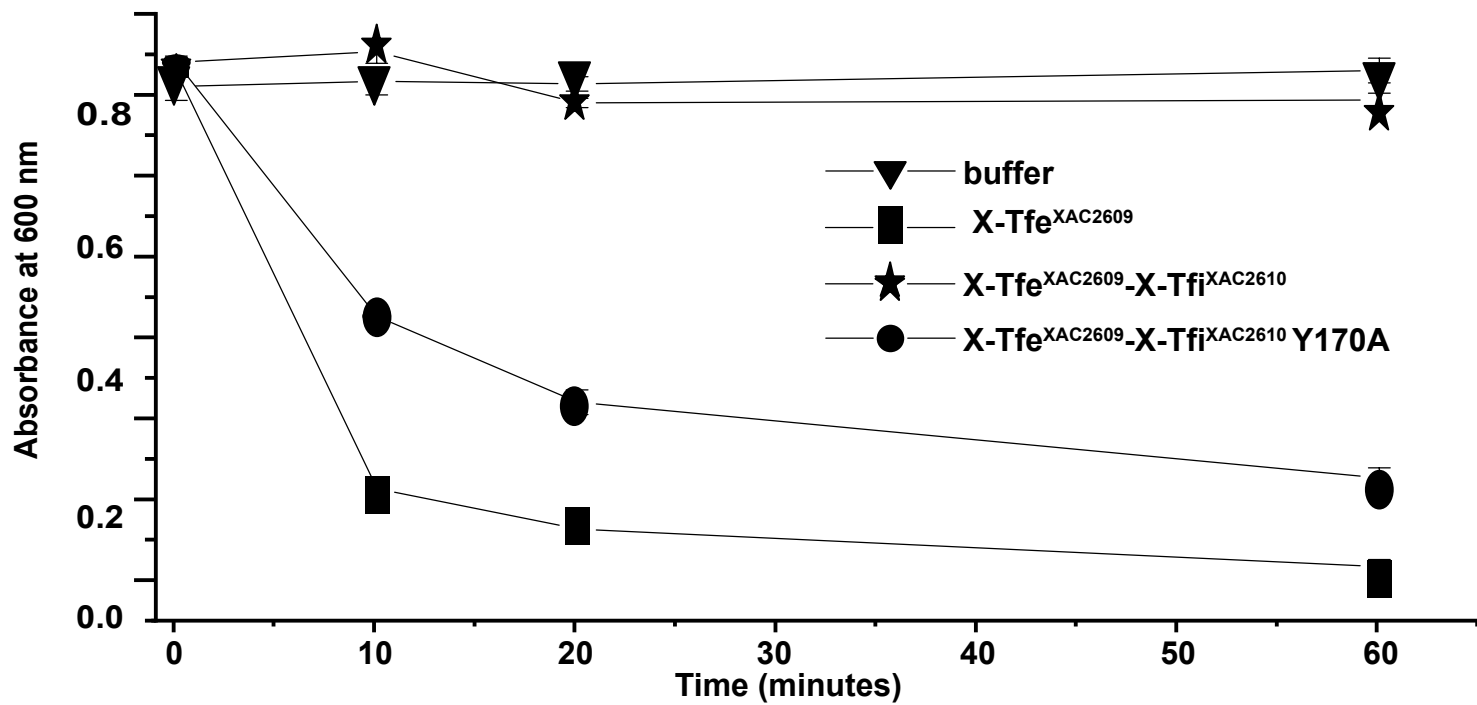

Supplement: Supplementary file 14 — Source Data Fig. 6 [file 44319_2024_60_MOESM14_ESM.zip › Fig 6/6D/Fig6D.pdf]
