# Supplementary material for: Xanthomonas immunity proteins protect against the cis-toxic effects of their cognate T4SS effectors
Source: EMBO Rep. 2024 Feb 8;25(3):27. doi: 10.1038/s44319-024-00060-6 (PMC10933484; doi:10.1038/s44319-024-00060-6)
Supplement: Supplementary file 13 — Source Data Fig. 5 [file 44319_2024_60_MOESM13_ESM.zip › Fig 5 no micrographs/5A/Read me Fig 5A.docx]

The raw data directory for Figure 5A contains Nd2 files that are Z-stacked fluorescence microscopy images obtained at 0.5 microns/step. These files can be opened using the FIJI software. The ND2 files for each strain are named as they appear in the figure. The files can be accessed at <https://www.ebi.ac.uk/biostudies/studies/S-BSST1204>
