# Supplementary material for: Xanthomonas immunity proteins protect against the cis-toxic effects of their cognate T4SS effectors
Source: EMBO Rep. 2024 Feb 8;25(3):27. doi: 10.1038/s44319-024-00060-6 (PMC10933484; doi:10.1038/s44319-024-00060-6)
Supplement: Supplementary file 13 — Source Data Fig. 5 [file 44319_2024_60_MOESM13_ESM.zip › Fig 5 no micrographs/5B/readme Fig 5B.docx]

Raw photographic images of the 24 well plates shown in Fig 5B.
