# Supplementary material for: Xanthomonas immunity proteins protect against the cis-toxic effects of their cognate T4SS effectors
Source: EMBO Rep. 2024 Feb 8;25(3):27. doi: 10.1038/s44319-024-00060-6 (PMC10933484; doi:10.1038/s44319-024-00060-6)
Supplement: Supplementary file 13 — Source Data Fig. 5 [file 44319_2024_60_MOESM13_ESM.zip › Fig 5 no micrographs/5A/Fig5A.pdf]

A

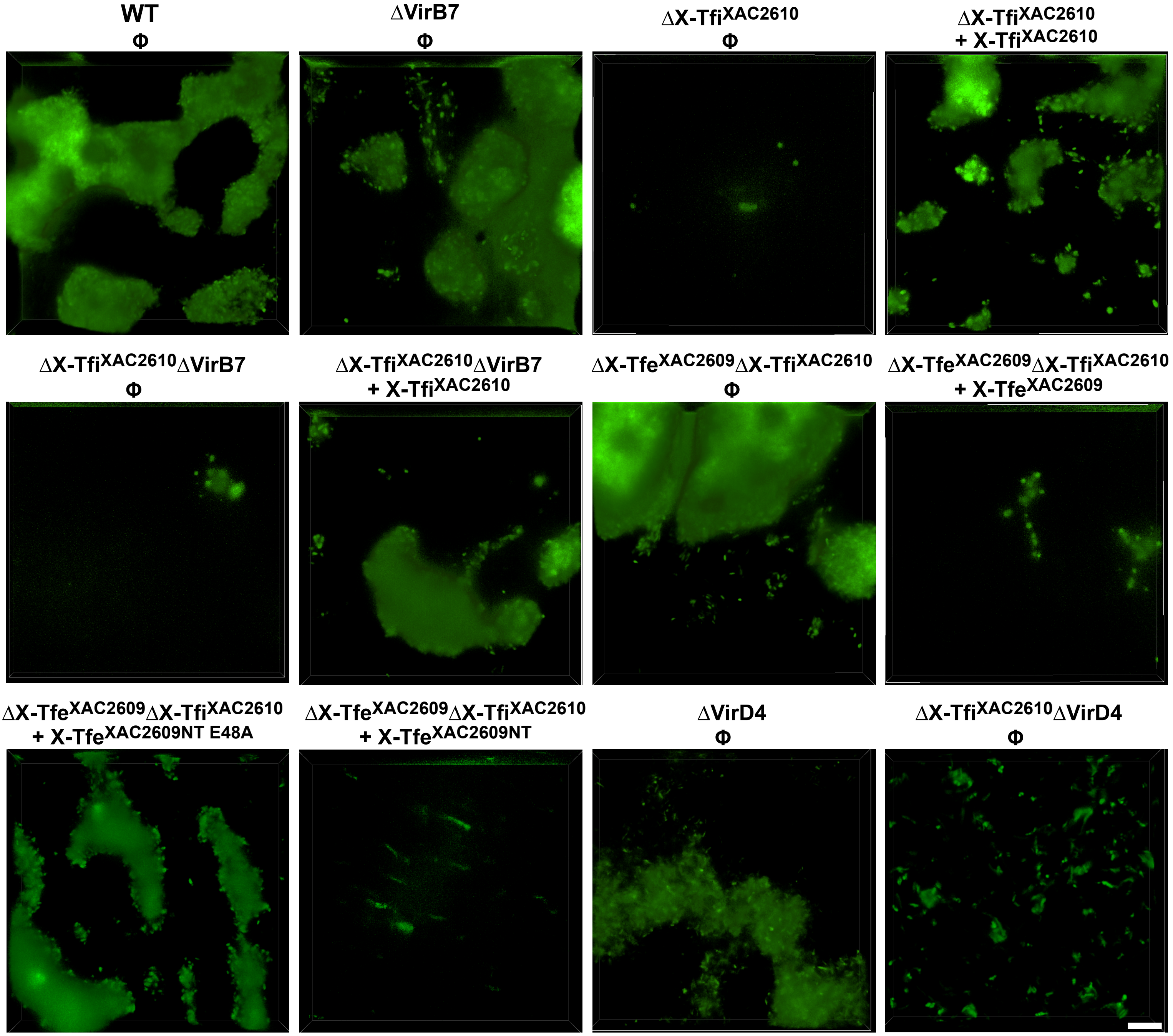
WT
Φ
ΔVirB7
Φ
ΔX-TfiXAC2610
Φ
ΔX-TfiXAC2610
+ X-TfiXAC2610
ΔX-TfiXAC2610ΔVirB7
Φ
ΔX-TfiXAC2610ΔVirB7
+ X-TfiXAC2610
ΔX-TfeXAC2609ΔX-TfiXAC2610
Φ
ΔX-TfeXAC2609ΔX-TfiXAC2610
+ X-TfeXAC2609
ΔX-TfeXAC2609ΔX-TfiXAC2610
+ X-TfeXAC2609NT E48A
ΔX-TfeXAC2609ΔX-TfiXAC2610
+ X-TfeXAC2609NT
ΔVirD4
Φ
ΔX-TfiXAC2610ΔVirD4
Φ
