# Supplementary material for: Xanthomonas immunity proteins protect against the cis-toxic effects of their cognate T4SS effectors
Source: EMBO Rep. 2024 Feb 8;25(3):27. doi: 10.1038/s44319-024-00060-6 (PMC10933484; doi:10.1038/s44319-024-00060-6)
Supplement: Supplementary file 14 — Source Data Fig. 6 [file 44319_2024_60_MOESM14_ESM.zip › Fig 6/6D/readme Fig6D.docx]

Numerical data file contains replicates (r1,r2,r3). mean and SD of peptidoglycan lysis assays assays using *M. luteus* cell walls as substrate and monitored by measuring optical density at 600 nm.

Time course at zero, 10 minutes, 20 minutes, and 1 hour.

Rows and columns are named according to the figure legend.
