# Supplementary material for: Xanthomonas immunity proteins protect against the cis-toxic effects of their cognate T4SS effectors
Source: EMBO Rep. 2024 Feb 8;25(3):27. doi: 10.1038/s44319-024-00060-6 (PMC10933484; doi:10.1038/s44319-024-00060-6)
Supplement: Supplementary file 16 — Expanded View Figures [file 44319_2024_60_MOESM16_ESM.pdf]

Expanded View Figures

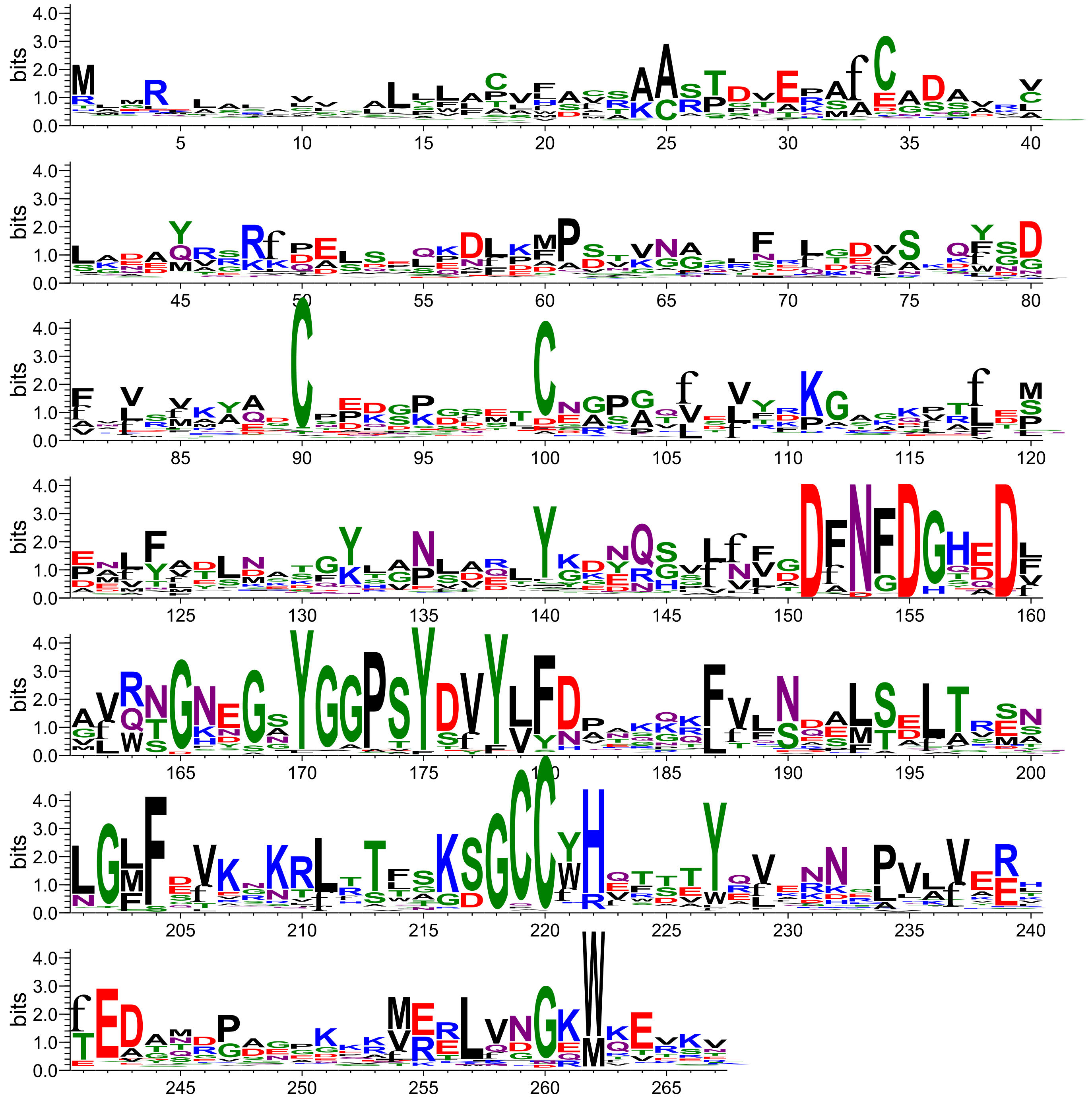

Figure EV1. Sequence conservation profile of the multiple alignment of X-Tfi^{XAC2610} homologs.

The sequences of 429 X-Tfi^{XAC2610} homologs (GenBank code AAM37459/refseq WP_011051709.1) were obtained as described in Material and Methods and are listed in Dataset EV1. The sequence conservation profile was generated using the Weblogo3 server (Crooks et al, 2004). Stacking height indicates sequence conservation at each position while the symbol within the stack indicates the relative frequency of each amino acid in that position. The numbering below the profile corresponds to the amino acid sequence of X-Tfi^{XAC2610}. The conserved motif from residues 151–159 corresponds to the Ca^{2+}-binding loop observed in the X-Tfi^{XAC2610} crystal structure (Souza et al, 2015). The conserved tyrosine at position 170 is found within a loop predicted to insert into the active site of the cognate effector as described in the main text.

© The Author(s)

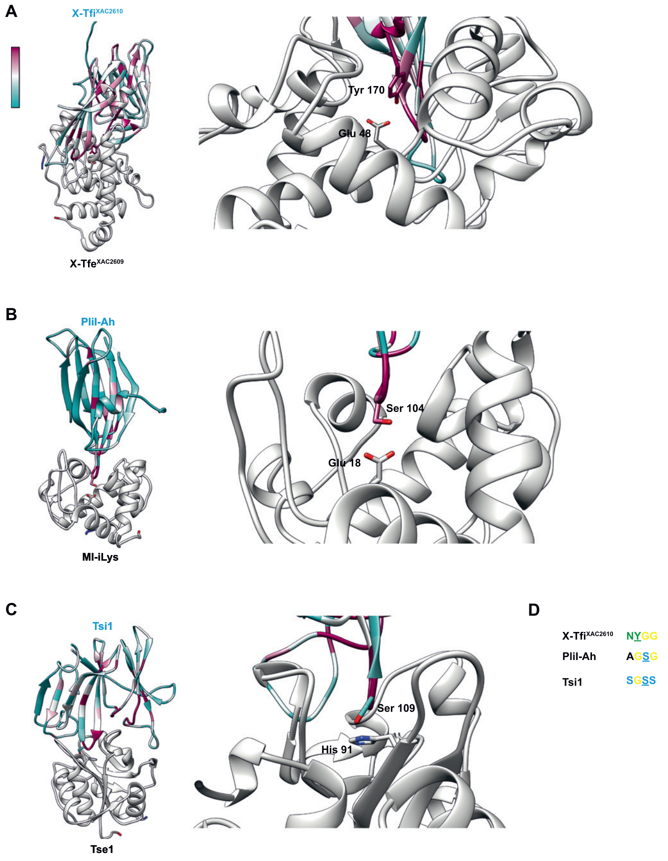

Figure EV2. Comparison of the model of the X-Tfe^{XAC2609}(1–194)-X-Tfi^{XAC2610}(54–267) complex with the crystal structures of the PliI-Ah - i-type lysozyme and Tsi1-Tse1 complexes.

(**A–C**) Left panels: Ribbon representation of the (**A**) X-Tfe^{XAC2609}-X-Tfi^{XAC2610} model generated by Alphafold2. (**B**) the crystal structure of the periplasmic i-type lysozyme inhibitor from *Aeromonas hydrophila* (PliI-Ah) in complex with the i-type lysozyme from *Meretrix lusoria* (Ml-iLys) (PDB 4PJ2; (Herreweghe et al, 2015) and (**C**) the crystal structure of Tsi1-Tse1 complex from (PDB 3VPJ) (Ding et al, 2012). (**A–C**) The inhibitors are colored according to the degree of conservation at each residue position in the corresponding family of homologs (lowest, cyan; highest, purple) and the cognate enzymes are colored in gray. (**A–C**) Right panels: Zoom of the interaction interfaces showing the insertion of the inhibitory loops into the active sites of the enzymes. Stick models highlight the interactions between residues in the inhibitory loops (X-Tfi^{XAC2610} Y170, PliI-Ah S104 and Tsi1 S109) and the enzyme active sites (X-Tfe^{XAC2609} E48, Ml-iLys E18, Tse1 H91). (**D**) Sequences in the inhibitory loops of X-Tfi^{XAC2610}, PliI-Ah and Tsi1 that interact directly with the catalytic site of the corresponding toxins. Underlined are X-Tfi^{XAC2610} Y170, PliI-Ah S104 and Tsi1 S109.

 © The Author(s)

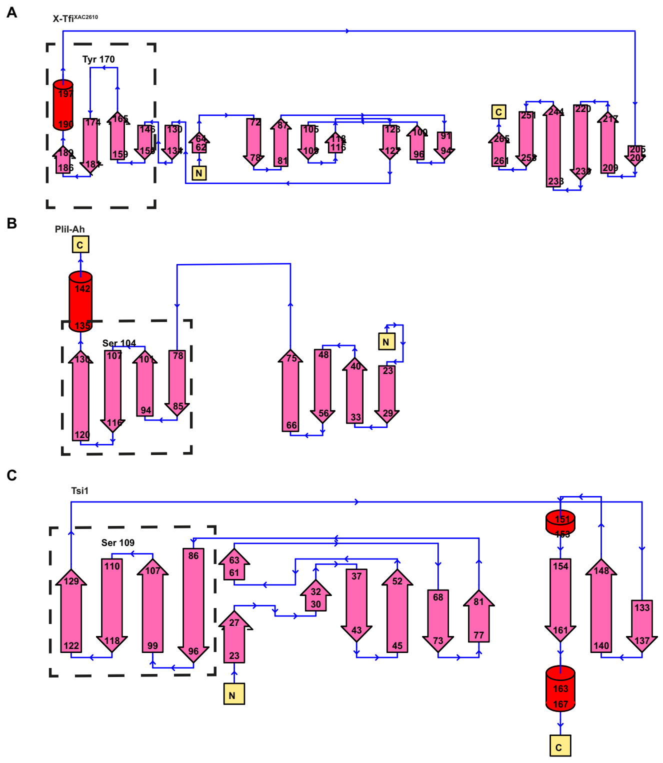

Figure EV3. Protein topology diagrams of X-Tfi[XAC2610], Pli-Ah and Tsi1.

Diagrams were generated using the PDBsum server (http://www.ebi.ac.uk/thornton-srv/databases/cgi-bin/pdbsum/GetPage.pl?pdbcode=index.html). Secondary-structure elements are indicated as red cylinders (α-helices) and pink arrows (β-strands). The dotted-squares highlight the common β-sheet found in the three immunity proteins containing a loop between the second and third β-strands that inserts into the active site of the cognate enzyme. (**A**) X-Tfi[XAC2610] (PDB 4QTQ). (**B**) Pli-Ah (PDB 4PJ2). (**C**) Tsi1 (PDB 3VPJ).

© The Author(s)
